# Comparative Genomic Analysis of the DUF34 Protein Family Suggests Role as a Metal Ion Chaperone or Insertase

**DOI:** 10.3390/biom11091282

**Published:** 2021-08-27

**Authors:** Colbie J. Reed, Geoffrey Hutinet, Valérie de Crécy-Lagard

**Affiliations:** 1 Department of Microbiology and Cell Science, University of Florida, Gainesville, FL 32611, USA; creed212@ufl.edu (C.J.R.); ghutinet@ufl.edu (G.H.); 2 Genetics Institute, University of Florida, Gainesville, FL 32611, USA

**Keywords:** comparative genomics, metabolic reconstruction, bioinformatics, conserved unknowns, function prediction, functional annotation, orthology

## Abstract

Members of the DUF34 (domain of unknown function 34) family, also known as the NIF3 protein superfamily, are ubiquitous across superkingdoms. Proteins of this family have been widely annotated as “GTP cyclohydrolase I type 2” through electronic propagation based on one study. Here, the annotation status of this protein family was examined through a comprehensive literature review and integrative bioinformatic analyses that revealed varied pleiotropic associations and phenotypes. This analysis combined with functional complementation studies strongly challenges the current annotation and suggests that DUF34 family members may serve as metal ion insertases, chaperones, or metallocofactor maturases. This general molecular function could explain how DUF34 subgroups participate in highly diversified pathways such as cell differentiation, metal ion homeostasis, pathogen virulence, redox, and universal stress responses.

## 1. Introduction

Protein families that are both highly conserved across domains of life and poorly characterized are referred to as conserved unknowns [1,2]. Though recent studies that use comparative genomics [3,4], classical genetics [5] and/or biochemistry [6,7] approaches have solved a few of these “orphan” family puzzles, their number remains high [1,8,9,10,11,12]. One of the issues is that, because these conserved proteins often harbor core functional roles, genetic approaches lead to pleiotropic phenotypes, making the elucidation of a precise molecular function quite difficult. For example, the COG0533 and COG0009 proteins involved in the synthesis of the universal tRNA modification threonylcarbamoyladenosine (t^6^A) [13,14,15], were first thought to be involved in protein degradation [16,17], transcriptional regulation [18], or cell division [14]. Similarly, RidA (reactive intermediate deaminase A), a subgroup within the Rid family of proteins (members also have been referred to as YjgF/YER057c/UK114), was a notable challenge for functional characterization due to the multiple and complex phenotypes associated with mutations in genes of this family in different organisms [19,20,21,22,23].

The DUF34/NIF3 protein family is reportedly ubiquitous, with members found in model organisms such as *Homo sapiens* (NIF3L1), *Mus musculus* (Nif3l1), *Saccharomyces cerevisiae* (Ngg1-interacting Factor 3/NIF3) [24,25], *Escherichia coli* (YbgI) [26] and *Bacillus cereus* (YqfO) [27]. Despite its conservation, the precise function(s) of members of this family remain undetermined. More than a decade has passed since the family was first formally identified as a target for characterization [24] and even longer since the gene encoding a homolog of NIF3 in *S. cerevisiae* was first described in *Drosophila melanogaster* [28,29]. Since, it has been linked to a variety of functions across superkingdoms and several diseases in humans (e.g., juvenile amyotrophic lateral sclerosis, Williams–Beuren Syndrome [30,31], among many others). The role of this protein family remains mysterious, even with recent studies trying to more proximately decipher its function in *E. coli* [32]. Automated annotation databases indicate that the human DUF34 family member, NIF3L1, is highly connected, for example listing 4178 functional associations for its entry in the Harmonizome database (i.e., 65 datasets, electronically extracted; https://amp.pharm.mssm.edu/Harmonizome/gene/NIF3L1 [33]; accessed on 22 June 2021). In addition, an annotation based on a single set of in vitro results examining the NIF3 homolog of *Helicobacter pylori* (HP0959) [34] led to the swift percolation of the annotation, “GTP cyclohydrolase I type 2 homolog”, throughout many databases, including UniProtKB. This annotation as the first enzyme of tetrahydrofolate biosynthesis is certainly incorrect for the whole protein family, as DUF34 members are found in folate auxotrophs such as *Mycoplasma* [35,36,37].

A comprehensive analysis of the literature was conducted to catalog all published knowledge for DUF34 family members, an endeavor that cannot be easily conducted using only simple PubMed searches, as many studies do not mention general family names of genes/proteins for which data has been generated, often only citing species- or system-specific gene names. In parallel, an extensive comparative genomic analysis was performed to investigate the validity of “GTP cyclohydrolase I type 2”, a dubious annotation widespread among DUF34 family members, and to ultimately propose a unifying functional role for the family as a metal insertase. With this, it was possible to divide the DUF34 protein family into subgroups by distinctions in structure, complete domain architecture, regulation, occurrence, localization, and functional associations. 

## 2. Materials and Methods

### 2.1. Capture of Literature, Structural, and Essentiality Data

The strategy used to compile published literature for members of the DUF34 family is detailed in the Supplemental Methods and all websites used, both here and in subsequent analyses, are listed in Supplemental Table S1. Most of the public search engines/web crawlers, and searchable libraries/depositories used required text as input while more specialized tools leveraged the use of protein sequences (e.g., PaperBLAST [38]). Protein Data Bank (PDB; RCSB PDB, Research Collaboratory for Structural Bioinformatics PDB)) was used to evaluate and acquire protein crystal structures and respective sequences, related literature, and relevant data files for subsequent search and analysis [38,39,40]. Structures were edited, aligned using PyMol (Edu PyMol, Schrödinger, Inc., New York, New York, USA, Educational edition). MetalPDB was used to survey ions present, indicated, or predicted to complex with published protein crystal structures [41,42].

Essentiality data was acquired using multiple different sources listed in Table S1. The BLAST search tool of DEG (Database of Essential Genes) [43] was used, with *H. sapiens* (NIF3L_HUMAN, Q9GZT8), *Methanocaldococcus jannaschii* (GCH1L_METJA, Q58337), *B. cereus* (Q818H0_BACCR, Q818H0), and *E. coli* (GCH1L_ECOLI, P0AFP6) as inputs. Ogee [44] was used to collect additional essentiality data through the browse function. Predicted essentiality data for *Mycoplasma* species were acquired using pDEG (Database of Predicted Essential Genes) [45].

### 2.2. Domain Analysis

The first set of sequences of DUF34 family members from model organisms was extracted using OrthoInspector 3.0 (accessed on 30 January 2020; iCube Laboratory, Illkirch-Graffenstaden, France) [46] using the following input sequences for retrieving sets of sequences per superkingdom: NIF3L_HUMAN (Q9GZT8), GCH1L_METJA (Q58337), and GCH1L_ECOLI (P0AFP6). An additional set of sequences from organisms with published data was extracted from UniProtKB [47] to generate a non-redundant list of 219 sequences to be used in subsequent analyses. The sequences of the corresponding DUF34 proteins were not available for a few organisms with which publications were associated. For *Desulfovibrio desulfuricans*, sequences of the closely related *Desulfovibrio alaskensis G20* were used, and those of *Schistosoma mansoni* were used in the place of *Schistosoma mekongi.* Although described in their respective publications, sequences for DUF34 family members could not be retrieved for three organisms: *Idiosepius paradoxus, Streptomyces sp. SN-1061M, Verrucomicrobium (Termite Associated, TAV) sp. strain 2*. Sequences were aligned using MAFFT (E-INS-i, default settings) [48,49,50]. Motif and domain logos were generated through the use of the WebLogo web server [51]. Sequence logos were manually aligned using Inkscape [52]. 

### 2.3. Absence-Presence, Phyletic Patterns & Homolog/Paralog Co-Occurrence

Species trees were generated with PhyloT (database version 2020.2) and iToL [53]. Absence-presence data was acquired, both, through manual curation using advanced searches of common databases (i.e., UniProt, NCBI [54]), subsequent BLAST validation, as well as the use of phyletic patterning tools available through MicrobesOnline (accessed on 7 June 2019) [55] and STRING (v11, released 19 January 2019) [56]. Paralogs were identified using EggNOG (EggNOG 5.0, EMBL, Heidelberg, Germany) [57] and KEGG Paralog Search (KEGG release 94.1, Kyoto University, Kyoto, Japan) [58]. 

### 2.4. Physical Clustering Analysis

Physical clustering data was acquired from Gene Context Tool NG (GeConT 3) of the Computational Genomic Group, IBT–UNAM, using the central orthologous group ID known for the DUF34 family, COG0327 (accessed on 3 May 2020) [59] and analyzed using a text-mining strategy we developed and termed Physical Clustering Keyword Frequency Analysis (PCKFA). This approach as well as the further annotation of a subset of families are described in detail in the Supplemental Methods (1.2). 

### 2.5. Coexpression, Covariation Data Acquisition & Enrichment Analysis

Lists of 300 genes co-expressed with DUF34 family members were retrieved for all 10 eukaryotic model organisms available using CoXPresDb (gene sets excluded respective DUF34 homologs) [60], except for *Caenorhabditis elegans,* which does not encode for a DUF34 family member. Protein covariation data for *Homo sapiens* was acquired using the ProteomeHD webserver (unsupervised query format) [61]; a threshold of 0.98 was used for data retrieval for NIF3L1 (specific protein reference ID within the database: Q9GZT8-2, resulting in 114 total covarying proteins). Gene set enrichment analyses (GSEA), was performed using two tools: g:GOSt (via g:Profiler web server, Bioinformatics, Algorithmics and Data Mining Group, University of Tartu, Tartu, Estonia) [62], and the functional annotation clustering tool (via DAVID bioinformatic suite, Frederick National Laboratory, Frederick, Maryland, USA) [63,64,65]. UniProtKB was used to map UniProt IDs to the Entrez Gene IDs of eukaryotic datasets prior to GSEA. If electronic mapping failed for a human identifier, the HGNC database was used in manual retrieval (HUGO Gene Nomenclature Committee at the European Bioinformatics Institute [66]). If mapping failed the “reviewed” entries in UniProtKB were selected over the “unreviewed” duplicates and/or isoforms listed.

### 2.6. Fusion Analysis

To analyze fusions present in the DUF34 family, the protein family members as defined by UniProt (e.g., “GTP cyclohydrolase I type 2/NIF3 family”) were exported and filtered for all sequences containing InterPro HMM profile signature annotations distinct from those already recognized in Results Section 3.5. To optimize coverage of all documented fusions, the second and third approaches for curating such homologs were implemented in parallel to the UniProt-dependent approach. For these two complementary methods, sequences of various domain architectures were directly exported from Pfam (PF01784) and InterPro (IPR036069), independently. Three lists of homologs generated by each method were concatenated and duplicate sequences removed. Fusions identified via the preceding literature review were added, defining the final collection of “noncanonical” homologs. All fusion/arrangement types were further evaluated for legitimacy through manual curation (i.e., comparative annotation review of the genome and sequence features) and the assignment of confidence scores: “valid” (highest confidence); “valid, conditional”; “conditional”/“conditional, singleton”; “inconclusive”; “invalid” (lowest confidence, no validity). To ensure results of fusion analyses were comparable to those of other bioinformatics presented, singularly representative COGs and COG descriptions were assigned to the final list of exceptional homologs using CDD Search, subsequently cross-referencing results with EggNOG records for optimal domain descriptions. For more information on data transformation, amendment, and clean-up, see Supplemental Methods (1.3).

### 2.7. Strain Construction & List

All strains and oligonucleotides used in this study are listed in Table S2. Two genes of *E. coli*, *ybgI* (encoding for DUF34) and *folE* (encoding for GTP cyclohydrolase I type 1) were cloned independently in pBAD24 between NcoI and SbfI following PCR amplification by Phusion^®^ High-Fidelity DNA Polymerase (New England Biolabs, Ipswitch, MA, USA, NEB) using GO285 and GO286 oligonucleotides for *ybgI*, while GO434 and GO435 were used for *folE*. After verification by sequencing, the plasmids generated were renamed “pGH50” and “pGH101”, respectively.

The *ybgI::Kan^R^ E. coli* mutant came from the Keio Collection [67], while the *folE::Kan^R^* had been previously constructed [68]. These mutations were transduced by P1vir into *E. coli K-12 MG1655*. The *ybgI* and *folE* double mutant were obtained by first flipping out the kanamycin cassette from the *ybgI* mutant using pCP20 [69], subsequently transducing the *folE::Kan^R^* mutation using P1vir. Mutation verifications were performed by oneTaq PCR (NEB) using a set of primers internal and external to the gene (GO563 to GO570). Each plasmid, including empty pBAD24, was individually transformed into the control strain and each mutant. Strains were grown at 37 °C using LB supplemented with glucose 0.2%, kanamycin sulfate 50 µg/mL, or ampicillin 100 µg/mL when necessary for selection. 2′-deoxythymidine (dT) 0.3 mM was used for *folE* mutants.

### 2.8. dT Sensitivity Assay

Strains (WT, single mutants, and double mutants) were grown overnight at 37 °C in LB supplemented with glucose 0.2%, kanamycin sulfate 50 µg/mL (except for WT), and dT 0.3 mM. Each strain was inoculated in various LB with or without dT 0.3 mM at an OD_600nm_ of 0.1 and grown at 37 °C in a bioscreen (Oy Growth Curves Ab Ltd., Turku, Varsinais-Suomi, Finland) for 40 h. This experiment was completed in quintuplicate.

### 2.9. dT Essentiality Complementation Assay

Strains containing pBAD24 variations were grown overnight at 37 °C in LB supplemented with glucose 0.2%, ampicillin 100 µg/mL and dT 0.3 mM. They were then normalized to an OD_600nm_ of 1.0 in LB, and a 5 µL drop was streaked on LB agar containing ampicillin 100 µg/mL, either glucose or arabinose at 0.2%, and either with or without dT 0.3 mM. These plates were left to grow for 10 h at 37 °C. This experiment was performed in triplicate.

## 3. Results and Discussion

### 3.1. Extensive Literature Capture and Analysis Confirms Pleiotropic Role of DUF34 Family Members

While the earliest mention of the family dates back to 1996 when the binding of a yeast homolog to NGG1/ADA3 via a GAL4 fusion domain was noted [70], the first dedicated description of a DUF34 family member was published in 2000 with the isolation and characterization of the human NIF3L1 and its mouse homolog [30]. Only seven papers in PubMed cite the latter study (per 6 June 2021) and 20 mostly unrelated publications cite the former (as of 6 June 2021; studies focused mostly on NGG1/ADA3 or SAGA complex, only 6 demonstrating relevance to DUF34). PaperBLAST, a sequence-based literature search tool, searches titles, abstracts, and full publication texts available through Europe PMC [71]. As PaperBLAST searches only open-source texts, we expanded our search using a cyclic approach described in Supplemental Methods Section (1.1). A final collection of sequences and keywords used for sequence-/text-based searches can be found in Data Table S1. The resulting list of curated publications was divided into two groups: “focal” (i.e., homolog mentioned in title or abstract; Table 1) and “non-focal’’ (i.e., mention occurs in other publication sections or supplemental/attached files). The complete collection of focal/non-focal publications is reported in Data Table S2. All individual DUF34 family members with publications are listed in Table S3. Using this integrative search approach, the ultimate total of reference terms reached upwards of 857 and provided DUF34 member-relevant data for ~100 unique organisms. This process increased the total number of DUF34 protein family-relevant papers from < 30 when using a simple PubMed search with the following query, [“DUF34” OR “NIF3” OR “NIF3L1” OR “YbgI” OR “YqfO”], to 333 distinct publications using the iterative approach.

Although the captured data covered all superkingdoms, the distribution of publication counts skewed largely toward bacteria, this domain having the greatest number of “non-focal” publications and, thereby, total publications overall. In contrast, work examining eukaryotic systems contributed the greatest proportion of “focal” publications. Only one “non-focal” publication featured a viral homolog. No publications were found to describe DUF34 family members for any species of plant (*Viridiplantae*), consistent with the absence of DUF34 homologs among annotated plant genomes discussed below.

To discern whether any common functional associations could be extracted from the final DUF34 corpus, word clouds were generated using publication titles of both focal and non-focal publications (Data Table S2, Figure S1). The resulting diagrams predominantly emphasized the systems of study (e.g., “Mycobacterium”, “Escherichia”, “Bacillus”, “yeast”) and terms relating to the characterization process (e.g., “reveal”, “novel”, “analysis”, “functional”, “identifies”, “associated”), both of which observations provided little insight into a specific function. However, other less pronounced keywords were indicative of more specific biological contexts, such as “mitochondrial”, “DNA repair”, “DNA methylation”, “[Fe]-hydrogenase cofactor biosynthesis”, “stress”, “virulence”, “heat”, “resistance”, and “secreted”, for example. Together, these diagrams illustrated that, of the surveyed literature, themes of bacterial pathogen virulence, gene regulation, cell signaling pathways, stress response, as well as metal ion metabolism and related membrane homeostasis, seemed to be emphasized. 

Across published data, differences in the localization of DUF34 proteins are reported with no clear consensus. In fungi, for example, family members have been linked to mitochondria (e.g., P53081, *Saccharomyces cerevisiae*), while also, in the same organism (*S. cerevisiae* [72]), being observed to translocate between the nucleus and cytosol. This translocation is also observed in higher eukaryotes (e.g., Q9GZT8, *Homo sapiens*; Q9EQ80, *Mus musculus*), and, in some cases, appears to be regulated by retinoic acid (Q09GP9, *Bombyx mori* [73]). Although understood as being predominantly cytoplasmic in bacteria, truncated DUF34 homologs are secreted in *Pseudomonas* species as a proposed nematocidal agent [74]. In another case, homologs have been observed to occur at the cellular poles of *E. coli*, co-localizing with PstB (phosphate transporter subunit, ATP-binding) and TktA (transketolase) [32].

Historically, associations of NIF3L1 with human disease have driven much of the impetus for research into this DUF34 homolog [30,31,75,76]. Such links to human disease have been particularly reinforced by many non-focal publications (Table S3; Data Table S2). Indeed, expression of DUF34 in eukaryotes has been associated with several human pathologies, including cancers [77,78,79,80,81,82,83,84,85,86,87,88,89,90,91,92,93], chemotherapeutic drug response [94,95], psychiatric disorders [96,97], cardiovascular disease [98,99,100], insulin resistance [101], osteoporosis [75,102], inflammation [103], Amyotrophic Lateral Sclerosis (ALS) [30,104], William-Beuren Syndrome [31], as well as several other degenerative and developmental neurological diseases [76,105,106]. The regulation of DUF34 homologs by retinoic acid or biochemical relatives (e.g., all-trans retinoic acid, ATRA; testosterone [Comparative Toxicogenomics Database]) appears to be conserved between humans, mice, and select life stages of some insects [73,107,108,109]. Associations to cell differentiation through gene regulation were also numerous [73,106,107,108,110,111,112,113].

Links to virulence and environmental stress responses dominated the studies of bacterial and fungal DUF34 homologs [32,74,114,115,116,117,118,119,120,121,122,123,124,125,126,127]. In addition, links to the regulation of central carbon metabolism were made in *Geobacillus stearothermophilus* [128] and *Bacillus subtilis* [129]. Although ssDNA- and dsDNA-binding properties in vitro were observed for at least one archaeal homolog [130], only ssDNA-binding activity has been reported in bacteria [131], observations of which later came under scrutiny in the context of UV-induced DNA damage responses in *E. coli* [32].

In this comprehensive review of the literature for members of the DUF34 family, observations and functional associations were highly pleiotropic and could be the result of many indirect effects. The only precise molecular function proposed with compelling biochemical evidence is the role as a metal ion insertase in metallocofactor biogenesis described for the homologs of *Methanocaldococcus jannaschii* [132] and *Methanococcus maripaludis* [133]. 

### 3.2. Conservation of Metal Binding Site but Variability of Metal Identity across DUF34 Structures

To complement the literature search, PDB was queried using select DUF34 sequences (YqfO, *B. subtilis*, P54472; NIF3L1, *H. sapiens*, Q9GZT8; YbgI, *E. coli*, P0AFP6; MJ0927, *M. jannaschii*, Q58337) as input. These initial queries returned 15 unique structure entries of DUF34 proteins from six different organisms (5 bacteria, 1 archaeon) (Table 2). Text-based queries of PDB were also performed using “NIF3”, yielding a total of 27 structures, of which only 16 were discernible members of the DUF34 family. These were found to represent two superkingdoms and, within these, seven distinct organisms (eight structures respectively from each, bacteria and archaea).

DUF34 monomers form a homohexameric quaternary structure assembled through the trimerization of homodimers in a “head-to-tail”, tessellating fashion. This homohexameric toroid is conserved across published structures with the central opening averaging a diameter of 31 Å (range: 24–38 Å). In some cases, this toroid is modified by the addition of trimeric “lids” to each side of the central opening, creating a cage-like structure; the monomeric structural features constituting these “lids” are the inserted P_II_-like domains observed in the DUF34 family members belonging to select bacterial clades, fungi, and vertebrates [134]. These inserted domains forming these trimeric “lids” have been described as highly flexible, affecting the resolution of the corresponding architecture [134,138]. 

A dinuclear metal-binding active site predicted to be catalytic, not structural [26] is highly conserved across available structures of DUF34 family proteins (Table 2). This active site structure is defined by a central cleft per monomer within which two divalent metal ions bind [26]. The nature of these divalent metal ions varies: from iron found in both bacterial and archaeal homologs [26,132] to zinc found in bacterial homologs containing the additional P_II_-like domain (i.e., SA1388 of *Staphylococcus aureus*; YqfO of *Bacillus cereus*) [134,138]. This difference in metal ion-binding does not appear to be attributable to the additional domain as the topology of the active site has been described as remaining entirely undisturbed, or “identical”, between homologs with and without the distinct domain architecture [134,138]. 

**Figure 1:**
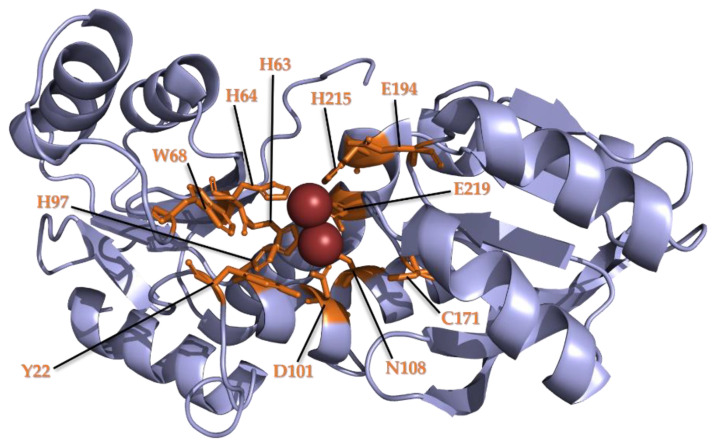
Dinuclear metal-binding site of the E. coli DUF34 homolog, YbgI. The crystal structure of YbgI (DUF34 homolog, *E. coli*) illustrates conserved residues of the protein family specific to the monomeric cleft of the active site and its dinuclear metal center. There are highly conserved residues noted by Ladner et al. [26] to demonstrate involvement in the structure of the binding pocket that are distinctively colorized, annotated (orange; residue identity and location labeled accordingly).

The metal ion-binding sites found in bacterial DUF34 structures contain seven highly conserved residues: five histidines, one glutamate, one aspartate [26,138] (Figure 1). These seven residues are conserved in both YbgI and YqfO forms, the latter possessing the additional, central “YqfO-like” domain [134]. The localization of the active sites within the inside of the toroid’s central channel is ubiquitous, however, solvent-accessibility of this space differs between the two types of quaternary structure, the “cage-like” prolate spheroid with trimeric “lids” demonstrating greater restriction of access to active sites [131,134]. It should be noted that one outlier publication regarding the archaeal DUF34 family member, MJ0927 of *M. jannaschii* (4IWG, 4IWM), appears to differ greatly from all other descriptions of quaternary structure for this family [130,137], even contradicting several structures published for the same homolog (3WSD, 3WSE, 3WSF, 3WSG, 3WSH, 3WSI), of which even go as far as to resolve the active site in different states of oxidation [132]. This anomalous structure is described as a homohexameric spheroid with three openings (~33Å in diameter), instead of the single, central opening of the toroid conserved in all other published structures of the DUF34 family.

### 3.3. Family Wide and Superkingdom-Specific Signature Motifs

The NIF3/DUF34 family is large, containing 6804 member sequences in Pfam (Pfam release 32.0), and its members span all kingdoms of life. Previous studies have already shown that proteins of this family can have different domain architectures [26,130,131,134,138] but no systematic, comparative analysis of the architectural distinctions had ever been performed across all superkingdoms. We, therefore, set out to classify the proteins of the DUF34 family into different subtypes based on the domain arrangements and the presence-absence of specific sequence motifs. Because several DUF34 protein structures were available (Table 2), these were used to guide alignment choices and to ultimately map conserved residues. 

**Figure 2:**
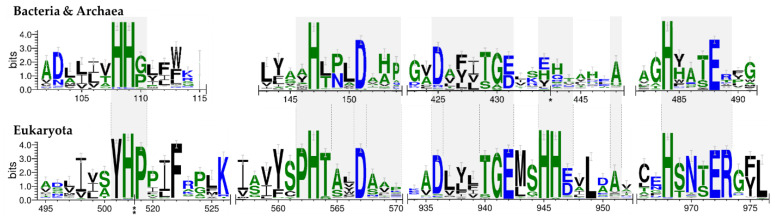
Key motifs of Bacteria and Archaea compared to those of Eukaryota. Sequences were aligned for eukaryotic sequences, separately, and, for bacterial and archaeal sequences, combined. A multiple motif method was used to determine and compare family signatures. A full figure illustrating the distinct levels of conservation per superkingdom can be examined in Figure S3.

To resolve subtypes within the DUF34 family, multiple sequence alignments were initially performed inclusive of members across all superkingdoms. Ortholog sequences were extracted from OrthoInspector for each superkingdom (Data Table S3), and structure-based alignments were generated for each group using the MultAlin and ESPript webservers (Figure S2) [141,142]. The motifs were divided into three groups, or “tiers”, based on their degree of cross-superkingdom conservation. Four motifs were found to be conserved across all three superkingdoms (logos with distinct tiers for all three superkingdoms are shown in Figure S3). These conserved residues of tier 1 were all integral to the metal-binding pocket and are the residues described in Figure 2.

The most notable difference in the more highly conserved motifs was within the dual-histidine motif of the N-terminal region (Figure 2). In eukaryotes, the first histidine residue is replaced by a tyrosine, which may alter the dimensions of the binding pocket (Figure 1). Another notable distinction in eukaryotes is the second histidine pair ((M/L)xHH) located after the C-terminal “Dxxx(T/S)G(E/D)” motif (Figure 2). As no published structures for eukaryotic homologs were available, a model of a representative tertiary structure was generated using the Phyre2 fold prediction webserver (Figure S4). This alignment suggested that the additional histidine pair did not contribute to the binding pocket (Figure S4d), and was, instead, positioned exposed on the protein surface, implying a possible role in protein-protein interactions; however, characterizations of this and similar structures have demonstrated a putative involvement in the architecture of the cleft of the active site formed upon dimerization [138]. A final distinguishing feature observed in the eukaryotic tier 1 sequence is an additional arginine residue following the C-terminal “HxxxE” motif of the C-terminus, a final motif indicated as a likely contributor to the binding pocket [26,134].

### 3.4. A Variable Central Insertion Occurs in Some DUF34 Family Members

**Figure 3:**
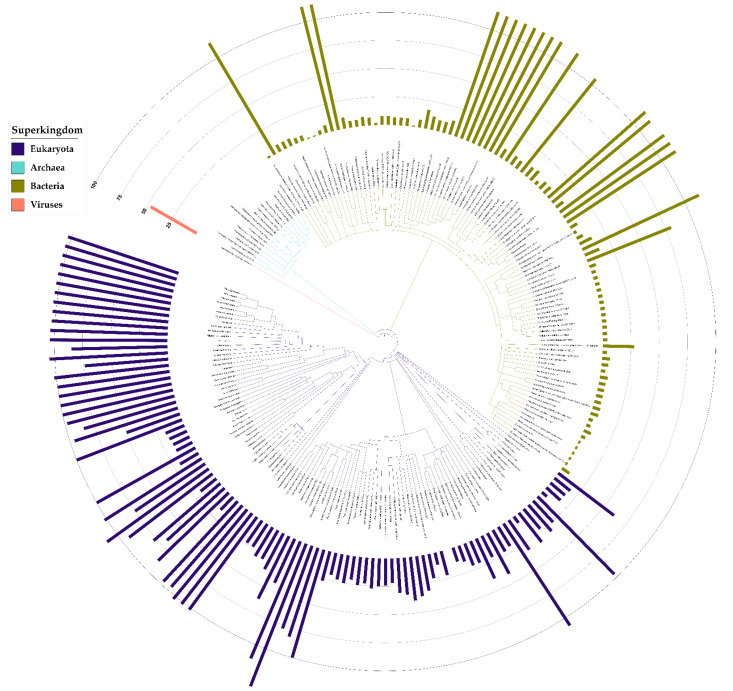
Inserted domain lengths across model taxa. The lengths of inserted domains were measured for each homolog. The sequences (organisms listed in Data Table S4) were aligned per superkingdom for delimiting domains, which then allowed for the measurement of each inserted region (if present). An evolutionary tree was generated using PhyloT and iToL, and was mapped with the lengths of inserted domains within each respective homolog. For all inserted domain lengths measured, these data were used to generate Figure S5, a histogram illustrating counts by ranges of domain lengths per superkingdom.

Alignments performed per superkingdom revealed a large diversity in the lengths of aligned sequences (Data Table S4). The spacing between the Tier 1 motifs seemed to vary greatly with the superkingdom. To better understand the occurrence and distribution of lengths for this inserted domain, the regions between the “YxxHxxxxD” and “Dxxx(T/S)G(E/D)” motifs were manually extracted, lengths measured, and their values were then superimposed onto a species tree (Figure 3). With this, it was revealed that the inserted domains were relatively well conserved in select clades of bacteria, a finding reminiscent of an earlier observation made by Godsey et al. [134]. Unexpectedly, an inserted region was frequent in proteins from higher-order eukaryotes but was absent from archaeal homologs. Among eukaryotic DUF34 proteins, the insertion sizes followed a pattern of diminishing length from vertebrate to invertebrate homologs (from higher-order to lower-order eukaryotes) (Figure 3). In contrast, the length of this domain was relatively stable among bacterial homologs, if occurring at all, with 28.3% harboring a large form of the insertion (~100 aa), while the remaining sequences lacked the domain entirely. Outside of the regions observed in vertebrates, the sizes of this domain varied greatly, especially in members of invertebrate bilateria and fungi, the latter taxon demonstrating domains of the shortest lengths. Only one viral DUF34 member, MIMI_R836 (Q5UQI9) of *Acanthamoeba polyphaga mimivirus*, was retrieved from published data and its length was notably dominated by the inserted domain. 

### 3.5. The DUF34 Family Can Be Split into Eight Interconnected Subgroups

**Figure 4:**
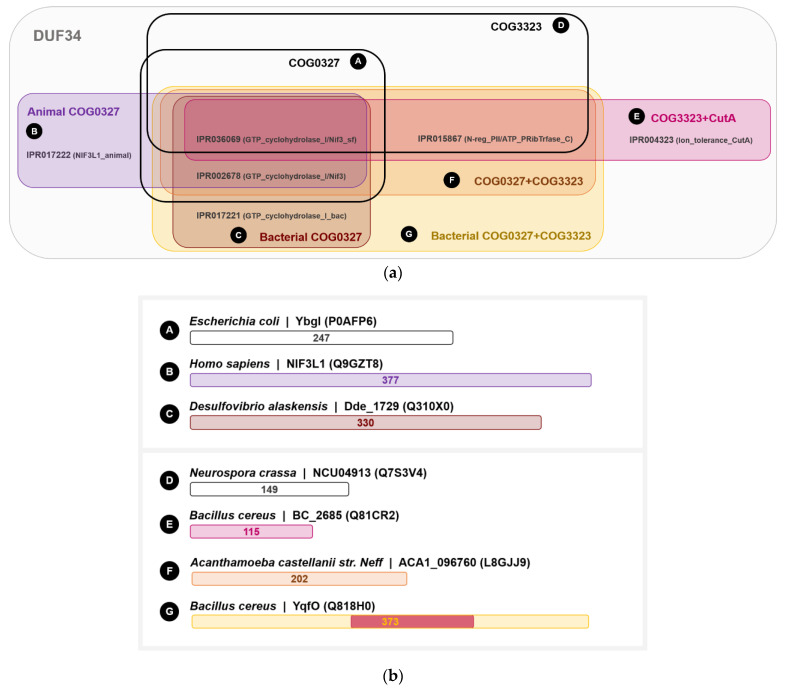
COG-InterPro HMM signature profile relationships and defined subgroups across DUF34 family members. The sequences of organisms across the DUF34 protein family, including all fusions and paralogs, were analyzed for co-occurrence relationships of COGs and HMM-determined InterPro family/superfamily/domain annotations. All organism homologs, paralogs & fusions were validated using eggNOG and KEGG Paralog Search. Sequences missing InterPro annotation were analyzed by NCBI CDD Search and InterProScan Search. See Data Table S5 for categories and respective COG designations/InterPro signature profiles in tabular format. The sequence source organisms considered were those also observed in Data Table S4. Groups were designated by differential keystone signatures shown in (**a**) and select representative sequences of subgroups (A–G) are shown (**b**).

To further characterize domain architectures and examine possibilities of functional subclasses, we collected the annotated domains linked to DUF34 family members, specifically leveraging InterPro HMM profile signature identifiers and EggNOG group IDs (Clusters of Orthologous Groups or COGs) (Figure 4; Data Table S5). Various overlapping combinations of COGs and HMM profile signatures were observed, generating a set of specific architectural patterns that were used to delineate alphabetically named subgroups (i.e., A–G). Most DUF34 members fell within one of two keystone COGs. The first, COG0327 (subgroup A; Figure 4a), is predominantly defined by the presence of two specific HMM profile signatures, IPR036069 and IPR002678, and largely defines the shared bases across subgroups. COG0327 is further divided by HMM profile signatures into two subgroups, subgroup B and subgroup C (Figure 4a), the former containing an animal-specific signature (IPR017222) and the latter harboring a bacteria-specific signature (IPR017221). Although subgroup C was described by InterPro-defined HMM profile signature annotations as being limited to bacteria, nearly all proteins observed within this subgroup belonged to eukaryotes. All members of subgroup B occurred in eukaryotes. The second keystone COG of the DUF34 family, COG3323, as defined by the presence of IPR015867 and IPR036069 (subgroup D; Figure 4a), with IPR036069 being shared between COG3323 and COG0327. The addition of a third HMM profile signature, IPR004323, to the pairing of IPR015867 and IPR036069 defined the fifth subgroup, subgroup E. Homologs containing all three keystone COG-definitive signatures (i.e., IPR002678, IPR015867, and IPR036069) was determinate for fusions of COG0327 and COG3323. These fusions were observed to occur in two forms: subgroup F and subgroup G, the latter of which was defined by the additional bacteria-specific signature, IPR017221 (Figure 4a), a signature previously noted in the definition of subgroup C. 

The D-G subgroups can be differentiated from the A-C subgroups by the presence of an “HPYE” motif attributable to the HMM profile signature, IPR015867 (Figure S6a,b). It can also be noted that subgroups D and E can be viewed as stand-alone forms of the inserted domain found in subgroups F and G. For example, for the DUF34 paralogs of *B. cereus*, BC_2685 (Q81CR2), and BC_4286 (Q818H0), the latter sequence was found to contain an inserted domain bearing high similarity to the former (31.0% identity, 48.0% similarity; EMBOSS Matcher; Figure S7d) (Figure 4b). This same paralog, BC_2685, was identified as a member of the CutA1 protein family (PF03091). Interestingly, this YqfO-like paralog was also found to have a greater identity to the CutA1 homolog of *H. sapiens* (O60888; 29.4% identity, 47.1% similarity) than to that of other bacteria (i.e., *E. coli*; P69488; 25.6% identity, 55.8% similarity). Interestingly, the final glutamate residue of the key motif also distinguishing DUF34 protein family member inserted domains, “HPYE” of the IPR015867 HMM signature profile (Figure S7g), was replaced by a glutamine in the CutA1 of *E. coli*, a replacement also observed in the inserted domain of NIF3L1, the DUF34 homolog of *H. sapiens*. The CutA1 protein family (formerly known as DUF190) has historically been linked to divalent cation tolerance, copper sensitivity, and cytotoxicity (PF03091; IPR004323; COG1324) [143,144,145,146,147,148,149]; however, due to characteristics of the quaternary structure (trimers form ferredoxin-like folds [150]), roles in signal transduction and regulation have also been suggested [151,152,153]. More recently, refute of the protein’s involvement in metal ion tolerance has led to predictions of CutA1 proteins acting in a small molecule carrier or signaling capacity [154,155]. Still, the functions of all three “CutA” proteins remain under-defined with only small attributions put forward for each, in addition to CutA1: CutA2 (DsbD) is thought to have disulfide oxidoreductase activity [156]; and CutA3 (YjdC) has been annotated as an HTH-type transcriptional regulator (TetR/AcrR family), more specifically a negative regulator of nitroreductase NfnB [157]. 

### 3.6. Taxonomic Distribution Suggests That the NIF3 (COG0327) and YqfO-like (COG3323) Domains Have Different Functions

**Figure 5:**
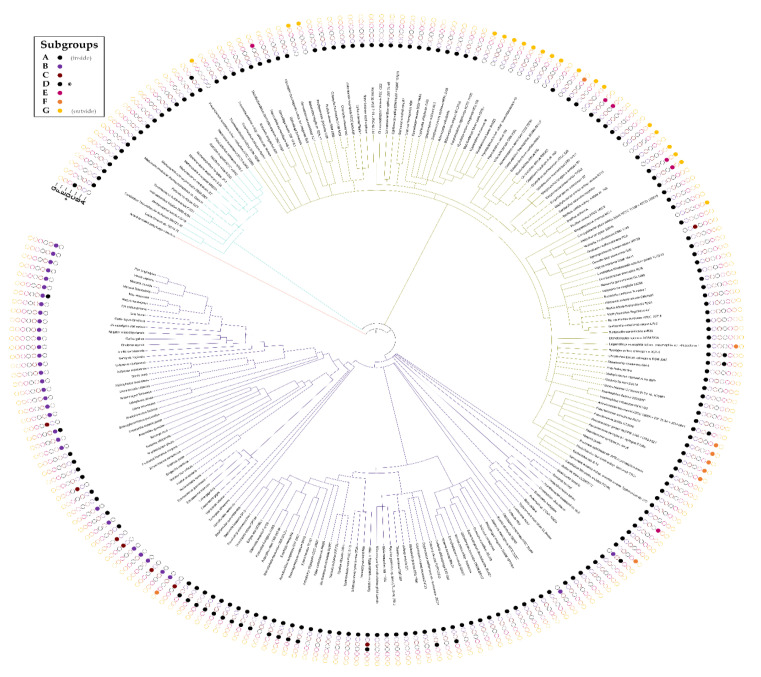
Absence–presence of DUF34 architectural domain subgroups. Absence–presence data of COGs and HMM-determined InterPro family/superfamily/domain signature profiles added to a species tree, generated using organisms harboring published homologs and those used in alignments acquired via OrthoInspector (Data Table S4). Proteins are designated as categories A–G, as detailed in Figure 4 and Data Table S5. These homologous domains are classified in the map according to their HMM-defined DUF34 domain identities (see Figure 4a).

Contrary to expectations for the universal conservation established by past publications, particularly in Eukaryota, DUF34 appeared absent from the eukaryotic clade of *Viridiplantae* with the closest incidence of homologs occurring in select haptophyta. Although some sequence-based queries of NCBI’s databases indicated the existence of a partial homolog belonging to a specific eudicot (i.e., histidinol dehydrogenase chloroplastic isoform X1, GEY60218.1; GFD1148.1; KYP77406.1), these few observations appear largely uncorroborated and were suspected to be products of bacterial contamination. *Caenorhabditis elegans*, a common model organism, was also observed to lack a DUF34 homolog. Among the organisms analyzed, Archaea exclusively harbored DUF34 members of subgroup A (Figure 5). The animal-specific subgroup B was restricted to *Metazoa*, occurring ubiquitously across *Euteleostomi*. Subgroup A often replaced the animal-specific subgroup B in other lower-order clades of *Metazoa* including, but not limited to: *Arthropoda*, *Annelida,* and *Mollusca* (Figure 5). Subgroup A also demonstrated the greatest overall prevalence and broadest taxonomic range, being observed in the majority of organisms across the three major superkingdoms. Almost all bacteria lacking a subgroup A homolog harbored a subgroup G, the bacterial COG0327-COG3323 fusion, in its place. Of all YqfO-like (COG3323) variants of the DUF34 family (subgroups D–G), only subgroup G was ever observed to occur without a subgroup A, B, or C form also present. The only exception to this pattern of subgroup absence-presence was *Acanthamoeba polyphaga mimivirus* (tax ID: 212035), which was found to only encode a subgroup D homolog. Interestingly, the DUF34 form annotated as being specific to bacteria, subgroup C, was exclusively observed among select species of non-metazoan bilateria, only occurring in a single bacterial organism (i.e., *Desulfovibrio alaskensis*). 

Approximately three-quarters of the genomes analyzed encoded only one subgroup of the DUF34 family. In organisms with two or more subgroups, the most frequent combination was the co-occurrence of either a subgroup A, B, or C with any member of subgroups D–G. Although seldom, subgroups A, B, and/or C were observed to co-occur together, most often in pairs, in eukaryotic organisms, but never in bacteria, archaea or viruses. Only members of subgroup G ever occurred alone more than once without any subgroups A–C. This suggests that this is the only form that can functionally replace any one of the A–C forms and that the stand-alone versions of the inserted domains definitive of subgroups D or E, relative to subgroups A–C, certainly perform a different function. 

In a larger survey of available complete bacterial genomes (JGI-IMG/M; accessed on 30 January 2020), DUF34 homologs annotated as belonging to both COGs (subgroups D–G) COG3323 and COG0327, occurred in 18% of complete bacterial genomes, while a much larger fraction of the bacterial family members (66%) were found to encode only the COG0327 designation (Subgroups A–C) (Data Table S6) [158,159,160].

### 3.7. Physical Clustering and Co-Expression Further Link the DUF34 Family to Metal Ion Homeostasis and Iron Sulfur-Cluster Metabolism

To determine associations based on physical clustering, gene neighborhoods for members of the DUF34 family were examined using the IBT–UNAM Computational Genomic Group’s Gene Context Tool (GCT). The GCT webserver was used to retrieve collections of commonly clustered COGs of DUF34-encoding operons for taxonomic subsets of bacterial and archaeal DUF34 family members (Data Table S7, a). These data were then used to develop a method of text analysis-enabled assessment of COG and COG description keyword/phrase frequencies, the methods of which are described further in the Supplemental Methods Section (1.2). This approach will be referred to, henceforth, as Physical Clustering Keyword Frequency Analysis (PCKFA). Using PCKFA, COGs and their descriptions were examined for common annotations and trends that could inform on potential functional associations. PCKFA of COG identifiers was used to generate a ranked list of co-occurring COGs. This data was sorted by frequency to generate a final list of the top 20 highest-ranking COGs occurring across all taxonomic ranges (Table 3). Upon closer review of the associated functional annotation, it was determined that 65% (13) of the top 20 most frequently co-occurring COGs of DUF34-containing operons were either predicted or confirmed to be “metal ion-binding/-dependent”, an incidence notably greater than the one-third of proteins within PDB predicted to require metal ions [161]. Three of the 13 metal ion-binding/-dependent COGs within those ranking within the top 20 were found to bind Fe-S clusters (Table 3). Despite the diversity of operon compositions that were observed within and between the data’s selected taxonomic ranges (Data Table S7), keywords linked to metal ion homeostasis and Fe-S cluster-dependent processes recurred with notable frequency (Figure S7a).

Representative operons were curated to facilitate more granular, context-driven analyses investigating the observed trends (Data Table S7, d–e). With an initial survey of metal bias based only on COG descriptions, whether or how many of the encoded COGs might be linked to pathways involving metal ions and/or Fe-S clusters remained unclear. This was largely due to the generally poor functional annotation statuses for many of the COGs retrieved. Therefore, the individual sequences constituting these operons were investigated thoroughly using functional annotation and key background literature (as described in Methods) to investigate annotations for any catalytic dependencies or interactions with metals ions. In 13 of the 51 selected bacteria (25.5%), COG0327 was observed to occur alone, and, of those not encoded alone (38 of 51), 31 were found to encode at least one protein with supported annotations of metal-binding/-dependence (81.6% of operons; count inclusive of Fe-S cluster-containing proteins) (Data Tables S7 and S8). Similar incidence was observed across archaeal representative operons with 3 of 9 archaeal COG0327 proteins (33.3%) being encoded alone, and, of those not, five were found to encode at least one metal-binding/-dependent protein (5 of 6 operons; ~83%).

Of all COGs encoded by COG0327-containing representative operons, COG1579 co-occurred most frequently. This COG was also determined through PCKFA to be the top-most ranked in, both, singular occurrence and paired occurrence with COG0327 across taxonomic ranges (Figure S8b,c). COG1579 is a family of unknown functions (DUF164) that is conserved primarily among bacterial clades, although homologs are found also in archaea. Members of this group have been linked to functional roles in chemotaxis, flagellin synthesis, type III secretion systems (i.e., *Helicobacter pylori* and *Chlamydia trachomatis* [125,170,171,172]), and bacteria-induced host cell maturation (i.e., *Mycobacterium avium* [173,174]) but the molecular mechanisms involved remain mysterious. The homolog of *Mycobacterium tuberculosis* has been noted as an essential gene under some circumstances [175]. COG1579 members have an obvious link because of the presence of a domain belonging to the zf-RING_7 Pfam family (PF02591 [176]). A characteristic feature of the zf-RING_7 family is the presence of a C4-type zinc-ribbon domain with two pairs of cysteines in a CxxC-x (18–26)-CxxC (zinc-finger) motif capable of binding zinc ions. Published structures (5Y06/5Y05 of *M. smegmatis* [171]; 4ILO of *Chlamydia trachomatis* [172]) demonstrate an unusual coiled-coil structure that is book-ended by the aforementioned distinctive zinc-finger domain. 

**Figure 6:**
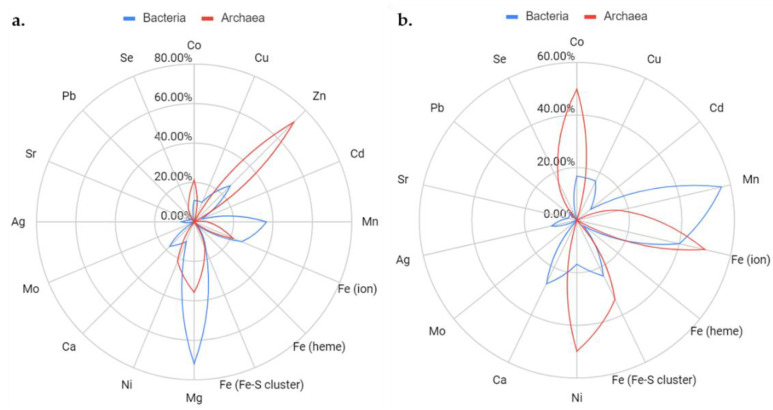
Metal ion-binding of proteins encoded in representative Bacterial and Archaeal operons. (**a**) A radar chart illustrating the proportions of DUF34-operon encoded proteins documented to interact with certain metals or metal-containing moieties. Accounting for the over-representation of magnesium and zinc among available protein structures, a second radar chart (**b**) was generated to show the same data without proteins found to exclusively bind either or both ions. Bacterial data are shown in blue while Archaeal data are shown in red. Data used to generate these figures can be found in Table S4.

Despite the high clustering frequencies discernible for several co-occurring COGs, a single link between DUF34 homologs and a distinct metabolic area remained unclear. The diversity of metals associated with proteins encoded by DUF34-containing operons failed to support a preference for a single metal or metal ion-complex, although zinc and iron were found to be common interactors, second to magnesium and manganese. In addition, many of the families listed in Table S4 were found to interact with several metal ions (up to eight) with averages, across the table, of ~2.5 different metals for bacterial proteins and ~1.9 for archaeal proteins (Figure 6). Several metal-dependent/-binding COGs found to frequently cluster within DUF34-containing operons across taxa (Table 3) were also common among representative operons (Data Table S7). When compared to all available PDB structures (PDB 2020), the relative abundance of metal-binding proteins across both archaeal and bacterial representative operons was observed to be significant (Data Table S8; Figures S9–S11). A strong association with Fe-S cluster associated proteins was observed (7 of the 40 bacterial and 2 of the 14 archaeal metal-binding proteins analyzed) (Figure 6 and Table S4). Examples include HcgA/BioB and HmdC/HcgG (FlpA homolog) in archaea, and MutY, SplB, NfuA, PhrB, and BolA in bacteria. 

Because DUF34 is conserved across bacteria, archaea, and most eukaryotes, and as physical clustering was appropriate for only two of three superkingdoms [177], co-expression (top 300 co-expressed, CoXPresDb; Data Table S9, sheets d.1–d.10) and coregulation databases (ProteomeHD; Data Table S10, a) were consulted to identify trends in putative functional associations of eukaryotic DUF34 family members shared with those observed through preceding analyses with bacterial and archaeal family members. Interestingly, a number of genes directly involved in iron homeostasis and Fe-S cluster biogenesis were observed to occur in most eukaryotic organisms surveyed (Data Table S9; Figure S12). BolA or BolA-like family members occurred in *H sapiens*, *M. mulatta*, and *S. cerevisiae*. However, in absence of a BolA-like homolog, *S. pombe* showed co-expression of a Fe-S cluster biogenesis factor, caf17 (IBA57-like; SPAC21E11.07), a member of the GcvT and CAF17 families [178]. Upon further review of the top 100 genes co-expressed in *H. sapiens*, YAE1D1 (57002, Yet Another Essential domain-containing 1), a highly conserved protein essential to cytosolic Fe-S cluster protein assembly (CIA) complex [179], was also observed. Although a Yae1 homolog was not observed in the acquired datasets for either yeast, another essential component of the CIA complex, the Fe-S cluster-binding ATPase, Nbp35 (2543416, *S. pombe*; 852789, *S. cerevisiae*), was found within the top 130 co-expressed genes of each. Genes encoding this protein were found co-expressed with NIF3L1 homologs in three eukaryotes of the 10 for which data was retrieved. Similar trends associating Fe-S cluster proteins and pathways were observed upon gene functional classification analyses of the same sets of co-expressed genes using the DAVID bioinformatics suite (Data Table S9, e.1–e.10).

### 3.8. DUF34 Fusions Fortify Links to Metals and Metallocofactors, Most Notably Fe-S Clusters

**Figure 7:**
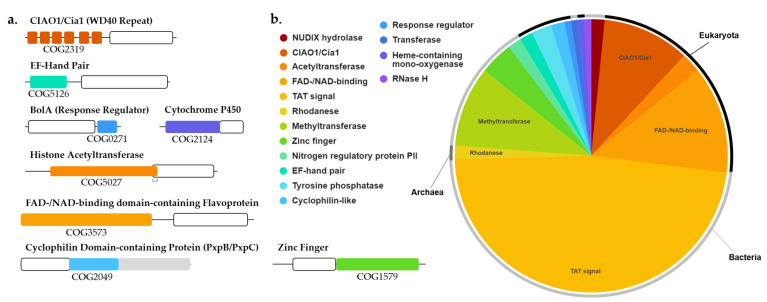

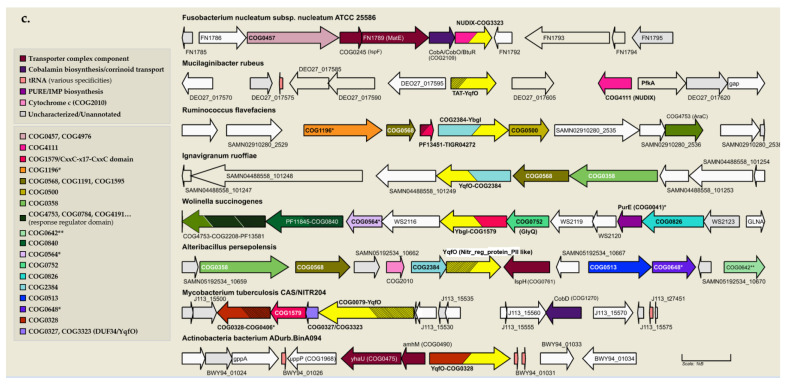
DUF34 fusions and select gene neighborhoods. (**a**) Domain architectures of DUF34 fusions. The domain rendering dimensions and positions are approximate. DUF34 domains are rendered in white with black outlines. Domain colors correspond to the key shown in panel b. COGs of fusion domains are listed below each. Fusions deemed “invalid” or “inconclusive” were excluded for panels a and b. (**b**) Pie chart of DUF34 fusions (126 sequences, total). The outer halo surrounding chart indicates the superkingdoms in which respective fusions were observed (Eukaryota: black; Archaea: dark gray; Bacteria: light gray). (**c**) Neighborhoods of select bacterial and archaeal fusions are shown (12 kb, each), all of at least “conditional” validation confidence (Data Table S11). DUF34 is depicted in bright yellow and fusion domains are indicated by hashing or alternative coloring. For DUF34 sequence labels, “YqfO” denotes a sequence also containing inserted domain, COG3323, while “YbgI” denotes a sequence without the inserted COG3323 domain. Rendered fusion domains do not reflect exact sizes or locations. The color key is divided into two sets of identities (gray boxes): (top) general metabolic theme or specific annotation with bioinformatic precedent; and (bottom) COGs observed in physical clustering analysis (PCA). COGs also observed in PCA (Table 3) are shown in bold. Six minor exceptions to the top-20 rank cut-off are shown in bold with an asterisk (*): COG1196 (top 31st); COG0564 (top 23rd); COG0648 (top 25th); COG0406 (top 48th) in a fusion with COG0328; and COG0041 (top 36th). Others observed in rep. operons but were ranked beyond the “minor exception” threshold (exceeded top-50) in PCA are shown without additional symbols, not bolded: COG0245 (116th) and COG0761 (61st). Finally, one was not observed in PCA (not bolded) but was in at least one rep. operon (double asterisk, **): COG0642 (SAMN05192534_10671 of *A. persepolensis*; rep. operon, *Desulfurispirillum indicum S5*) (Data Table S7). Note: COG4111 (NUDIX hydrolase), present in panel c (neighborhood of *M. rubeus*), was absent from PCA (any rank) and rep. operons, despite the fusion with COG3323 in *F. nucleatum* having been resolved in preceding homolog capture and literature review.

Fusions can provide substantial insight into putative functional relationships between their constituent protein families. To better understand the full diversity of fusions across the DUF34 family, three different methods were used, as described in the methods section, to generate a curated set of 226 sequences of varying validity (Data Table S11, b), covering 47 distinct fusion classes and 65 different fusion subclasses (see Supplemental Methods, 1.3). After further curation focusing on fusions of highest confidence, nine fusion classes were observed in eukaryotes and seven in bacteria. Eukaryotic fusions of note included those with the following domains: WD40 repeat; BolA (BolA-like); FAD-binding flavoprotein; RING- or THAP-type zinc finger; EF-Hand pair; or histone acetyltransferase (Figure 7a). The most common fusion among eukaryotes were those containing the WD40 repeat domain, CIAO1/Cia1 (COG2319), which is thought to play a role in Fe-S cluster biogenesis. Somewhat consistent with this finding, a fusion with BolA was also observed (COG0271, PF01722; *Fusarium oxysporum Fo47*). It was also remarked that the neighboring of BolA family members, a phenomenon shared by at least one bacterial representative operon (Data Table S7, d.1–d.2), was not necessarily uncommon in fungal genomes, as Bol2, for example, is divergently encoded immediately upstream of DUF34 in *S. cerevisiae*. 

Notable bacterial fusions included domains belonging to COG1579, COG2384, and COG0328, all three COGs having occurred independently in the top-20 ranked COGs determined through PCKFA that were also metal-binding, in addition to being observed among bacterial representative operons (COG1579, *Wolinella succinogenes ATCC 29543*; COG2384, *Ruminococcus flavefaciens Sab67*; COG0328, *Clostridia bacterium 1MN72D_59_214* (taxid: 2044939)). Although without recognizable COGs, the most common gene fusion among bacteria were TAT signals, a sequence feature neglected at the protein annotation level. While the neighborhoods of many bacterial fusions appeared very diverse (Figure 7b), 55% (11) of the top-20 co-occurring COGs of the DUF34 family (Table 3) were represented at least once across all observed neighborhoods. Additionally, genes encoding proteins involved in cofactor biosynthesis, corrinoid/siderophore/metal ion transport, metal- and metal ion stress-dependent processes, as well as DNA/RNA metabolism (e.g., de novo purine biosynthesis), were pronounced among these selected neighborhoods. 

### 3.9. A Role of the DUF34 Family Protein in Folate Synthesis Is Precluded by Bioinformatic and Experimental Evidence

GTP cyclohydrolase I activity was reported using an in vitro assay with the *H. pylori* DUF34 family member, HP0959, expressed in *E. coli* [34]. With the roll-out of UniRule, an automated curation and annotation transfer program, by UniProtKB, the annotation of “GTP cyclohydrolase I type 2” was subsequently electronically propagated across thousands of proteins without further substantiation or review outside of this singular publication.

The canonical GTP cyclohydrolase I (GCYHI) enzymes catalyze a complex reaction, the formation of H_2_-neopterin-triphosphate (H_2_NTP) from GTP, required for the first step of tetrahydrofolate (THF) synthesis in most bacteria [180,181,182]. H_2_NTP is also a precursor to the cofactor BH_4_ and 7-cyano-deazaguanine (preQ_0_) and intermediate in the synthesis of modified RNA and DNA bases [183,184]. Two non-orthologous protein families have been shown to harbor GCYHI activity [185]. The first, COG0302 (PF01227), was first characterized as FolE in *E. coli* K12 and is called GTP cyclohydrolase I type 1 [35]. The second named FolE2 and part of the COG1469 (PF02649) family was discovered much more recently and is called GTP cyclohydrolase I type 2 [186]. The distribution of the two families in Bacteria and Archaea vary greatly, some have FolE1, some FolE2 and some have both [4,187]. Humans encode FolE as the first step of BH_4_ synthesis but no other folate enzyme [183]. A minority of bacteria are auxotrophic for THF, requiring the uptake of a folate source; hence, they do not encode any *de novo* folate biosynthesis enzymes [188]. However, as folate transporters are not present in most bacteria that are folate prototrophs, it follows that the *de novo* THF synthesis genes are often found to be essential in these organisms [35,36]. Folate prototrophy is common in most plants (*Viridiplantae*). although minor differences are observed among specific pathway contributors between select clades [189].

Despite the proposed role of the *H. pylori* DUF34 protein (HP0959) in folate synthesis [34], this hypothesis is not supported by the patterns of occurrence of DUF34 family members across folate auxotrophs or prototrophs. Indeed, organisms prototrophic for folate do not encode DUF34 proteins (e.g., plants), whereas folate auxotrophs, such as *M. genitalium*, do. In general, genes encoding DUF34 proteins are not essential with a few exceptions (Table S5). The gene encoding for GTP cyclohydrolase I, *folE*, is essential in *E. coli*, as is expected in most folate prototrophic bacteria [37]. The same essentiality, however, is not observed in mutants of *ybgI* in *E. coli* (Table S5). Moreover, this would imply that YbgI lacks the GTP cyclohydrolase I activity necessary to effectively compensate for the absence of *folE*, an alternative explanation to this compensatory failure being that the gene had not been sufficiently expressed in previously tested conditions to do so. An additional observation of note, however, is that even the YbgI-encoding operon, as a whole, has been reported as being non-essential in *E. coli* [190]. Although DUF34/NIF3 homologs are considered non-essential in an overwhelming majority of bacteria for which data is available (Table S5), one published case of bacterial DUF34 homolog mutant inviability was found, but it occurred in the context of using a specialized method of mutagenesis in *H. pylori* (i.e., in vitro mutagenesis using the Tn7 transposon) [191]. Moreover, this case stands out compared to other systems again in that the homolog is essential for *H. pylori*, a rare observation among DUF34 family members (Table S5).

**Figure 8:**
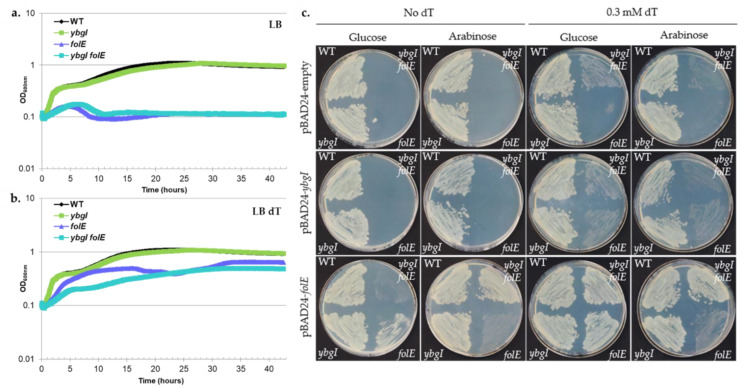
DUF34 of *E. coli*, *ybgI*, fails complementation in the absence of *folE*. Plates were imaged after 20 h of growth at 37 °C. (**a**,**b**) dT essentiality assay. WT, single mutants, and double mutant (*folE*, *ybgI*) strains have been grown at 37 °C in LB supplemented in the absence (**a**) or presence (**b**) or dT 0.3 mM. Each curve shown is averaged across 5 replicates. (**c**) dT essentiality complementation assay. WT, single mutants, and double mutant (*folE*, *ybgI*) strains, containing various derivatives of pBAD24 encoding for either *E. coli* YbgI or FolE, have been streaked on LB plates supplemented with Ampicillin 100 µg/mL in the presence of either 0.2% glucose for repression of the gene expression, or 0.2% arabinose for overexpression of the gene of interest, and in presence or absence of dT 0.3 mM.

With differences in essentiality considered, a series of complementation assays were performed to better illustrate the relationship of *ybgI* to *folE* and the folate biosynthetic pathway. The essentiality of folate in *E. coli* is partially linked to the *de novo* synthesis of thymidine, as the thymidylate synthase (ThyA, [192]), that catalyzes the formation of dTMP from dUTP, uses THF as a cofactor. It was previously reported that complementing the growth media with dT allowed a *folE* mutant of *E. coli* to grow at a low rate [184]. The *ybgI* mutant of *E. coli* had a similar growth compared to a WT in the presence and absence of dT, while the *folE* mutant could only grow in presence of dT (Figure 8). Interestingly, the double mutant also required dT to grow but grew at a slower rate than the *folE* single mutant, eventually reaching the same final OD as the *folE* single mutant (Figure 8a,b). Expression of *E. coli folE in trans* complemented the essentiality of dT upon plating for, both, the single and double mutants (Figure 8c), whereas the expression of *E. coli ybgI in trans* did not complement this phenotype. It can be noted that the overexpression of *folE* in the single mutant did not fully complement the growth phenotype, while successfully doing so in the double mutant (Figure 8c, + arabinose). The WT was not impacted by the overexpression of *folE*, eliminating the hypothesis for toxicity of high FolE levels but revealed a genetic interaction between *ybgI* and *folE* that is also observed with the better growth of the double mutant on dT compared to the single *folE* mutant. Further studies will have to be performed to dissect this interaction but it can be noted that FolE is a metal-dependent zinc-requiring enzyme [193].

## 4. Conclusions

In this comprehensive comparative genomic analysis of the DUF34 family, we presented a collection of arguments refuting a role in folate synthesis as a GTP cyclohydrolase I type 2 in most organisms, including the gram-negative model, *E. coli*. While we concede that it is possible the in vitro GTP cyclohydrolase I activity described for the DUF34 member of *H. pylori*, *HP0959*, may still accurately reflect the enzyme’s ability, further controls―such as site-directed mutagenesis of essential residues or in vivo complementation data―would be necessary to ensure that the observed activity was not related to a contaminating endogenous enzyme or non-biological assay conditions such as low pH. In light of our analyses, the propagation of this annotation should therefore be limited until further experimental work is conducted.

The published quorum emphasizes a pleiotropic role of the DUF34 that is typical of a core molecular function. We propose that members of this family have a general metal ion insertase function that may vary in the substrate and target individual members and clades. Diiron proteins have long been implicated in metal shuttling [194], but the only member of the DUF34 family with notable biochemical and structural characterization is the archaeal HcgD, which has been proposed to act as an iron chaperone in the maturation of the iron-guanylylpyridinol (FeGP) cofactor required by [Fe]-hydrogenase [132]. The structural data presented here strongly link the DUF34 family to metal homeostasis, while the physical clustering, fusion, and co-expression data also suggest a metal link, most notably to Fe-S clusters. Proving metal insertion activity in vivo can be a very difficult task. For example, our group predicted that members of the COG0523 family were involved in metal insertion over 15 years ago and the experimental validation of this prediction has only been published within recent years [195,196,197]. We believe that the thorough analysis presented here should guide future experimental efforts to solve this long-standing functional enigma for one of the most conserved unknowns remaining to be confidently characterized.

## Figures and Tables

**Table 1:** Focal publications featuring members of the DUF34 protein family.

Name	Organisms	Phenotype, Biological Relevance	Reference
YqfO/BC_4286	*Bacillus cereus*	Inserted domain similar to PII-like/CutA1 family proteins; present in select bacterial clades; domain may regulate catalytic activity	[134]
YqfO/BSU_25170	*Bacillus subtilis subsp. subtilis str. 168*	With YlxR, coregulates *tsaEBD* (t^6^A synthesis [62]); disruption impairs *tsaEDB* regulation, loss of glucose-induction of *sigX* via PDHc expression dysregulation	[129]
BmNIF3l	*Bombyx mori*	Translocates to nucleus from cytoplasm upon ATRA tx; higher transcript levels in differentiating tissues; no expression detected in the egg stage	[73]
YbgI/b0710	*Escherichia coli*	Structure, homohexameric toroid; monomers possess dinuclear metal ion-binding site; putatively involved in DNA repair	[26]
		No survival impairment upon mutant UV tx; polar localization during cell division (co-localized with PstB, TktA); GlmS putative interaction partner; mutant sensitive to antibiotics affecting cell wall synthesis	[32]
XynX	*Geobacillus stearothermophilus*	Negatively regulates expression of *xynA* (encodes a secreted xylanase); may be negatively regulated by *xylR*	[128]
NIF3L1/ALS2CR1/CALS-7/MDS015/My018	*Homo sapiens*	Ubiquitously expressed during embryonic development; strong over-expression in spermatogonia-derived, teratocarcinoma cell lines; Isolated, characterized; cytosolic subcellular localization; highly conserved N-, C-terminal regions; shares inserted region of its murine homolog (CutA1-like)	[24]
		NIF3L1 interacts with splice variant, NIF3L1 BP1 (THOC7), cytosolic colocalization; C-terminal leucine zipper-like domain of variant mediates interaction; not indicated in repression in NIH3T3 cells; binding partner, NIF3L1 BP1, demonstrates additional passive presence in the nucleus	[25]
		Retinoic acid-induced binding, cooperative translocation with Trip15/CSN2 from the cytosol to the nucleus (early neuronal development, silences differentiation suppressor Oct-3/4); ubiquitous expression, important in neuronal development	[107]
		Detected in brain, spinal cord, and lymphocytes; observed as two distinct transcripts with similar patterns of expression; highest levels of both transcripts in heart, skeletal muscle, testis; smaller transcript was expressed at a higher level than the other; no deletions, polymorphisms linked to ALS patients relative to controls; 1 of 6 candidates eliminated for a causative link to ALS2	[30]
		1 of 4 hypermethylated, significant differential expression shared between two cancellous bone specimen groups: osteoarthritis, osteoporosis	[75]
		With 14-3-3, co-regulates transcriptional of Wbscr14 by preventing its nuclear localization via complex formation (Wbscr14 participates in the complex-mediated transcription of lipogenic enzymes, promoting fat accumulation)	[31]
		Included in a 7.5-Mb interstitial deletion on 2q32.3–33.1 (28 genes) inpatient diagnosed with SATB2-Associated 2q32-q33 microdeletion syndrome	[76]
		Significantly associated with triptolide chemosensitivity in lymphoblast cell lines	[135]
		COPS2 point mutations consistent with previously defined NIF3L1-COPS2 co-repression interaction model (limited; pathogenesis associated COPS2 mutations: S120C, N144S, Y159H, R173C)	[136]
HP0959	*Helicobacter pylori*	GTP-binding, hydrolysis in vitro, biologically irrelevant pH, temperature	[34]
HcgD/MJ0927	*Methanocaldococcus jannaschii*	Proposed iron chaperone required for FeGP cofactor biosynthesis Homohexameric via 2 interfaced homotrimeric units; binds to ssDNA/dsDNA	[132] [130,137]
Nif3l1/1110030G24Rik	*Mus musculus*	Isolated, characterized; ubiquitous expression across tissues; cytosolic localization; highly conserved N-, C-terminal regions; shares inserted region of the human homolog	[24]
		Retinoic acid-induced binding, cooperative translocation with Trip15/CSN2 from the cytosol to the nucleus (early neuronal development, results in the silence of the differentiation suppressor Oct-3/4); ubiquitous tissue expression, important in neuronal development	[107]
WP_046236688 WP_032702676 PP_1038 VT47_06255 WP_017124074 WP_054077596	*Pseudomonas sp.*	(“YqfO03”) small, secreted protein; demonstrated high potency as nematicide against *C. elegans*, *M. incognita*; free-standing YqfO domain-containing protein (no NIF3/DUF34 domains) is a member of the NIF3 protein family	[74]
Nif3/YGL221C	*Saccharomyces cerevisiae*	Determined to have dual/multiple localizations (cytosolic, mitochondrial)	[72]
SA1388	*Staphylococcus aureus*	The central domain of NIF3 homolog has high structural similarity to CutA1 (family linked to cation tolerance, homeostasis)	[138]
SP1609	*Streptococcus pneumoniae*	Described as a member of the same orthologous group (COG2384) as TrmK, RpoD protein families via structural alignment (incorrect*)	[139]
TTHA1606	*Thermus thermophilus HB8*	Binds to ssDNA (very weakly, in vitro)	[131]
NIF3-like protein superfamily	NA	(electronic translation) describes family members of model organisms (Eukaryota, Bacteria), structures published prior to 2007	[140]

**Table 2:** Published structures of DUF34 protein family members.

Name	Organisms	Ligands	P_II_ Domain	PDB	Phenotype	Reference
YbgI	*Escherichia coli*	(2)Fe^3+^	No	1NMO	NA	[26]
		(2)Mg^2+^	No	1NMP		
HcgD/MJ0927	*Methanocaldococcus jannaschii*	(1)Cl^−^, (2)Fe^3+^	No	3WSD	Weaker Fe1 site under oxidized conditions in vitro	[132]
		(2)Fe^2+^, (1)PO_4_^3−^	No	3WSE		
		(1)Fe^3+^, (1)citrate	No	3WSF		
		(1)Fe^2+^, (1)citrate	No	3WSG		
		(1)Fe^3+^, (1)SO_4_^2−^	No	3WSH		
		(1)Fe^2+^, (1)PO_4_^3−^	No	3WSI		
		NA	No	4IWG	Binds to ssDNA, dsDNA in vitro	[130,137]
		NA	No	4IWM		
SA1388	*Staphylococcus aureus*	(2)Zn^2+^, (1)B3P	Yes	3LNL	Cavity diameter = 38 Å; opening edge length = 20 Å (triangular opening)	[138]
		(2)Zn^2+^	Yes	2NYD		
SP1609	*Streptococcus pneumoniae*	NA	No	2FYW	NA	PDB only
TTHA1606	*Thermus thermophilus*	NA	No	2YYB	Binds ssDNA not dsDNA in vitro	[131]
Sthe_0840	*Sphaerobacter thermophilus*	(7)Cl^−^ *, (14)FMT *, (1)ACT *	No	3RXY	NA	PDB only
YqfO	*Bacillus cereus*	(2)Zn^2+^, (1)HEPES, (1)TRS	Yes	2GX8	NA	[134]

* Asterisk indicates that ion count is per the respective asymmetrical unit as opposed to per monomer.

**Table 3:** Top 20 COGs found to occur in operons containing COG0327.

Rank	COG	Name/Description	Metal(s)	References (PMID, EC Number)
1	COG0327	Putative GTP cyclohydrolase 1 type 2, NIF3 family	Fe^2+^/Fe^3+^, Zn^2+^, Mg^2+^	[26], [132], [138], [26.88.147.156], [26.89.148.157]
2	COG1579	Predicted nucleic acid-binding protein DR0291, contains C4-type Zn-ribbon domain	Zn^2+^	[125]
3	COG0568	DNA-directed RNA polymerase, sigma subunit (sigma70/sigma32)	Zn^2+^, Mg^2+^	[162], [2.7.7.6]
4	COG0358	DNA primase (bacterial type)	Zn^2+^, Mg^2+^, Mn^2+^	[163], [2.7.7.101]
5	COG0457 ^a^	Tetratricopeptide (TPR) repeat	*NA*	None listed
6	COG2384	tRNA A22 N1-methylase	*NA*	[2.1.1.217]
7	COG0079	Histidinol-phosphate/aromatic aminotransferase or cobyric acid decarboxylase	*NA*; Co (cobalamin)	[164], [2.6.1.9]
8	COG0240	Glycerol-3-phosphate dehydrogenase	*NA*	[1.1.1.94]
9	COG0328	Ribonuclease HI (RnhA)	Mg^2+^, Mn^2+^, Co^2+^, Ni^2+^	[165], [3.1.26.4]
10	COG0500 ^b^	SAM-dependent methyltransferase	*NA*	[2.1.1.242]
11	COG0513 ^c^	Superfamily II DNA and RNA helicase (SrmB/RhlB)	Mg^2+^, Mn^2+^	[3.6.4.13]
12	COG0596	2-succinyl-6-hydroxy-2,4-cyclohexadiene-1-carboxylate synthase MenH and related esterases, alpha/beta hydrolase fold (MhpC)	*NA*	[3.7.1.14]
13	COG0655	Multimeric flavodoxin WrbA, includes NAD(P)H:quinone oxidoreductase	Most req. Fe-S cluster; subtypes without Fe-S clusters	[1.6.5.2], [1.6.5.6]
14	COG0752	Glycyl-tRNA synthetase, alpha subunit	Mg^2+^, Mn^2+^, Co^2+^	[166], [6.1.1.14]
15	COG0826	23S rRNA C2501 and tRNA U34 5’-hydroxylation protein RlhA/YrrN/YrrO, U32 peptidase family; ubiquinone biosynthesis protein, UbiU/YhbU	Fe-S cluster/Fe, Ca^2+^	[167,168]
16	COG1028	NAD(P)-dependent dehydrogenase, short-chain alcohol dehydrogenase family	Co^2+^, Fe/Fe^2+^, Mg^2+^, Mn^2+^, Zn/Zn^2+^	[1.1.1.2]
17	COG1897	Homoserine O-succinyltransferase	*NA*	[2.3.1.31], [2.3.1.46]
18	COG0177 ^d^	Endonuclease III (Nth)	Fe-S cluster, Ca^2+^, Co^2+^, Fe/Fe^2+^, Mg^2+^, Mn^2+^, Ni^2+^, Zn^2+^	[169], [4.2.99.18]
19	COG0477 ^d^	MFS family permease (includes anhydromuropeptide permease AmpG, ProP)	*NA*	None listed
20	COG0494 ^e^	8-oxo-dGTP pyrophosphatase MutT and related house-cleaning NTP pyrophosphohydrolases, NUDIX family	Co^2+^, Mg^2+^, Mn^2+^, Zn^2+^	[3.6.1.13]

Exceptions to representative operons relative to table contents: ^a^ Proteins containing TPR repeat domains present in archaeal operons. ^b^ SAM-dependent methyltransferase domains present (not designated COG0500). ^c^ Though not assigned COG0513, helicase domain-containing proteins are present (e.g., Era/COG1159, YhaM/COG3481). ^d^ MutY is present (COG1194), another endonuclease family member. ^e^ MutM/NUDIX domain containing proteins are present (COG0266).

## Supplementary Materials

The following are available online at https://www.mdpi.com/article/10.3390/biom11091282/s1. Figure S1: Word clouds generated from titles of focal and non-focal publications listed in Data Table S2; Figure S2: Secondary structural annotation by superkingdom using MultAlign-based ESPRIPT analyses; Figure S3: Complete DUF34/NIF3 homolog sequence logos across and for each superkingdom (Eukaryota, Archaea, Bacteria) with three tiers of relative conservation; Figure S4: Phyre2 generated model of NIF3L1 (*H. sapiens*) structurally aligned with YqfO to illustrate binding pockets, residues differences within and adjacent to the active site; Figure S5: count per domain length range as a function of superkingdom (histogram); Figure S6: Motif differences in sequences of the D-G subgroups with and without the IPR015867 HMM profile signature annotation; Figure S7: Pairwise alignments of *B. cereus* DUF34 paralogs; Figure S8: PCKFA of COGs and COG descriptions; Figure S9: Abundances of metal ion ligand annotations across published protein structures; Figure S10: Relative abundances of metal-binding proteins per distinct ion across representative operons comparing those of bacteria and archaea to those observed in PDB; Figure S11: Relative abundances of metal-binding proteins per distinct ion as fractions of all encoded proteins across representative operons; Figure S12: Distributions of GO terms retrieved for each set of top 300 co-expressed genes of eukaryotic DUF34 family members; Figure S13: STRING network of GSEA output of DUF34 co-regulated genes of *H. sapiens*; Table S1: All resources used in systematic literature review and subsequent analyses; Table S2: Lists of strains and oligos used in growth assays; Table S3: Formatted table of all organisms, genes/proteins with published data (both focal and non-focal publications); Table S4: Metal ion interactions of proteins encoded by representative operons; Table S5: Essentiality data of DUF34 homologs; Data Table S1: Table of search terms used and generated in the literature review/data capture process; Data Table S2: Catalog of all focal and non-focal publications collected through comprehensive literature review and data capture process of the DUF34 protein family; Data Table S3: Model organism sequences used in initial sequence alignments across and for each superkingdom exported from OrthoInspector (FASTA format); Data Table S4: Collating lists of sequences from model organisms (exported from OrthoInspector) and those acquired from comprehensive data capture and literature review (Table S3); Data Table S5: All COGs and InterPro signature profiles of the DUF34 family including paralogs and some fusions; Data Table S6: “IMG-occurrence” data sheet; Data Table S7: Physical clustering keyword frequency analysis (PCKFA) and representative operons; Data Table S8: Representative operon metal-binding protein abundance; Data Table S9: CoXPresDb (Eukaryota) exports of the top 300 co-expressed genes of DUF34; Data Table S10: Co-regulated genes of *Homo sapiens* DUF34 homolog; Data Table S11: Concatenated list of sequences indicated to be possible non-canonical fusions of the DUF34 family; Data Table S12: STRING network export generated following the results of Data Table S10.
