# Supplementary material for: Comparative Genomic Analysis of the DUF34 Protein Family Suggests Role as a Metal Ion Chaperone or Insertase
Source: Biomolecules. 2021 Aug 27;11(9):1282. doi: 10.3390/biom11091282 (PMC8469502; doi:10.3390/biom11091282)
Supplement: Supplementary file 1 [file biomolecules-11-01282-s001.zip › Supplemental Materials.pdf]

Supplemental Materials

Tables of Contents

1. Supplemental Methods

Item	Contents
**1.1**	Capture of literature, structural, and essentiality data.
**1.2**	Physical clustering analysis.
**1.3**	Fusion analysis.

2-4. Supplemental Tables, Supplemental Figures, and Data Tables

Item	Contents
**Table S1**	All resources used in systematic literature review and subsequent analyses
**Table S2**	Lists of strains and oligos used in growth assays
**Table S3**	Formatted table of all organisms, genes/proteins with published data (both focal and non-focal publications)
**Table S4**	Metal ion interactions of proteins encoded by representative operons
**Table S5**	Essentiality data of DUF34 homologs
**Data Table S1**	Table of search terms used and generated in the literature review/data capture process
**Data Table S2**	Catalog of all focal and non-focal publications collected through comprehensive literature review and data capture process of the DUF34 protein family:<br>**(a)** publications curated prior to September 2020.<br>**(b)** additional unique publications curated on January 18, 2021.<br>*Important*: the organisms and/or sequences added with the addendum to this data table were not included in the analyses completed before this date (January 18, 2021); this applies to all figures, supplemental or in-text, as well as all data tables, supplemental and in-text tables.*
**Data Table S3**	Model organism sequences used in initial sequence alignments across and for each superkingdom exported from OrthoInspector (FASTA format):<br>**(a)** sequences exported with query of NIF3L_HUMAN (Q9GZT8);<br>**(b)** sequences exported with query of GCH1L_METJA (Q58337);<br>**(c)** sequences exported with query of GCH1L_ECOLI (P0AFP6)
**Data Table S4**	Collating lists of sequences from model organisms (exported from OrthoInspector) and those acquired from comprehensive data capture and literature review (Table S3):<br>**(a.1-a.3 [archaea, bacteria and eukaryote, respectively])** Sequences used in Mafft-based alignments selected from merged list of sequences from OrthoInspector (Data Table 3) and those of the organisms listed in Table S3 (FASTA format);<br>**(b)** Mafft (E-INS-i) alignment files per superkingdom (b.1-b.3) with duplicates, exceptional fusions and free-standing YqfO-like protein (~80-100 aa) sequences removed (FASTA format;<br>**(c.1-c.3 [archaea, bacteria and eukaryote, respectively])** extracted sequences per domain region (FASTA format);<br>**(d)** central inserted domain lengths per organism with superkingdoms noted (determined from extracted domain regions, Data Table 4, c.1-c.3), used to generate Figure 3a;<br>**(e)** binned lengths of inserted domain lengths across organisms (with superkingdoms) used to generate Figures 3b-3c
**Data Table S5**	All COGs and InterPro signature profiles of the DUF34 family, including paralogs and some fusions:

	**(a.1-a.4)** raw COG exports;<br>**(b.1-b.4)** duplicates curated and organized per tax ID to represent inferred paralogs;<br>**(c)** final dataset of sequences per organism (list of organisms defined in Data Table 4) classified by InterPro signature profiles (subgroups A-G), including paralogs;<br>**(d)** counts per subgroup relative to each organism (counts of data presented in Data Table 3, c);<br>**(e.1)** count data (Data Table 5, d) formatted with tax IDs for facilitating tree mapping (Figure 6) with categorical key for mapping included (e.2);<br>**(f)** brief analysis of subgroup distribution of sequences;<br>**(g.1)** paralog occurrence for organisms featured in Figure 6, and **(g.2)** brief analysis of those data
**Data Table S6**	"IMG-occurrence" data sheet: **(a)** Phylogenetic Occurrence Profiles for bacterial genomes, raw export from IMG database with counts; **(b)** COG3323 only; **(c)** COG0327 only
**Data Table S7**	Physical clustering keyword frequency analysis (PCKFA) and representative operons:<br>**(a.1-a.2)** Input data for PCKFA, COG (a.1) and COG description (a.2) lists as determined by GCT NG webserver per taxonomic range;<br>**(b.1-b.2)** raw output data of text analysis performed via textalyser.net (seoscout.com/tools/keyword-analyzer/) for taxonomic range-specific COG (b.1) and COG description (b.2) lists;<br>**(b.3)** COG description PCKF output with irrelevant/uninformative keywords and phrases removed;<br>**(b.4)** irrelevant/uninformative keywords and phrases removed from COG description PCKF output;<br>**(c.1-c.2)** formatted output of PCKF analysis for COG (c.1) and COG description (c.2) lists;<br>**(c.3)** formatted output of PCKFA for COG description list unfiltered for irrelevant/uninformative keywords and phrases;<br>**(d.1)** COGs only, bacterial representative operons<br>**(d.2)** bacterial representative operons with cell coloring representative of metal status;<br>**(e.1)** COGs only, archaeal representative operons;<br>**(e.2)** archaeal representative operons with cell coloring representative of metal status
**Data Table S8**	Representative operon metal-binding protein abundance:<br>**(a)** PDB annotation data across metal ligands, PDB 2020 (13 metal ions, 2 forms of bound Fe), used to generate Figure S9;<br>**(b)** metal-binding protein abundance across representative operons, determined for each ion relative to all representative operon-encoded proteins (per superkingdom) (Figure S10, Figure S11);<br>**(c)** individual protein counts per distinct ion listed as a function of individual representative operons (cells formatted to simulate a heat map, 0 [min] to 5 [max])
**Data Table S9**	CoXPresDb (eukaryota) exports of the top 300 co-expressed genes of DUF34.<br>**(a.1-a.10)** eukaryotic coexpression data retrieved from CoXPresDb for the top 300 co-expressed genes with their respective DUF34 homologs; UniProt and Gene Entrez IDs are provided:<br>– NIF3L1 (Q9GZT8, 60491) of *Homo sapiens*<br>– Nif3l1 (Q9EQ80, 65102) of *Mus musculus*<br>– Nif3l1 (Q4V7D6, 301431) of *Rattus norvegicus*<br>– NIF3L1 (F1NDR9, 424076) of *Gallus gallus*<br>– nif3l1 (Q4KMJ5, 574421) of *Danio rerio*<br>– anon-35F/36A / CG4278 (Q9NK57, 34970) of *Drosophila melanogaster*<br>– NIF3L1 (F7B590, 700572) of *Macaca mulatta*<br>– NIF3L1 (E2RQI7, 607446) of *Canis lupus familiaris*<br>– NIF3 / YGL221C (P53081, 852651) of *Saccharomyces cerevisiae*<br>– NIF3 / SPCC126.12 (O94404, 2539063) of *Schizosaccharomyces pombe*<br>**(b.1-b.10)** functional cluster analysis (via DAVID suite) of CoXPresDb co-expression data:<br>– NIF3L1 (Q9GZT8, 60491) of *Homo sapiens*<br>– Nif3l1 (Q9EQ80, 65102) of *Mus musculus*

	– Nif3l1 (Q4V7D6, 301431) of *Rattus norvegicus*<br>– NIF3L1 (F1NDR9, 424076) of *Gallus gallus*<br>– nif3l1 (Q4KMJ5, 574421) of *Danio rerio*<br>– anon-35F/36A / CG4278 (Q9NK57, 34970) of *Drosophila melanogaster*<br>– NIF3L1 (F7B590, 700572) of *Macaca mulatta*<br>– NIF3L1 (E2RQI7, 607446) of *Canis lupus familiaris*<br>– NIF3 / YGL221C (P53081, 852651) of *Saccharomyces cerevisiae*<br>– NIF3 / SPCC126.12 (O94404, 2539063) of *Schizosaccharomyces pombe*<br>**(c.1-c.10)** GSEA (via g:Profiler, g:GOSt; options: ordered query, all known genes for statistical domain scope, remaining options remained set to default; any multiple-returned genes were filtered by selection of the ENSEMBL ID with the greatest number of GO annotations; ENSG IDs used for *S. pombe* and *S. cerevisiae* queries) of CoXPresDb co-expression data:<br>– NIF3L1 (Q9GZT8, 60491) of *Homo sapiens*<br>– Nif3l1 (Q9EQ80, 65102) of *Mus musculus*<br>– Nif3l1 (Q4V7D6, 301431) of *Rattus norvegicus*<br>– NIF3L1 (F1NDR9, 424076) of *Gallus gallus*<br>– nif3l1 (Q4KMJ5, 574421) of *Danio rerio*<br>– anon-35F/36A / CG4278 (Q9NK57, 34970) of *Drosophila melanogaster*<br>– NIF3L1 (F7B590, 700572) of *Macaca mulatta*<br>– NIF3L1 (E2RQI7, 607446) of *Canis lupus familiaris*<br>– NIF3 / YGL221C (P53081, 852651) of *Saccharomyces cerevisiae*<br>– NIF3 / SPCC126.12 (O94404, 2539063) of *Schizosaccharomyces pombe*<br>Data Tables c.1-c.10 each produced a supplementary figure (Figures S11) illustrating distributions of GO terms retrieved for each query.
**Data Table S10**	Co-regulated genes of *Homo sapiens* DUF34 homolog; all top genes reported at or above score cut-off of 0.98; co-regulatory proteins exported directly to STRING-db (Permalink: https://version-11-0b.string-db.org/cgi/network?networkId=bXPk8HQtAarr; Figure S12; Data Table 12):<br>**(a)** exported data from ProteomeHD for *Homo sapiens* DUF34 homolog (Q9GZT8-2), 114 genes (excluding NIF3L1)<br>**(b)** functional cluster analysis (via DAVID suite) of ProteomeHD data for *Homo sapiens* DUF34 homolog (Q9GZT8-2)<br>**(c.1)** GSEA (via g:Profiler, g:GOSt) of ProteomeHD data for *Homo sapiens* DUF34 homolog (Q9GZT8-2); evidence format of one column per query gene with evidence codes<br>**(c.2)** GSEA (via g:Profiler, g:GOSt) of ProteomeHD data for *Homo sapiens* DUF34 homolog (Q9GZT8-2); list of genes in a query that belong to a particular term (intersections)
**Data Table S11**	Concatenated list of sequences indicated to be possible non-canonical fusions of the DUF34 family:<br>**(a)** master concatenated list of exported domain architecture data of the DUF34 family from Pfam, InterPro, and UniProt (for each method's exported data, annotations were completed for all rows);<br>**(b)** concatenated list of exported sequences for each of the three methods with duplicates removed (duplicates determined relative to the UniProt family-driven method, then, as a secondary option, the InterPro based method);<br>**(c)** counts for assigned classes and subclasses of domain families listed in b
**Data Table S12**	STRING network export generated following the results of Data Table 10; nodes transferred from ProteomeHD output for *H. sapiens* DUF34 homolog, NIF3L1:<br>**(a)** Network data<br>**(b)** Annotations per node<br>**(c)** Values
**Figure S1**	Word clouds generated from titles of focal and non-focal publications listed in Data Table 2
**Figure S2**	Secondary structural annotation by superkingdom using MultAlign-based ESPRIPT analyses
**Figure S3**	Complete DUF34/NIF3 homolog sequence logos across and for each superkingdom (eukaryota, archaea, bacteria) with three tiers of relative conservation

**Figure S4**	Phyre2 generated model of NIF3L1 (*H. sapiens*) structurally aligned with YqfO to illustrate binding pockets, residues differences within and adjacent to the active site
**Figure S5**	Histogram illustrating count per domain length range as a function of superkingdom
**Figure S6**	Motif differences in sequences of the D-G subgroups with and without the IPR015867 HMM profile signature annotation
**Figure S7**	Pairwise alignments of *B. cereus* DUF34 paralogs
**Figure S8**	PCKFA of COGs and COG descriptions. **(a)** Core PCKFA (0.5-1.5% use (%)) for COG descriptions. Color shades indicate distinct functional categories. **(b)** 1-word COG PCKFA. **(c)** 2-word (word pair) COG PCKFA.
**Figure S9**	Abundances of metal ion ligand annotations across published protein structures (PDB 2020) (Data Table 7)
**Figure S10**	Relative abundances of metal-binding proteins per distinct ion across representative operons comparing those of bacteria and archaea to those observed in PDB (PDB 2020; Figure S8) (Data Table 7)
**Figure S11**	Relative abundances of metal-binding proteins per distinct ion as fractions of all encoded proteins across representative operons (again, calculated separately for each superkingdom); abundances as fractions for PDB (PDB 2020) also shown (Data Table 7)
**Figure S12**	**(a-j)** Distributions of GO terms retrieved for each set of top 300 co-expressed genes of eukaryotic DUF34 family members (exported from CoXPresDb; figures retrieved from g:Profiler g:GOSt webserver for each queried gene set):<br>a. NIF3L1 (Q9GZT8, 60491) of *Homo sapiens*<br>b. Nif3l1 (Q9EQ80, 65102) of *Mus musculus*<br>c. Nif3l1 (Q4V7D6, 301431) of *Rattus norvegicus*<br>d. NIF3L1 (F1NDR9, 424076) of *Gallus gallus*<br>e. nif3l1 (Q4KMJ5, 574421) of *Danio rerio*<br>f. anon-35F/36A / CG4278 (Q9NK57, 34970) of *Drosophila melanogaster*<br>g. NIF3L1 (F7B590, 700572) of *Macaca mulatta*<br>h. NIF3L1 (E2RQI7, 607446) of *Canis lupus familiaris*<br>i. NIF3 / YGL221C (P53081, 852651) of *Saccharomyces cerevisiae*<br>j. NIF3 / SPCC126.12 (O94404, 2539063) of *Schizosaccharomyces pombe*
**Figure S13**	STRING network of GSEA output of DUF34 co-regulated genes of *H. sapiens* (ProteomeHD).

1. Supplemental Methods

1.1. Capture of literature, structural, and essentiality data.

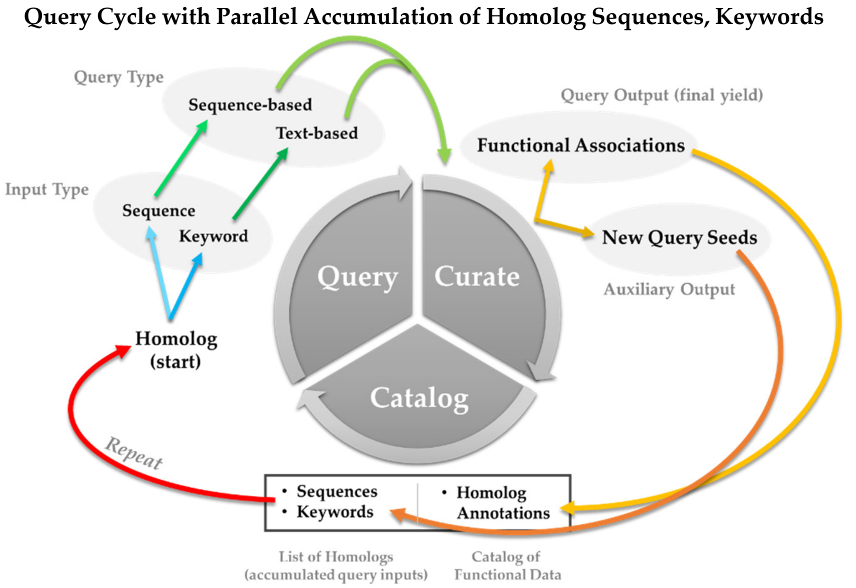

By design, PaperBLAST cannot identify papers that mention homologs within supplemental data or in the main text of publications that are not open access, which can lead to false negatives when in search of published homologs. Additionally, the tool ultimately still relies on a text-based search method with each sequence-based identification of homologs from which the search terms (e.g., ORF names, gene names, UniProt accession IDs) are retrieved, resulting in the risk of false positives. These observed weaknesses supported the impetus for an integrative approach to published homolog capture, using multiple different tools, both text- and sequence-based. The multiplicity of names—a problem inherent to biological sequence identification and nomenclature—presents another challenge for such a pipeline. To address this problem, DUF34 homologs, keywords, and sequences were simultaneously curated from query results for use in subsequent iterations of searches. That is, with each new homolog discovered, new associated keywords, in addition to their sequences, were added to a cumulative list. Each new sequence or keyword was then used in the repetition of sequence- and text-based queries, as well as curation and cataloging. Per seeding homolog, the order in which different tools were used varied per search cycle (one cycle = one sequence or one keyword linked to a sequence), changing in response to the distinguishing characteristics and results observed over the course of a sequence or keyword's query cycle. Resulting publications were manually reviewed for relevancy to the queried homolog, keyword, and/or sequence to identify false positives. If false positives were observed, it was noted that they often were the product of coincidental homonyms used by authors to designate experimental objects, variables, or the like that were ultimately irrelevant to the queried homolog. The cyclic method by which homologs/keywords were curated, accumulated, and used in queries is shown above.

1.2. Physical clustering analysis.

Using the GeConT 3 webserver, the rank-ordered lists of co-occurring COGs were extracted as copied text with each query of Phylum, and, for a select number of Phyla, Class of organisms. Total organism limit for output was set to "All selected", an output total of which varied between queries. Webtool-defined operons were used in generation of gene neighborhood figures and subsequent analyses. Text analysis (PCKFA) of, both, the rank-ordered lists of clustered COGs and respective COG descriptions were performed using textalyser.net/. Punctuation was added to the end of each tabular row (i.e., per queried taxonomic range output) for both COG and COG description lists as for them to be recognized as individual "sentences" by the text analysis application. The 1-, 2-, 3- and 4-word phrases of the COG descriptions were filtered for strings deemed uninformative regarding functional associations or biological roles; these removed words and phrases were recorded upon removal. With the generation of representative operons, individual encoded genes were reviewed and compared between organisms; this analysis included the identification of metal-dependency or interaction. To do so, further literature review and bioinformatic meta-analysis of protein families for the genes identified in COG0327-encoding operons was performed through the text-based query of COGs and common gene/protein family names/terms (e.g., Google, Google Scholar; subsequently, used to navigate to key database entries and publications), as well as sequence-based queries of structure-focused webtools to identify or predict EC numbers (CATH-db [1], HHPred [2], PaperBLAST [3], CDD [4]). Most frequently, sequenced-based queries led to the identification of EC numbers via either CATH-db or KEGG. Regardless of the route used to determine annotated enzymatic activity, if EC numbers were retrieved, they were reviewed in BRENDA [5] for any documented interactions with metal ions. If only one publication was supplied in support of a unique metal ion

interaction for any EC number's BRENDA entry, the interaction was not recorded as valid; this criterion was applied for all BRENDA-acquired annotation data, and implementation of this method applies to select supplemental Data Tables, as well as Tables 4, 5, and 6. PDB entries were also examined for complexed metal ions and evaluated for biological relevance through examination of their associated publications [6], [7]. Auxiliary resources employed in these surveys of functional annotation included UniProtKB, KEGG, PIR [8], EggNOG [9], InterPro [10], and the SMART database [11]. The COG descriptions listed in tables were determined through COG-based query of the NCBI Database of Clusters of Orthologous Genes [12], unless otherwise specified.

1.3. Fusion Analysis.

For the concatenated list resulting from the three distinct fusion-retrieval methods (prior to confidence scoring), InterPro superfamilies and families were used to define fusion *classes*, while InterPro families and domain names were used to define fusion *subclasses*. Additional annotations were added to the sequence records: source organism, organism superkingdom, protein name, gene name/locus tag, UniProt accession ID, as well as all associated COGs (EggNOG, sequence-based query; all root, or "COG", COGs above a score of 20), Pfam and InterPro identifiers (if absent from exported sequence data). For assignment of single COGs to distinct fusion types for the final list: if no COGs were determinable for a given fusion domain, the CDD Clan, Pfam, or InterPro identifier (all retrieved via CDD Search) was used in its place. Multiple identifiers were listed only if multiple domains were found to separately contribute to the sequence alongside respective DUF34 domains.

2. Supplemental Tables

Table S1. All webtools and resources used.

NAME	LINK
**PubMed**	https://pubmed.ncbi.nlm.nih.gov
**Google**	https://www.google.com
**Google Images**	https://images.google.com/
**Google Scholar**	https://scholar.google.com
**NCBI (all Db)**	https://www.ncbi.nlm.nih.gov
**Scinapse**	https://www.scinapse.io
**WorldWideScience**	https://worldwidescience.org
**PaperBLAST**	https://papers.genomics.lbl.gov/cgi-bin/litSearch.cgi
**MonkeyLearn**	MonkeyLearn.com
**TagCrowd**	TagCrowd.com
**PDB/RCSB**	https://www.rcsb.org
**MetalPDB**	http://metalpdb.cerm.unifi.it
**DEG Database**	http://www.essentialgene.org
**Ogee**	https://v3.ogee.info/#/home
**Database of Predicted Essential Genes**	http://tubic.org/pdeg/
**OrthoInspector 3.0**	lbgi.fr/orthoinspectorv3/
**MAFFT**	https://mafft.cbrc.jp/alignment/server/
**WebLogo 3**	http://weblogo.threeplusone.com
**Inkscape**	https://inkscape.org
**PhyloT**	https://phylot.biobyte.de/
**iToL**	https://itol.embl.de/
**MicrobesOnline**	http://www.microbesonline.org/
**STRING**	https://string-db.org/cgi/input.pl
**EggNOG**	http://eggnog5.embl.de/#/app/home
**KEGG - SSDB**	https://www.kegg.jp/kegg/ssdb/
**Gene Context Tool (GeConT) 3**	http://biocomputo.ibt.unam.mx:8080/GeConT/
**CDD**	https://www.ncbi.nlm.nih.gov/cdd/
**PaperBLAST**	https://papers.genomics.lbl.gov/cgi-bin/litSearch.cgi
**HHPred**	https://toolkit.tuebingen.mpg.de/tools/hhpred
**CATH/Gene3D**	http://www.cathdb.info/
**BRENDA**	https://www.brenda-enzymes.org/
**UniProtKB**	https://www.uniprot.org/

**KEGG**	https://www.kegg.jp/
**PIR**	https://proteininformationresource.org/
**InterPro**	https://www.ebi.ac.uk/interpro/
**SMART**	http://smart.embl-heidelberg.de/
**Database of Clusters of Orthologous Genes**	https://www.ncbi.nlm.nih.gov/research/cog
**CoXPresDb**	https://coxpresdb.jp/
**ProteomeHD**	https://www.proteomehd.net/proteomehd
**g:Profiler – g:GOSt**	https://biit.cs.ut.ee/gprofiler/gost
**DAVID – Functional Annotation Clustering**	https://david.ncifcrf.gov/summary.jsp
**HUGO Gene Nomenclature Committee, EBI**	https://www.genenames.org/
**SEO Scout**	textalyser.net/ (https://seoscout.com/tools/keyword-analyzer)
**EMBOSS Needle**	https://www.ebi.ac.uk/Tools/psa/emboss_needle
**EMBOSS Matcher**	https://www.ebi.ac.uk/Tools/psa/emboss_matcher
**Harmonizome**	https://maayanlab.cloud/Harmonizome/
**JGI-IMG/M**	https://img.jgi.doe.gov

Table S2. Lists of strains and oligos used in growth assays.

Strains	**Name**	**Genotype**	**Plasmid**	**Note on Plasmid**
	MG1655	F- λ- ilvG- rfb-50 rph-1	none	
	GJH1072	F- mrcA D(mrr-hsdRMS-mrcBC) F80dlacZDM15 DlacX74 endA1 recA1 D(ara, leu)7697 araD139 galU galK nupG rpsL l T1R	pBAD24	
	GJH1358	F- mrcA D(mrr-hsdRMS-mrcBC) F80dlacZDM15 DlacX74 endA1 recA1 D(ara, leu)7697 araD139 galU galK nupG rpsL l T1R	pGH50	ybgI in pBAD24
	GJH1132	MG1655	pBAD24	
	GJH1227	MG1655 folE::kanR	none	
	GJH1289	GJH1227	pBAD24	
	GJH1361	MG1655 ybgI::kanR	none	
	GJH1408	GJH1227	pGH50	ybgI in pBAD24
	GJH2042	MG1655	pGH50	ybgI in pBAD24
	GJH2435	MG1655 ybgI::FRT	none	
	GJH2438	MG1655 ybgI::FRT folE::kanR	none	
	GJH2441	GJH1361	pBAD24	
	GJH2444	GJH1361	pGH50	ybgI in pBAD24
	GJH2447	GJH2438	pBAD24	
	GJH2450	GJH2438	pGH50	ybgI in pBAD24
	GJH2453	F– Φ80lacZΔM15 Δ(lacZYA-argF) U169 recA1 endA1 hsdR17 (rK–, mK+) phoA supE44 λ– thi-1 gyrA96 relA1	pGH101	folE in pBAD24
	GJH2454	MG1655	pGH101	folE in pBAD24
	GJH2457	GJH1361	pGH101	folE in pBAD24
	GJH2460	GJH1227	pGH101	folE in pBAD24
	GJH2463	GJH2438	pGH101	folE in pBAD24

Oligos	**Name**	**Sequence**	**Description**
	GO285	GCACGCCATGGAAAACACCGAACTGGAAC	NcoI + start of ybgI E. coli
	GO286	GCACGCCTGCAGGTTATGCAGGATTAGGAATATCAATAAAGG	SbfI + end of ybgI E. coli
	GO434	GCACGCCATGGCATCACTCAGTAAAGAAG	NcoI + start of folE E. coli
	GO435	GCACGCCTGCAGGTCAGTTGTGATGACGCACAG	SbfI + end of folE E. coli
	GO563	ACTGGCGAGGTAACGCTATG	verification ybgI deletion forward external
	GO564	CCACATTCGGCATATCCACC	verification ybgI deletion reverse external
	GO565	TGCAGGTGGAAGGCAAAGAG	verification ybgI deletion reverse external
	GO566	GCGAATACCACCACGTTCAG	verification ybgI deletion reverse external

GO567	ATGCGTGGCTCCTTGTTGTG	verification folE deletion forward external
GO568	GCCGATGAGATCAAGAAATGC	verification folE deletion forward external
GO569	ATCTCGACCTGGCTGATGAC	verification folE deletion forward external
GO570	CAATCCACCAAGAGAGGTCG	verification folE deletion forward external

Table S3. All focal and non-focal publications collected through comprehensive literature review and data capture process of the DUF34 protein family. Includes each publication's functional implications/associations for DUF34 family members. See Supplemental References section for cited literature.

NAME	ORGANISMS	PHENOTYPE, BIOLOGICAL RELEVANCE	REF	FOCAL
MIMI_R836	*Acanthamoeba polyphaga mimivirus*	(COG3323) ORF acquired by Mimivirus from bacteria via horizontal gene transmission; ORF is shared exclusively with bacteria (not eukaryota or archaea)	[13]	No
PRK_00227/ HMPREF0010_02337	*Acinetobacter baumannii*	Significantly down-regulated (-1.07 Log2 fold-change; FDR = 1.75E-03) after exposure to human respiratory cells (A549 cells)	[14]	No
Y1Q_0008769	*Alligator mississippiensis*	Notably enriched in both embryonic and juvenile hearts of alligators exposed to developmental hypoxia	[15]	No
AFUA_6G12480	*Aspergillus fumigatus*	Notably upregulated in *Δpes3* mutant relative to WT germlings (1.7-fold change)	[16]	No
An05g01490	*Aspergillus niger*	Notably upregulated (+1.08698 log2-fold change) in *ΔfluG* relative to controls in most central growth zone; identified as protein with signal sequence for secretion	[17]	No
BA4512	*Bacillus anthracis str. Ames*	Identified in cytoplasmic proteome	[18]	No
		Low antisense (AS) transcriptional abundance (operon containing homolog); upstream operon encoding *sigA*, *dnaG* show abundance AS transcripts	[19]	No
YqfO/ BC_4286	*Bacillus cereus*	Inserted domain with similarity to PII-like and CutA1 family proteins; present in a minority of bacterial homologs; may act as a signal sensor for regulating catalytic activity or access to active sites	[20]	Yes
YqfO/ BSU_25170	*Bacillus subtilis subsp. subtilis str. 168*	Secreted; greatest inhibition of Fusarium oxysporum growth	[21]	No
		Co-regulator with YlxR of operon *tsaEBD* (TsaEBD req. for synthesis of t^6A[22]); disruption of *yqfO* impairs *tsaEDB* regulation, resulting in loss of glucose induction of *sigX* through PDHc expression dysregulation	[23–25]	Yes/No(2)
		Co-purified with RecN (small polypeptide fraction, 0.1 ±0.1% relative proportion of M_R = 520000 RecN peak), alongside small proportions of AhpC, PncB, PNPase GroEL	[26]	No
		Upstream of region predicted to have high riboswitch potential	[27]	No
		Predicted to be regulatory target of sigma factor A (σ^A; RNAP major σ factor) by best-fit of gene expression profile to σ^A-regulated model of expression	[28]	No
ybgI/ M139_2446	*Bacteriodes fragilis S23L24*	14th gene downstream from Type IIIB CRISPR-Cas system array; neighborhood noted for high percentage of genes involved in efflux	[29]	No
A0A0A2VS40	*Beauveria bassiana*	Quantified secreted protein of *B. bassiana* isolates grown in preparations of cockroach-infused liquid medias; absolute peptide count = 2 fmol/μL (ANOVA (p) = 4.04E-10)	[30]	No
BL0984	*Bifidobacterium longum NCC2705*	Identified in proteome of whole cell protein extract; mass fingerprinting score greater than 45	[31]	No
BmNIF3l	*Bombyx mori*	Translocates to the nucleus from the cytoplasm when cell treated with all-trans retinoic acid (ATRA); higher transcript levels in actively differentiating tissues; no expression detected in egg stage	[32]	Yes
bb0468	*Borrelia burgdorferi*	Described as a Lac I homolog (**false; confirmed with BLAST*); likely not a regulator of p66 expression (importance: *p66* may influence host-response during infection)	[33]	No
		Significantly upregulated after infection of human H4 neuroglial cells (+2.17 mean fold-change); no listed paralogous gene families	[34]	No
		46th of 297 genes indicated as being regulated by Hk1-Rrp1	[35]	No

NIF3L1	*Bos taurus-Bos indicus hybrid*	Significantly differentially expressed in Wangdong cattle (Bos taurus-B. indicus hybrid) relative to Dabieshan cattle (4 independent loss of function variant analyses)	[36]	No
NIF3L1	*Bos grunniens, Bos taurus, Bos taurus-grunniens hybrid*	Protein not notably differentially expressed in dzo relative to cattle; downregulated in yak relative to dzo, relative to cattle; investigation intended to identify proteins with adaptive value for life in low oxygen environments to which yak have been evolutionarily adapted	[37]	No
ybgI	*Buchnera sp.*	Present across *Buchnera* species commonly found in both *Aphidinae* aphids and *Pentalonia nigronervosa*	[38]	No
		One of 13 genes across four *Buchnera aphidicola* Mp genomes to contain nonsynonymous mutations; (of those 13) one of seven predicted to have a deleterious effect upon protein function; deleterious mutations in *ybgI* were detected in both non-tobacco-adapted and tobacco-adapted lineages	[39]	No
BTH_I2978	*Burkholderia thailandensis E264*	Encoded downstream of Tat pathway genes; divergently encoded relative to serine protease gene, *degS* (BTH_I2979; deletion of this gene results in robust meropenem nontolerance phenotype [40])	[41]	No
Cj0705	*Campylobacter jejuni*	Transcripts upregulated (1.8-fold) in presence of 0.1% sodium deoxycholate	[42]	No
		Co-occurs in operon encoding crucial virulence-linked chemotaxis proteins (i.e., CheZ)	[43]	No
		High similarity shared between operons of select bacterial pathogens; operon constituents linked to flagellar components, chemotaxis, virulence	[44]	No
		Internal promoter enables transcription of both Cj0705 homolog and downstream Cj0706; fusion of homolgos occur in other Epsilonproteobacteria to provide similar transcriptional behavior	[45]	No
CPn0137	*Chlamydia pneumoniae*	Identified in human saliva with 95-99% confidence; 1 of many microbial proteins detected in human saliva samples	[46]	No
CT_108	*Chlamydia trachomatis D/UW-3/CX*	Demonstrated low potential as a secreted effector (SIEVE score = -1.94748)	[47]	No
		Base 608 variant (C→T; Valine→Alanine) determined via clinical sample GWAS (n=5 of 74 genomes/samples) examined as a possible candidate in predisposition of failure in *C. trachomatis* infection treatment	[48]	No
CBO2935	*Clostridium botulinum A str. ATCC 3502*	One of the top 40 differentially expressed genes during heat shock stress (+4.1-fold change)	[49]	No
		Greatest differential expression post-heat shock observed at 1 hour (+1.4 log2 fold-change)	[50]	No
CD630_06720/ CDR20291_1306	*Clostridium difficile*	Experienced 16% decreased transcript level in *ΔcodY*; characteristically increased in transcript level with F101W and F74Y *codY* point mutations relative to what was observed on average across transcripts, unexpectedly higher than levels anticipated on average in *ΔcodY*	[51]	No
		One of 124 genes significantly negatively regulated by c-di-GMP	[52]	No
		Gene disrupted in same fashion for two pathogenic isolates (Opal, TGA selenocysteine incorporation codon [tga]), distinct from WT	[53]	No
		Detected in soluble fraction of proteome; not predicted to be a lipoprotein	[54]	No
		Significantly under-expressed (1.9 log2 fold-change; adj. p-value = 1.70248E-28) in JIR8094::*ΔsinR′* relative to JIR8094 (note*: *codY* was not differentially regulated, but *ccpA* was upregulated ~13.4-fold)	[55]	No
DIP1680	*Corynebacterium diphtheriae*	Gene neighbor, DIP1679, is co-transcribed with ncRNA, RNase P M1 RNA, as a product of a primary operon also containing DIP1678 and DIP1677; this primary operon is expressed as two sub-operons (DIP1678-DIP1677; M1 RNA-DIP1677)	[56]	No
Cp1002B_1165	*Corynebacterium pseudotuberculosis biovar ovis*	One of 80 hypothetical proteins identified (of 172 predicted) and assigned a putative function through sequence-, annotation-based analyses; predicted to be localized to the cytoplasm; predicted to be metal-binding (GO term analysis); predicted to not occur in a pathogenicity island; not differentially expressed in abiotic stress conditions (osmotic, acid stress; thermal shock)	[57]	No
I79_014620	*Cricetulus griseus*	1 of 9 Host cell proteins (HCPs) detected in cell culture fluid exclusively under condition 2 (starvation conditions; "glucose and ion limitation"); 0.9% of 994 host cell proteins identified in cell culture fluids under	[58]	No

		defined analytical confidence parameters; condition 2 only described as having lowered glucose feed rate and reduced concentration of a single, unnamed, essential ion regarded as a common enzyme cofactor (proprietary)		
Dde_1729	*Desulfovibrio desulfuricans G20*	Significantly dysregulated in *psrA* mutants (+1.6 fold-change; p-value = 0.01)	[59]	No
		One of 4 slow-growing mutants [lactate-sulfate medium]; implicated as essential for growth in sediment (mutant failed to demonstrate growth upon inoculation)	[60]	No
DMEL_CG4278/ DS02740.16/ anon-35F/36A	*Drosophila melanogaster*	Identified, mapped within the *Adh* genomic region; may have an allele encoded by a nearby P-element insertion (k17003) 1185 bp upstream of anon35F/36A	[61]	No
		Encoded immediately 3′ of the *cactus* gene (FBgn0000250; IκB homolog)	[62]	No
		Identified as a component of the Seryl-tRNA synthetase-Like Insect Mitochondrial Protein (SLIMP) interactome, SLIMP being a novel paralog of mitochondrial Seryl-tRNA Synthetase (SerRS2)	[63]	No
YbgI/b0710	*Escherichia coli*	Identified as one of 10 "unknown unknown" genes prioritized for characterization	[64]	No
		Resolution of structure; homohexameric toroid quaternary structure; each monomer displays a di-nuclear metal ion-binding site; likely involved in DNA repair	[65]	Yes
		Members of operon, regulon (*pxpABC/ybgJKL*) characterized to constitute requisite steps of 5-oxoproline metabolism (in prokaryotes); data applies to *B. subtilis* for this association, but ybgI homolog is absent from this operon	[66]	No
		Mutant: sensitivity to chemically-induced ss-/ds-DNA, -RNA lesions	[67]	No
		Cytosolic subcellular localization	[68]	No
		Decreased survival when mutant subjected to IR	[69]	No
		No survival impairment when mutant subjected to UV radiation; co-localized at poles (during cell division; localized w/ PstB and TktA); GlmS likely interaction partner; sensitivity to antibiotics affecting cell wall synthesis	[70]	Yes
		Predicted GO annotation: (BP) Molybdopterin cofactor metabolic process; (MF) hydrolase activity, acting on acid anhydrides, in phosphorus-containing anhydrides	[71]	No
		Predicted binding sites compatible with role in DNA metabolism	[72]	No
		Ranked 63/4290 in ethanol gene response (ethanologenic *E. coli* strain)	[73]	No
		Expression notably activated by paraquat	[74]	No
		Significantly increased abundance (+3.1 fold-change) in response to vanillin	[75]	No
		Determined theoretical values of pI, MW (5.07, 26,892.48, respectively)	[76]	No
		Observed to bind (as a 20bp sequence fragment) with MarA, SoxS and Rob (K_d(nM^{-1}) = >1000, >1000 and 750, respectively)	[77]	No
		One of 3 protein domains (det. via Pfam; NIF3, p-value = 0.00203) indicated as being preferentially recognized by both cathelicidin-derived antimicrobial peptides, Bac7 (bactenecin 7) and PR-39 (proline-arginine-rich peptide 39); these two antimicrobial peptides indicated to likely interact with proteins involved in aspartate family amino acid catabolism/metabolism (GO enrichment of 82 hits), proteins involved in fructose/mannose metabolism and terpenoid backbone biosynthesis (KEGG analysis of 82 hits)	[78]	No
		Predicted to function as an alpha-/beta-amylase (residues: H63, H64, H97, H215, E194*, E219, D101, N108, C171, Y22, W68) via structure-based prediction	[79]	No
		One of 4 purported stress response genes differentially expressed by >-2-fold with exposure to 6-HO-BDE-47; behavior of longitudinal expression across 3 distinct concentrations (0.09, 0.9, 9 mg/L) was most similar to *ydiV*, *dcd*, and *pepD* (cluster diagram, distance kernel = Manhattan)	[80]	No
		Experimentally validated to be bound by σ^{70} (aerobic conditions) post-prediction of binding events by dPeak analysis of σ^{70} PET ChIP-Seq data	[81]	No
		Shown to interact with TorR (cell lysate); found to co-localize at the cell poles with OmpT, MalF, PstB, YcfH and OmpR (GFP-fusions)	[82]	No

		Notably elevated expression of operon upon *sdiA* amplification (>2.1-fold increase across operon); linked to *sdiA*-mediated mitomycin C resistance; correlated with AcrA/B-mediated efflux (>2.0-fold increase)	[83]	No
		Identified as one of 6 proteins identified to only survive trypsin digestion (cell lysate digested with 0.40 mg/mL trypsin for 4 days)	[84]	No
		Listed as being localized to the inner membrane; significantly induced (>40% change) alongside 10 other proteins with the depletion of EcfE, an essential inner membrane-anchored protease regulated by $E\sigma^E$	[85]	No
		Predicted regulation target of cAMP-CRP, divergently transcribed with *ybgH*	[86]	No
		Downregulated (>2-fold) in WT FADD (Fas-associated death domain)-DED(death effector domain)-expressing cells	[87]	No
		Notably down-regulated in ESBL (extended spectrum beta-lactamase) gene carrier when incubated under bentonite condition (p<0.05)	[88]	No
		Transcribed with *nei* (endonuclease VIII) as a component of the same operon, of which also contains no internal promoters; this operon partner, *nei*, can compensate for the absence of *nth*, another endonuclease (III)	[89]	No
		Mutant: UV sensitivity = "slightly"	[90]	No
		Significant differential expression observed in two of three strains (EC100, DH5-alpha; RM109 was the exception) upon (Aminomethyl)phosphonic acid (AMPA) but not glyphosate treatment	[91]	No
		Describes how CutA family was identified as a group within the PII superfamily through use of COG3323-member NIF3 proteins	[92]	No
Eab7_0807	*Exiguobacterium antarcticum strain B7*	Indicated as one of 132 hypothetical proteins functionally annotated in genome; predicted to be cytoplasmic, not containing transmembrane or signal peptide domains	[93]	No
FN1791	*Fusobacterium nucleatum subsp. nucleatum*	Gene fusion with NUDIX family protein (pfam00293); convergently encoded downstream relative to *ptsH-ptsI*, contributing genes to the phosphoenolpyruvate:carbohydrate phosphotransferase system (PTS) (operators of which co-regulate CcpA in Firmicutes)	[94]	No
NIF3L1	*Gallus gallus*	Downregulated in ileal tissue of H9N2 AIV-infected chickens relative to uninfected controls after 12 hours, increased relative to controls after 24 hours of infection	[95]	No
XynX	*Geobacillus stearothermophilus*	*xynX* negatively regulates expression of *xynA* (encodes a secreted xylanase), the latter of which is encoded divergently relative to *xynX*	[96]–[98]	Yes/No(2)
		xynX may be negatively regulated by XylR	[96], [98], [99]	Yes/No(2)
		Leading gene encoded within operon containing *axe2* (acetylxylan esterase) and *xynB3* (β-xylosidase)	[100], [101]	No(2)
GSU3085	*Geobacter sulfurreducens*	Significantly downregulated (−1.227 ± 0.246 (log2-fold ratio ± SD)) in OmcB-deficient mutants grown in acetate-limiting Fe(III) citrate-grown continuous culture	[102]	No
HD1471	*Haemophilus ducreyi*	Co-transcribed with *mazG* (nucleoside triphosphate pyrophosphohydrolase), *cpxR* and *cpxA* (the latter pair compose the organism's only two-component regulatory system, a system that represses key virulence genes[103])	[104]	No
		Expression levels were unchanged in *ΔcpxA*	[105]	No
		In stationary phase, significant differential expression observed in *ΔcpxA* (fold-change = -2; p-value = 6.90E-15)	[103]	No
		+3.54 fold-change in *Δhfq* (inactivated insertion); -3.5 fold-change stationary phase relative to mid-log phase	[106]	No
HI0105	*Haemophilus influenzae*	Identified as a conserved hypothetical protein by sequence-based functional characterization; emphasized point: functional differences between conserved unknowns are likely to occur between disparate taxa	[107]	No
		15 bp at the 3'-end of gene overlapped by a 46-bp repeat sequence, NTHI0188, a gene characterized as "M enzyme type I" (part of a restriction-modification (RM) gene set: NTHI0188, NTHI0190, NTHI0192, NTHI0193); links NIF3 homolog to protective cell features assoc. with methylation and the digestion of foreign, unmodified DNA	[108]	No
		Listed as being one of many hypothetical proteins to have some form of functional annotation	[109]	No

		No cleavage by GlpG observed (*glpG* mutant demonstrating hyperactivity)	[110]	No
HVO_2298	*Haloferax volcanii*	Located in operon with genes pivotal to polyamine metabolism, the modification of translation initiation factor 5A (IF5A)	[111]	No
		Notable differential expression in *ΔglpR* relative to WT under two growth conditions: glycerol= -0.14 [Log10 transformed ratio], SD= 0.58; glucose= -0.33 [Log10 transformed ratio], SD= 0.33	[112]	No
HP0959	*Helicobacter pylori*	GTP-binding, ostensible cyclohydrolysis *in vitro* (source of "GTP cyclohydrolase I type 2" annotation)	[113], [114]	Yes/No
		Adjacent *flgZ* (HP0958), an essential motility gene (shared operon)	[44]	No
		Not required for RpoN stability or motility; zinc-ribbon domain of adjacent gene, HP0958, req. for RpoN accumulation	[115]	No
NIF3L1/ ALS2CR1/ CALS-7/ MDS015/ My018	*Homo sapiens*	Ubiquitously expressed throughout embryonic development, throughout tissues; strong over-expression in spermatogonia-derived, teratocarcinoma cell lines; isolated, characterized; cytosolic subcellular localization; highly conserved N-, C-terminal regions; shares inserted region of its murine homolog	[116]	Yes
		NIF3L1 interacts with its splice variant, NIF3L1 BP1 (THOC7), colocalizing in the cytosol; C-terminal leucine zipper-like domain mediates variant interaction; not indicated to act as a repressor in NIH3T3 cells; binding partner, NIF3L1 BP1, differs in localization due to additional passive presence in the nucleus	[117]	Yes
		Differentially upregulated in osteoporosis (top 20 DEGs; blood)	[118]	No
		Located within a low-risk breast cancer locus region	[119]	No
		Within homozygous consensus region significantly overrepresented in late-onset Alzheimer's disease	[120]	No
		Listed as biomarker of Amyotrophic Lateral Sclerosis (ALS) for systematic analyses	[121]	No
		Retinoic acid-induced binding & cooperative translocation with Trip15/CSN2 from the cytosol to the nucleus (early neuronal development; results in gene silencing of Oct-3/4, a suppressor of cell differentiation); ubiquitously expressed across tissues, important for neuronal development	[122]	Yes
		Described as a transcriptional regulator, identified (by RIP, RNA immunoprecipitation) as 1 of many RNA interacting with APE1 (IPA, Ingenuity Pathway Analysis)	[123]	No
		1 of top 15 Fas/Fas ligand pathway polymorphisms in Fluid and Catheter Treatment Trial linked to acute kidney injury (AKI) in Caucasians (odds ratio = 2.36 (1.15-4.88); p-value = 0.02; FDR = 0.67; MAF (minor allele freq.) = 0.10)	[124]	No
		Significantly (several SNPs) associated with triptolide IC50 (concentration resulting in death of 50% of cells) in LCLs (lymphoblastic cell lines) ($p < 0.00001$; GWAS)	[125]	No
		Binding partner NIF3L1 BP1 is 1 of few genes affected by epidermal growth factor (EGF) to first-trimester decidua cultures (FTDC) (downregulated; fold change = -7.4), illustrating a reciprocal negative interaction of EGF & PIF (preimplantation factor)	[126]	No
		Binding partner, NIF3L1 BP1, interacts with FMIP (Fms-interacting protein; both members of the THO complex, involved in mRNA processing), which localizes to the nucleus, cytoplasm; FMIP is required for the localization of NIF3L1 BP1 to the nucleus	[127]	No
		Significantly decreased protein abundance (mean fold change = 0.136) in human corneal stromal keratocytes (CSKs) supplemented with cryopreserved human amnion extract (C-AME) compared to F-AME (fresh AME); C-AME-driven downregulation correlated with reduced proliferation rate of CSKs in culture	[128]	No
		Significantly differentially regulated in two major sCJD (sporadic Creutzfeldt-Jakob disease)-associated variant genotypes of PRNP (human prion protein) gene, PrP^{Sc} type 1 (MM1; fold change = 6.8; increased; p-value = 0.0231) and PrP^{Sc} type 2 (VV2; fold change = 3.2; opposite; p-value = 0.1423)	[129]	No
		Identified as being of low-abundance and inconsistently detectable in erythrocytes, absent in platelets and plasma	[130]	No

Not significantly altered in *Plasmodium falciparum* trophozoite stage parasites extracted from human red blood cells treated with either 2 ozonides (OZ277, OZ439) nor DHA relative to untreated parasites (parasite + host-derived proteome)	[131]	No
Described as being 701 S/G2-M phase-regulated despite its lack of characterization	[132]	No
Included in 1 of the deletions presenting in 23 PAH (pulmonary arterial hypertension) cases affecting the BMPR2 (bone morphogenic protein type 2 receptor) locus	[133]	No
Present in brain, spinal cord and lymphocytes; detected as two distinct transcripts with similar patterns of expression; highest levels of both transcripts presented in the heart, skeletal muscle and testis; smaller transcript was expressed at a higher level than the other; no deletions or polymorphisms linked to ALS patients when compared to controls; 1 of 6 candidates eliminated as being causatively linked to ALS2	[134]	Yes
Observed notable detection in sporadic sarcoma tissue (difference = 0.339; p-value = 0.0052; frequency = 66.8) but not in radio-induced sarcoma tissues	[135]	No
Binding partner (NIF3L1 BP1/THOC7) linked to negative regulation of innate antiviral immunity through inhibition of TBK1 (via interaction, ubiquitin-dependent degradation)	[136]	No
Within locus significantly associated with mast cell degradation by silver nanoparticles (AgNP) (adjusted p-value = 0.00431)	[137]	No
Decreased expression with TET1 knockdown positively correlated to upregulated methylation in 5' regions (transcription start sites, 1st exons)	[138]	No
Correlated with lapatinib sensitivity of breast cancer cell lines *in vitro* (HER2-targeted therapy) (r(Spearman) = 0.490196; p-value = 0.0389), SNV (single nucleotide sequence variant) variant type associated with HER2-positive tumors	[139]	No
Significantly increased relative abundance in nucleus (fold of regulation = 70.76; log2 = 6.14; Anova (p) = 0.00) in muscle cells deficient of functional laminin-211 (patients with congenital muscular dystrophy type 1A (MDC1A) (mutant, recessive; LAMA2, alpha 2 subunit of laminin-211))	[140]	No
1 of 9 genes strongly correlated with both metastatic lesions and primary tumors, highly upregulated in metastases	[141]	No
1 of 4 hypermethylated, significantly differentially expressed genes shared between two cancellous bone specimen groups, osteoarthritis & osteoporosis	[142]	Yes
1 of 89 direct target genes of estrogen receptor regulation (breast tumor cells); predicted to possess ERE (estrogen response element) in *cis*-regulatory region; of minority of genes with ER-regulation targeted orthologs with EREs in mice	[143]	No
Predicted to affect risk for ASD (autism spectrum disorder) (de novo loss-of-function mutations = 0; false discovery rate (q-value) = 0.0025-0.025)	[144]	No
mRNA interacts *in vivo* with RBBP8 (*z*-score = 19) in HuH-7 cells (HuProt array)	[145]	No
Showed significantly differential pattern of expression across cell lines following high dose of gamma radiation (≥8 Gy) but not after low dose (≤2 Gy)	[146]	No
Cooperatively with 14-3-3 regulates the transcriptional activity of Wbscr14 by preventing its nuclear localization through complex formation (Wbscr14 participates in complexes activating transcription of lipogenic enzymes, promoting fat accumulation)	[147]	Yes
Notably decreased in expression across lung CD103+ resident memory T cells, while substantially increased in peripheral blood T cells (i.e., naive, central memory and effector memory T cells)	[148]	No
(In βTC-tet cells) binds to Clic4, a cytokine-induced gene in pancreatic β-cells linked to cytokine-induced apoptosis	[149]	No
Decreased gene expression relative to healthy controls linked to myocardial infarction (MI), possible pathology biomarker	[150]	No

Ranked within the top 20 genes most significantly correlated with MRPS30 (elevated gene expression of MRPS30, ER-positive risk allele neighbor, is exclusively linked to ER-positive tumors)	[151]	No
Nearest gene (in add. to PPIL3) to genomic methylation location linked to prenatal maternal stress exposure in neonates through EWAS meta-analysis (2nd in list of ascending P-values)	[152]	No
Differential expression was notable in peripheral blood samples where PFOS (perfluorooctane sulfonate, an environmental pollutant) was determined "high"; differential gene sets were enriched for contributors to the citrate cycle pathway	[153]	No
In top 10 ranked genes in combined analysis of 4 disease-associated echocardiographic traits (left atrial size, left ventricular internal dimension, left ventricular mass) & Alzheimer's Disease through genome wide association meta-analysis, gene-wise statistics calculation	[154]	No
Downregulation in-parallel with demethylation genes, upregulation of methyl-transferase genes linked to hyper-methylation, histone modification; associated with ventricular septal defects (VSD) in monozygotic twins with trisomy 21	[155]	No
Notably suppressed in BL2 cells (transformed germinal centre B cells) *in vitro* after αIgM treatment	[156]	No
Shown to significantly activate autoantibodies (p-value = ≤0.05)	[157]	No
Shown to interact directly with the tax and rex proteins of HTLV-1 (Human T-cell Leukemia virus type-1), as well as tax2 and rex proteins of HTLV-2 (Human T-cell Leukemia virus type-2); interaction implicated in post-transcriptional export of viral mRNAs from the nucleus to the cytoplasm	[158]	No
listed as contributing gene within a novel exomic copy number variant (CNV) >100 kbp (duplication) linked to independent cases of specific language impairment (genes: *fam126b*, *ndufb3*, *nif3l1*, *orc2*)	[159]	No
Expression linked to role in embryonic stem cell pluripotency	[160]	No
Contributes to interaction network of JAK2/STAT3 signaling pathway; predicted to be regulated by miRNAs of genes associated with JAK2/STAT3 pathway	[161]	No
Decreased expression correlated (by co-expression module; FDR <0.05)) to Timothy Syndrome in vitro, in vivo (NIF3L1, LIN28A, TEX10, AMMECR1, NCAPG); co-expression module linked to cell cycle phase, cell division, nuclear division by enrichment	[162]	No
Copy number variant (CNV) linked to cases of spontaneous premature ovarian failure (POF) (carriers among cases = 0.33%; carriers among controls = 0.03%) (genes: *satb2*, *ftcdnl1*, *c2orf69*, *tyw5*, *c2orf47*, *spats2l*, *kctd18*, *sgol2*, *aox1*, *aox2p*, *bzw1*, *clk1*, *ppil3*, *nif3l1*, *orc2*, *fam126b*)	[163]	No
Significantly repressed through the p53-LIN37/DREAM pathway (adj. P <0.001)	[164]	No
Unlikely to be regulated by variant regulatory elements (REs) associated with atrial fibrillation; highly expressed in heart tissue	[165]	No
Significantly increased abundance detected in inflammasome complexes after treatment of HK1/ASC cells with H2O2, but not after treatment with poly (dA:dT) or EBER (Epstein-Barr virus noncoding RNAs) [inconsistent with: pathogen response; consistent with: oxidative stress response]	[166]	No
Large copy number variant (CNV) linked to case(s) of autism spectrum disorder (ASD) (genes: *aox1*, *bzw1*, *c2orf47*, *c2orf60*, *cflar*, *clk1*, *fam126b*, *flj38973*, *kctd18*, *loc26010*, *ndufb3*, *nif3l1*, *orc2l*, *ppil3*, *satb2*, *sgol2*)	[167]	No
Identified as a driver gene of adenocarcinoma of the ovary grade 2 (significantly correlated, differential expression)	[168]	No
Differentially upregulated in endolysosome fractions (7.5-TLR3 cells) upon infection with hepatitis C virus (HCV); not implicated in HCV-induced antiviral gene expression	[169]	No
HLA surface expression unaffected with the deletion of TAP (deletion of TAP reduces this expression 5x)	[170]	No
Strongly associated with insulin resistance in human male subjects (as antibody target) (insulin resistance prevalence = 44.44%; insulin sensitivity prevalence = 5.56%; p-value = 0.003399)	[171]	No

Differentially upregulated in FEMX-I and FEMX-V (melanoma cell lines) that were not differentially upregulated in their respective xenographs (fold change = 5.10; p-value <0.05)	[172]	No
Differential gene expression (subnetwork/cluster; log-rank test p-value = 0.003697) in glioblastoma multiforme (GBM) patients linked to expected survival prognosis	[173]	No
Substantially decreased expression upon treatment with MNPs (metallacage-loaded nanoparticles) (U87MG, human glioblastoma cell line; relative to untreated)	[174]	No
Included in 7.5-Mb interstitial deletion on 2q32.3-33.1, containing 28 genes of a patient diagnosed with SATB2-Associated 2q32-q33 microdeletion syndrome (severe developmental disorder)	[175]	Yes
Significantly associated with triptolide chemosensitivity (lymphoblast cell lines)	[176]	Yes
COPS2 point mutations consistent with NIF3L1-COPS2 co-repression relationship (limited; pathogenesis assoc.: S120C, N144S, Y159H, R173C)	[177]	Yes
Referenced as the binding partner of THOC7 or NIF3L1-binding protein 1; described as a transcriptional repressor	[136]	No
Identified as a putatively secreted protein of laryngeal tumors; one of 17 genes demonstrating increased expression in patients with shorter survival	[178]	No
Referenced in review summary as one of several genes with variants associated with increased breast cancer risk	[179]	No
One of four genes downregulated DEGs in medial temporal gyrus (MTG), superior frontal gyrus (SFG), primary visual cortex (VCX) in brains of Alzhaimer's disease patients relative to controls	[180]	No
Significantly downregulted in heart tissues of patients undergoing off-pump coronary artery bypass graft (OPCABG) procedure and were treated with Sevoflurane (relative to control patients undergoing same procedure)	[181]	No
Predicted one of nine mRNA prognostic markers in hepatocellular carcinoma (survival analysis; P<0.01), high expression of was associated with poor prognosis	[182]	No
Indicated as one of several genes targeted by microRNA hsa-miR-384 (of which is competitively inhibited by circRNA hsa-circ_0001338 via binding microRNA response elements); upregulation of this circRNA implicated in the development and progression of hepatocellular carcinoma	[183]	No
Identified as an intracellular idiopathic pulmonary fibrosis (IPF)-specific antigen targeted by auto-antibodies; upregulated in IPF lesions	[184]	No
Significantly increased expression and interaction with APE1 associated with poor prognosis in colorectal cancer	[185]	No
Differentially expressed >50% (>1 log fold-change) in ON-class rod bipolar cells (cells that release neurotransmitters in response to increases in illumination)	[186]	No
Contained within one of 148 correlated gene modules (CGMs, clusters of 10-200 genes highly correlated with each other): cluster 35 (183 genes), enriched for genes involved in cell cycle	[187]	No
Indicated (via whole genome sequencing data derived from patients affected by polycystic ovary syndrome) to have >2.0 log of the odds ratio (LOD) of allele-sharing probability between affected relative pairs relative to discordantly affected relative pairs (high heritability, disease status associated)	[188]	No
Contributor to protein-protein interaction network (p-value=0.018) of top 300 hub genes from intracellular transport/autophagy module (SC.M2) indicated as ALS genetic risk factors; not highlighted as a having annotation indicating relevance to ALS pathophysiology	[189]	No
One of 56 genes indicated as being regulated in two separate carcinoma cell types by distinct transcription factors SOX2 (TF, embryonal carcinoma cells) and SOX17 (TF, seminoma cells); also target of NANOG in embryonic stem cells	[190]	No
Clustered with 24 other genes based on an ingenuity pathway analysis (QIAGEN IPA), implicated in cardiovascular disease; cluster labeled	[191]	No

		"Cellular Movement, Hematological System Development and Function, Immune Cell Trafficking"		
		One in 108 proteins in MCF7 and 111 proteins in MDA-MB231 (breast cancer cell lines) significantly downregulated in mitochondria upon treatment with TNF-α	[192]	No
		Found to have altered expression in two Marinesco-Sjogren syndrome models (up in humans, down in mice): SIL1-KD HEK293 cells, as well as Woozy mouse sciatic nerve cells	[193]	No
NIF3L1	*Idiosepius paradoxus*	Significantly differentially expressed in embryonic head (including brain, eye tissue) relative to body tissue (remaining embryo) (upregulated; fold-change = 71.51518)	[194]	No
CEN27895.1	*Lactococcus piscium*	Downregulated at 185 minutes of incubation at 0ºC (cold shock treatment)	[195]	No
LDBND_1102	*Lactobacillus delbrueckii STYM1*	Isolated as a notably abundant protein in cell extracts of strain STYM1 also observed to be bactericidal/inhibitory of *Porphyromonas gingivalis* growth	[196]	No
lpg2515	*Legionella pneumophila*	Incorrectly annotated as a RtxA homolog; designated as a known virulence factor but appears to be a novel factor influencing amoeba infiltration (via scatter screen)	[197]	No
		Mutant not observed to notably impact, either positively or negatively, the intracellular growth of *L. pneumophila* in *Drosophila* cells (both, untreated and treated with dsDNA encoding the following: *sar1*, *arf1*, *sec22*, *rab1* and *bet5*)	[198]	No
		Shares operon with *smlA* (now, *phtK* [lpg2516]), which was identified as a putative solute efflux system (MFS, major facilitator superfamily protein)	[199]	No
		Found across 27 species of Legionella; not indicated as being a virulence factor or "effector"	[200]	No
		Operon partner, lpg2516 (*phtK*/*smlA*), is upregulated by nicotinic acid, which is also shown to incite expression of virulence traits	[201]	No
LMXM_11_0130	*Leishmania mexicana*	Identified as the top enriched interactor in DUB2 (deubiquitinating cysteine peptidase 2) interactome, of which has been shown to play an essential role in establishing infecition	[202]	No
NIF3L1	*Macaca sp.*	Targeted by miRNA (mml-miR-582-5p) that is decreased in expression in striatum by simian immunodeficiency virus (SIV) infection and is restored to control levels by chronic administration of THC (9-tetrahydrocannabinol); targeting miR expression during SIV infection and chronic THC administration, alone, were nearly equivalent	[203]	No
		Mutant RNA phenotype conserved in development of monkey (Macaca fascicularis) PG-haESC genomic hybridization mutant screening model for human disease (one of several human genes used to evaluate cells)	[204]	No
		Indicated likely to have functional roles as it relates to neuronal regeneration and recovery from central nervous system lesions (fold change (LPZ/peri-LPZ) = 0.35 +/- 0.05; LPZ = lesion projection zone of primary visual cortex (V1); peri-LPZ = zone adjacent to LPZ, furthest from projection zone)	[205]	No
NIF3L1	*Meleagris gallopavo*	Located in ROH (runs of homozygosity) island 1,727,826 of one of two autocthonous groups (ROH island: where SNPs are found in ROH are found in at least 70% of birds in each group); noted as being previously linked to skin thickness in swine (*Sus scrofa domesticus*) (PMID: 24796629)	[206]	No
HcgD/ MJ0927	*Methanocaldococcus jannaschii*	Likely iron chaperone required for FeGP cofactor biosynthesis	[207]	Yes
		Homohexameric quaternary structure composed by the interfacing of 2 homotrimeric units; dinds to ssDNA/dsDNA	[208], [209]	Yes(2)
		Genomic associations of Archaeal E1-like enzymes (ThiF/MoeB)[arCOG01677]: CinA C-terminal domain homologs (arCOG04863, arCOG04865), NAD-binding domain containing proteins (arCOG04864), NIF3 homolog (arCOG04454); predicted as being associated with tRNA modification pathway (Ub1/URM1, Nsc6p, Ncs2p)	[210]	No
		Component of operon containing (HcgC) an S-adenosylmethionine (SAM)-dependent methyltransferase important for the biosynthesis of the iron guanylylpyridinol (FeGP) cofactor of [Fe]-hydrogenase	[211], [212]	No(2)
		Component of operon containing two contributors to steps crucial in the biosynthesis of the iron guanylylpyridinol (FeGP) cofactor of [Fe]-hydrogenase; HcgE adenylylates the carboxy group of guanylylpyridinol	[213]	No

		(GP), generating AMP-GP and HcgF catalyzes the transesterification of this product to produce a Cys (HcgF)-S-GP thioester		
Mmp0053	*Methanococcus maripaludis*	One of several *hcg* genes required for the function [Fe]-hydrogenase, Hmd (while *hmdII* is not); Triple mutant with *Δfrc* and *Δfru* induces least significant impact upon growth on H_2 compared to triple mutants of *Δfrc* and *Δfru* with *ΔhcgA*, *ΔhcgB*, *ΔhcgC*, *ΔhcgE*, *ΔhcgF*, *ΔhcgG*, or *Δhmd*; no differences in growth observed between mutants grown on formate	[214]	No
MTBMA_c15300/ HcgD	*Methanothermobacter marbugensis*	Described as a putative redox-state-dependent iron chaperone within the context of the Hmd and Hmd-co-occurring genes involved in the biosynthetic pathway of iron-guanylylpyridinol (FeGP) cofactor of [Fe]-hydrogenase	[215]	No
MPC_396	*Moranella endobia*	Gene has been shortened (due to either a frameshift or premature stop codon) in comparison to orthologs in free-living relatives	[216]	No
Nif3l1/ 1110030G24Rik	*Mus musculus*	Isolated, characterized; ubiquitous expression pattern across tissues; cytosolic subcellular localization; highly conserved N-, C-terminal regions; shares inserted region of its human homolog	[116]	Yes
		Retinoic acid-induced binding & cooperative translocation with Trip15/CSN2 from the cytosol to the nucleus (early neuronal development; results in gene silencing of Oct-3/4 a suppressor of cell differentiation); ubiquitously expressed across tissues, important for neuronal development	[122]	Yes
		Significantly decreased protein abundance in MPS VII mice (mucopolysaccharidosis type VII; liposomal storage disease, β-glucuronidase deficiency-driven) (fold-change = -2.81; p-value = 0.031)	[217]	No
		Listed as 1 of 5 interactors with RP1, a purported modulator of cilium length, in the description of ciliopathy disease model characterization; RP1 phenotype described as slow retinal degeneration, photoreceptor domain is listed as "Axoneme"	[218]	No
		Detected at a notably high abundance in brown fat tissue in a phosphorylated form (site = T254; spectral count = 12) with lack of tissue-enriched expression	[219]	No
		Binding partner, NIF3L1 BP1, is also ubiquitously expressed across tissues and localizes in the same manner (nuclear, cytosolic); alongside NIF3L1 BP1 is strongly overexpressed in spermatogonia-derived cells; interaction with NIF3L1 BP1 is restricted to the cytosol; expression pattern is opposite of NIF3L1 BP1, which gradually increases in germ cells, reaching maximum expression in adult mice	[220]	No
		Participant in IPA (Ingenuity Pathways Analysis) network generated for Oct-4-regulated genes (enrichment: cancer, reproductive system disease, cell death)	[221]	No
		1 of 9 candidates encoded in an eQTL (expression quantitative trait locus) "super hotspot" [chromosome 1, male mice] described as likely being responsible for the trans regulation of many genes	[222]	No
		Not linked to albinism; elevated expression with significant correlation to CCT (central corneal thickness, an ethnicity-correlated trait in humans); not considered protein of the known corneal proteome	[223]	No
		One of small fraction of proteins (5.4%) that showed significant differences between $SOCS2^{KD}$ (RNAi knockdown) & $SOCS2^{WT}$ mouse embryonic fibroblasts (MEFs) (p-value (-log10) = 2.376; log2 fold-change = 0.644)	[224]	No
		Target of IRF8 (interferon regulatory factor 8; important for B cell differentiation) significantly impacted by IRF8 knockdown (upregulated; q-value = 0.262948901; log2 fold-change = 0.370053659)	[225]	No
		Binding partner, NIF3L1 BP1, interacts with FMIP (Fms-interacting protein; both members of the THO complex, involved in mRNA processing), which localizes to the nucleus, cytoplasm; FMIP is required for the localization of NIF3L1 BP1 to the nucleus	[127]	No
		Increased expression correlated with exposure to sphingolipids produced by Bacteroides thetaiotaomicrob strain VPI 5482 (in vitro), increased [GI] loads of which strain are positively correlated with host insulin resistance	[226]	No
		Significantly downregulated in BdkrB2-/- relative to BdkrB2+/+ (p-value = 0.0489), the resulting phenotype of the former being renal dysgenesis during gestation	[227]	No

		Significantly down-regulated in murine tumors expressing human apoA-I (A-ITg$^{+/-}$; high plasma HDL-c levels) relative to apoA-I null mice (A-I KO; low plasma HDL-c levels)	[228]	No
		Differentially detected in liver tissues of 9-month old mice treated with short-chain fatty acids	[229]	No
		Significantly deregulated in combined treatment groups with ketamine and irradiation (100 mGy, 200 mGy); associated with permanent cognitive impairment in mice	[230]	No
		Downregulated in murine tissues of the kidney and aorta in EGFR knockout mice subjected to a high fat diet relative to a standard fat diet (kidney, KO-WT = -1.5; aorta, KO-WT = -3.0)	[231]	No
		Found to have altered expression in two Marinesco-Sjogren syndrome models (up in humans, down in mice): SIL1-KD HEK293 cells, as well as Woozy mouse sciatic nerve cells	[193]	No
MAV_2209	*Mycobacterium avium 104*	Operon partner, MAV_2210, selectively binds to COP9 signolosome subunit (C5N5; C5N5 is linked to protein inactivation in macrophage cytosol); MAV_2210 is described as a Mn++/Fe++ transporter	[232]	No
MAP1982c	*Mycobacterium avium subsp. paratuberculosis*	Operon partner, MAP1981c, incites immune response (confirmed immunogen), significantly increasing IgA and IgG levels after enteric infection of calves	[233], [234]	No(2)
		Operon partner, MAP1981c, induces phenotypic maturation of dendritic cells, Th1-polarization; MAP1981c is not cytotoxic but induces DC maturation via TLR4-mediated, MAPKs/NH-kB signalling pathways; evidenced link between organism, via its antigen, MA1981c, and pathology of Crohn's disease, Johne's disease	[235]	No
MLBr_01639	*Mycobacterium leprae str. Br4923*	Predicted functional annotation for 1 of 312 hypothetical protein-coding genes	[236]	No
MMAR_3306	*Mycobacterium marinum*	Operon partner, mmar_3307, is linked to *iniBAC* induction through its role in cobalamin biosynthesis; *iniBAC* is an operon that is robustly upregulated during treatment with ethambutol, isoniazid	[237]	No
MSMEG_4307	*Mycobacterium smegmatis*	This homolog shows a conserved signature indel (CSI) of two amino acids that is present in all *Mycobacterium* species except in members of the "*Abscessus-Chelonae*" clade	[238]	No
		Expression, including that of operon members MSMEG_4306 and MSMEG_4305, is driven by an essential RNase H1 enzyme encoded by *rnhC* via native promoter; a subset of *Mycobacterium* encode only RnhC as sole RNase H1 (*M. smegmatis* & 34+ other taxa); RnhA-minus group incl. human-/animal-pathogenic species	[239]	No
		Described as a member of histidine kinases; present in all Mycobacterium; 64% identity with *Nocardia farcinica* (NFA16340); no listed *Streptomyces* (SCO/SAV) homolog; several listed *Corynebacteria*: CG2457 (49%), DIP1680 (53%), JK0667 (51%), CE2135 (50%)	[240]	No
		Determined to be co-expressed with neighbors (operon members) MSMEG_4306 and MSMEG_4305 (RNA-seq, RT-PCR)	[241]	No
		Operon neighbor and expression partner, MSMEG_4306, is validated in DNA-binding capabilities (DNA-association evidenced through several assays)	[242]	No
		Expression partner, MSMEG_4306, is a structural, sequence homolog of *C. trachomatis* (CT398), *H. pylori* (HP0958); by remarkable homologies, MSMEG_4306 predicted to be component of a type III secretion system	[243]	No
		Expression partner, MSMEG_4306, demonstrated substantial but not* significant, positive fold-change response to VapC conditional expression in *ΔvapBC* mutants (*vapBC* operons encode the largest family of toxin-antitoxin modules; *vapBC* negatively regulates glycerol utilization pathways, VapC targets cohort of genes involved in sugar metabolism, transport)	[244]	No
		Operon partner, MSMEG_4305, is essential for the survival of *M. smegmatis*	[245]	No
Rv2230c	*Mycobacterium tuberculosis*	Significantly downregulated in *ΔsigM* mutant (fold-change = 2.7; p-value =0.014); SigM linked to host-pathogen interaction (late-stage infection)	[246]	No
		Operon partner, Rv2228c, is proposed to primarily function in phosphate generation (environment of obligate intracellular pathogen is phosphate-	[247], [248]	No(2)

		poor); fusion of *cobC* and RnhA (RNase HI), increased activity relative to RNase H homologs		
		Operon partner, Rv2228c (RnhA-CobC fusion), is unique to mycobacterial replisome/repair (not present in E. coli)	[249]	No
		Significantly decreased protein abundance (fold-change = -1.018063891 (log10, NM vs. WT); p-value = 0.010833) in clinically isolated natural mutant (15kb genomic deletion = 12 genes; decreased virulence)	[250]	No
		No significant differential expression observed with mefloquine treatment	[251]	No
		Operon partner, Rv2229c, observed to act as an antigenic peptide by γδ T cells	[252]	No
		Operon partner, Rv2231c (*cobC*), is indicated as being under purifying selection (3/3 codons under selection; GWAS); purifying selection of cobalamin-related genes indicated adaptive value in clinical settings (AB resistance), although non-essential to organism survival	[253]	No
		Operon partner, Rv2231c (*cobC*), indicated as being conditionally essential for growth in C57BL/6 Mice	[254]	No
		Operon partner, Rv2231c (*cobC*), predicted to have L-threonine O-3-phosphate as its essential binding partner	[255]	No
		Operon partner, Rv2231c (*cobC*), determined to contribute to amino acid metabolism by the conversion of glutamate to histidinol-phosphate via improvement of *in silico* model using empirical data reflecting *in vivo* physiology	[256]	No
JCVSYN2_01085/ CDS_60	*Mycoplasma mycoides JCVI-Syn3B*	Encoded in minimal genome of persister cell population (ciproflaxacin-, streptomycin-treated), overlaps with *trmK*/*yqfN* (JCVSYN2_01080)	[257]	No
NGO2028	*Neisseria gonorrhoeae*	Upregulated (5.28 fold-change) in *ΔnrrF* mutant relative to *ΔnrrF* mutant with complement (p-value = 0.0715)	[258]	No
NMB2054	*Neisseria meningitidis*	Encoded immediately divergent of MetR-like transcription factor linked to methionine metabolism and oxidative stress response	[259], [260]	No(2)
NCU06108	*Neurospora crassa*	transcriptional regulator indicated as being under clock control; 1 of 295 clock-controlled genes of unknown function	[261]	No
CI960_11205	*Parabacteroides sp. CT06*	Detected within a widely disseminated integrative and conjugative element demonstrated to promote *cfxA* (beta-lactam resistance gene) dissemination in *Bacteroides*, *Parabacteroides*	[262]	No
NIF3L1	*Phoca largha*	Protein found to be one of 26 notably upregulated in the blood of wild pups relative to that of captive pups; was found to interact with HSP90AB1, a protein noted to have the highest number of predicted interactions in a generated protein-protein interaction network	[263]	No
Plu1424	*Photorhabdus luminescens*	Identified as component of extracellular proteome of stationary phase	[264]	No
		(Relative to YbgI of E. coli) Protein divergence = 0.446 (Reciprocal Sequence Distance method of pairwise homolog divergence determination)	[265]	No
PG2043	*Porphyromonas gingivalis*	Notably upregulated (fold-change = 3.7208481) after 10-minute exposure to 0.25 mM hydrogen peroxide in *ΔvimA* mutant; was not detected to be upregulated after 15-minute exposure to 0.25 mM hydrogen peroxide	[266]	No
		Significantly downregulated in ΔPG0228 mutant (ratio (635/532nm) = 2.51) (PG0228: PF02274, Amidinotransferase)	[267]	No
		One of top 20 upregulated genes (fold-change = 1.384237; p-value = 0.392613) with nitrite (200 μM) treatment; differential no longer notable in *Δhcp* mutant (hcp was superlative among those upregulated in WT)	[268]	No
PA3762	*Pseudomonas aeruginosa PAO1*	Differentially expressed in biofilms treated with cis-2-deconoic acid (CDA) (+1.57-fold change); treatment causes dispersion response in microbial biofilms	[269]	No
		Differentially expressed in an elevated c-di-GMP background (-2.4-fold change)	[270]	No
		Left without dysregulation with treatment with peptide 1018 (treatment that inhibits biofilms formation, swarming motility of bacterial); notable differential regulation was observed in actively swarming cells relative to those at the swarm edge (fold-change = -2.0)	[271]	No
		Significantly upregulated in *ΔphoQ* mutant (fold-change = +1.6; p-value = 0.01); *ΔphoQ* mutant is polymyxin B hyper-resistant	[272]	No
WP_046236688 WP_032702676	*Pseudomonas sp.*	YqfO, or "YqfO03", is a small secreted protein; demonstrates high potency as a nematocidal against *C. elegans*, *M. incognita*. Assert that free-	[273]	Yes

PP_1038<br>VT47_06255<br>WP_017124074<br>WP_054077596		standing YqfO domain-containing protein not containing any NIF3 domains is a member of the NIF3 protein family		
		Referenced, described in the context of previous work done investigating nematocidal properties of the virulence-associated protein (*P. syringae*)	[274]	No
PSTAB_1087	*Pseudomonas stutzeri ATCC 17588*	Enzymatic conserved domain predicted for hypothetical protein identified in genome; structure predicted using best-score template (2GX8; bit score=357.6; e-value= 8.60E-14; identity(%)=38.24; aligned(%)=97.14)	[275]	No
Nif3l1	*Rattus norvegicus*	Contributor to rat-specific cholestasis gene signature (1 of top 100 genes); only male rats were used*	[276]	No
		In cocaine-trained rats subjected to 30 days of forced withdrawal, was observed to be hypomethylated (sample source: nucleus accumbens) in those subjected to extinction test examining cue-induced cocaine-seeking behaviors without reinforcement; fell within "cancer" (hypermethylated; p-value = 1.34E-13) and "cell death" (hypermethylated; p-value = 4.10E-05) categories of enrichment; only male rats used*	[277]	No
		mRNA indicated to interact with lincRNA ENSRNOT00000076905, which interacted with several other mRNAs associated with neurogenesis and neuron development	[278]	No
OG0002099	*Reticulitermes flavipes*	Did not demonstrate caste-biased gene expression, while exhibiting queen-biased expression in *Zootermopsis nevandensis*, *Cryptotermes secundus*, and *Macrotermes natalensis*	[279]	No
Z518_09833	*Rhinocladiella mackenziei*	Determined to be a member of single-copy orthologs (SCOs) shared between genomes of *Rhinocladiella*; observed RTH (read-through, SNP) suppression through mutation of stop codons (8%)	[280]	No
Nif3/<br>YGL221C	*Saccharomyces cerevisiae*	1 of 5 proteins determined to interact with NGG1	[281]	No
		NMR crystal structure is of lower quality (grade C); quality rationalized to be most likely caused by aggregation or oligomerization of the protein	[282]	No
		Mutant: 2.5-fold decrease in sporulation efficiency implicating NIF3 in meiosis; coexpression metagene pair w/ RIM11 and RIM15 (4 networks); coexpression metagene pair w/ CGI121, a component of the EKC/KEOPS complex (2 networks)	[283]	No
		Mitochondrial subcellular localization	[284]	No
		Protein abundance: 6.00E+03 of a avg. total of 4.67E+07 protein molecules per cell (total cell extract) (~0.013%)	[285]	No
		Promotes Ty1 transposition (synthetic lethal esp1-1 screen); mutant suppresses hypertransposition phenotype of *rtt101Δ* and *med1Δ* [286]	[287], [288]	No
		During co-culture with *Lachancea thermotolerans* during alcoholic fermentation, NIF3 was observed to be significantly downregulated during T1 (early death phase) relative to phase control (fold-change = 0.757858; p-value = 0.010471)	[289]	No
		Predicted by RCDP (Relative Co-evolution of Domain Pairs) to interact with YNL189w/SRP1, a karyopherin alpha homolog, forms a dimer with karyopherin beta Kap95p to mediate import of nuclear proteins	[290]	No
		Indicated to possibly play a role in recruitment of San1 for the degradation of various toxic abnormal proteins	[291]	No
		Mutant failed to exhibit multinucleate cell body phenotype	[292]	No
		Protein shown to interact with PBP1 which interacts with MUD1 (U1 snRNP A protein, a protein involved in nuclear mRNA splicing)	[293]	No
		Determined to have dual/multiple subcellular localization (cytosolic and mitochondrial)	[294], [295]	No/Yes
		Mitochondrial protein localization did not change with treatment of cells with mitochondrial inner membrane depolarizing agent, trifluoromethoxy carbonyl cyanide phenylhydrazone (FCCP)	[296]	No
comp3044_seq3	*Schistosoma mekongi*	Significantly upregulated in males relative to females in adult worms (12 Log2-fold change; p-value = 1.01E-20; FDR = 4.89E-19)	[297]	No
SPCC126.12	*Schizosaccharomyces pombe*	Mutant: UV sensitivity = "medium"	[90]	No
		Mutant: no change in cell shape, morphology	[298]	No
		Mutant: normal count of mating projections but projections were observed to be abnormally long and straight (highly penetrant; grade = 9 out of 10)	[299]	No
		Mutant: showed increased sensitivity to MMS (methyl methanesulfonate)	[300]	No

CGQ25_08800	*Sinomonas sp. R1AF57*	Shown encoded within the neighborhood of a DUF328 homolog & PF02591, the latter of which is within the same operon	[301]	No
A0A0E3ZV03/ SD10_07820	*Spirosoma radiotolerans DG5A*	Identified as an uncharacterized protein conserved across organisms of *Bacteroidetes* (excluding class *Bacteroidia*); one of two unknown pairs of Pfam annotations consistently encoded next to each other (PF02591 [zf-RING_7] & PF01784 [NIF3]; PF01327 [Pep_deformylase] & PF03652 [RuvX] were an additional, unrelated pair)	[302]	No
SA1388	*Staphylococcus aureus*	Central domain of NIF3 homolog has high structural similarity to CutA1 proteins (family is linked to cation tolerance)	[303], [304]	Yes/No
		Instigated no immunologic response in mouse model (screening of multimerizing proteins of pathogens for vaccine scaffold candidacy)	[305]	No
		P_{II}-like domain returned as structurally similar match to SPOR domain of DamX (SPOR domains bind septal peptidoglycan; linked to bile salt resistance, improved efficiency of cell division [306])	[307]	No
		Down-regulated in acid-shock conditions (4.9-fold reduction)	[308]	No
		Determined by transcription pattern profiling to be upregulated by SarA, an established regulator of virulence genes	[309]	No
		Structure contains sequence repeats of 25% identity, 60 residues in length	[310]	No
		Found in isolated specialized mobile genetic element associated with drug resistance	[311]	No
		Predicted to have a high probability of essentiality	[312]	No
		Significantly differentially expressed in VISA strain CMRSA-2 relative to VISA strain Mu50 (log2 ratio = 2.9; p-value = 1.7E-6)	[313]	No
		One of several genes described to have acquired SNPs relative to their parent genomes, ultimately not linked to emergent copper tolerance observed in mutant strain of MRSA; mutant mRNA expression levels consistent with parent samples	[314]	No
SP1609	*Streptococcus pneumoniae*	Virulence factor	[315], [316]	No(2)
		Mutant: slight growth defect	[317]	No
		Significantly upregulated in lung infection relative to nasopharynx (mouse)	[318]	No
		Operon partner, SP1610, characterized as a tRNA methyltransferase; resolved crystal structure of SP1610 shown in complex with S-adenosyl-l-methionine	[319]	No
		Described as a member of the same orthologous group (COG2384) as members of the TrmK and RpoD protein families in structural alignment (incorrect)	[320]	Yes(No)
Spy_0931/ M5005_Spy0732	*Streptococcus pyogenes serotype M1*	indicated as being cell wall-attached as an antigen contributing to host plasma/saliva interactome; interacts greatest with IgG1 relative to other host proteins	[321]	No
		Observed as a significant contributor to the microvesicle proteome while being absent from the membrane proteome	[322]	No
		Detected in the extracellular proteome; greatest levels of detection occurred during the late stationary and biofilm phases of growth	[323]	No
SCO2301/ SCC30.09c	*Streptomyces coelicolor*	Determined to be a top-30 priority candidate orphan gene of *Streptomyces coelicolor* (orphanicity rank = 4; final rank = 6); phosphate starvation did not produce significant trends in gene expression (24 hr = duration of P-depleted conditions starting after 36 hr of cultivation, expression)	[324]	No
"Orf11"	*Streptomyces sp. SN-1061M*	Homolog located immediately adjacent to LPM (liposidomycin) biosynthetic gene cluster; highly similar to SCO2301 of *S. coelicolor* (absent in strains that do not produce LPMs)	[325]	No
STCUT_1350	*Sulfobacillus thermosulfidooxidans*	Noted in list of genes with lower codon information bias (CIB) in consortium of acidophilic organisms demonstrating increased copper resistance (CIB = 0.158)	[326]	No
YqfO	*Tenacibaculum sp.*	Used in the classification of *Tenacibaculum* species due to regions of variability within members of the genus, described as a housekeeping gene	[327]	No
		Described as one of several genes used to classify Tenacibaculum species	[328]	No
YqfO	*Tenacibaculum maritimum*	Described as one of 11 housekeeping genes utilized in multi-locus sequence typing of isolates	[329]	No
		Listed as one of several housekeeping genes used in the multi-locus sequence typing of isolates	[330]	No

Theam_0231	*Thermovibrio ammonificans*	Identified in the proteome of *T. ammonificans* grown under nitrate reducing conditions (NSAF [normalized spectral abundance factors NSAF] = 0.04%)	[331]	No
TTHA1606	*Thermus thermophilus HB8*	Binds to ssDNA (very weakly; *in vitro*)<br>Predicted to be regulated by SbtR (TetR family regulator)	[332]<br>[333]	Yes<br>No
TP_0977/ TPASS_0977	*Treponema pallidum subsp. pallidum SS14*	Listed as 1 of 207 proteins assigned functional annotation with high confidence; list also includes gene neighbor, TPASS_0979, which was designated as a TatD related DNase	[334]	No
TWT257	*Tropheryma whipplei str. Twist*	Significantly upregulated when subjected to temperature differential of 4°C from 37°C (1.5 fold-change)	[335]	No
ObacDRAFT_2308	*Verrucomicrobium sp. strain TAV2*	Significantly upregulated in expression in 2% oxygen growth conditions (1 of 23 genes)	[336]	No
VC2093/ VC_2093	*Vibrio cholerae*	Increased gene expression in the presence of soluble chitin oligosaccharides $(GlcNAc)_{2-6}$, GlcNAc, or the glucosamine dimer $(GlcN)_2$ as alternative carbohydrate sources relative to an absence of carbohydrates (average = 1.18); differential expression in *ΔchiS* mutant relative to WT in presence of $GlcNAc_2$ = 0.68<br>Mutant showed decreased colonization of rabbit intestine (fold-change [log2] = 0.34; p-value [log10] = 0.0269; output-input ratio = 2.9597); mutant of gene neighbor, VC2094, exhibited decrease in colonization of rabbit intestine (fold-change ratio = 1.57; p-value = 0.00139)<br>Significantly decreased expression in *ΔdegS* (log2 fold-change = -0.939; FDR = 0.0000397)<br>Greatest transcript reads observed in pandemic El Tor strains isolated in Bangladesh (N16961, 1971, RPKM = 25.4; MDC126, 2008, RPKM = 27.9) relative to other strains isolated in Haiti (H1, 2010, RPKM = 15.3) and Peru (C6706, 1991, RPKM = 18.7)	[337]<br>[338]<br>[339]<br>[340]	No<br>No<br>No<br>No
VF_0817	*Vibrio fischeri*	Expressed in isolates grown in chitin-supplemented seawater (was not observed in isolates grown in the light organs of field-caught hosts); may be involved in initial colonization processes<br>Encoded immediately downstream of phosphoglucomutase (VF_0816), a gene characterized as a promoting factor for the symbiotic colonization of the bacterium's host organism, *Euprymna scolopes*; 74% identity, 81% similarity to *V. cholerae* homolog; 62% identity, 78% similarity to YbgI of *H. influenzae*	[341]<br>[342]	No<br>No
WS2117	*Wolinella succinogenes*	Fused with gene encoding DUF164-containing protein (DUF164 is a relatively uncharacterized zinc finger-like domain) in operon with *glyQ* (glycyl-tRNA synthetase, alpha subunit), *kdtA* (3-deoxy-D-manno-octulo-sonic-acid transferase), and *rluD* (pseu- douridine synthase), a common collective in sub-groups of Proteobacteria	[44], [115]	No(2)
NIF3L1	*Xenopus allofraseri*	Two isoforms up-regulated with both endurant- and burst-performant individuals relative to non-performant wildtype; evidence implicated isoforms to interact with *arpc4* (actin-binding component of the Arp2/3 complex; up-regulated in burst-performance individuals) and *abi2* (component of the WAVE complex; up-regulated in endurant individuals)	[343]	No
NIF3L1	*Xenopus sp.*	Binding partner, NIF3L1 BP1, interacts with FMIP (Fms-interacting protein; both members of the THO complex, involved in mRNA processing), which localizes to the nucleus, cytoplasm; FMIP is required for the localization of NIF3L1 BP1 to the nucleus	[127]	No
nif3l1	*Xiphophorus maculatus*	Uniquely downregulated in skin of males relative to that of females when adults exposed to 4100K fluorescent light (z-score = 2.4; cell proliferation; category, 71 of 372 total uniquely DEGs, males); neg. corr. with up-reg. of genes related to chromosome, DNA damage; pos. corr. with down-reg. of genes related to DNA repair, cell cycle checkpoint control, recombination, and chromosome segregation	[344]	No
NIF3-like protein superfamily	*NA*	(translated electronically from Chinese for review of contents; Google Docs Translate Document Tool) describes homologs of model organisms (Eukaryota, Bacteria), structures published prior to 2007	[345]	Yes
NIF3L1	*NA*	Not translatable; functional association uncertain; NIF3L1 (formal, human homolog gene/protein name) is mentioned more than once within body of text	[346]	No

YbgI	*NA; gram positive, gram negative bacteria*	One of 421 differentially expressed universal stress responders also involved in basic biological processes (on the low-end of the above-threshold range of determined metric)	[347]	No

Two publications requiring electronic translation to English (also missing DOI identifiers relevant to US publishers) were included in the above table. Only one of these were considered "focal" and, therefore, able to be included in the within-text table of focal publications (Table 2). These publications were not used in any analyses involving the results of the literature review/data capture process (e.g., final metrics regarding data capture yields, distributions of relevant publications across superkingdoms, word clouds generated from publication titles).

Table S4. Metal ion interactions of proteins encoded by representative operons.

	Biological Category	COG	Protein	Metal(s)	References (PMID, BRENDA, EC)
**Bacteria**					
**Metal-binding/ -dependent**	DNA and RNA metabolism	COG0328	RnhA	Mg^{2+}, Mn^{2+}, Co^{2+}, Ni^{2+}	16601679, [3.1.26.4]
		na	RNase P	Mg^{2+}, Mn^{2+}, Zn^{2+}	15867194, [3.1.26.5]
		COG0125	Tmk	Mg^{2+}	28627020, [2.7.4.9]
		COG0752	GlyQ/S	Mg^{2+}, Mn^{2+}, Co^{2+}	4295604, [6.1.1.14]
		COG3935	DnaD	Mg^{2+}	18703019
		COG0358	DnaG	Zn^{2+}, Mg^{2+}, Mn^{2+}	1511009, [2.7.7.101]
		COG0568	RpoD/SigA	Zn^{2+}, Mg^{2+}	29514271, [2.7.7.6]
		COG3481	YhaM	Mn^{2+}, Co^{2+}	12399495, 9868367
		COG0319	YbeY	Zn^{2+}, Ni^{2+}	15632286, 16511207
		COG1381	RecO	Zn^{2+}	25170075, 15719017
		COG0228	RpsP	Mg^{2+}, Mn^{2+}	8730873
		na	Initiator $tRNA^{Met}$	Mg^{2+}	4563246
	A proportion of cases ►	COG0336	TrmD	Mg^{2+}, Mn^{2+}, Ca^{2+}	25219964, [2.1.1.228]
		COG0266	Fpg/MutM/Nei	Zn^{2+}, Fe^{2+}, Mg^{2+}, Mn^{2+}, Ca^{2+}, Cu^{2+}/Ni^{2+}*	7955043, [4.2.99.18]
		COG0232	Dgt	Mg^{2+}, Mn^{2+}	25694425, [3.1.5.1]
	Membrane biosynthesis; transport; signalling	COG1137	LptB	Mg^{2+}	19234479, 11080142, [3.6.3.-]
		COG1496	YfiH/RL5/PgeF	Zn^{2+}, Cu^{2+}, Fe^{2+}, Co^{2+}, Ni^{2+}, Cd^{2+}	16498617, 16740638, 28612943, [1.7.2.1, 1.10.3.3, 1.10.3.2, 1.16.3.1]
		COG0541	Ffh	Mg^{2+}	14696184, [3.6.5.4]
		COG1127	MlaF/ttg2A	Mg^{2+} (ATP-Mg^{2+})	25916755
	Regulation	COG1692	YmdB	Fe^{2+}, Fe^{3+}, Mg^{2+}, Mn^{2+}, Ca^{2+}	24163345, [3.1.4.16]
		COG0265	DegQ	Zn^{2+}, heme, Mn^{2+}, Ca^{2+}, Fe^{2+}, Mg^{2+}	23695557, 23176475, 18723647 [3.4.21.107]
		COG3744	VapC16	Mg^{2+}, Mn^{2+}	28575517
		COG0645	Zeta toxin/P-loop containing NTPase	Mg^{2+}	21445328
		COG1366	SpoIIAA	Mg^{2+}	15236958
		COG0466	Lon	Mg^{2+}, Mn^{2+}, Ca^{2+}	16511355, [3.4.21.53]
		COG1579	DUF164, zinc ribbon containing	Zn^{2+}	22408721
		COG0642	BaeS/NtrC/AtoS	Fe/Fe^{2+}/Fe^{3+}, Cu^{+}/Cu^{2+}, Ag^{+}, Mg^{2+}	26950881, 21886814, [2.7.13.3]
		COG2204	AtoC-REC/NtrC (PilR-like)	Mg^{2+}, Mn^{2+}, Sr^{2+}, Zn^{2+}/Pb^{2+}, Ag+, Fe/Fe^{2+}/Fe^{3+}, Cu^{+}/Cu^{2+}	REC domain: cd00156; CheY-like receiver: 8257674; Other: [2.7.13.3], 11243806 (BRITE/KEGG)

		na	VanSB-like (MGA_0021)	Ag^{+}, Cu^{+}/Cu^{2+}, $Fe/Fe^{2+}/Fe^{3+}$, Mg^{2+}	[2.7.13.3]**
	Small molecule metabolism	COG0346	GloA	Ni^{2+}, Co^{2+}, Cd^{2+}, Mn^{2+}, Mg^{2+}, Zn^{2+}, Ca^{2+}, Fe^{2+}	21820381, VOC-like domain: cd07245; [3.4.17.13]
		COG0041	PurE	Mg^{2+}	2464576, [5.4.99.18]
	fusion ►	COG0328-COG0406	RnhA-CobC	Mg^{2+}	6312898
	fusion ►	COG1211-COG0245	IspD-IspF	Mn^{2+}, Mg^{2+}	21543842, [2.7.7.60], 10694574, [4.6.1.12]
		COG0699	CrfC	Zn^{2+}, Mg^{2+}	[3.6.5.5]
		COG2049, COG1984, COG1540	PxpB, PxpC, PxpA	Mg^{2+}, Mn^{2+}	28830929, [3.5.2.9]
**Heme/metalloenzyme/ metallocofactor biosynthesis, transport** (Non-metal-dependent indicated by asterisk)		COG2274	SunT	Mg^{2+}	26201595 (cd02424: Peptidase_C39E)
	Cytochrome c maturation ►	COG1131, COG1277	CcmA/GldA (CcmABC), GldF/NosY*	Heme (Cu) (GldAFG)	28472044, 11948149; P-loop: cl38936; COG1277 [CDD]; 12618453
		COG0760	SurA	Mg^{2+}	12429090, [5.2.1.8]
		COG0778	RdxA/NfnB-like	Fe/Fe^{2+}	[1.13.11.55]**
	Homogentisic acid, pyomelanin production/transport ►	COG1127	MlaF/ttg2A, MlaB (MlaBCDEF/ Ttg2ABCDE)	Fe^{3+}, Mg^{2+} (ATP-Mg^{2+}); Fe^{2+}(assoc. enzymes)	20870774, 23858455, [3.6.3.-]; SulP antagonist domain: cd07042; 15236958
		COG0079	CobC*	Co (cobalamin)	32973726, [2.6.1.9]
**Fe-S cluster**	Base excision repair; DNA repair, maintenance	COG1194	MutY	$[4Fe\text{-}4S]^{2+}$	25445713
		COG1533	SplB	$[2Fe\text{-}2S]^{2+}$, $[4Fe\text{-}4S]^{2+}$	16829676
		COG0415	PhrB	[4Fe-4S]	23589886
	Fe-S cluster biogenesis	COG0694	NfuA/NifU	[4Fe-4S]	22966982
	Regulation	*na*	ENOG5030DA8 (BC_4767)	Fe/Fe^{2+}, Fe-S, [2Fe-2S], [4Fe-4S], Mg^{2+}, Ca^{2+}	[7.1.1.2]**
		COG0826	PrtC/UbiU/YhbU	Fe-S cluster/Fe, Ca^{2+}	31289180, 1317840
	Regulation of virulence, membrane traits and interactions with environment	COG5007	BolA	[2Fe-2S]	27951647
**Archaea**					
**Metal-binding/ -dependent**	DNA and RNA metabolism	COG2016	Tma20	Zn^{2+}, Mg^{2+}	12054814
		COG0024	Map	Co^{2+}, Fe^{2+}, Mg^{2+}, Mn^{2+}, Ni^{2+}, Zn^{2+}	9811545, [3.4.11.18]
		COG1833	Uri	Zn^{2+}	16646971
		COG5431	Shu2-like, SWIM domain containing	Zn^{2+}	29069504
		COG0640	ArsR	Zn^{2+}, Co^{2+}, Ni^{2+}	10995250, 9466913; HTH ArsR: cd00090
		COG0013	AlaS	Zn^{2+}, Mg^{2+}	16374837, [6.1.1.7]
	Small molecule metabolism	COG0388	YafV (YafV-AguA fusion)	Zn^{2+}, Ni^{2+}, Mg^{2+}, Co^{2+}, Fe	pfam00795 [3.5.5.1, 3.5.1.4, 3.5.1.12, 3.5.1.6]
	Metal chelation, homeostasis	COG0679	RhaT	Se	SelP domain: cl04615
	Cofactor biosynthesis, maturation	COG4074	Mth/Hmd	Zn^{2+}	8599536, [1.12.98.2]
		COG4015	HcgE	Mg^{2+}	25882909
		COG0502	HcgA/BioB	$[4Fe\text{-}4S]^{1+}$, $[2Fe\text{-}2S]^{1+}$, Fe/Fe^{2+}	22095926, [2.8.1.6]
		COG4018	FlpA/HmdC/HcgG	[4Fe-4S]	22095926

Uncharacterized	*Na*	Mhun_0036	Zn^{2+}, Mg^{2+}	18433773
	na	Mhun_0038	Zn^{2+}	PF04434: SWIM zinc finger domain

***Indicates EC number acquisition via KEGG GFIT (≥30% identity); *indicates interaction with metals is indirect*

Table S5. Essentiality data of DUF34 homologs.

Organism	Gene/ORF	Essentiality	Source
Acinetobacter baumannii ATCC 17978	A1S_2494	NE	Ogee
Acinetobacter sp. ADP1	ACIAD1347	NE	Ogee
Bacillus cereus ATCC 14579	YqfO/BC_4286	NE	DEG
Bacillus subtilis subsp. subtilis str. 168	BSU25170	NE	Ogee
Burkholderia cenocepacia J2315	BCAL0327	NE	Ogee
Drosophila melanogaster	FBgn0014092	NE	Ogee
Escherichia coli K-12 MG1655	YbgI/b0710	NE	DEG, Ogee
Escherichia coli O23b:H4-ST131	EC958_0819	NE	Ogee
Francisella tularensis subsp. novicida U112	FTN_1077	NE	Ogee
Haemophilus influenzae Rd KW20	HI0105	NE	Ogee
Helicobacter pylori ATCC 700392 / 26695	HP0959	E*	DEG, Ogee
Homo sapiens	NIF3L1	C*	DEG, Ogee
Methanocaldococcus jannaschii DSM 2661	MJ0927	NE	DEG
Mycobacterium tuberculosis H37Rv	Rv2230c	NE	Ogee
Mycoplasma agalactiae PG2	PE_MAG0990	E**	pDEG
Mycoplasma pulmonis UAB CTIP	MYPU_4560	NE	Ogee
Neisseria gonorrhoeae MS11	NGFG_01855	NE	Ogee
Pseudomonas aeruginosa PAO1	PA4445	NE	Ogee
Pseudomonas aeruginosa UCBPP-PA14	PA14_57740	NE	Ogee
Saccharomyces cerevisiae	NIF3	NE	Ogee
Salmonella enterica subsp. enterica serovar Typhi str. CT18	STY0751	NE	Ogee
Salmonella enterica subsp. enterica serovar Typhimurium str. SL1344	SL1344_0692	NE	Ogee
Schizosaccharomyces pombe	SPCC126.12	NE	Ogee
Shewanella oneidensis MR-1	SO_2621	NE	Ogee
Staphylococcus aureus subsp. aureus NCTC 8325	SAOUHSC_01660	NE	Ogee
Vibrio cholerae O1 str. C6706	VC_2093	NE	Ogee
Yersinia pestis KIM	y1272	NE	Ogee

C = "conditional", E = "essential", NE = "non-essential"
** denote that a single study directly related to the consensus determined for essentiality may not indicate evidence of essentiality with clarity or specificity*
*** indicates singular entry from pDEG, which contains predicted essential genes of sequenced Mycoplasma species; this particular gene was indicated to be an unique fusion with other genes that may be alternative sources of implicated essentiality*

3. Supplemental Figures

Figure S1. Word cloud of focal and non-focal publication titles. **(a)** Word cloud generated MonkeyLearn.com (Theme= Rounded; Font= Quicksand; Word Quantity= 100. **(b)** Word cloud generated using TagCrowd.com (Maximum number of words to show= 100; Minimum frequency= 1; Show frequencies= yes; Group similar words= yes; Convert to lowercase= lowercase; Don't show these words= none listed).

a.

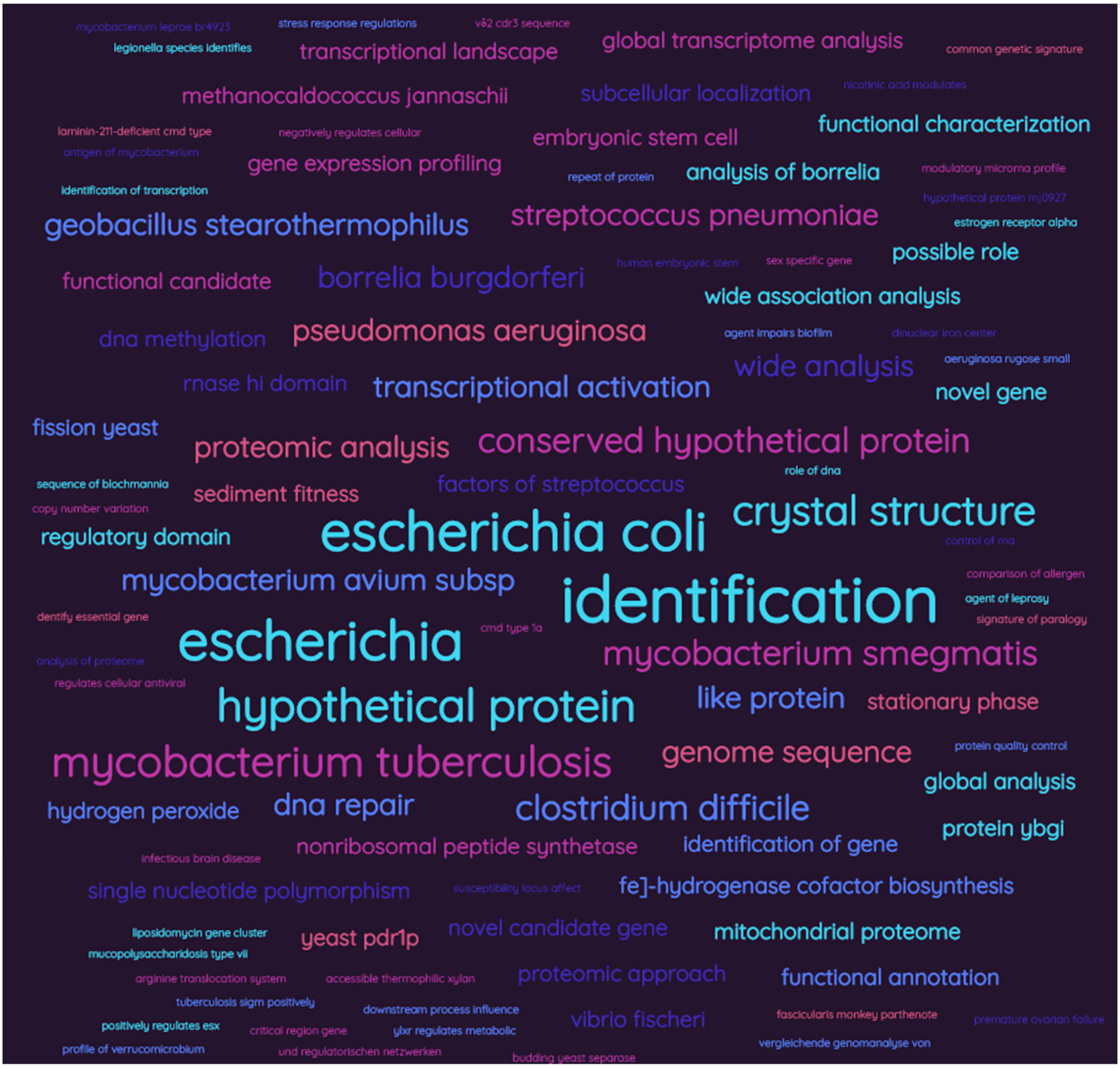

b.

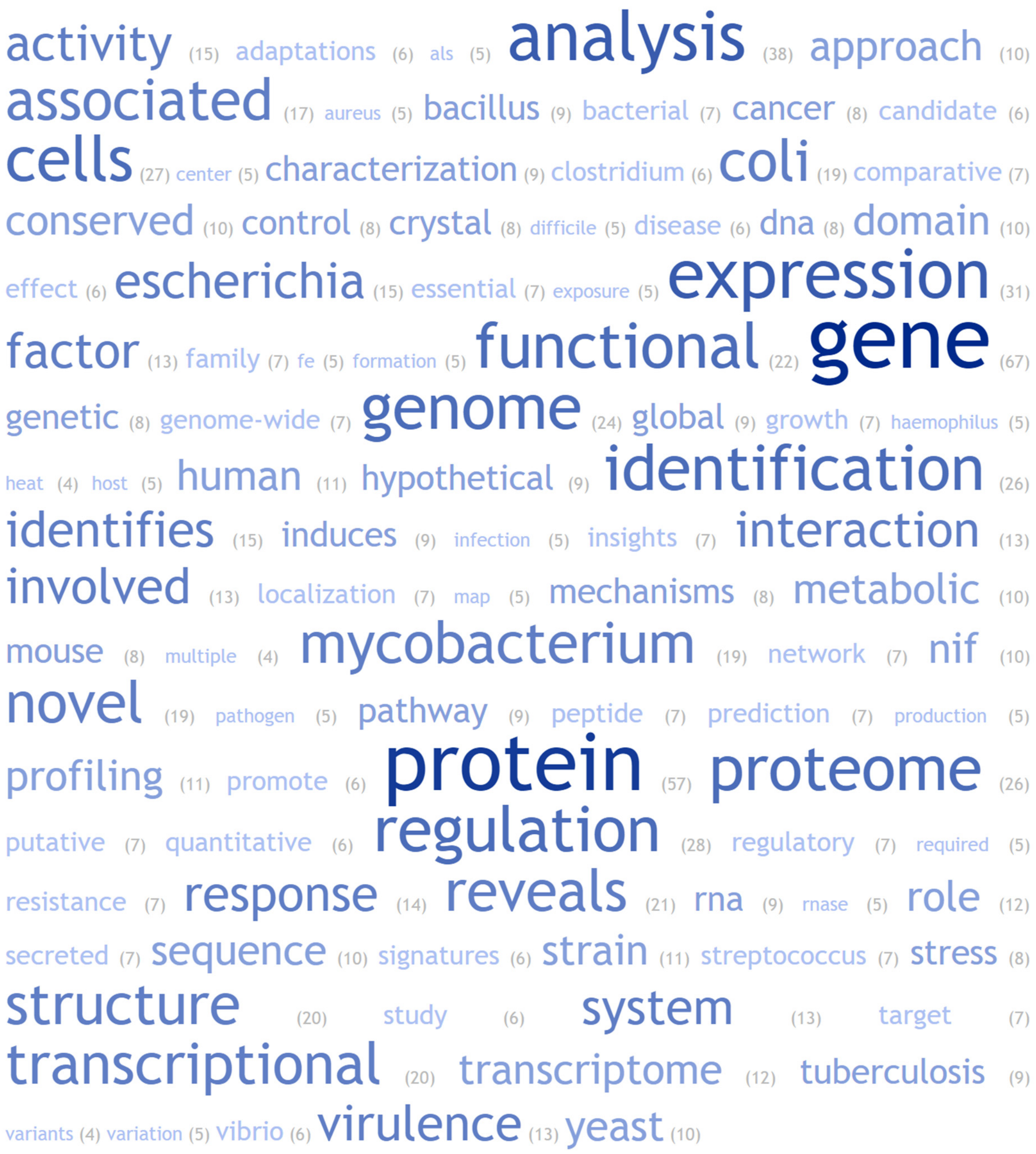
activity (15) adaptations (6) als (5) analysis (38) approach (10)
associated (17) aureus (5) bacillus (9) bacterial (7) cancer (8) candidate (6)
cells (27) center (5) characterization (9) clostridium (6) coli (19) comparative (7)
conserved (10) control (8) crystal (8) difficile (5) disease (6) dna (8) domain (10)
effect (6) escherichia (15) essential (7) exposure (5) expression (31)
factor (13) family (7) fe (5) formation (5) functional (22) gene (67)
genetic (8) genome-wide (7) genome (24) global (9) growth (7) haemophilus (5)
heat (4) host (5) human (11) hypothetical (9) identification (26)
identifies (15) induces (9) infection (5) insights (7) interaction (13)
involved (13) localization (7) map (5) mechanisms (8) metabolic (10)
mouse (8) multiple (4) mycobacterium (19) network (7) nif (10)
novel (19) pathogen (5) pathway (9) peptide (7) prediction (7) production (5)
profiling (11) promote (6) protein (57) proteome (26)
putative (7) quantitative (6) regulation (28) regulatory (7) required (5)
resistance (7) response (14) reveals (21) rna (9) rnase (5) role (12)
secreted (7) sequence (10) signatures (6) strain (11) streptococcus (7) stress (8)
structure (20) study (6) system (13) target (7)
transcriptional (20) transcriptome (12) tuberculosis (9)
variants (4) variation (5) vibrio (6) virulence (13) yeast (10)

Figure S2. Secondary structural annotation by superkingdom using MultAlign and ESPRIPT. Sequences used were those listed in Table S2 per superkingdom and per their sequential and domain architectural similarity. Crystal structures used for the structural analyses for Archaea, Bacteria and Eukaryota were as follows, respectively: **(a)** 3WSD (MJ0927/HcgD, *Methanocaldococcus jannaschii*), **(b)** 1NMO (YbgI, *Escherichia coli*), and **(c)** NIF3L1 (model structure produced using Phyre2; input template structure for Phyre2 model was 2GX8 (YqfO, *Bacillus cereus*), chosen because it also contains an inserted domain region).

a.

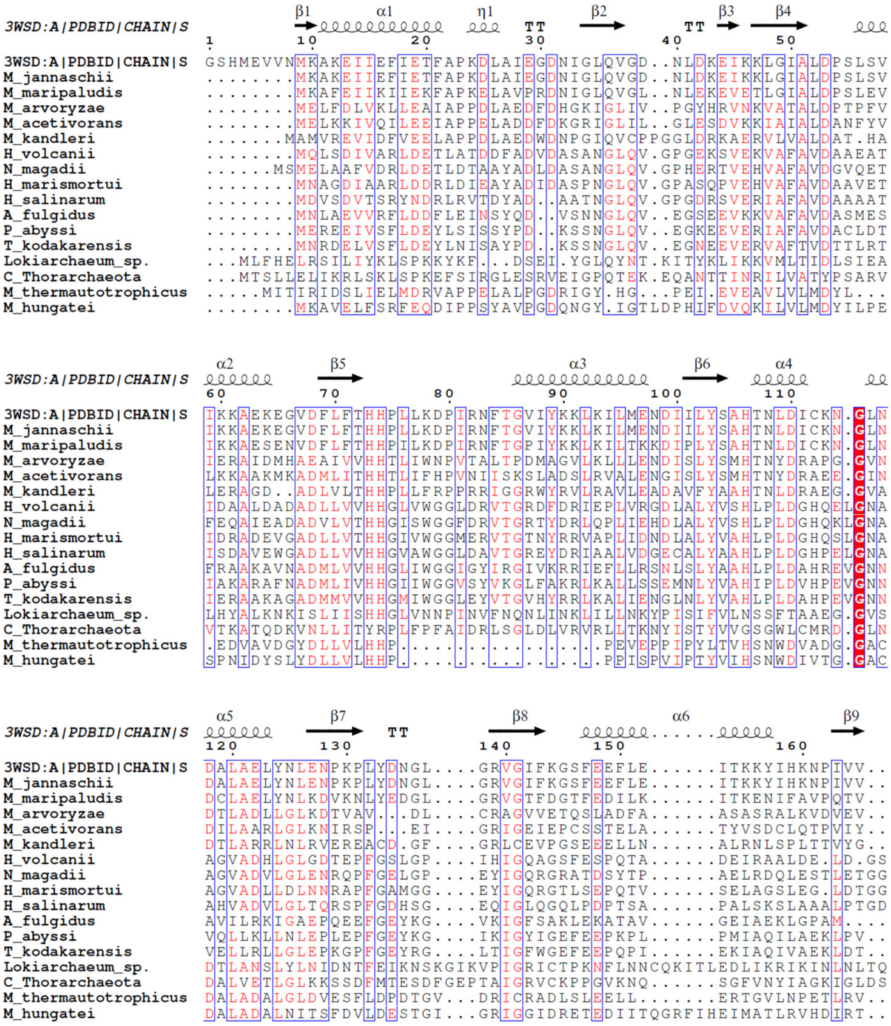

b.

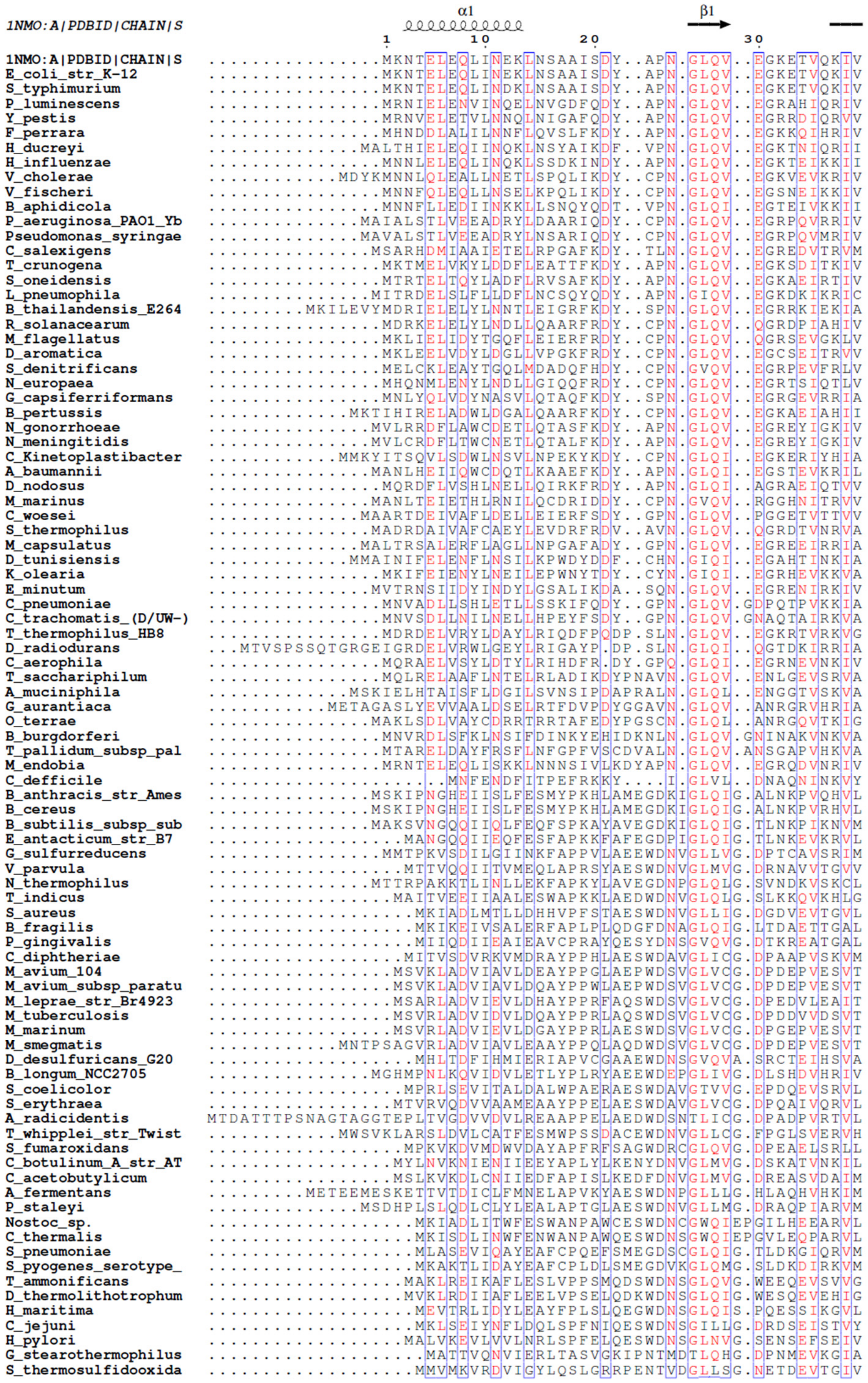
1NMO:A|PDBID|CHAIN|S
α1
β1
1
10
20
30
1NMO:A|PDBID|CHAIN|S
E_coli_str_K-12
S_typhimurium
P_luminescens
Y_pestis
F_perrara
H_ducreyi
H_influenzae
V_cholerae
V_fischeri
B_aphidicola
P_aeruginosa_PAO1_Yb
Pseudomonas_syringae
C_salexigens
T_crunogena
S_oneidensis
L_pneumophila
B_thailandensis_E264
R_solanacearum
M_flagellatus
D_aromatica
S_denitrificans
N_europaea
G_capsiferriformans
B_pertussis
N_gonorrhoeae
N_meningitidis
C_Kinetoplastibacter
A_baumannii
D_nodosus
M_marinus
C_woesei
S_thermophilus
M_capsulatus
D_tunisiensis
K_olearia
E_minutum
C_pneumoniae
C_trachomatis_(D/UW-)
T_thermophilus_HB8
D_radiodurans
C_aerophila
T_saccharophilum
A_muciniphila
G_aurantiaca
O_terrae
B_burgdorferi
T_pallidum_subsp_pal
M_endobia
C_defficile
B_anthracis_str_Ames
B_cereus
B_subtilis_subsp_sub
E_antacticum_str_B7
G_sulfurreducens
V_parvula
N_thermophilus
T_indicus
S_aureus
B_fragilis
P_gingivalis
C_diphtheriae
M_avium_104
M_avium_subsp_paratu
M_leprae_str_Br4923
M_tuberculosis
M_marinum
M_smegmatis
D_desulfuricans_G20
B_longum_NCC2705
S_coelicolor
S_erythraea
A_radicidentis
T_whipplei_str_Twist
S_fumaroxidans
C_botulinum_A_str_AT
C_acetobutylicum
A_fermentans
P_staleyi
Nostoc_sp.
C_thermalis
S_pneumoniae
S_pyogenes_serotype_
T_ammonificans
D_thermolithotrophum
H_maritima
C_jejuni
H_pylori
G_stearothermophilus
S_thermosulfidooxida

```
                          β2         α2                        β3                          α3
1NMO:A|PDBID|CHAIN|S                                                            T.T
                         40        50         60        70        80        90
1NMO:A|PDBID|CHAIN|S     TGVTASQALLDEAVRLG.....ADAVIV..HHGY.FWK.GESPVIRGMKRNRLKTLLAND
E_coli_str_K-12          TGVTASQALLDEAVRLG.....ADAVIV..HHGY.FWK.GESPVIRGMKRNRLKTLLAND
S_typhimurium            TGVTASQALLDEAVRLQ.....ADAVIV..HHGY.FWK.GESPVIRGMKRRRLKTLLAND
P_luminescens            TGVTACQALLDEAVRLQ.....ADAVIV..HHGY.FWK.NEPAVIRGMKRNRLKTLLCND
Y_pestis                 TGVTASQALLDAAVAHQ.....ADAILV..HHGY.FWK.NEPVVVRGMKRNRLKTLLTHD
F_perrara                TGVTACQRLIDAAVDLN.....ADAVLV..HHGF.FWK.NEPLTITGIKYKRIKTLIKHD
H_ducreyi                TGVTASLPLIEQAIAKK.....ADAILV..HHGY.FWK.NESPCIRGMKSKRIKQLLLND
H_influenzae             TGVTASQALINYAISQN.....ADAILV..HHGY.FWK.SETPCIRGMKGKRIKALLVND
V_cholerae               TGVTASMALIEAAIELK.....ADALLV..HHGY.FWK.NEPEAIRGMKGRRIRTLIQND
V_fischeri               TGVTASQALIDAAIEEN.....ADALLV..HHGY.FWK.GEAEPITGMKGKRIRSLIQND
B_aphidicola             TGVTACQALLDKALFYN.....ADTLIV..HHGY.FWK.NESKYIHNMQRQRLKTILSHN
P_aeruginosa_PAO1_Yb     SGVTASQALLDAAVEAD.....ADVVLV..HHGY.FWK.GENPCVVGMKQRRLKTLLNND
Pseudomonas_syringae     SGVTASQALLDAAVDAQ.....ADLVLV..HHGY.FWK.GENPCVVGMKQRRLKTLLKHD
C_salexigens             SGVTACQALLDEAVAWN.....ADLLLV..HHGY.FWK.NEPVEVTGMKRRRLATLLAHD
T_crunogena              TGVTACQALIDAAIAEK.....ADAIIV..HHGY.FWK.NEPVTITGMKQKRIRSLLESE
S_oneidensis             TGVTACQALIDEAIRLK.....ADAILV..HHGF.FWK.SEPEVITGMKQRRIKALLAHD
L_pneumophila            TAVSASEDVISQAIEQQ.....ADALLV..HHGY.FWK.GENPVISGMKRRRIAKLLGHN
B_thailandensis_E264     TGVTASLAFLDRALEWG.....ADAVLV..HHGY.FWR.NEAPQITGRKYQRLKRLLAND
R_solanacearum           TGVTASLALIEAAIDVR.....ADAILV..HHGY.FWK.GEDARVIGQKHARLKQLLAHD
M_flagellatus            TGVTASLTLLEAANRAG.....ADAILV..HHGY.FWR.NESPVVTGIKHARLKFLLQHD
D_aromatica              AGVTASQALLDAAIERH.....ADAILV..HHGY.FWK.GEDGRVTGIRRQRLASLLAND
S_denitrificans          SGVTASLDLLKAAIGVG.....ADAILV..HHGY.FWK.NEDARIAGVKRARIEFLLRHN
N_europaea               SGVTASQALIQAAVGLR.....ADAIIV..HHGY.FWR.GEDACLRGMKRHRIATLIKHD
G_capsiferriformans      TAVTASKQVIDESIAWG.....ADAILV..HHGY.FWR.NEAPCIVGVKKQRIAELLKHD
B_pertussis              TGVTASEALLRAAIERG.....ADAVLV..HHGW.FWR.NEDRRVLGPRRTRLALALGHE
N_gonorrhoeae            TSVTASRAAIDFAVEQK.....ADLLLV..HHGM.FWK.SELPTVTGWKKERIAALLRHD
N_meningitidis           TSVTASRAAIDFAVEQK.....ADLLLV..HHGM.FWK.NELPTVTGWKKERIAALLRHD
C_Kinetoplastibacter     LGVTACELFLQKAIDSN.....ADAVIV..HHGL.LWE.NDNLSITGIKRRRIELCLSNN
A_baumannii              CAVTASEDAIDAAIAQN.....ADLLLV..HHGY.FWK.GEPYPITGMRGNRIKKLIQNN
D_nodosus                TAVSASQAAIDFAVANN.....ADALLV..HHGY.FWK.GEAAELVGVKRKRIATLLHHE
M_marinus                SGVTACMALFEAAVAVN.....AQLIIT..HHGL.FWN.KDPRVVEGMLKHRLKLLLEHD
C_woesei                 TGVSAQLELFERAVDHG.....AELVLA..HHGI.LWD.FDPRRIGPAHARRLRTLLAAD
S_thermophilus           TAVSVSQYTIDAALDWG.....ADVLLV..HHGL.LWG.ERTGPIVGPLRERLKRLLAAD
M_capsulatus             FAVSATAHSIAAAAEWQ.....ADALIV..HHGL.FWKFHGARPLVGPFARRVFPLVRQA
D_tunisiensis            VGVSFNEEFISTAIKEN.....CDLLLV..HHGI.FGK..DFFELKGYLKRRVEKVIKNN
K_olearia                VGVSFNKAFIDHALEER.....VDMLLV..HHGI.FGS..GFFSLKGYIKRRVEKVIKND
E_minutum                FGVSANLEFFKKAAAAK.....ADMVIT..HHGL.LW..SQNPAVTGIFKKRIEFLIKHD
C_pneumoniae             VAVTADLETIKQAVAAE.....ANVLIV..HHGI.FWK.GMPYPITGMIHKRIQLLIEHN
C_trachomatis_(D/UW-)    VAVTADLATIEKAIACE.....ANVLLV..HHGI.FWK.GMPYSITGILYQRMQRLMEGN
T_thermophilus_HB8       AAVDAGEAIFRKALEEE.....VDFLIV..HHGL.FW..GKPFPIVGHHKRRLETLFQGG
D_radiodurans            ASVDTSLQTLQAAAESG.....ADLLLV..HHGL.FW..GRPLAITGPHYERVRTAIQAD
C_aerophila              GAVDAQMPCVEAALSRG.....ADLLLV..HHGI.FW..GPPQRLVGSFGALVRMYLTAG
T_sacchariphilum         VAVDACEYILRAAVEQG.....ADLLIV..HHGL.FW..SGLQPVTGALYRKLKIALEAD
A_muciniphila            AAVDGSEKAIHAALETG.....ADLLLL..HHGI.FW..QPMQPITGIAYRKLKAAMDGN
G_aurantiaca             TAVDASKASIDMAAATG.....ADLLIV..HHGL.FW..SGAQPLVGVRYAKYRTLFERD
O_terrae                 AAVDAGVIPFQRAVDAE.....VDFLIV..HHGM.YW..DMPRPLTGPAYDRVATLINGN
B_burgdorferi            FAVDASFSTLKEAK..G.....NDFLIT..HHGI.FW..SKKERIVSNMYDKTKFLIENN
T_pallidum_subsp_pal     FAVDACAQSIDAAARAG.....ARMLFV..HHGL.FW..GRIEPLTGMQYRRVQALLTHD
M_endobia                TGVTACQALLDSAIAYS.....ADAIIV..HHGY.FWQ.KESPVINGIKRWRLKTLLTND
C_defficile              TTTFPDKEIIKQVIDKD.....EKDILIFSHHAMGYIASDEGFPFHDIPLSYMEEMKNRR
B_anthracis_str_Ames     IALDVTEEVVDEAIQLG.....ANVIIA..HHPL.IFKPLKAIHTDKAYGKIIEKCIKND
B_cereus                 IALDVTEEVVDEAIQLG.....ANVIIA..HHPL.IFNPLKAIHTDKAYGKIIEKCIKND
B_subtilis_subsp_sub     VTLDVLESVIDEAIEKE.....VDLIIA..HHPP.IFRSLKHISTDQPAGRLIEKCLKHD
E_antacticum_str_B7      VTLDVLESVVDEAIEKN.....IDLIIA..HHPP.IFSKLAKVTDQSAAGRIVMKCIRHD
G_sulfurreducens         VALDGTRETVDAAIAAD.....CQLLLT..HHPF.LFRPLKRITANDPTGATVLRAISGG
V_parvula                TTLDVTEEAVEYAIEQN.....CNLIVS..HHPL.IFKGLKQISCDTAQGRTINKLIQHK
N_thermophilus           VALDPSKQVIEQAIKMK.....AELIIT..HHPL.FMPPL.HVVTDEKIGDVTKRAIKND
T_indicus                VCLDLTPETLAQALTQK.....IDCLVT..HHPF.FFKSTKQICWDTWPGKAVKTLAEKD
S_aureus                 TALDCTLEVVNEAIEKG.....YNTIIS..HHPL.IFKGVTSLKAN.GYGLIIRKLIQHD
B_fragilis               LCLDVTEAVLDEAIASG.....CNLIIS..HHPL.IFKGYKSITGKDYVERCILKAIKND
P_gingivalis             LCVDVTEAVLEEAIRLG.....CNLVIA..HHPI.LFKPLKRLTGSSYVERCVELAVRHG
C_diphtheriae            LALDCTQEVADKAVDIG.....ADMLIV..HHPL.LLRGVSSVAADTPKGKVIHTLITGG
M_avium_104              VAVDATPAVVDEVPAGG.......LLLA..HHPL.LLRGVDTVAASTPKGALVHRLIRTG
M_avium_subsp_paratu     VAVDATPAVVDEVPAGG.......LLLA..HHPL.LLRGVDTVAASTPKGALVHRLIRSG
M_leprae_str_Br4923      IAVDATPAVIDEVPDSG.......LLLV..HHPL.LLHGVDTVAVSTPKGALVHRLIRSG
M_tuberculosis           VAVDATPAVVDQVPQAG.......LLLV..HHPL.LLRGVDTVAANTPKGVLVHRLIRTG
M_marinum                VAVDATPAVVDEVPERG.......LLLA..HHPL.LLRGVDTVAANTAKGALIHHLIRTG
M_smegmatis              IAVDATADVIAEVPDRG.......LLLA..HHPL.LLRGVDTVAASTAKGALIHRLIRTG
D_desulfuricans_G20      VALDPSEANIEKAVSQG.....ADFFLA..HHPL.AFKP.QPLSGNDSYFRSVSQLITND
B_longum_NCC2705         FAADPTSAIIDKAIATG.....ADLLIT..HHPL.FFRSVHETSGLGFRGDIVRRLYQHG
S_coelicolor             FAVDPVQETVDEAVGLG.....ADLLVT..HHPL.YLRGTTTVAASTFKGRVVHTLIKND
S_erythraea              FCVDPVEATVDEAADFG.....AQLLIS..HHPL.LLRGVHGVPADDPKGRLVHRMIRAG
A_radicidentis           LAMDPLTAVVDEAIERG.....VDMVIT..HHPL.FLRGTDHVGAVDPKGRSVHRLIRAG
T_whipplei_str_Twist     FVVDVTSATINEAIKAG.....ADLLVS..HHPL.IFKPIKKLTCDTYESFLLSQLIESK
S_fumaroxidans           VALDPGSTTLGEALSLK.....CQCVVT..HHPL.IFQPLESVRADVFPGKLIMTAVREG
C_botulinum_A_str_AT     VALDCTLDVIEEAKKKE.....CNFIVT..HHPL.LFKKPSSITTDTLLGKKIIELIKND
C_acetobutylicum         TALDCTMDVIDEAIEKN.....CNMIIT..HHPI.LFKKPSKITMDTLLGKKIIKIISNN
A_fermentans             VALDLSPEVAEQAIEQE.....CDIIIT..HHPY.YFSLPKTLAVTDTKMEVLYELIKHD
P_staleyi                TCLTITPASAAEAIERQ.....ADLVIA..HHPL.PFRALKRITTDDTVGSLLWNLARHG
Nostoc_sp.               VCLTPTLAVMEEAIALQ.....ANLIFA..HHPL.IFSPPKSLRRGEAIADMARLAFTKN
C_thermalis              VCLTPTLAVMQEAIALQKSGIPVNLIFA..HHPL.IFSPLKSLCSGNAIADMTRLAFRHH
S_pneumoniae             VALDIREETVAEAIEKG.....VDLIIV..KHAP.IFRPIKDLLASRPQNQIYIDLIKHD
S_pyogenes_serotype_     ITLDIRESTVAEAIKNE.....VDLIIT..KHAP.IFKPLKDLVSS.PQRDILLDLVKHD
T_ammonificans           FALSVSREVIECAKERG.....VDLIVT..HHPL.TLSGVKALVPTSYPANLFLELARSS
D_thermolithotrophum     FALSVSKKILEQAKRLK.....VDLIIT..HHPI.TISGIKSFVNYSYPSHLFLELARNG
H_maritima               LSLDVNSQTINEAIKLG.....CNLIVS..HHPL.LFSSTKKIFNDFYPYNVLYKAIQKG
C_jejuni                 LSLDIDENIIKEASENS.......LIIT..HHPL.IFKGLKDLYDKTYPRAFIKEMIYKN
H_pylori                 ACLEITLKIALNAPQNA.......LIIT..HHPL.IFKPLKTLNDEIYPGNILKILIQKN
G_stearothermophilus     TSFMPTYRVIQQAVSME.....ANLLITHEGLFYSHTDNTEMMQKDSVYQEKIRLIRESG
S_thermosulfidooxida     TTFMPTMAVLQQARIRG.....MNLVIAHESPFYDHRCQTPVMD.DPVYRNKQQVIDHTH
```

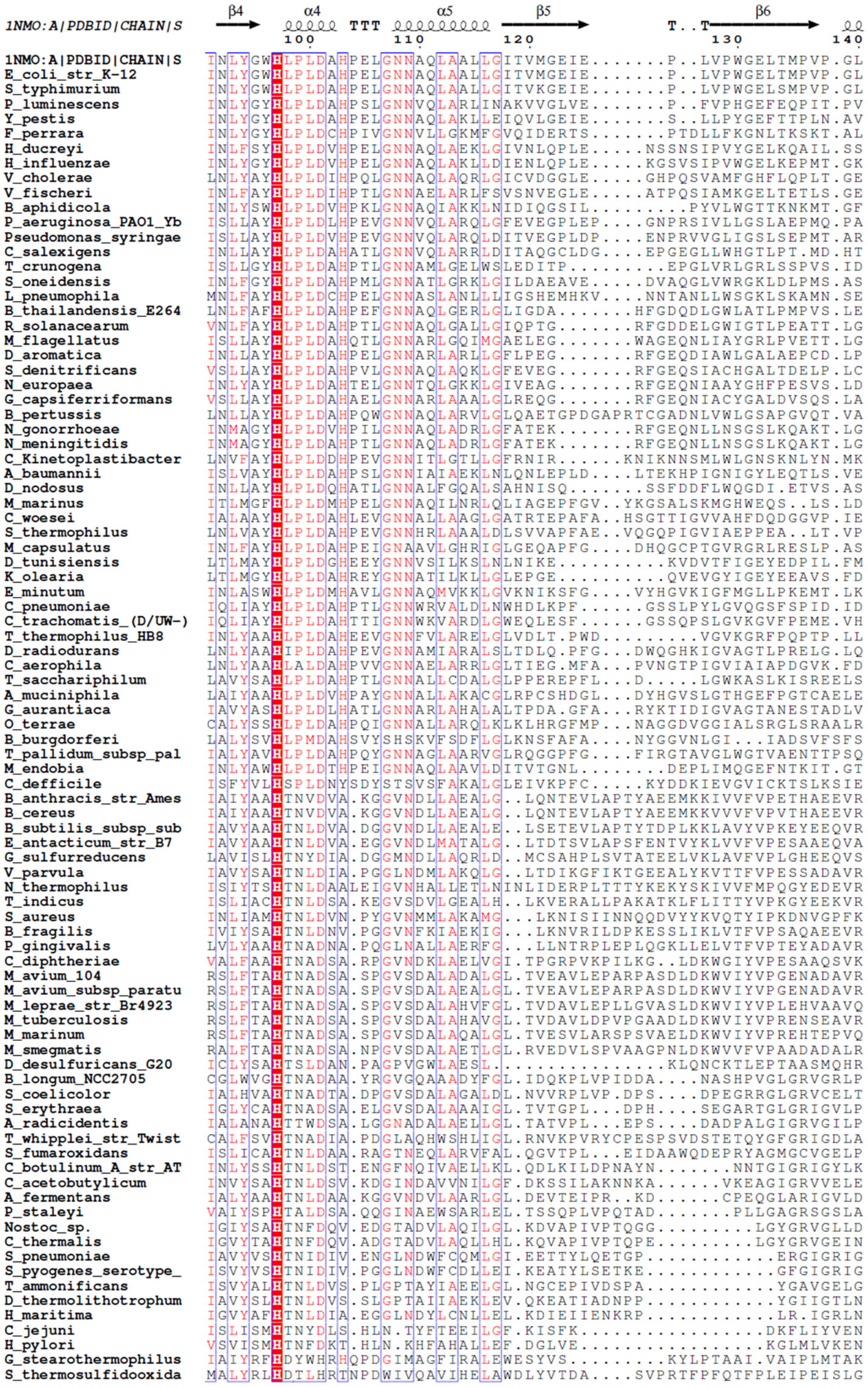
1NMO:A|PDBID|CHAIN|S
β4
α4
TTT
α5
β5
T..T
β6
100
110
120
130
140
1NMO:A|PDBID|CHAIN|S
E_coli_str_K-12
S_typhimurium
P_luminescens
Y_pestis
F_perrara
H_ducreyi
H_influenzae
V_cholerae
V_fischeri
B_aphidicola
P_aeruginosa_PAO1_Yb
Pseudomonas_syringae
C_salexigens
T_crunogena
S_oneidensis
L_pneumophila
B_thailandensis_E264
R_solanacearum
M_flagellatus
D_aromatica
S_denitrificans
N_europaea
G_capsiferriformans
B_pertussis
N_gonorrhoeae
N_meningitidis
C_Kinetoplastibacter
A_baumannii
D_nodosus
M_marinus
C_woesei
S_thermophilus
M_capsulatus
D_tunisiensis
K_olearia
E_minutum
C_pneumoniae
C_trachomatis_(D/UW-)
T_thermophilus_HB8
D_radiodurans
C_aerophila
T_saccharophilum
A_muciniphila
G_aurantiaca
O_terrae
B_burgdorferi
T_pallidum_subsp_pal
M_endobia
C_defficile
B_anthracis_str_Ames
B_cereus
B_subtilis_subsp_sub
E_antacticum_str_B7
G_sulfurreducens
V_parvula
N_thermophilus
T_indicus
S_aureus
B_fragilis
P_gingivalis
C_diphtheriae
M_avium_104
M_avium_subsp_paratu
M_leprae_str_Br4923
M_tuberculosis
M_marinum
M_smegmatis
D_desulfuricans_G20
B_longum_NCC2705
S_coelicolor
S_erythraea
A_radicidentis
T_whipplei_str_Twist
S_fumaroxidans
C_botulinum_A_str_AT
C_acetobutylicum
A_fermentans
P_staleyi
Nostoc_sp.
C_thermalis
S_pneumoniae
S_pyogenes_serotype_
T_ammonificans
D_thermolithotrophum
H_maritima
C_jejuni
H_pylori
G_stearothermophilus
S_thermosulfidooxida

```
                        α6                                         β7          β8
1NMO:A|PDBID|CHAIN|S    ℓℓℓℓℓℓℓℓℓℓℓ                                →     TT    ━━━━━
                                 150                                      160
1NMO:A|PDBID|CHAIN|S    ELASWIEARLG...........................RKPLWC....GDTGPEVVQRVA
E_coli_str_K-12         ELASWIEARLG...........................RKPLWC....GDTGPEVVQRVA
S_typhimurium           ELASWIEARLG...........................RKPLWC....GDTGPENVQRVA
P_luminescens           ELAARIEQALG...........................RKALYV....GDNAPAEIRKLA
Y_pestis                ALRERLEKQLG...........................RSVLHC....GDRAPAEVRRIA
F_perrara               ELKSLIEEKLQ...........................RTVLFC....GDDAPEQIKRVA
H_ducreyi               EFARQIEKTLA...........................RKPIFCGDFVEGYPQKAIKTVA
H_influenzae            DFAEKIEKVLN...........................RKPLIC...IENGPH.LIRKIG
V_cholerae              ELAHRIGQVLN...........................RTPLHI...APDQADKLIETVG
V_fischeri              EFAQRIQQVLN...........................REPLHI...KPTQ.DKPIKTVA
B_aphidicola            EFANKIERKFK...........................KYPIHL....YENAPLYISRVA
P_aeruginosa_PAO1_Yb    DFARHVRDALG...........................REPLLV.....DA.GQPIRRIA
Pseudomonas_syringae    DFARRVQDALG...........................REPLLI.....EG.SQMIRRVG
C_salexigens            AFARLIESRLG...........................RSPLVI.....NGHSRPLKRVA
T_crunogena             TFIESVGNSLN...........................RMPLHL.....PGGPSEVETVA
S_oneidensis            EFAAMLAEVLG...........................RDPLHI.....GESSDEIQYLA
L_pneumophila           QFSSFLEHKLG...........................RYPVHI.....AGNEKMIHSIA
B_thailandensis_E264    HFTAKVENTLG...........................RAPLVL.....GDSDQQLRRVA
R_solanacearum          AFAEVVAARLD...........................RMPLVI.....GGADRPVHTIG
M_flagellatus           AFSDTLSNQLR...........................RSPQVL.....GDPQKKVRRIA
D_aromatica             SLVSRVAGVLG...........................RQPLVV.....GGGDRLIRRIG
S_denitrificans         GLEERVTRVLG...........................RAPLII.....GEGDTPVRRIA
N_europaea              ELSNLLNSVLG...........................RKPLII.....GDPLKPVRRVA
G_capsiferriformans     DFALGIESALQ...........................RAPLVI.....GHAGKMLHRAA
B_pertussis             QLQQRVAERLG...........................RTPLVV.....GEPDRPVRRVA
N_gonorrhoeae           ALAAHIETVLQ...........................RKPVAI.....GNPEREIRRVA
N_meningitidis          ALAAHIETVLQ...........................RKPVVI.....GNPEREIRRVA
C_Kinetoplastibacter    DLNTVISNKLN...........................RSALLI.....GDPKKNIKNIV
A_baumannii             EFKAKLQNSFD...........................FKVIHL.....PAEKQSIQKVG
D_nodosus               QWIAAVEKILQ...........................RSIIAV.....GNTQKMIQKIA
M_marinus               QVQQKLQKLFG...........................GEPLVL.....PYGPPNIRKVA
C_woesei                ELVARVATLTD...........................RDPLVN.....AAGPPAVRTLG
S_thermophilus          DLVARLERVTE...........................RAPVVL.....DGHHGPLRRVA
M_capsulatus            ALRERLAEILE...........................HPVLLA....TPDATAPVCTIG
D_tunisiensis           DFHKQVEKLFN...........................PQPLLV.....YQNNKFVKTVG
K_olearia               DFYKRLKQIFP...........................NQPILK.....YVNSDFVKKVC
E_minutum               E....VESKFA...........................VKPNLV....FNFGKSKNKIVA
C_pneumoniae            SFIDLLSQYYQ...........................APLKGS....ALGGPSRVSSAA
C_trachomatis_(D/UW-)   DFISQLSAYYQ...........................TPVLAK....ALGGKKRVSSAA
T_thermophilus_HB8      QVADRLGQLTG...........................MQPLV.....HQGGLDHVETVI
D_radiodurans           DFADRIQKLTG...........................EICLV.....HGGGSPNIHRVG
C_aerophila             YLVDRFEQNVG...........................PVRLV.....QAHGPRISHKIG
T_sacchariphilum        DRL...ARAVG...........................GRVHV....APGGAGTIRSVGV
A_muciniphila           QKL...ETVLG...........................APVQA..FWKDSPEAPAGDIFI
G_aurantiaca            ARVSHYAAGYG...........................GTVRT..SVPVDGRTTKRWAIC
O_terrae                AKLEKLYPRVI...........................AI.........EYGSPTPREIA
B_burgdorferi           EILEK.................................IKKENKHILFSKKFKESVNKVA
T_pallidum_subsp_pal    EAMQQHAACTAPDTHRVTHANAISPSAGLSLQQVVHRLFPAEEQPVRLLPFGKQRIERVG
M_endobia               ELHRRLEQTLR...........................GNVLHY....GDGGPAYIKRLA
C_defficile             EIKELIKKSVG............................HDVKLYDYGDSVLKDGLVAIA
B_anthracis_str_Ames    KALGDAGAGHI...................GNYSHCTFSSEGTGAFIPQEGTNPYIGETG
B_cereus                KALGDAGAGHI...................GNYSHCTFSSEGTGTFVPQEGTNPYIGETG
B_subtilis_subsp_sub    AALGNAGAGHI...................GEYSHCAFSSEGIGSFKPLDGAKPFIGEVG
E_antacticum_str_B7     EALGRAGAGHI...................GAYSGCQFHSTGIGQFKPTADANPYRGKPG
G_sulfurreducens        EALFRF.SGTV...................GTYRDCSFRSGGTGTFRPLEGARPFLGTVG
V_parvula               MAMGDAGAGRI...................GNYEYCSFSTHGEGRFVGNEDSHPVIGSAG
N_thermophilus          TALSSAGAGSL...................GHYCDVSFQSQGIGTFKPLAGTDPFIGEQG
T_indicus               EILLKEGAGQR...................GKYSGCSFATEGIGSFYPKEEAKPYRGKVG
S_aureus                DKLSENGLAQE...................GNYEYCFFESEGRGQFKPVGEANPTIGQID
B_fragilis              NALFTAGCGCI...................GNYDSCSYNTEGEGTFRAQEGSHPFCGTVG
P_gingivalis            QALWQAGAGRL...................GHYDCCSFSHAGTGTFRAAEGANPFVGAIS
C_diphtheriae           DAVFEAGAGAI...................GDYSQCSFEWQGTGQFLPHHNAQPVVGHPG
M_avium_104             EAVFAAGAGHI...................GDYSHCSWSVTGIGQFLPHEGASPAVGSVG
M_avium_subsp_paratu    EAVFAAGAGHI...................GDYSHCSWSVTGIGQFLPHEGASPALGSVG
M_leprae_str_Br4923     AAVFEAGAGHI...................GDYSHCSWSVTGTGQFMPHDGASPVVGSIG
M_tuberculosis          AAVFEAGAGHI...................GDYSHCSWSVAGTGQFLAHDGASPAIGSVG
M_marinum               AAIFDAGAGHI...................GDYSHCSWSVTGIGQFLPLDGASPAIGSVG
M_smegmatis             EALFAAGAGRI...................GDYSHCSWSVAGTGQFLPHDGASPAIGEVG
D_desulfuricans_G20     IIAPSAASGSI...................PAVIAGSIRCR.............HIQGIG
B_longum_NCC2705        KPVALKDFAQR...................VFDEVSDHGMTSALGIQVCGDSDTFVQYVA
S_coelicolor            HPLTVRELAAR...................AAERLP....ATAQGIRVAGDPEATVRTVA
S_erythraea             EPEPFERFVQR...................VAAGLP....TTAWGVRGAGDPQRPVRTVA
A_radicidentis          EPTTLRALAEH...................VARALP....DTAPGLLVGGDPEAAVERVA
T_whipplei_str_Twist    EPVSLYDFAKD...................LKAFLP....ETVGGIMVAGDEAKEIHSAA
S_fumaroxidans          AEMSLDGLASF...................AAAAMG......GGAVRVVGEPGRSVRRVA
C_botulinum_A_str_AT    EEVTLDKLCEI...................VKEVFN......IPALRYSGEDSLKIKKVA
C_acetobutylicum        QNMTLKELCDR...................VKESFK......IQSLRYCGDEDKKIHSFA
A_fermentans            EPMALKDFAAK...................VRDDLG......AEHLTYAGGDE.PVYKVA
P_staleyi               TTETLAQFITR...................VKRAVK......IEHVAYVGQLAQAITRVA
Nostoc_sp.              PFLNLQELLTV...................IQTRLA....PPDLIFSPTADLQQIISRVA
C_thermalis             PSCTLQELLQQ...................IQTVLN....PPDLIFSPTVDLQKTIERVA
S_pneumoniae            NIQPQTFWELA...................QQVKQVFDLDSLRMVHYQENDLQKPISRVA
S_pyogenes_serotype_    TVKEQALEELA...................SKVKRVFDLDTVRLIRYDKEN..PLISKIA
T_ammonificans          EPKTQRELLVK...................LAGVLGVGADVFRCVNYRPE...AVVRRVA
D_thermolithotrophum    TALTQKELFKK...................LTSFLP..KDIFRGINYRPD...SAVKRIA
H_maritima              RGLDFEVFVDY...................VKEKLS..CDAIKYIKAN.D...RLIKRVA
C_jejuni                S.MSFEALCDW...................VKKKLN......LQILRVSDCGKKDIKRIA
H_pylori                ANIEFDALVKK...................IKSSLG......VGSLACVK.SSQTIKDLA
G_stearothermophilus    EVAEYAKEMLS............................IPFVRIAGDLSAPCTRIGILV
S_thermosulfidooxida    DLALYVKHRLQ............................APSVRVVGDLNRVCHRIGLLP
```

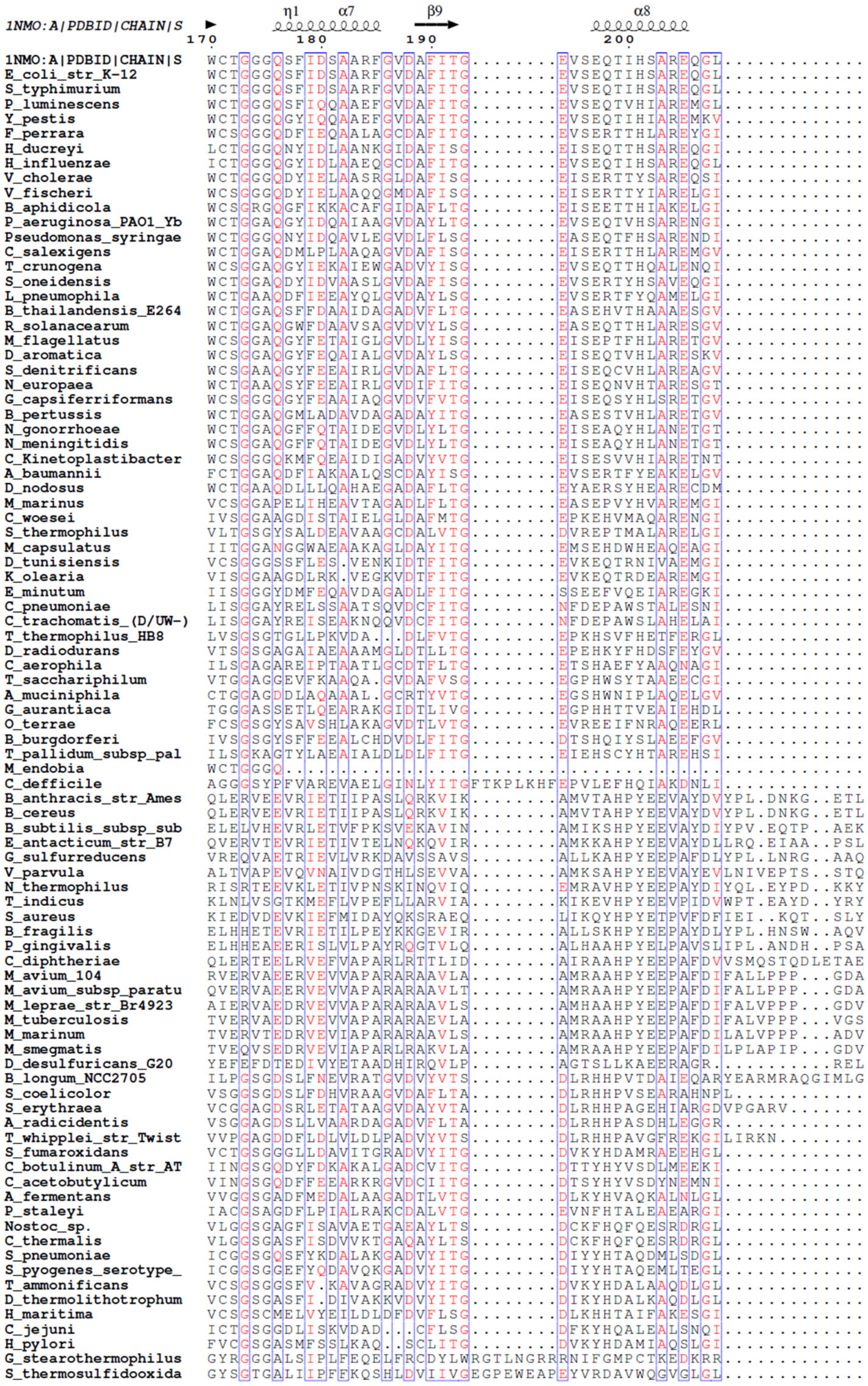
1NMO:A|PDBID|CHAIN|S
η1
α7
β9
α8
170
180
190
200

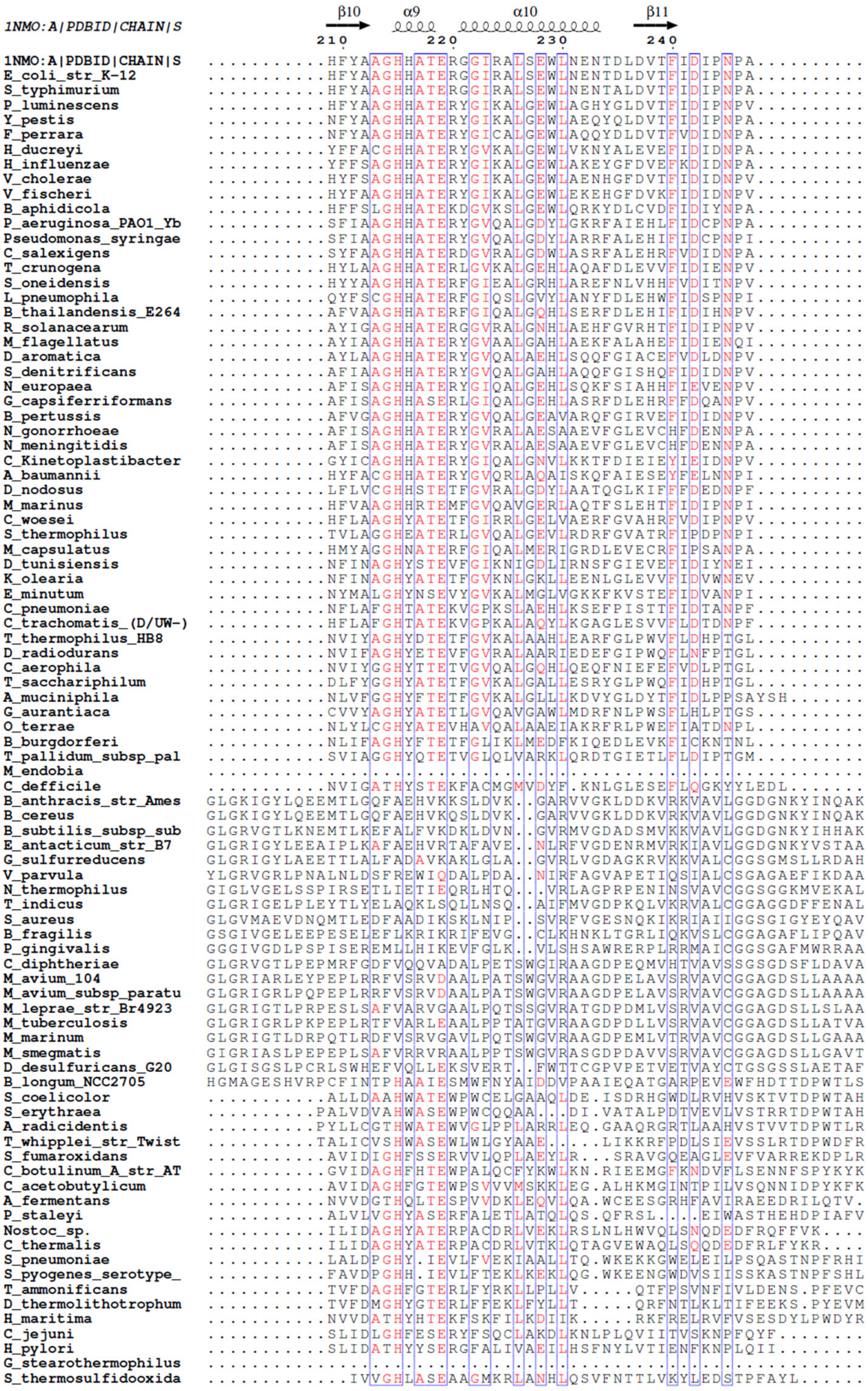
1NMO:A|PDBID|CHAIN|S
β10
α9
α10
β11
210
220
230
240
1NMO:A|PDBID|CHAIN|S
E_coli_str_K-12
S_typhimurium
P_luminescens
Y_pestis
F_perrara
H_ducreyi
H_influenzae
V_cholerae
V_fischeri
B_aphidicola
P_aeruginosa_PAO1_Yb
Pseudomonas_syringae
C_salexigens
T_crunogena
S_oneidensis
L_pneumophila
B_thailandensis_E264
R_solanacearum
M_flagellatus
D_aromatica
S_denitrificans
N_europaea
G_capsiferriformans
B_pertussis
N_gonorrhoeae
N_meningitidis
C_Kinetoplastibacter
A_baumannii
D_nodosus
M_marinus
C_woesei
S_thermophilus
M_capsulatus
D_tunisiensis
K_olearia
E_minutum
C_pneumoniae
C_trachomatis_(D/UW-)
T_thermophilus_HB8
D_radiodurans
C_aerophila
T_saccharaphilum
A_muciniphila
G_aurantiaca
O_terrae
B_burgdorferi
T_pallidum_subsp_pal
M_endobia
C_defficile
B_anthracis_str_Ames
B_cereus
B_subtilis_subsp_sub
E_antacticum_str_B7
G_sulfurreducens
V_parvula
N_thermophilus
T_indicus
S_aureus
B_fragilis
P_gingivalis
C_diphtheriae
M_avium_104
M_avium_subsp_paratu
M_leprae_str_Br4923
M_tuberculosis
M_marinum
M_smegmatis
D_desulfuricans_G20
B_longum_NCC2705
S_coelicolor
S_erythraea
A_radicidentis
T_whipplei_str_Twist
S_fumaroxidans
C_botulinum_A_str_AT
C_acetobutylicum
A_fermentans
P_staleyi
Nostoc_sp.
C_thermalis
S_pneumoniae
S_pyogenes_serotype_
T_ammonificans
D_thermolithotrophum
H_maritima
C_jejuni
H_pylori
G_stearothermophilus
S_thermosulfidooxida

1NMO:A|PDBID|CHAIN|S

```
1NMO:A|PDBID|CHAIN|S  ............................................................
E_coli_str_K-12       ............................................................
S_typhimurium         ............................................................
P_luminescens         ............................................................
Y_pestis              ............................................................
F_perrara             ............................................................
H_ducreyi             ............................................................
H_influenzae          ............................................................
V_cholerae            ............................................................
V_fischeri            ............................................................
B_aphidicola          ............................................................
P_aeruginosa_PAO1_Yb  ............................................................
Pseudomonas_syringae  ............................................................
C_salexigens          ............................................................
T_crunogena           ............................................................
S_oneidensis          ............................................................
L_pneumophila         ............................................................
B_thailandensis_E264  ............................................................
R_solanacearum        ............................................................
M_flagellatus         ............................................................
D_aromatica           ............................................................
S_denitrificans       ............................................................
N_europaea            ............................................................
G_capsiferriformans   ............................................................
B_pertussis           ............................................................
N_gonorrhoeae         ............................................................
N_meningitidis        ............................................................
C_Kinetoplastibacter  ............................................................
A_baumannii           ............................................................
D_nodosus             ............................................................
M_marinus             ............................................................
C_woesei              ............................................................
S_thermophilus        ............................................................
M_capsulatus          ............................................................
D_tunisiensis         ............................................................
K_olearia             ............................................................
E_minutum             ............................................................
C_pneumoniae          ............................................................
C_trachomatis_(D/UW-) ............................................................
T_thermophilus_HB8    ............................................................
D_radiodurans         ............................................................
C_aerophila           ............................................................
T_sacchariphilum      ............................................................
A_muciniphila         ............................................................
G_aurantiaca          ............................................................
O_terrae              ............................................................
B_burgdorferi         ............................................................
T_pallidum_subsp_pal  ............................................................
M_endobia             ............................................................
C_defficile           ............................................................
B_anthracis_str_Ames  FKGADVYVTGDMYYHVAHD.AMMLGLNIVDPGHN.VEKVMKQGVQKQLQEKVDAKKL.NV
B_cereus              FKGADVYVTGDMYYHVAHD.AMMLGLNIVDPGHN.VEKVMKQGVQKQLQEKVDAKKL.NV
B_subtilis_subsp_sub  RKGADVYVTGDLYFHVAHD.AMMLGLNVVDPGHY.AEKIMKEGVTRKLTSMCNDKKF.GV
E_antacticum_str_B7   FAGADVLVTGDLYFHVAHD.AMALGLAVVDPGHH.VESVMKQGVVDLLTERFDKQNIKGV
G_sulfurreducens      RQGADVFVTGDVKYHEARD.AEALGIALLDAGHFGTEAIMVSGVAERLTADLVLKGF.EA
V_parvula             RLHVDAYITGDVKYHEAQM.AKELGLLVVDAGHFGTESIVARGLCDYLISVGFT.....V
N_thermophilus        KLGVDAFITGDIKYHDALR.AEQEGLFLIDAGHRETELPVVDKVTKFLDEQLSDDY..NL
T_indicus             SLGAEVYITAEVKYHQARE.AEARGLSLISVGHFESESLIVPKMAEFFQKMANSKG..EE
S_aureus              QQGADVFVTGDIKHHDALD.AKIHGVNLIDINHY.SEYVMKEGLKTLLMNWFNIEKI.NI
B_fragilis            RSGADVFITGEIKYHDYFG.RETD.ILLAEIGHYESEQYTKEIFYSIIRDLFP.....NF
P_gingivalis          QEGADLFLTGEAKYNDFFD.AGEH.LLLVTIGHYESEEVANELFMRIISQKFP.....TF
C_diphtheriae         ALGVDVYVTSDLRHHPADESRRAQGPLLIDTAHWASEFPWTYQVQELLAQEFAQ.....L
M_avium_104           GAGVQAYLTADLRHHPADEHRRASEVALIDVAHWASEFPWCAQAADLLRSRFGG....RL
M_avium_subsp_paratu  GAGVQAYLTADLRHHPADEHRRASEVALIDVAHWASEFPWCAQAADLLRSRFGE....RL
M_leprae_str_Br4923   VADVQAYVTADLRHHPADEHRRASNVALIDVAHWASEFPWCGQAADVLRSHFGT....AL
M_tuberculosis        AADVQAYVTADLRHHPADEHCRASQVALIDVAHWASEFPWCGQAAEVLRSHFGA....SL
M_marinum             AADVQAYLTADLRHHPADEHRRASAVALIDVAHWASEFPWCGQAAEVLRSHFGE....AL
M_smegmatis           AAGVQAYVTADLRHHPADEHRRASDVALVDVAHWASEFPWCAQAAGVLRKHFSD....AL
D_desulfuricans_G20   ARGADVFITGDVKYHTALDTTGC....IIDVGHFSLEEEMMRRFAALLAAGAPD......
B_longum_NCC2705      IAAHRD......................................................
S_coelicolor          VASAPTSSAMGAPN..............................................
S_erythraea           VAGAVSDPGRTLA...............................................
A_radicidentis        LSTGS.......................................................
T_whipplei_str_Twist  V...........................................................
S_fumaroxidans        IVGSAVEPLSP.................................................
C_botulinum_A_str_AT  ............................................................
C_acetobutylicum      ............................................................
A_fermentans          ............................................................
P_staleyi             ............................................................
Nostoc_sp.            ............................................................
C_thermalis           ............................................................
S_pneumoniae          ............................................................
S_pyogenes_serotype_  ............................................................
T_ammonificans        DVER........................................................
D_thermolithotrophum  RSC.........................................................
H_maritima            ............................................................
C_jejuni              ............................................................
H_pylori              ............................................................
G_stearothermophilus  ............................................................
S_thermosulfidooxida  ............................................................
```

1NMO:A|PDBID|CHAIN|S

```
1NMO:A|PDBID|CHAIN|S     .............................
E_coli_str_K-12          .............................
S_typhimurium            .............................
P_luminescens            .............................
Y_pestis                 .............................
F_perrara                .............................
H_ducreyi                .............................
H_influenzae             .............................
V_cholerae               .............................
V_fischeri               .............................
B_aphidicola             .............................
P_aeruginosa_PAO1_Yb     .............................
Pseudomonas_syringae     .............................
C_salexigens             .............................
T_crunogena              .............................
S_oneidensis             .............................
L_pneumophila            .............................
B_thailandensis_E264     .............................
R_solanacearum           .............................
M_flagellatus            .............................
D_aromatica              .............................
S_denitrificans          .............................
N_europaea               .............................
G_capsiferriformans      .............................
B_pertussis              .............................
N_gonorrhoeae            .............................
N_meningitidis           .............................
C_Kinetoplastibacter     .............................
A_baumannii              .............................
D_nodosus                .............................
M_marinus                .............................
C_woesei                 .............................
S_thermophilus           .............................
M_capsulatus             .............................
D_tunisiensis            .............................
K_olearia                .............................
E_minutum                .............................
C_pneumoniae             .............................
C_trachomatis_(D/UW-)    .............................
T_thermophilus_HB8       .............................
D_radiodurans            .............................
C_aerophila              .............................
T_sacchariphilum         .............................
A_muciniphila            .............................
G_aurantiaca             .............................
O_terrae                 .............................
B_burgdorferi            .............................
T_pallidum_subsp_pal     .............................
M_endobia                .............................
C_defficile              .............................
B_anthracis_str_Ames     NIHASQLHTDPFTFV..............
B_cereus                 HIHASQLHTDPFIFV..............
B_subtilis_subsp_sub     NIFVSETDTNPFTFL..............
E_antacticum_str_B7      ELFVSVTNTNPFQFL..............
G_sulfurreducens         DVVAFNGERDPFVWR..............
V_parvula                PVKAFTEQNDFFFV...............
N_thermophilus           EIKSFHNDEYVFYR...............
T_indicus                LQITMLKEKNPFIPLF.............
S_aureus                 DVEASTINTDPFQYI..............
B_fragilis               ALQFSKVNTNPIKYL..............
P_gingivalis             ATHKSSVATNPVNYL..............
C_diphtheriae            DVEVATLRTDPWNISAHPQLR........
M_avium_104              DVRVSSIRTDPWNVEHDGGEGR.......
M_avium_subsp_paratu     DVRVSAIRTDPWNVGHDGGEG........
M_leprae_str_Br4923      SVRVCTIRTDPWNLGARRVNDVSDSGRDQ
M_tuberculosis           PVRVCTICTDPWNLDHETGRDQA......
M_marinum                PVRVCPLRTDPWNVAASRENDS.......
M_smegmatis              PVRVCEVRTDPWNVESLEEHES.......
D_desulfuricans_G20      LDITFLPSQDPIRLHPVHG..........
B_longum_NCC2705         .............................
S_coelicolor             .............................
S_erythraea              .............................
A_radicidentis           .............................
T_whipplei_str_Twist     .............................
S_fumaroxidans           .............................
C_botulinum_A_str_AT     .............................
C_acetobutylicum         .............................
A_fermentans             .............................
P_staleyi                .............................
Nostoc_sp.               .............................
C_thermalis              .............................
S_pneumoniae             .............................
S_pyogenes_serotype_     .............................
T_ammonificans           .............................
D_thermolithotrophum     .............................
H_maritima               .............................
C_jejuni                 .............................
H_pylori                 .............................
G_stearothermophilus     .............................
S_thermosulfidooxida     .............................
```

c.

```
H_sapiens_model_Q9GZ

H_sapiens_model_Q9GZ   ............................................................
H_sapiens              ............................................................
P_troglodytes          ............................................................
M_mulatta              ............................................................
M_fascicularis         ............................................................
C_lupus                ............................................................
B_taurus               ............................................................
C_griseus              ............................................................
M_musculus             ............................................................
M_domestica            ............................................................
G_gallus               ............................................................
A_carolinensis         ............................................................
L_chalumnae            ............................................................
R_norvegicus           ............................................................
A_mississippiensis     ............................................................
X_maculatus            ............................................................
O_niloticus            ............................................................
S_formosus             ............................................................
D_rerio                ............................................................
N_vectensis            ............................................................
S_purpuratus           ............................................................
C_intestinalis         ............................................................
C_gigas                ............................................................
T_adhaerens            ............................................................
C_owczarzaki           ............................................................
A_queenslandica        ............................................................
A_pisum                ............................................................
T_urticae              ............................................................
D_discoideum           ............................................................
O_dioica               ............................................................
D_pulex                ............................................................
H_robusta              ............................................................
Z_nevadensis           ........MGKL.ELIQTLLGHEGRVWDVCWH.PQGNTLASCGEDKTIRIWGREGSKWAN
R_allomycis            MNENHEYYPGMLTQMASQVDPHEDPFTKLAYQLAQQDLNKENRASSNRRPLSPLNNTSSS
S_mansoni              ............................................................
H_microstoma           ............................................................
E_granulosus           ............................................................
G_theta                ............................................................
S_maritima             ............................................................
A_mexicanus            ............................................................
A_fumigatus            ............................................................
N_fumigata             ............................................................
R_mackenziei_CBS_650   ............................................................
E_mesophila            ............................................................
X_heveae               ............................................................
A_subglaciale          ............................................................
P_nodorum              ............................................................
B_bassiana             ............................................................
G_zeae                 ............................................................
N_crassa_OR74A         ............................................................
E_lata                 ............................................................
C_cerevisiae           ............................................................
S_pombe                ............................................................
S_complicata           ............................................................
Y_lipolytica           ............................................................
T_melanosporum         ............................................................
A_oligospora           ............................................................
P_carinii              ............................................................
T_deformans            ............................................................
W_ichthyophaga         ............................................................
S_punctatus            ............................................................
B_dendrobatidis        ............................................................
C_neoformans           ............................................................
A_macrogynus           ............................................................
B_saltans              ............................................................
T_cruzi                ............................................................
L_major                ............................................................
M_osmundae             ............................................................
M_lychnidis-dioicae    ............................................................
Perkinsela_sp.         ............................................................
R_irregularis          ............................................................
G_prolifera            ............................................................
B_floridae             ............................................................
F_alba                 ............................................................
T_anomala              ............................................................
U_maydis               ............................................................
S_borealis             ............................................................
M_globosa              ............................................................
P_graminis             ............................................................
B_mori                 ............................................................
N_vitripennis          ............................................................
P_humanus              ............................................................
D_melanogaster         ............................................................
A_gambiae              ............................................................
L_gigantea             ............................................................
I_scapularis           ............................................................
X_tropicalis           ............................................................
E_histolytica          ............................................................
C_viscosa              ............................................................
A_niger_YbgI           .......MPPTISPLACCIPRTPISRSFRNFHSSRLSRTLSPLSSSTSSPFRSPLSVSLP
R_microsporus          ................................MSLLSKVTRCMRAIAPLELAETSWDNVG
Chrysochromulina_sp.   ...............MAWFGSPRGPPNAASSFTHLISSSLERRFPKKFASAGHTSGLTIG
E_huxleyi              .....................MRAGNVHLQPFHRVLSASLSRRFPEAIAEAGDTVGLTID
N_ceranae              MADNRFLYIFNIYGLIFFIYVSRFNTLKVKPQLMLITDIEDKINQIYPLSGAQ.EWDNVG
E_cuniculi             .................................MSIHEVSKAIDRFASLRRAKTDWDNVG
V_culicis              ...................................MNHILPFFQLYAPLSGAA.SWDNVG
C_cinerea              ............................................................
B_hominis              ............................................................
C_albicans             ............................................................
A_castellanii          ............................................................
consensus>50           ............................................................
```

H_sapiens_model_Q9GZ

```
H_sapiens_model_Q9GZ   ............................................................
H_sapiens              ............................................................
P_troglodytes          ............................................................
M_mulatta              ............................................................
M_fascicularis         ............................................................
C_lupus                ............................................................
B_taurus               ............................................................
C_griseus              ............................................................
M_musculus             ............................................................
M_domestica            ............................................................
G_gallus               ............................................................
A_carolinensis         ............................................................
L_chalumnae            ............................................................
R_norvegicus           ............................................................
A_mississippiensis     ............................................................
X_maculatus            ............................................................
O_niloticus            ............................................................
S_formosus             ............................................................
D_rerio                ............................................................
N_vectensis            ............................................................
S_purpuratus           ............................................................
C_intestinalis         ............................................................
C_gigas                ............................................................
T_adhaerens            ............................................................
C_owczarzaki           ............................................................
A_queenslandica        ............................................................
A_pisum                ............................................................
T_urticae              ............................................................
D_discoideum           ............................................................
O_dioica               ............................................................
D_pulex                ............................................................
H_robusta              ............................................................
Z_nevadensis           KTILAD....GHQRTIREVAW.SPCGN.YLASASFDATTAVWDKKSGQFECNATLEGHEN
R_allomycis            AYVLKI....DNSKIRREKNYNSPVGTPFRDLKKIDRLENRKTPEKSTAKSNPVNESYYG
S_mansoni              ............................................................
H_microstoma           ............................................................
E_granulosus           ............................................................
G_theta                ............................................................
S_maritima             ............................................................
A_mexicanus            ............................................................
A_fumigatus            ............................................................
N_fumigata             ............................................................
R_mackenziei_CBS_650   ............................................................
E_mesophila            ............................................................
X_heveae               ............................................................
A_subglaciale          ............................................................
P_nodorum              ............................................................
B_bassiana             ............................................................
G_zeae                 ............................................................
N_crassa_OR74A         ............................................................
E_lata                 ............................................................
C_cerevisiae           ............................................................
S_pombe                ............................................................
S_complicata           ............................................................
Y_lipolytica           ............................................................
T_melanosporum         ............................................................
A_oligospora           ............................................................
P_carinii              ............................................................
T_deformans            ............................................................
W_ichthyophaga         ............................................................
S_punctatus            ............................................................
B_dendrobatidis        ............................................................
C_neoformans           ............................................................
A_macrogynus           ............................................................
B_saltans              ............................................................
T_cruzi                ............................................................
L_major                ............................................................
M_osmundae             ............................................................
M_lychnidis-dioicae    ............................................................
Perkinsela_sp.         ............................................................
R_irregularis          ............................................................
G_prolifera            ............................................................
B_floridae             ............................................................
F_alba                 ............................................................
T_anomala              ............................................................
U_maydis               ............................................................
S_borealis             ............................................................
M_globosa              ............................................................
P_graminis             ............................................................
B_mori                 ............................................................
N_vitripennis          ............................................................
P_humanus              ............................................................
D_melanogaster         ............................................................
A_gambiae              ............................................................
L_gigantea             ............................................................
I_scapularis           ............................................................
X_tropicalis           ............................................................
E_histolytica          ............................................................
C_viscosa              ............................................................
A_niger_YbgI           ASSTRLTSTSITNTTLPTIKKFSYQIVQNRTMSAAPVPDRYKLVFFVPHSHLETC....K
R_microsporus          VLVEPPFPSPKQNKVLLTIDLTPQVLEEALADPKFGTIVSYHPPIFRGFKRLTSDDPQQA
Chrysochromulina_sp.   TLREAE.RG.GTAVVVICVDLTEQVLAEAIS.LHANVIIACSLFPSEPLRSLRVDEPVGR
E_huxleyi              TLRDVE.RG.ASALVLLCVDLTEQVLSEAAS.ARAHFVVTYSPMPHSPFTTLSTDDVLGR
N_ceranae              VLVDTG..S.SSNKVLLTIDVTELVIDECNR.LGIKHIISYHPVIFSGVKKL.LKN...D
E_cuniculi             VIVDSG..T.QDKKILLTIDLTEPVLEECIH.LGVKNVVAYHPIIFKAVKKLGSKE...S
V_culicis              ILIPSNIEN.NDRKYLLTIDFTPQVLRECIE.KNIKKVISYHPVLFNAQRDVGL......
C_cinerea              .............MSNPLLNLTTSVLSEALA.KETSVIVAYHPTIFKGLQSLTLANPLQR
B_hominis              ................................MNASAIITYHPIIFHGIKKLTPDDRVAR
C_albicans             ......................................MAYHPFIFRGLKSITHNDPQQR
A_castellanii          ............................................................
consensus>50           ............................................................
```

H_sapiens_model_Q9GZ

```
H_sapiens_model_Q9GZ   ............................................................
H_sapiens              ............................................................
P_troglodytes          ............................................................
M_mulatta              ............................................................
M_fascicularis         ............................................................
C_lupus                ............................................................
B_taurus               ............................................................
C_griseus              ............................................................
M_musculus             ............................................................
M_domestica            ......................................................MLACVG
G_gallus               ............................................................
A_carolinensis         ............................................................
L_chalumnae            .................................................MVLTRPTERVS
R_norvegicus           ............................................................
A_mississippiensis     ............................................................
X_maculatus            ...................................................MLPGWRNLS
O_niloticus            ...................................................MLTGCRNLS
S_formosus             ............................................................
D_rerio                ............................................................
N_vectensis            ............................................................
S_purpuratus           ............................................................
C_intestinalis         ............................................................
C_gigas                ............................................................
T_adhaerens            ............................................................
C_owczarzaki           ............................................................
A_queenslandica        ............................................................
A_pisum                ............................................................
T_urticae              ............................................................
D_discoideum           ............................................................
O_dioica               ............................................................
D_pulex                ............................................................
H_robusta              ............................................................
Z_nevadensis           EVKSVAWAKSGHL.....LATCSRDKSVWVWEVAD...DDECCDRVQRTSVTIESLNWQM
R_allomycis            TPKMGRIESVEKITP.SPLSSSSKSKSDLLFEVVTPSIEKLRQDRLKTPSPTPSSSSKQS
S_mansoni              ............................................................
H_microstoma           ............................................................
E_granulosus           ............................................................
G_theta                ............................................................
S_maritima             ............................................................
A_mexicanus            ............................................................
A_fumigatus            ............................................................
N_fumigata             ............................................................
R_mackenziei_CBS_650   ............................................................
E_mesophila            ............................................................
X_heveae               ............................................................
A_subglaciale          ............................................................
P_nodorum              ............................................................
B_bassiana             ............................................................
G_zeae                 ............................................................
N_crassa_OR74A         ............................................................
E_lata                 ............................................................
C_cerevisiae           ............................................................
S_pombe                ............................................................
S_complicata           ............................................................
Y_lipolytica           ............................................................
T_melanosporum         ............................................................
A_oligospora           ............................................................
P_carinii              ............................................................
T_deformans            ............................................................
W_ichthyophaga         ............................................................
S_punctatus            ............................................................
B_dendrobatidis        ............................................................
C_neoformans           ............................................................
A_macrogynus           ............................................................
B_saltans              ............................................................
T_cruzi                ............................................................
L_major                ............................................................
M_osmundae             ............................................................
M_lychnidis-dioicae    ............................................................
Perkinsela_sp.         ............................................................
R_irregularis          ............................................................
G_prolifera            ............................................................
B_floridae             ............................................................
F_alba                 ............................................................
T_anomala              ............................................................
U_maydis               ............................................................
S_borealis             ............................................................
M_globosa              ............................................................
P_graminis             ............................................................
B_mori                 ............................................................
N_vitripennis          ............................................................
P_humanus              ............................................................
D_melanogaster         ............................................................
A_gambiae              ............................................................
L_gigantea             ............................................................
I_scapularis           ............................................................
X_tropicalis           ............................................................
E_histolytica          ............................................................
C_viscosa              ............................................................
A_niger_YbgI           EAVFATGAGTFPGGKYRKCCFQMPGQGQFLPSEGANPAIGEVGTIETVEEMKVE......
R_microsporus          MILKAIASGVGIYSPHTACDNCINGVNDWLASGLGKGRVEPITPADNPPE..........
Chrysochromulina_sp.   IVLRCAQQSIAAHCLSSACANAPGGVADWLAKSIASGATRPIVPHAECAEAGEGRL...L
E_huxleyi              IVLGCAKHQIAVHSVHSACDAASGGVNAWLARSLARGVVRPIVKHPKVADAGQGRV...L
N_ceranae              IVTKLIKFDINVFCPHTSWDEE...MNRYLFCLLDN........................
E_cuniculi             IVIGCIKNGINVFTPHSALDPL...MNTYVYNMINSGPFFY...........KKNC....
V_culicis              FTAAIIENHISVFSPHTALDNL...MNTTLLSKLGCFGIEV...........ADSV...A
C_cinerea              SLLQCAAEGVSVYSPHTALDSVWGGINDWLARGMLGSSLSSSSSPEEGIVEPLVGLKLGA
B_hominis              IVMGCIKNDIAVYSPHTACDASKGGVNDWIVDGLGE...IASSAPITPDRE.........
C_albicans             SLIKLIQNKISVYSPHTAVDSAKGGVNDFLVEGITKNQKVESSIPIEQDKT.........
A_castellanii          ...........MLEKHSECRLEMNCGKRQLVELEKVIRSVHPFEEPAWEAYPLADIP...
consensus>50           ............................................................
```

```
H_sapiens_model_Q9GZ                                                          **********T.....
                                                   1        10
H_sapiens_model_Q9GZ  ........................................M...LSSCVRPVPTT.....
H_sapiens             ........................................M...LSSCVRPVPTT.....
P_troglodytes         ........................................M...LSSCVRPVPTT.....
M_mulatta             ........................................M...LSSCVRPVPTT.....
M_fascicularis        ........................................M...LSSCVRPVPTT.....
C_lupus               ....................MYLYHFLCFGFRYSVLPDFCM...LSSRVRLIPRT.....
B_taurus              ........................................M...LSSRVRLVATT.....
C_griseus             ..........MLYASKCRTELYINAISCVLTPDTVLPGFCM...LSS.AHLLPTS.....
M_musculus            ........................................M...LSS.AHLVPTS.....
M_domestica           LVSRKFQAPQSLFSRKFQAPQSLFSRKFQAPQSLVSRKFQAPQSLTSRKFQAPQS.....
G_gallus              ................................................MLLLCRP.....
A_carolinensis        ...........................................MQLSRLKPTPQR.....
L_chalumnae           FSVCSFEGNTALFFRRMEVSCRLYVRMAESPPGRQEKGGQWSTDLPSCFSLFMLARCLFA
R_norvegicus          ............................................MLSSAHVVPTS.....
A_mississippiensis    ............................................................
X_maculatus           WTFYFLKNRRLLFCKSSNFNT.......SALFPSASSATQTRLTSGLLLRSSVHS.....
O_niloticus           WTFFSLTSRRLWPRQSFTSRAALCSSAFSSVLHSLSSSTHSHHFLPSTQSSARLP.....
S_formosus            ......................................................M.....
D_rerio               ............................................................
N_vectensis           ............................................................
S_purpuratus          ............................................................
C_intestinalis        ............................................................
C_gigas               ............................................................
T_adhaerens           ............................................................
C_owczarzaki          ............................................................
A_queenslandica       ............................................................
A_pisum               .....................MISKSHFRCLLNRHRSFTFLKATTTRTGSH...P.....
T_urticae             ..........MLRSLIKLIPHKLSDSRSFGIFKDFSKKTLPLNNTFGFNSINKLP.....
D_discoideum          ............................................................
O_dioica              ............................................................
D_pulex               ............................................................
H_robusta             ............................................................
Z_nevadensis          I.LSFLSGNKLQQTCSVPLKCLINAWYNKYNCSYPCRNIQNSKALKFVTNQLPKDN....
R_allomycis           SSTSFSLGKNKSSKPDFVTSFAVISPSEKTRSGLSLKTSKGTLEIASIMELEKKDLRMIK
S_mansoni             ............................................................
H_microstoma          ............................................................
E_granulosus          ............................................................
G_theta               ............................................................
S_maritima            ....................................MRLLVYLQFVKLTGFHRFC.....
A_mexicanus           ............................MIPGCRIVTRRLFNLRHC.LSSLNPSC.....
A_fumigatus           ...............................MTESVTDGFGRLSPLLSLGVYLAV.....
N_fumigata            ...............................MTESVTDGFGRLSPLLSLGVYLAV.....
R_mackenziei_CBS_650  ...........................MFRHLLRAIP....RLQPQYIQNSIPIV.....
E_mesophila           ..........MLRPSLSRIISSLYSSSPKFHLRLAPPFSHSRVQPSTSLPSLPLR.....
X_heveae              ............................................................
A_subglaciale         ............................................................
P_nodorum             ............................................................
B_bassiana            ................................................MAA...A.....
G_zeae                ......................................................M.....
N_crassa_OR74A        .......MSAVFKIPIRITSRISTKPLALPYKPSILLPGY..FRRSYCQNK...N.....
E_lata                ............................................................
C_cerevisiae          ............................................................
S_pombe               ............................................................
S_complicata          .....MLNCEVRNDKDGRTMLPGLKNLFNWSMGCMLFTPVTDMRDSYAPSLPPYN.....
Y_lipolytica          ............................................................
T_melanosporum        ............................................................
A_oligospora          ............................................................
P_carinii             ............................................................
T_deformans           ............................................................
W_ichthyophaga        ............................................................
S_punctatus           .................................................MGERHH.....
B_dendrobatidis       ............................................................
C_neoformans          ............................................................
A_macrogynus          ............................................................
B_saltans             ............................................................
T_cruzi               ............................................................
L_major               ............................................................
M_osmundae            ............................................................
M_lychnidis-dioicae   ...............................................MSSTSAAS.....
Perkinsela_sp.        ............................................................
R_irregularis         ............................................................
G_prolifera           ............................................................
B_floridae            ............................................................
F_alba                ............................................................
T_anomala             ..MASLLSGIYKGGDSEATPRIPPPPGACSPSGDGGRGSGGGVGNASSTST...A.....
U_maydis              ................................................MSL...P.....
S_borealis            ............................................................
M_globosa             ............................................................
P_graminis            ............................................................
B_mori                ............................................................
N_vitripennis         ............................................................
P_humanus             ............................................................
D_melanogaster        ............................................................
A_gambiae             .......................................MLLKTCSTILFHRNIL.....
L_gigantea            ............................................................
I_scapularis          .......................................MRVLELRRLALHTSLL.....
X_tropicalis          ............................................................
E_histolytica         ............................................................
C_viscosa             ............................................................
A_niger_YbgI          ..VMCLGRSVMLQAVEALIKAHPYEEVAYEVYKMENV.......................
R_microsporus         .GQEGSGSGRLFTFSEP.TSLSEIVDRVKKLIGLPYGNEQGYV.................
Chrysochromulina_sp.  ECNEAISLSAIIARLKDILGVRHLRISLGAVVDELNIAKASDSCFIKSIAVHIGEGGELL
E_huxleyi             ECDEATPISTVVERLKALLGVRFLRLALAAVVAPEALAKAQEFLTVKTVAVQVGEGATLL
N_ceranae             .....QNVEQVFDTLKNKIGLKHIRVA.....NMSAGRKS..........LVVGVGSAFK
E_cuniculi            .GPNTSTIGNAIRILKERSGLESFRICLAREHTMESVPEF..........MHVGVGATFR
V_culicis             VGNNGNRMSVIIDRLKKTCNAKEIRLALSDIHRMDSIPPY..........VYVGVGSA.H
C_cinerea             NGESEGAEGRLVTLNQP.IEIDELVRRIKSHLKLANVQVGYPDVAEGSPSKLVQTIAICA
B_hominis             ..NPEFGIGRIATLASPYPTISQLIERMKVHFAIPHLQLATNLP.LDSP...VRKVAVCA
C_albicans            ..DSECGMGRLVKLSKP.SKLKNLVSNIKEQLRLDHVQVALSKDGIEHD...IKTIAICA
A_castellanii         ..SSTTGSGRVRVLSEA.VELRALIDRVKAMLNLKTVRLAVSPKHSLQSA..VKRVAMCA
consensus>50          ............................................................
```

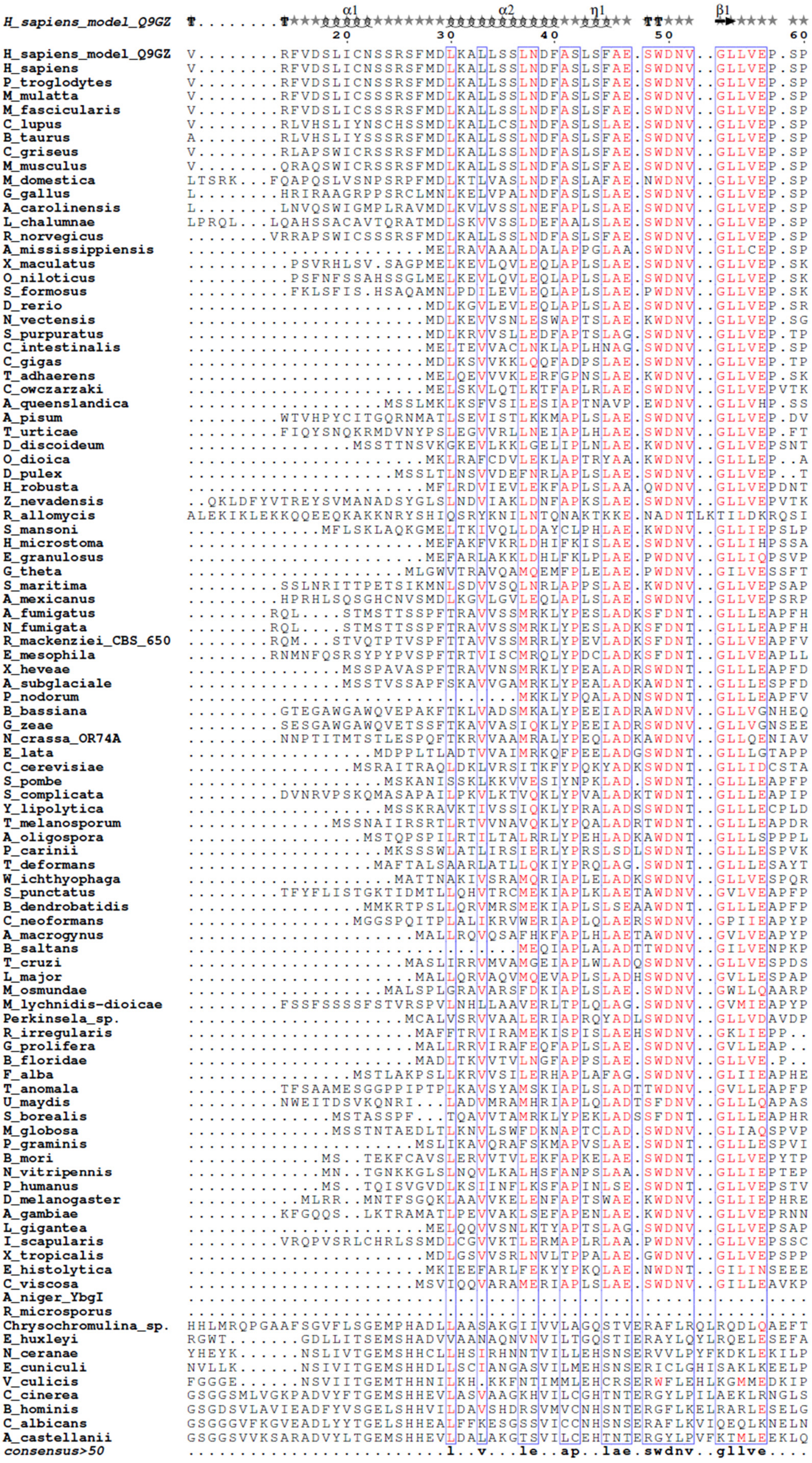
α1 α2 η1 β1
H_sapiens_model_Q9GZ
20 30 40 50 60
H_sapiens_model_Q9GZ V........RFVDSLICNSSRSFMDLKALLSSLNDFASLSFAE.SWDNV..GLLVEP.SP
H_sapiens V........RFVDSLICNSSRSFMDLKALLSSLNDFASLSFAE.SWDNV..GLLVEP.SP
P_troglodytes V........RFVDSLICNSSRSFMDLKALLSSLNDFASLSFAE.SWDNV..GLLVEP.SP
M_mulatta V........RFVDSLICSSSRSFMDLKALLSSLNDFASLSFAE.SWDNV..GLLVEP.SP
M_fascicularis V........RFVDSLICSSSRSFMDLKALLSSLNDFASLSFAE.SWDNV..GLLVEP.SP
C_lupus V........RLVHSLIYNSCHSSMDLKALLCSLNDFASLSLAE.SWDNV..GLLVEP.SP
B_taurus A........RLVHSLIYSSSRSFMDLKALLSSLNDFASLSFAE.SWDNV..GLLVEP.SP
C_griseus V........RLAPSWICRSSRSFMDLKALLSSLNDFASLSFAE.SWDNV..GLLVEP.SP
M_musculus V........QRAQSWICRSSRSFMDLKALLSSLNDFASLSFAE.SWDNV..GLLVEP.SP
M_domestica LTSRK...FQAPQSLVSNPSRPFMDLKTLVASLNDFASLAFAE.NWDNV..GLLVEP.SP
G_gallus L........HRIRAAGRPPSRCLMNLKELVPALNDFASLSLAE.SWDNV..GLLVEP.SP
A_carolinensis L........LNVQSWIGMPLRAVMDLKVLVSSLNEFAPLSLAE.SWDNV..GLLVEP.SP
L_chalumnae LPRQL...LQAHSSACAVTQRATMDLSKVVSSLDEFAALSLAE.SWDNV..GLLVEP.SP
R_norvegicus ........VRRAPSWICSSSRSFMDLKALLSSLNDFASLSFAE.SWDNV..GLLVEP.SP
A_mississippiensis ....................MELRAVAAALDALAPPGLAA.SWDNV..GLLCEP.SP
X_maculatus ..........PSVRHLSV.SAGPMELKEVLQVLEQLAPLSLAE.SWDNV..GLLVEP.SK
O_niloticus ..........PSFNFSSAHSSGLMELKEVLQVLEQLAPLSLAE.SWDNV..GLLVEP.SK
S_formosus ..........FKLSFIS.HSAQAMNLPDILEVLEQLAPLSLAE.PWDNV..GLLVEP.SK
D_rerio ....................MDLKGVLEVLEQLAPLSLAE.SWDNV..GLLVEP.SR
N_vectensis ....................MDLKEVVSNLESWAPTSLAE.KWDNV..GLLVEP.SG
S_purpuratus ....................MDLKRVVSLLEDFAPTSLAG.SWDNV..GLLVEP.TP
C_intestinalis ....................MELTEVVACLNKLAPLHNAG.SWDNV..GLLVEP.SP
C_gigas ....................MDLKSVVKKLQQFADPSLAE.SWDNV..GLLVEP.TP
T_adhaerens ....................MELQEVVVKLERFGPNSLAE.KWDNV..GLLVEP.SK
C_owczarzaki ....................MELSKVLQTLKTFAPLRLAE.SWDNV..GLLVEPVTK
A_queenslandica ................MSSLMKLKSFVSILESIAPTNAVP.EWDNV..GLLVHP.SS
A_pisum .........WTVHPYCITGQRNMATLSEVISTLKKMAPLSLAE.SWDNV..GLLVEP.DV
T_urticae .........FIQYSNQKRMDVNYPSLEGVVRLLNEIAPLHLAE.SWDNV..GLLVEP.FT
D_discoideum ...............MSSTTNSVKGKEVLKKLGELIPLNLAE.KWDNV..GLLVEPSNT
O_dioica ....................MKLRAFCDVLEKLAPTRYAA.KWDNV..GLLLEP..A
D_pulex .................MSSLTLNSVVDEFNRLAPLSLAE.SWDNV..GLLVEP..T
H_robusta ....................MFLRDVIEVLEKFAPLSLAA.QWDNV..GLLVEPDNT
Z_nevadensis ..QKLDFYVTREYSVMANADSYGLSLNDVIAKLDNFAPKSLAE.SWDNV..GLLVEPVTK
R_allomycis ALEKIKLEKKQQEEQKAKKNRYSHIQSRYKNILNTQNAKTKKE.NADNTLKTILDKRQSI
S_mansoni .............MFLSKLAQKGMELTKIVQLLDAYCLPHLAE.KWDNV..GLLIEPSLP
H_microstoma ....................MEFAKFVKRLDHIFKISLAE.SWDNV..GLLIHPSSA
E_granulosus ....................MEFARLAKKLDHLFKLPLAE.PWDNV..GLLIQPSVP
G_theta ...................MLGWVTRAVQAMQEMFPLELAE.PWDNV..GILVESSFT
S_maritima .........SSLNRITTPETSIKMNLSDVVSQLNRLAPPSLAE.KWDNV..GLLVEPSAP
A_mexicanus .........HPRHLSQSGHCNVSMDLKGVLGVLEQLAPLSLAE.SWDNV..GLLVEPSRP
A_fumigatus ........RQL....STMSTTSSPFTRAVVSSMRKLYPESLADKSFDNT..GLLLEAPFH
N_fumigata ........RQL....STMSTTSSPFTRAVVSSMRKLYPESLADKSFDNT..GLLLEAPFH
R_mackenziei_CBS_650 ........RQM...STVQTPTVSPFTTAVVSSMRRLYPEVLADKSFDNT..GLLVEAPFV
E_mesophila ........RNMNFQSRSYPYPVSPFTRTVISCMRQLYPDCLADKSFDNT..GLLVEAPLL
X_heveae ..............MSSPAVASPFTRAVVNSMRKLYPEALADRSWDNT..GLLLEAPFD
A_subglaciale ..............MSSTVSSAPFSKAVVGAMRKLYPEALADKAWDNT..GLLLESPFD
P_nodorum .........................MKKLYPQALADNSWDNT..GLLLEAPFV
B_bassiana .........GTEGAWGAWQVEPAKFTKLVADSMKALYPEEIADRAWDNV..GLLVGNHEQ
G_zeae .........SESGAWGAWQVETSSFTKAVVASIQKLYPEEIADRSWDNV..GLLVGNSEE
N_crassa_OR74A .........NNPTITMTSTLESPQFTKRVVAAMRALYPEQLADKAWDNV..GLLQENIAV
E_lata .................MDPPLTLADTVVAIMRKQFPEELADGSWDNT..GLLLGTAPP
C_cerevisiae ...............MSRAITRAQLDKLVRSITKFYPQKYADKSWDNT..GLLIDCSTA
S_pombe .................MSKANISSKLKKVVESIYNPKLAD.SWDNT..GLLLEAPFP
S_complicata .........DVNRVPSKQMASAPAILPKVLKTVQKLYPVALADKTWDNT..GLLLEAPIP
Y_lipolytica .................MSSKRAVKTIVSSIQKLYPRALADSSWDNT..GLLLECPLD
T_melanosporum ..............MSSNAIIRSRTLRTVVNAVQKLYPQALADRTWDNT..GLLLEAPDR
A_oligospora ................MSTQPSPILRTILTALRRLYPEHLADKAWDNT..GLLLSPPPL
P_carinii ..................MKSSSWLATLIRSIERLYPRSLSDLSWDNT..GLLLESPVK
T_deformans .................MAFTALSAARLATLLQKIYPRQLAG.SWDNT..GLLLESAYT
W_ichthyophaga ..................MATTNAKIVSRAMQRIAPLELADKSWDNV..GLLVESPQR
S_punctatus .........TFYFLISTGKTIDMTLLQHVTRCMEKIAPLKLAETAWDNV..GVLVEAPFP
B_dendrobatidis .................MMKRTPSLLQRVMRSMEKIAPLSLSEAAWDNT..GLLLEAPFP
C_neoformans .................MGGSPQITPLALIKRVWERIAPLQLAERSWDNV..GPIIEAPYP
A_macrogynus .....................MALLRQVQSAFHKFAPLHLAETAWDNV..GVLVEAPYP
B_saltans ..........................MEQIAPLALADTTWDNV..GILVENPKP
T_cruzi ...................MASLIRRVMVAMGEIAPLWLADQSWDNV..GLLVESPDS
L_major .....................MALLQRVAQVMQEVAPLSLADHSWDNV..GVLLESPAP
M_osmundae ..................MALSPLGRAVARSFDKIAPLSLAE.SWDNV..GWLLQAARP
M_lychnidis-dioicae .........FSSFSSSSFSTVRSPVLNHLLAAVERLTPLQLAG.SWDNV..GVMIEAPYP
Perkinsela_sp. ...................MCALVSRVVAALERIAPRQYADLSWDNV..GLLVDAVDP
R_irregularis ....................MAFFTRVIRAMEKISPISLAEHSWDNV..GKLIEPP..
G_prolifera .....................MALLRRVIRAFEQFAPLSLAE.SWDNV..GVLLEAP..
B_floridae .....................MADLTKVVTVLNGFAPPSLAE.SWDNV..GLLVE.P..
F_alba .................MSTLAKPSLLKRVVSILERHAPLAFAG.SWDNV..GLIIEAPHE
T_anomala .........TFSAAMESGGPPIPTPLKAVSYAMSKIAPLSLADTTWDNV..GVLLEAPFP
U_maydis .........NWEITDSVKQNRI...LADVMRAMHRIAPLQLADTSFDNV..GLLQAPAS
S_borealis ..............MSTASSPF...TQAVVTAMRKLYPEKLADSSFDNT..GLLLEAPHR
M_globosa ..............MSSTNTAEDLTLKNVLSWFDKNAPTCLAD.SWDNV..GLIAQSPVP
P_graminis ......................MSLIKAVQRAFSKMAPVSLAE.SWDNT..GLLLESPVI
B_mori .............MS..TEKFCAVSLERVVTVLEKFAPKELAE.SWDNT..GLLVEPYTP
N_vitripennis .............MN..TGNKKGLSLNQVLKALHSFANPSLAA.SWDNV..GLLIEPTEP
P_humanus .............MS..TQISVGVDLKSIINFLKSFAPINLSE.SWDNT..GLLVEPSTV
D_melanogaster ...........MLRR..MNTFSGQKLAAVVKELENFAPTSWAE.KWDNV..GLLIEPHRE
A_gambiae .........KFGQQS..LKTRAMATLPEVVAKLSEFAPENLAE.KWDNV..GLLVEPRNN
L_gigantea .....................MELQQVVSNLKTYAPTSLAG.SWDNV..GLLVEPSAP
I_scapularis .........VRQPVSRLCHRLSSMDLCGVVKTLERMAPLRLAA.PWDNV..GLLVEPSSC
X_tropicalis .....................MDLGSVVSRLNVLTPPALAE.GWDNV..GLLVEPSPP
E_histolytica .....................MKIEEFARLFEKYYPKQLAE.NWDNT..GILINSEEE
C_viscosa ...................MSVIQQVARAMERIAPLSLAE.SWDNV..GILLEAVKP
A_niger_YbgI ....
R_microsporus ....
Chrysochromulina_sp. HHLMRQPGAAFSGVFLSGEMPHADLLAASAKGIIVVLAGQSTVERAFLRQLRQDLQAEFT
E_huxleyi RGWT.......GDLLITSEMSHADVVAANAQNVNVILTGQSTIERAYLQYLRQELESEFA
N_ceranae YHEYK......NSLIVTGEMSHHCLLHSIRHNNTVILLEHSNSERVVLPYFKDKLEKILP
E_cuniculi NVLLK......NSIVITGEMSHHDLLSCIANGASVILMEHSNSERICLGHISAKLKEELP
V_culicis FGGGE......NSVIITGEMTHHNILKH.KKFNTIMMLEHCRSERWFLEHLKGMMEDKIP
C_cinerea GSGGSMLVGKPADVYFTGEMSHHEVLASVAAGKHVILCGHTNTERGYLPILAEKLRNGLS
B_hominis GSGDSVLAVIEADFYVSGELSHHVILDAVSHDRSVMVCNHSNTERGFLKELRARLESELG
C_albicans GSGGGVFKGVEADLYYTGELSHHEALFFKESGSSVICCNHSNSERAFLKVIQEQLKNELN
A_castellanii GSGGSVVKSARADVYLTGEMSHHEVLDALAKGTSVILCEHTNTERGYLPVFKTMLEEKLQ
consensus>50 ....l..v...le..ap..lae.swdnv..gllve....

```
                                          β2              α3          β3
H_sapiens_model_Q9GZ   **           ***-..---->*****QQQQQQQQQQ.....Q****--->**
                                          70          80          90
H_sapiens_model_Q9GZ   PH..........TVNT..LFLTNDLTEEVMEEVLQ.....KKADLILSYH..........
H_sapiens              PH..........TVNT..LFLTNDLTEEVMEEVLQ.....KKADLILSYH..........
P_troglodytes          PH..........TVNT..LFLTNDLTEEVMEEVLQ.....KKADLILSYH..........
M_mulatta              PH..........TVNT..LFLTNDLTEEVMEEGLQ.....KKADLILSYH..........
M_fascicularis         PH..........TVNT..LFLANDLTEEVMEEGLQ.....KKADLILSYH..........
C_lupus                PH..........TVNT..LFLTNDLTEEVMEEALQ.....KKADLILSYH..........
B_taurus               PH..........TVNT..LFLTNDLTEEVMEEALQ.....KKADLILSYH..........
C_griseus              PH..........TVNT..LFLTNDLTEEVMEEALQ.....KKADLILSYH..........
M_musculus             PH..........TVNT..LFLTNDLTEEVMDEALQ.....KKADFILSYH..........
M_domestica            PH..........TVKT..LLLTNDLTEEVMEEALQ.....RKADLILSYH..........
G_gallus               PH..........AVRT..LLLTNDLTEPVMEEAVQ.....KNADLILSYH..........
A_carolinensis         PH..........TVST..LFLTNDLTEEVMEEALQ.....KKANLILSYH..........
L_chalumnae            PH..........TVRT..LFLTNDLTEDVMGEALA.....QRADLILAYH..........
R_norvegicus           PH..........TVNT..LFLTNDLTEEVMEEALQ.....KKADLILSYH..........
A_mississippiensis     PH..........AVRT..LLLTTDLTEPVLDEALA.....RGAELVLAYH..........
X_maculatus            SR..........PIKT..VLLTNDLTDGVMEEAEA.....LSCDLIVSYH..........
O_niloticus            PR..........PVKT..ILLTNDLTDAVMEEAVA.....VSCDLIISYH..........
S_formosus             PR..........LVQK..ILLTNDLTVSVMEEAEV.....MSCDLIISYH..........
D_rerio                PR..........PIKT..ILLTNDLTAAVMDEAVG.....MKCDLIVSYH..........
N_vectensis            QH..........NVRS..ILLTNDLTEKVLQEAIE.....KRANMIISYH..........
S_purpuratus           PH..........TVST..ICLTNDLTEEVLDEAIE.....KQTDLILSYH..........
C_intestinalis         PH..........TVSR..IMLTNDLTETVLQEGIS.....ANVNMIVSYH..........
C_gigas                PH..........EVKK..VMLTNDLTEAVYREACD.....WNANLIISYH..........
T_adhaerens            PH..........LVRK..LMITNDLTEAVLKESIA.....EKADMILSYH..........
C_owczarzaki           RR..........PIDK..ILLTIDLTERVVDEAAQ.....HSIPLIIAYH..........
A_queenslandica        AS..........NVSH..IFMTIDLTEEVLEEALECSQSGEKVDMIISYH..........
A_pisum                QT..........SVSR..VFLTNDLTEDVLEEAVS.....VDANLIVSYH..........
T_urticae              QI..........SIGK..IVLTIDLTEKVIEEAKT.....YGTELIISYH..........
D_discoideum           DSM.........DIER..IFLTNDLTEPVLQEAIN.....EKASFIISYH..........
O_dioica               DC..........FVER..VLLTNDLTSRVMKEAVD.....FKANIIISYH..........
D_pulex                SSK.........IIKK..ILLTNDLTNSVMQEAES.....SSVDLIYSYH..........
H_robusta              SAD.........FIKK..IMLTNDLTLNVMKECID.....KEINLIISYH..........
Z_nevadensis           KN...........VRK..ILLTNDLTEDVMEEAVN.....LCTDLIISYH..........
R_allomycis            PD...........LLE..SSETNKKLAEIEGKLYE...NQISKDAWINLK..........
S_mansoni              HH...........VDR..IFITNDLTEQVLDEAIS.....QKCGLIVSYH..........
H_microstoma           TT...........IKR..IFLTNDLTEPVLNEAIS.....NGADFIISYH..........
E_granulosus           TF...........VSR..ICLTNDLTEPVLNEVTH.....IGANFIISYH..........
G_theta                EEE.........IGESGAILFTNDLTEKVLEEALS.....AGAKLIITYH..........
S_maritima             HH...........VEK..MLLTNDLTENVLKEALD.....LKVNLILSYH..........
A_mexicanus            RP...........IKT..ILLTNDLTSAVMDEAEE.....MACDLVISYH..........
A_fumigatus            P....A......RRQKNSVLLTIDLTKAVADEAIK.....RKDSVVVAYH..........
N_fumigata             P....A......RRQKNSVLLTIDLTKAVADEAIK.....RKDSVVVAYH..........
R_mackenziei_CBS_650   A...SR......PRNRNSVLLTVDLTRAVADEAIE.....NKHSVVVAYH..........
E_mesophila            PTHNSR......PRNTNKVLLTIDLTRAVATEAID.....HNVSLVVSYH..........
X_heveae               P....A......RKQANSVLLTVDLTRAVADEAIE.....RKDSIIIAYH..........
A_subglaciale          P....I......RRQMNSALLTIDLTKAVADEAIE.....RGDSVIVAYH..........
P_nodorum              A....S......RRQSNNVLLTIDLTKAVADEAIA.....LNSSIIIAYH..........
B_bassiana             ESS......QKR....PIVLVTNDLTYQVAMDAIR...QGASV..IVSYH..........
G_zeae                 DTK......KKK....R.VLVTNDLTYQVAMDAIE...QDVSV..IVSYH..........
N_crassa_OR74A         AGQ......DVP....QTVLLTNDLTVAVAEEAIR...KRASV..IVSYH..........
E_lata                 SSS......SL...................VVDEALK...ENTRI..IICYH..........
C_cerevisiae           QVT......TADANAKTKVLLTVDLTKSVAQEAVD...ANCNV..IVAYH..........
S_pombe                RTN......ASS......VLLTIDLTEKVAEEAIS....NKLVSSIVAYH..........
S_complicata           HRP......AAN.GV.SRVLLTIDLTTAVAEEALR...EGSGVAVIVAYH..........
Y_lipolytica           TEP......EKE.KL.N.VLLTIDLTTSVVDEAIR...NNSGV..VMAYH..........
T_melanosporum         TDR......SSP.EA.HNVLLTIDLTKAVVDEAIS...LRSSV..IVAYH..........
A_oligospora           TLS......SSTLRA.PRILLTVDLTTAVATEAIS...LNAAM..VVTYH..........
P_carinii              NPD......RK......RVLLAIDLTTAVAEEALT...MYPPVGAIVAYRECFESIYSGY
T_deformans            RVA......TAQSSL.TKILLCIDLTTSVCDEAIN...LD.GCSHIISYH..........
W_ichthyophaga         NGS......GQH......VVIVNDLTEKVFDRIIS...DNPFVSVVVSYH..........
S_punctatus            RTN.........A...TRIFLTIDLRQHVLEEALK....DPKIGVIVAYH..........
B_dendrobatidis        RPN.........A...SKVFLTIDLTQEVLEEAIQ....DPAVGVIVAYH..........
C_neoformans           NPS.........H...RQVLLTIDLTPSVCAEALK....HPSLSLIVSYH..........
A_macrogynus           RQE.........A...DTVFLTIDLTPQVLEEALQ....LGNVGVIVAYH..........
B_saltans              N.A.........Y...SRVMLTIDLTPAHLQECIE....K.QIGVIVSYH..........
T_cruzi                N.N.........S...GIVLLTVDLTPEVMEECLR....H.NVEVIVAYH..........
L_major                N.K.........S...NAIMLTIDLTPEVMEECLE....K.NVEVILAYH..........
M_osmundae             RTG......RPGR...TVVLT.IDLTTAVYRNL......PEDTGIVVAYH..........
M_lychnidis-dioicae    RQE......IKGK...KQVLTCIDLTTAVTEEALA....SSSTAAVLTYH..........
Perkinsela_sp.         PAH......T..Q...NRVGLALDLTTLVVQECVD....K.SVGVLVCYH..........
R_irregularis          ..........YPRHFPSRVLLTIDLTPLVLEEALA...DKQ.IGVIIAYH..........
G_prolifera            ..........FSNADATSVLLTIDLTTPVLEEALA...IRPPVGVIVSYH..........
B_floridae             ..........SPPHEVSRMLLTNDLTEGVLAEAVH...KK..ANFILSYH..........
F_alba                 TLDFLPPRPVSANATHECILLTNDLTLPVVREAAA...LG..ASVIVAYH..........
T_anomala              .............RRSKGVFLAIDLTPAVADELLS..DANSSISVAIIYH..........
U_maydis               T........TSQSSEQQSVMLAIDLTTDVCNEILS...SSANVKVALVYH..........
S_borealis             ...........ENYLKDSVLLTIDLTKGVADEAIR...RKDSIIVT..YH..........
M_globosa              ..........KEDDKARGLFLAIDLTPAVAEELLA...KP.DVRVAIVYH..........
P_graminis             ..........RP..LANKVLLTIDLTPSVAEEALA...KDSNVGVIVAYH..........
B_mori                 RQ.............ITKILLTNDLTEDVALEAEI....L.ACEMIISYH..........
N_vitripennis          KN.............ISHILLTNDLTEDVMQEAVE....L.QVDMIISYH..........
P_humanus              TP.............VKCVLLTNDLTEDVLKEALE....F.KTGLIISYH..........
D_melanogaster         KQ.............IKKILLTNDLTEPVVKEALE....K.EAELIISYH..........
A_gambiae              KS............SITNILLTIDLTEAVVAEARE....T.GAQMIISYH..........
L_gigantea             HI.............VKTLFLTNDLTPPVLDEAIE....K.KADMILSYH..........
I_scapularis           R..............VRTVLLTNDLIPEVVDEAVS....L.GADLIVSYH..........
X_tropicalis           HQ.............VHKLLLTNDLTEDVLDEAID....T.GANMILSYH..........
E_histolytica          ...............FNLVLVCIDITESVIDEAIN....K.KCKAILSYH..........
C_viscosa              LT.............SQRVLLTIDLTPPVLASALS..HPN.PPSTIISYH..........
A_niger_YbgI           ............................................................
R_microsporus          ............................................................
Chrysochromulina_sp.   DADWNVKIKCSETDCHTLTVV.......................................
E_huxleyi              DSDWNVLFKCS.TDCNPLSVV.......................................
N_ceranae              ECDIFI....SKNDKDPIDIL.......................................
E_cuniculi             EYEIFV....SKNDKDPVTIL.......................................
V_culicis              GVQVIV....SENDASPISFV.......................................
C_cinerea              EDQEFGE...AVEVLVSRGYTLRYSNIRIHNHAMSTRCRQSNQTTDRCRLA.........
B_hominis              EEFTFVV...SQTDRDPLSVFCFVCSTPVIRLIVYLFVDVSS..................
C_albicans             EADQVVI...SETDKDPFETW.......................................
A_castellanii          GKIDVHI...ATTDADPLTVV.......................................
consensus>50           ..................vllt.dlt..vmeea............ivsyh..........
```

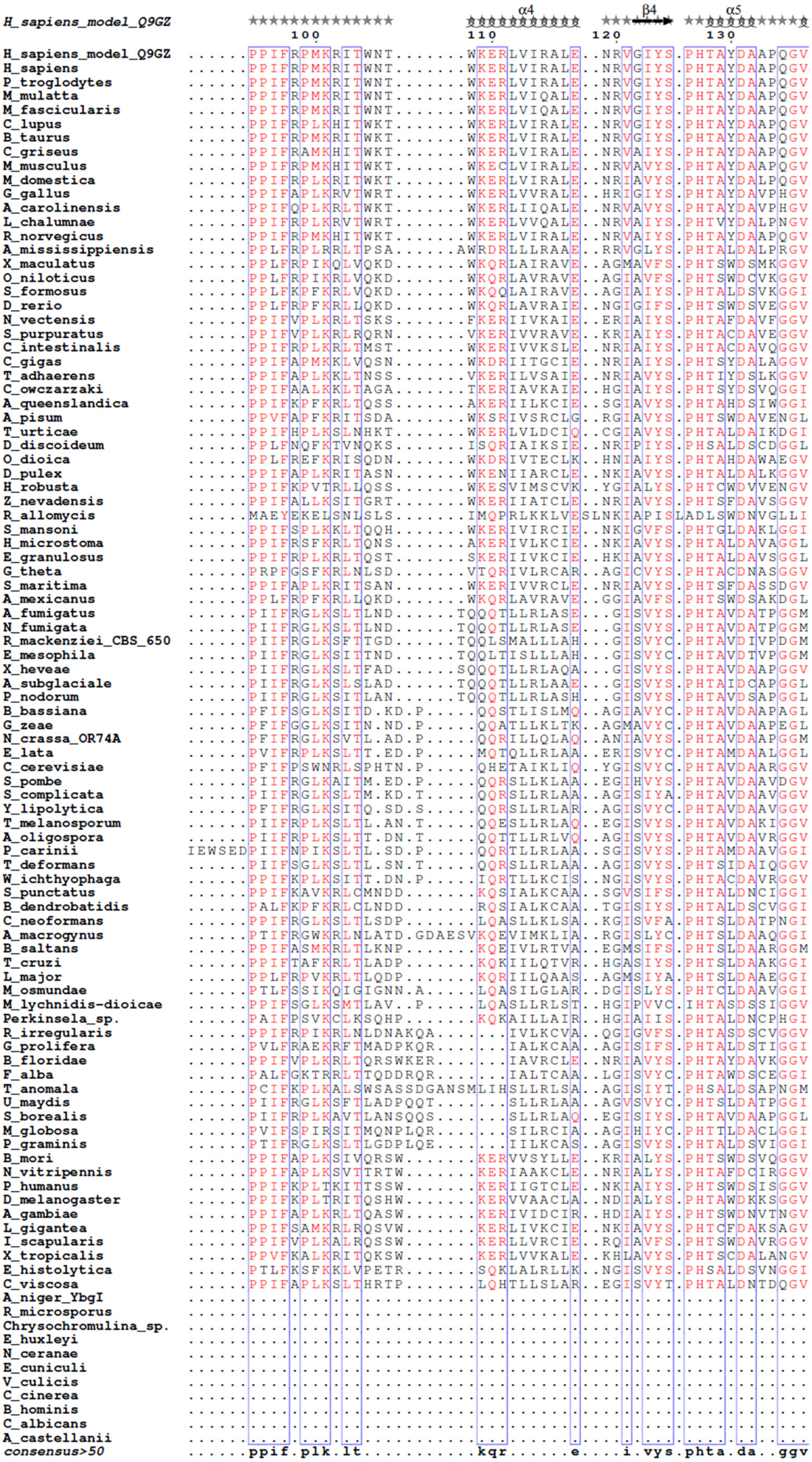
H_sapiens_model_Q9GZ
α4
β4
α5
100
110
120
130
consensus>50
.....ppif.plk.lt..........kqr......e....i.vys.phta.da..ggv

```
                          α6         β5                         α7                        β6
H_sapiens_model_Q9GZ
                         140          150            160          170            180

H_sapiens_model_Q9GZ    NNWLAKGL.GACTSRPIHPSK....APNYPTEGNHRVEFNVNYTQ....DLDKVMS...A
H_sapiens               NNWLAKGL.GACTSRPIHPSK....APNYPTEGNHRVEFNVNYTQ....DLDKVMS...A
P_troglodytes           NNWLAKGL.GACTSRPIHPSK....AANYPTEGNHRVEFNVNYTQ....DLDKVMS...A
M_mulatta               NNWLAKGL.GACTSRPIHPSK....APNYPTEGNHRVEFNVSHTQ....ALDKVMS...A
M_fascicularis          NNWLAKGL.GACTSRPIHPSK....APNYPTEGNHRVEFNVSHTQ....ALDKVMS...A
C_lupus                 NSWLAKGL.GVCTSRPIHPSR....APNYPTTGTHRVEFSVNGTE....DLDKVIS...A
B_taurus                NNWLAKGL.GVCTSRPIHPSK....APDYPTEGTHRVEFSVTHTQ....DPDKVIS...A
C_griseus               NNWLAKGL.GTCSTRPIHPSR....APNYPTEGAHRLEFSVNHTQ....DLDKVMS...A
M_musculus              NSWLAKGL.GTCTTRPIHPSR....APDYPTEGAHRLEFSVNRSQ....DLDKVMS...T
M_domestica             NNWLAKGI.GVCTSLPIHPSK....APNCPTTGTHRVEFNINDTP....SMDKFMS...A
G_gallus                NNWLSKGL.GTCTSVPLHPST....APSHPTEGTHRVEFCADTTE....HLEIVLS...K
A_carolinensis          NNWLAKGLEGPCTSVPLQPAS....ALDYPSEGSHRVEFNACSSE....NLETVLS...S
L_chalumnae             NKWLAKGL.GVCSTEPIQLST....ASAYPSLGSHRIEFSVHQSQ....DLDGILS...S
R_norvegicus            NSWLAKGL.GTCTTRPIHPSR....APNYPTEGTHRLEFSANHSQ....DLDKVMS...A
A_mississippiensis      NAWLAAALGASTSSVPIQPST....ALTYPTEGSHRVEFIVGQAE....QLEAVLS...K
X_maculatus             NDWLVGGLGSGQVSVLSQALG....GASH....SHKLEFMVRSPE....ELNAVVE...E
O_niloticus             NDWLVGGLGSGQVSVLSQVLG....AASH....SHKLEFTVRSAE....ELNMIME...E
S_formosus              NDWLIGGVGSGSVSVLRQAVS....SGPL....CHKLEFTVRNHE....ELETVLS...E
D_rerio                 NDWLVGGMGRGKVSVLSQAVS....SGAQ....KHRIEFSCMDDG....EVNSLLE...Q
N_vectensis             NDWLAKGLGRGSVDPITYSME....ACVKGC..DYNVTAIAPSTE....AAEKINS...Q
S_purpuratus            NDWLVEGLGDTSSNKPLEPCL....DVKNGD..DFSICVMAPQTD....V.ETVEA...T
C_intestinalis          NDWLASGLGEMKSIKPIIRSN....IKAD....NYSVSIAIQSSN....DKDLLDS...F
C_gigas                 NDWLLQAF.DISSSKPVTQSV....TSAGPY..NKKLQLTISST.....DRESILK...C
T_adhaerens             TDWLASCL.GEYTTQPVIPFF....DESASGYYKLEMYISKDKQH....NWSEVLN...N
C_owczarzaki            NDWLASGLGEASAISVLQPHTFHHDGDSQTSSGTHRASV.YGLAG....QLAELRT...A
A_queenslandica         NDWILKAFNQGTVHPISYNQS....PDKQPIELLVSSFSNKTEAN....EVAKSII...A
A_pisum                 TDWLISCFDGTVSTIQ.........PLKINPEFSHVVTLSATLSD....NTLLGGK...E
T_urticae               NDWLLSPFN..IKEVR.........PINQSSMDSRS.TNHYSYRL....ETVLPSE...S
D_discoideum            NDWISNALIKLNSGKGRSKP.....ITPYQESLKSTQKISIYLHT....NQPLTAE...I
O_dioica                NAWLIEGIIGHSN.....HSP............ISPTIDSRETYE....ISGKVAE...S
D_pulex                 NDWLAEAFEGKKL.....VRS............ILELVASVPEAR....LEEHIST...A
H_robusta               NDWLLTPFKGKKQCLKARERK............VYRFTVELPETK....LSDDALE...S
Z_nevadensis            NDWLANGFETESVRPLIQSDS...ADIGSKLYQVEITGFTESALD....QIKKDVE...R
R_allomycis             ESPVL..VNTGVSKVLVTNDL...TLEVCQEAIRKTANFIVTYHP....VIFSPMK...R
S_mansoni               NDWLLEPLAVNRRESL..................SRSPITQTLSR....LTVVMNE...N
H_microstoma            NDWLLSPFDLKHKKPL..................NGTKLSRYPPT....VTSLNNR...T
E_granulosus            NDWLLSPF.........................GNKLLDRPPT....VTTRHDR...R
G_theta                 NDWLAQAFSSDPQSISP................VKPMKIEGNVNS....TMRLEAP...V
S_maritima              NSWLTEAFDVADCKPISPSI.....STSTPSGFSHKVEALIPSSVANNPEVNKVLEKLEL
A_mexicanus             NDWLIGGLGHGQVSVLSQAL.....CSAPQR....HRVEFTAGSQV....ELDSILK...L
A_fumigatus             GDWLCDVVTGAI.............................APSTDSSPPLS
N_fumigata              GDWLCDVVTGAI.............................APSTDSSPPLS
R_mackenziei_CBS_650    ADWLCDIVTGKMDAPEPEA.........DSRSSTKPTSLFAEGLDQDKDALSDPFTTPTA
E_mesophila             ADWLCDVVTGRVDDPESEAFTAQTPSDTEISSDTEVTTSDSDHGYDEGDTLSDASTKPTS
X_heveae                NDWLADIVTGQLIGEEETP.......DEDEIAESVSESDVPDQNEKTADQVSTPRTPEEQ
A_subglaciale           GDWLADIVSGTPMSAEELA...................AQAEEKQKEQAKEPETPSQG
P_nodorum               GDWLADIVTGTKKALSEDD.....................SEAGSSQQGDKPDADDPFIE
B_bassiana              NTWLADAVAGPH.A.............................................
G_zeae                  NTWLADIVSGPH.E.............................................
N_crassa_OR74A          NDWLADMLDGHGVE.............................................
E_lata                  NDWLCDLLT.QGVD.............................................
C_cerevisiae            NDWLVRGLNNGE.N.............................................
S_pombe                 NDWLAQGIAGGR.N.............................................
S_complicata            NDWLADGVSGGK.E.............................................
Y_lipolytica            NDFLIDVVSLGK.E.............................................
T_melanosporum          NDWLADGISGGR.G.............................................
A_oligospora            NDWLASGISGGE.E.............................................
P_carinii               NDWLADGIWDNK.C.............................................
T_deformans             NGMLSDCFRSTG.E.............................................
W_ichthyophaga          NDWMFTAITGHKTS.............................................
S_punctatus             NDWLARG...LGVG.............................................
B_dendrobatidis         NDWLSRG...LGKG.............................................
C_neoformans            NTWLIKPF..IPIS.............................................
A_macrogynus            NDWLAKGL..EPFG.............................................
B_saltans               NDWLASIV..DSQS.............................................
T_cruzi                 NDWLASIV..DSSG.............................................
L_major                 NDWLASLV..AGDG.............................................
M_osmundae              NTWLASAF..DNVS.............................................
M_lychnidis-dioicae     NDHIASAFTLDLRA.............................................
Perkinsela_sp.          NSWIAKGILKGSHH.............................................
R_irregularis           NDWLAKGLGAGTIK.............................................
G_prolifera             NDHLASIFP..NVK.............................................
B_floridae              NDWLASGLDSSGDC.............................................
F_alba                  NDWLASIVGRASSQ.............................................
T_anomala               NDWLAFVVSS.....................................................
U_maydis                NDWLGKVVTSRSAF.............................................
S_borealis              NDWLADIVTNR....................................................
M_globosa               NDWLVTGLEHAWES.............................................
P_graminis              NDELCEIV.....S.............................................
B_mori                  NDWLAS.AFS.VAE.............................................
N_vitripennis           NDWLAG.AFK.HKE.............................................
P_humanus               NDWLAK.GFD.VET.............................................
D_melanogaster          NDWLSK.AVN.IIS.............................................
A_gambiae               NDWLAD.SLP.HAD.............................................
L_gigantea              NDWLIS.AFE.LSN.............................................
I_scapularis            NDWLAS.CFDGAAD.............................................
X_tropicalis            NDWLAR.ALG.PSK.............................................
E_histolytica           NDWLASLVLNEPVS.............................................
C_viscosa               NWWLAQ.......A.............................................
A_niger_YbgI            .....................................................
R_microsporus           .....................................................
Chrysochromulina_sp.    .....................................................
E_huxleyi               .....................................................
N_ceranae               .....................................................
E_cuniculi              .....................................................
V_culicis               .....................................................
C_cinerea               .....................................................
B_hominis               .....................................................
C_albicans              .....................................................
A_castellanii           .....................................................
consensus>50            ndwla.......................................................
```

```
H_sapiens_model_Q9GZ                            β7                β8     β9             α8
                                                190       200       210       220

H_sapiens_model_Q9GZ   VK....................GIDGVS.VTSFSARTGNEEQTRINLNCTQKALMQVVDF
H_sapiens              VK....................GIDGVS.VTSFSARTGNEEQTRINLNCTQKALMQVVDF
P_troglodytes          VK....................GIDGVS.VTSFSARTGNEEQTRINLNCTQKALMQVVDF
M_mulatta              VK....................GIGDVS.VTSFSARFDNEEQTRISLNCTQKALMQVVDF
M_fascicularis         VK....................GIGDVS.VTSFSARFDNEEQTRISLNCTQKALMQVVDF
C_lupus                VK....................RIADVS.VTSFSTRTDNEEQTRISLNCSQEALIQVVAF
B_taurus               LK....................EIAGVS.VTSFSARTDDEEQTRLSLNCTQQALMQVVAF
C_griseus              VK....................GIGDVS.VTSFAARCDDEEQTRISLNCTQKALMQVLAF
M_musculus             LR....................GVGGVS.VTSFPARCDGEEQTRISLNCTQKTLMQVLAF
M_domestica            VK....................EIPEIS.ITTLSARNDGEEQVRVSLNCTQKSLLRVVAF
G_gallus               IK....................DIPEVFCLTTLPVRVDGEEQTRVSLNCSQKALLEVVAL
A_carolinensis         MH....................KLPGVSFITTLPIRAEGDEQTRVSLNCTQQALVQVMAL
L_chalumnae            VW....................KIQGIS.VSTLPVRCDGEERTRVCLNTTQQALPEVMAL
R_norvegicus           VK....................GVGGVS.VTSFPARCDGEEQTRVSLNCTQKALMQVLAF
A_mississippiensis     VQ....................TIPEVSSVVTLPARAEGEDQTRVSLNCSEKAMQDVVAL
X_maculatus            LK....................ANDDGTALQCSGTRPDSSSI.HVSLTCSASALTSSVQI
O_niloticus            LK....................ACDSGTTLQHSGSRPDSSGI.HVSVTCCDSALTPSVQA
S_formosus             L......................GDRGTGVTFSAARPDSHAY.SVRVSCCDSALTSVVQT
D_rerio                LR....................HTEGSETFQCTRIKSESGGQ.QVNLTCRSSALTGAVQI
N_vectensis            IN....................SLGGIGSNSIGKTERFGDPALPKIVTHCSGDTLSAVMQ
S_purpuratus           LQ....................KMSGAHNVTCIVTNNSNYQITCSCKKSDLGNILSLV.Q
C_intestinalis         EQ....................NLSQI....VKFVRRTNVDAIEYECQCAKSKMKDIVD.
C_gigas                VS....................GIEN....TFTTVNELNKEFCEINILCGSGTIPAILES
T_adhaerens            YN....................KENSVQDIIVSDL.VDPKGCVKYGITCSRSGMFQILDS
C_owczarzaki           IA....................SILGSESSFATFSSNDSASRSKLEFKCQQSHLGSLAAL
A_queenslandica        SG....................NVTGDNMEILVTENLSGISSYKLKCSVTETAVKPLMPM
A_pisum                IR....................TIVESSDFQADKHFKIVGYNDSQLQLLCNSSSLVTLNK
T_urticae              DS....................KLLE....RVEKLGKVLRLPSSAVIFSEDKNLPLIINI
D_discoideum           LK....................QLE.......LNSNFQFYSTDKIELSCDQQQLLILINL
O_dioica               ........................VNRKSFRKHFNKTKISDSGDISGRIFKRDLPKLSEK
D_pulex                ........................DGYEQMTIMAPEKALPSIIELVNQC..........S
H_robusta              GV....................LSDIWEIYGPISHIKLPNDKKAFTFVCDEVDLNDAKAK
Z_nevadensis           NT....................RGAQE.ELVGDVSVSCGEMKLTISCSKSSVPLLAXXXX
R_allomycis            IV....................LGHSACQTITSLCLANNISVFSPHTSLDSVKHGVNDWL
S_mansoni              FG....................DFVISTGVGVCATNIKSNAGITAIVTCTESDLKRVVDV
H_microstoma           LQ....................EYCIENNVAPLNSDIPSLDGQMSEVTVTGDAPK.....
E_granulosus           FQ....................EYCIRNNIAPINCKAASSNGKLSEAIMSGDALK.....
G_theta                VT....................TVPRLKPVTPSIPSLVLQDAFEAMVAGYPGAGSGRIVQ
S_maritima             IM....................QRPESQNEMEVNFRSTELCPCILTVLCTEAKLPEIAAV
A_mexicanus            LK....................ETENSEAFQYTLLRNEEGGQQVILTCPGSALTPVVQTV
A_fumigatus            AS............................TSQQYSQPTYPQPRPASITPSSAAPHVRST
N_fumigata             AS............................TSQQYSQPTYPQPRPASITPSSAAPHVRST
R_mackenziei_CBS_650   AT...................KSKHPRPPAPHRTYSKPTYL..RPTSSFHAVVVPHTRSV
E_mesophila            HSDAVSDPYVSSPGPQSPNTPKAKSMRPQTTRRTYSKPTYP..RPTSHFHPAVISHTRSV
X_heveae               AD...................TDEGQLSVQPIPKYSDPTYP..EP.........PQEKAI
A_subglaciale          L......................KRPTAQLQHHPSQLSIKDRTLKLGNDT.....HTRRP
P_nodorum              K......................KRPAFTLNHHPSQTTLRTAKLPATTATTASVSHTRRP
B_bassiana             ...............................................TTRSVAIPCKTA.
G_zeae                 ...............................................SKRSVANPAVNA.
N_crassa_OR74A         ...............................................TKRSICQPISSSI
E_lata                 ...............................................AEPVFRRAIQPVS
C_cerevisiae           ...............................................VAKSYALETVSGE
S_pombe                ...............................................NIKSVVPTQQNSV
S_complicata           ...............................................HEEKREVLQNGTK
Y_lipolytica           ...............................................NEASREVCQPVDG
T_melanosporum         ...............................................EESGRVVIEGI..
A_oligospora           ...............................................YEASRTAIQPCAS
P_carinii              ...............................................IE..RTVIRPSES
T_deformans            ...............................................IKTSK.CIEPCKV
W_ichthyophaga         ...............................................NAIIPTTSTIDSH
S_punctatus            ...............................................QTTVINAIKDPPA
B_dendrobatidis        ...............................................RTVPISVNVNPPP
C_neoformans           ...............................................ISHDPIIPSDPLE
A_macrogynus           ...............................................IKSTAPITPSNAA
B_saltans              .................................................DRVFPIIPTAG
T_cruzi                ...............................................ACRPIQPCECVNR
L_major                ...............................................AYTA.SPIQPTSS
M_osmundae             ...............................................DAKVCRPVANPPA
M_lychnidis-dioicae    ...............................................TSKAIEESKEQPE
Perkinsela_sp.         ...............................................PCDDRIANERVAS
R_irregularis          ...............................................PIAPAQNPPEGLF
G_prolifera            ...............................................VVAPITPSTSPLA
B_floridae             ...............................................TRINVRCSQHALV
F_alba                 ...............................................PTSVRPISQFTGA
T_anomala              ................................................DVAFQAAGLAAL
U_maydis               ...............................................PELKSAESVNDFR
S_borealis             ...............................................PLKKDQSTGTESI
M_globosa              ...............................................SHVPKPESLLRAV
P_graminis             ...............................................GQIPHQNKIYSVS
B_mori                 ...............................................SKPILQ...SNNP
N_vitripennis          ...............................................SHPIEP...SVEP
P_humanus              ...............................................INPITPTNLSDNP
D_melanogaster         ...............................................IRPLEP.ELGAPP
A_gambiae              ...............................................SRPILE..NPINA
L_gigantea             ...............................................LAMLNP...VPKI
I_scapularis           ...............................................SEPVEK..DSVDP
X_tropicalis           ...............................................SVPLRASTSLTYP
E_histolytica          ...............................................LTSVPKNTTAIEP
C_viscosa              ...............................................FQPFRGRWEKWER
A_niger_YbgI           ............................................................
R_microsporus          ............................................................
Chrysochromulina_sp.   ............................................................
E_huxleyi              ............................................................
N_ceranae              ............................................................
E_cuniculi             ............................................................
V_culicis              ............................................................
C_cinerea              ............................................................
B_hominis              ............................................................
C_albicans             ............................................................
A_castellanii          ............................................................
consensus>50           ............................................................
```

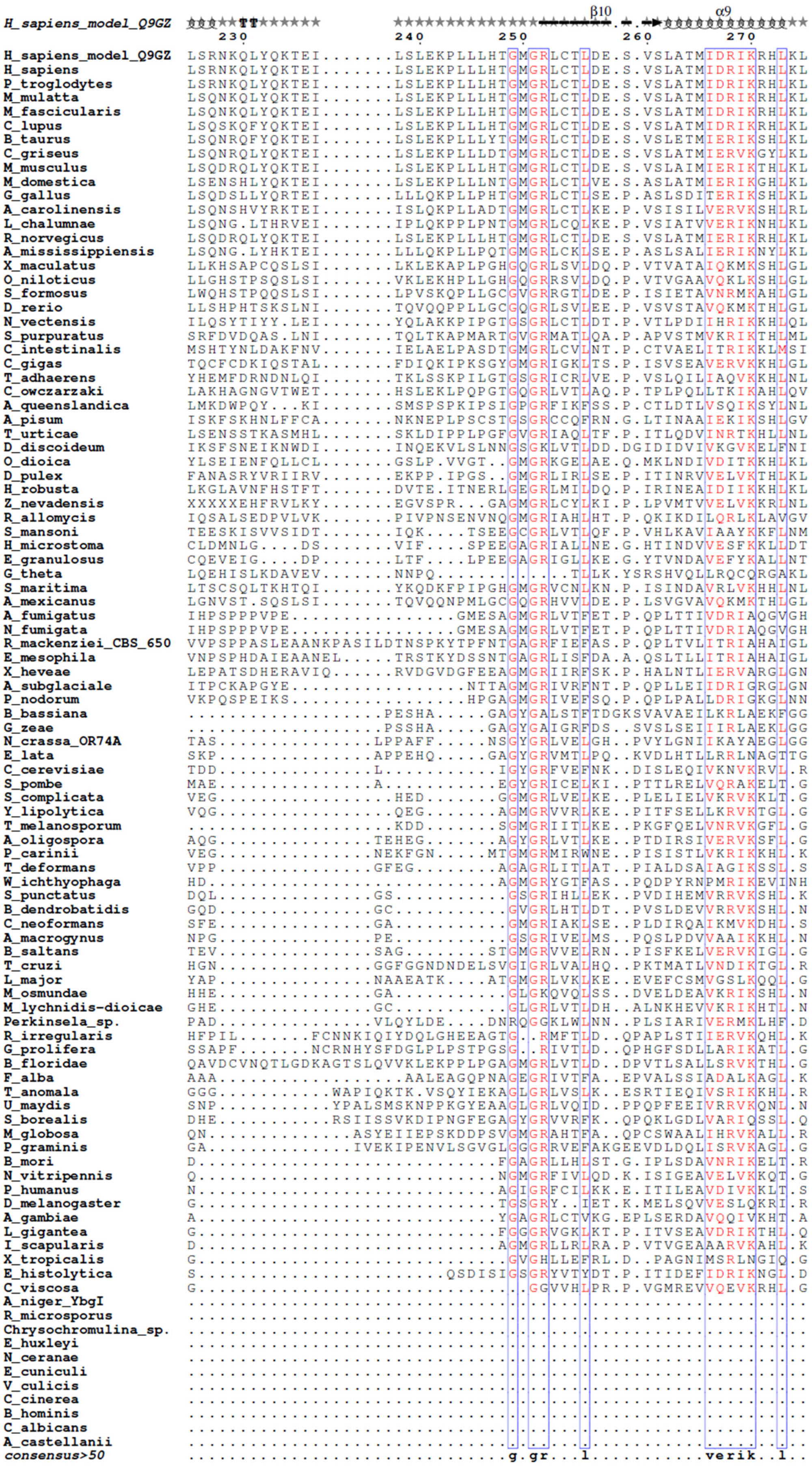
H_sapiens_model_Q9GZ
β10
α9
230
240
250
260
270
consensus>50
.g.gr..l..........verik..l..

```
                           β11                         β12             η2
H_sapiens_model_Q9GZ
                            280       290                     300                310

H_sapiens_model_Q9GZ     SH.IRLALGVGRTLESQV.................KVVALCAGSGS.SVLQGV......E
H_sapiens                SH.IRLALGVGRTLESQV.................KVVALCAGSGS.SVLQGV......E
P_troglodytes            SH.IRLALGVGRTLESQV.................KVVALCAGSGS.SVLQGV......E
M_mulatta                SH.IRLALGVGRTLESQV.................KVVALCAGSGS.GVLQGV......K
M_fascicularis           SH.IRLALGVGRTLESQV.................KVVALCAGSGS.SILQGV......K
C_lupus                  SH.VRLALGVGRTLESPV.................KVVALCAGSGS.TVLQGA......E
B_taurus                 SH.VRLALGIGKTLESPV.................KVVALCAGSGS.SVLQGT......D
C_griseus                SH.LRLALGVGKTLESPV.................KVVALCAGSGG.SVLQGV......E
M_musculus               SH.LRLALGVGRTLESQV.................KVVALCAGSGG.SVLQGV......E
M_domestica              AH.LRLALGVGRTLESPI.................KVVALCAGSGS.SVLQGV......E
G_gallus                 PH.VRLAVGTGKTLDSPV.................KKAALCAGSGS.SVLKGV......E
A_carolinensis           PH.VRLALGTGKSLESQV.................KVVALCAGSGA.SVLRGT......E
L_chalumnae              NH.VRLALGSGKTLDSVL.................KTAAVCAGSGG.SVLQGI......E
R_norvegicus             PH.LRLALGVGRTLGHRM....................ASNNCSFC.FYLRST......G
A_mississippiensis       SY.VRLALGVGKTLESQI.................RTAAVCAGSGR.SVLQGV......E
X_maculatus              AN.LRLALGAGRTLESSV.................CTAAVCAGSGA.SVLSGV......Q
O_niloticus              SH.LRLAVGWGQTLESSV.................CTVAVCAGSGA.SVLNGV......K
S_formosus               KH.LRLALGAQKTLESTV.................TTVAVCAGSGG.SVLQGV......T
D_rerio                  PH.LRLALGDQQTLDSMV.................KTVAVCAGSGA.SVIQGV......T
N_vectensis              PH.LRVAYAPSKAKDTLL.................STVALCAGSGA.SVLQGV......A
S_purpuratus             EH.VQIALGQGKTLDSEI.................STVGICAGSGS.SVLNGL......K
C_intestinalis           PY.VRLSLGSDKTVTSMV.................NTVALCAGSGS.SVLSGV......K
C_gigas                  AH.IRLAKGIGC...DSI.................SSVAVCAGSGG.GLLKNL......N
T_adhaerens              SS.LRLAADFYKPLSKLI.................RTVAVVAGAGG.SLLKGV......K
C_owczarzaki             EH.VRIALPSAAVPAAQVPPKTQAAALEHAKNVLVQKIALCAGSGS.SVMRGV......R
A_queenslandica          TN.IRLARPSSWKEGQLA.................SSLAVCVGSGA.SVLRCT......R
A_pisum                  DN.LMIALARGSTFESTI.................DSAAAVAGSGA.SVLRGV......H
T_urticae                NH.VRLALAPKHSLDSNI.................NSIAVCAGSGG.SVLRGL......S
D_discoideum             EY.VRIGRPLSGESKKI..................KTISLCAGSGG.SVVFNA......K
O_dioica                 AN.LRLVTANDKTSE.................DMVSTVAVCAGSGA.SVLSHAG.....K
D_pulex                  QH.VRLATAGNAENN.................MNVETIALCAGSGA.SLLKNV......K
H_robusta                DH.VLLASSHHLQKKN...........FNDGYQLISSIAVCAGSGS.SVLKGA......K
Z_nevadensis             LK.IRLALARHKHMESPV.................STVALCAGSGS.SVLRGA......K
R_allomycis              KH.LRVSVSKNHQIEDEI................INGIAVCAGSGG.SLFKKIEDGDLEK
S_mansoni                GH.LTVAPGLGKTLDCPI.................KTVAVCAGSGG.SLLCQTPA..SFA
H_microstoma             SL.LKVALGYGKTFDSPV.................SALAVCAGSGG.SMFTSEPM..AQV
E_granulosus             SI.LKVALGYGKTFDSPV.................TAVAVCAGSGG.SLFSQEAI..VQV
G_theta                  EN.IKI.............................KSVAVQAGSGG.SVLLGC......P
S_maritima               NF.LRVASGVNKI...............................................
A_mexicanus              PH.LRLALGEQKTLGKKIIYFIIYRTL.................................
A_fumigatus              PGGIPIAIPQTVPVDLIK................IRTIGICPGSGS.SIL....MSSGSL
N_fumigata               PGGIPIAIPQTVPVDLIK................IRTIGICPGSGS.SIL....MSSGSL
R_mackenziei_CBS_650     PKGFAVAIPQGRGIENIQ................IATVATCPGSGA.SVL....RSCHA.
E_mesophila              PKGFSMAIPQGVRIEDIQ................IKTVATCPGSGG.SVL....SKCRA.
X_heveae                 PKAFPIAIPQGKTIDEIE................ISSVGICAGSGG.SLF....RNLDV.
A_subglaciale            PKGFPIAIPQGKTMEEID................ISSVAICAGSGG.SLFSGLGKNGEED
P_nodorum                PKGFPIAIPQNATISDIK................IRSVGICAGSGG.SLL......GDLE
B_bassiana               LRHVMVASPV................GADVRTTRVRSYGVCAGSGY.DVLKSA......D
G_zeae                   LKHVMVASPV................GADIKTTKVSSFGVCAGSGY.DVLKKA......D
N_crassa_OR74A           LNHIMIAAP..................KDKKVTTIRSVGICAGSGA.DVLKNC......D
E_lata                   SPYAMVATPS............HIRNSDASNPPMIGRIAVCAGSGA.GVFKDLGDDN..D
C_cerevisiae             VPYVQVASLA..............APSAWNQLK.IKKVAVCAGSGS.GVFKQLKE....D
S_pombe                  LQYVQVCAPN..............GLDSH.....ISKVSLCAGSGG.SVVMNT......D
S_complicata             LKYVQVARPQ..............LLE....K..VKTIAICAGSGG.SLFRGV......Q
Y_lipolytica             LKHVQVALTE..............KHENPEAK..ISSVAVCAGSGA.SVLRGV......D
T_melanosporum           LKNVMVGKTP..............GDK....R..VRTIAICAGSGA.SMFKNV......D
A_oligospora             MSYLQVALAK..............RHTATPTERDIRTIGICAGSGG.SVFRDV......K
P_carinii                LAQIRLALSE..............KHRREGGNC.IYKIGICAGSGG.SILRNV......H
T_deformans              ISHVQVARAT..............NKKDDEM...IHTIALCAGSGG.SVLKGL......A
W_ichthyophaga           IKSVFNLDTL...............QFADGGKKHINSVAVCAGSGS.SILSKAP.....S
S_punctatus              LAHVRLATP....................TQNKPITSIAICAGSGA.SVLMNTKA.....
B_dendrobatidis          LNHLRLARAA................DYRTDTKLVSTIAICAGSGF.SVLDGVTA.....
C_neoformans             LDFVQLAEPQ................P..DVRKPIKSVAVCAGSGG.SVFKGVEA.....
A_macrogynus             LPFVRVATA....................PRNHKISSIAMCAGSGA.SVFSRATT.R..P
B_saltans                IPTVRVSAPG................GQ.E..RLVRRVAVCVGSGT.SVFKSLKS.G..A
T_cruzi                  IPTVRVSLPH................GW.SGNTSVHSVAICAGSGS.SVFRMIRE....H
L_major                  LATVRVALPD................SW.EPSHKVSSVALCAGSGT.SVFRLLRL....P
M_osmundae               LKTVMLARSD................Q..PRSQRVRSIAICAGSGG.SVIGNADA.....
M_lychnidis-dioicae      LDHAQAAWAP................N..G.AEKIKTIAICAGSGS.SVLSGVKA.....
Perkinsela_sp.           VQFVRCALGV................EH.TMDSPVRTIGICAGSGS.SVL.GPYA.G..Q
R_irregularis            LRYVRVATTN.........KHS....SG....ELISTIGICAGSGS.SVLLPAT......
G_prolifera              LPRIRIS..R.........PFN....PV....EEIRTIAICAGSGS.SVLVPLAG....K
B_floridae               LNHVRLAMGE.........GRTM...EG....STVRSVALCAGSGG.SVLKGV......K
F_alba                   LEYLRCVLPD.........QATP....VAAGQQTVSQLAICAGSGA.SVLAAAGP....G
T_anomala                LKYVLVAQA...................QPPDHQIETIAVQVGSGG.SVLQGV......Y
U_maydis                 LD.HVQGCKA....................SDKPISTVAVCAGSGS.SVFSGV......K
S_borealis               IEGRISGLSV.........AVPQSIPRGQKSSIEISSIGICAGSGG.SMLNGL......D
M_globosa                VSHVQIAAAS.................GVNETTQIRSIAVCAGSGG.SVLRGW.T....D
P_graminis               IPHVQLAKSS.................FAPPT..IRSVGVCAGSGG.SICSALGK....S
B_mori                   LSHVRIAAAK...............GKSMTH..TVQRVALCAGSGS.SVLKGVD......
N_vitripennis            LPHVRLARAR...............GSSMHFYGSISKIGLCAGSGA.SVLKGLS......
P_humanus                LQFINLALAR...............SASLET..KIKKIGICAGSGS.SVLQNIQ......
D_melanogaster           .NSVHVALAV...............GHTPKT..LIQSVGICAGSGA.SLLKGIQ......
A_gambiae                MDCAMVSFAS...............SGDKNR..KVQTYAVCAGSGA.SVLKGVK......
L_gigantea               LNHIRLAAKP...............SNKP.....IQSVAVCAGSGS.SVLRGVR......
I_scapularis             LPSVRLALGS...............GQTAES..TVQAVAVCAGSGG.SVLKGVAG.....
X_tropicalis             VSVCTSTARH...............DGDNGTRVSLSCSQNALVEVL.SILSGVP......
E_histolytica            VP.LRYAIPS...............TKKE.....IKTVAICCGSGS.MMFANKC......
C_viscosa                VQTVNVGTSN...............PEGTV...QVQRIALCAGSGGTSLAEQGGE.....
A_niger_YbgI             ............................................................
R_microsporus            ............................................................
Chrysochromulina_sp.     ............................................................
E_huxleyi                ............................................................
N_ceranae                ............................................................
E_cuniculi               ............................................................
V_culicis                ............................................................
C_cinerea                ............................................................
B_hominis                ............................................................
C_albicans               ............................................................
A_castellanii            ............................................................
consensus>50             ...vr.a...........................i..vavcagsg..svl..........
```

```
                      β13          α10            β14   η3      α11
H_sapiens_model_Q9GZ
                             320       330       340       350
H_sapiens_model_Q9GZ  ADLYLTGEMSHHDTLDAA.SQ..GINVILCEHSNTERGFLSDLRDMLD.SHL........
H_sapiens             ADLYLTGEMSHHDTLDAA.SQ..GINVILCEHSNTERGFLSDLRDMLD.SHL........
P_troglodytes         ADLYLTGEMSHHDILDAA.SQ..GINVILCEHSNTERGFLSDLRDMLD.SHL........
M_mulatta             ADLYLTGEMSHHDILDAA.SQ..GINVILCEHSNTERGFLSDLRDMLG.SHL........
M_fascicularis        ADLYLTGEMSHHDILDAA.SQ..GINVILCEHSNTERGFLSDLRDMLG.SHL........
C_lupus               ADLYLTGEMSHHDVLDAA.SQ..GINVILCEHSNTERGFLSDLRDMLG.AHL........
B_taurus              ADLYLTGEMSHHDVLDAA.SQ..GISVILCEHSNTERGFLSDLRDMLD.AHL........
C_griseus             ADLYLTGEMSHHDVLDAA.SK..GINVILCEHSNTERGFLSDLQQMLG.VHL........
M_musculus            ADLYLTGEMSHHDVLDAA.SK..GINVILCEHSNTERGFLSELQEMLG.VHF........
M_domestica           ADLYLTGEMSHHDVLDAA.AQ..GINVILCEHSNTERGFLSELQDTLT.AHL........
G_gallus              ADLYLTGEMSHHDVLDAV.AN..GISVILCEHSNTERGFLSELRDTLT.IHL........
A_carolinensis        SDLYLTGEMSHHDVLDAV.SK..GITVILCEHSNTERGFLLELQQALA.LHL........
L_chalumnae           ADLYLTGEMSHHDVLDAV.AK..GTSVVLCEHSNTERGFLSELKEMLT.ARF........
R_norvegicus          IQVYIAIIHSLQSKLWPC.VL..VLGAAFYKE............................
A_mississippiensis    ADLYLTGEMSHHEVLDVV.GN..GISKSIETLFNWHRRKQRLTN................
X_maculatus           ADLFITGEMSHHEVLDAV.AK..GTSVILSDHSNSERGFLAVFRERLA.VRL........
O_niloticus           ADLYVTGEMSHHEVLDAV.AK..GTSVILSDHSNSERGYLAVFRERLA.VRL........
S_formosus            ADLYITGEMSHHEVLDAV.AA..GTSVILTDHSNSERGFLSVLRERLS.VHL........
D_rerio               ADLYITGEMSHHEVLDAV.SK..GTSVILSEHSNSERGFLGVFRERLS.ARL........
N_vectensis           ADVYLTGEMSHHEVLEAV.SR..GVHVILCEHSNTERGFLVPFRQQLY.NML........
S_purpuratus          ADMFLTGEMSHHDLLAAV.AE..GTTVVVCNHSNTERGYLHVLQGKLT.SMC........
C_intestinalis        ADVYLSGEMSHHEVLDAA.SN..GVTTVLCEHSNTERGHLKSMKKSLE.DML........
C_gigas               VSLYVTGEMSHHDVLHAA.QS..GVSVILCDHSNTERGFLKVLQESLT.KVF........
T_adhaerens           ADLYLTGEMSHHDVLAAV.AS..GTCVLLCDHTNTERGYLFNKLKLDL.GSL........
C_owczarzaki          ADVYLTGEMGHHDVLDAV.SS..GAAVVLCEHTNTERGYLAKLRESLA.ELF........
A_queenslandica       PDVYLTGEMSHHEILEAT.AK..GIVVILCEHSNTERGFLHILKSLIE.EKL........
A_pisum               AGLYLTGEMSHHDVLDAVHNN...VTVVLANHSNTERSYLNVFAGRLL.AEM........
T_urticae             VDLLITGEMSHHEVLDALYSN..GSSVILCEHTNTERGYLHVLREKLI.TKL........
D_discoideum          ADLYLTGELTHHAILDAC.AK..GSYVIVCDHSNSERDYLPQILKPNL.EKL........
O_dioica              VDLWITGEMGHHEALDAHENN...TSVILAEHSNTERGFLESFKAKLMDNEQ........
D_pulex               ADVFLTGEMSHHEVLDAVHRG...TSVILCEHSNTERGFLSKWKEVL...HS........
H_robusta             ANLYITGEMGHHELLDVTNNTCAPSSVILCNHSNTERGFLSDFMLGYL..KG........
Z_nevadensis          ADLYLTGEMQHHDILDAVHR...GTHVILCDHSNTERGFLK.VFAEKLSMDL........
R_allomycis           VDLLITGEMSHHDILSWRFK...GKSVILCEHSNSERGFLKNVYAKRIQELL........
S_mansoni             ADLFITGELSHHDRLEAVSR...GISIILAGHSVTERGFFKERFIPDFRHLL........
H_microstoma          ADLLFTGEASYHDQLDTISR...GNTIITAGHSVSERGYLTQRLKPWLEKEI........
E_granulosus          ADLLVTGEASHHEQLEAVAR...GATIITAGHSVSERGYLSQRLRPWLQHEL........
G_theta               ADLLVMGEMSHHDALAAIAA...GSSVILTEHSNSERGFLPQMVDLLQTRMQ........
S_maritima            ............................................................
A_mexicanus           ............................................................
A_fumigatus           PDLLFTGELSHHEALSAVER...GSVVIALAHSNTERGYLHAVMRQKLAATL........
N_fumigata            PDLLFTGELSHHEALSAVER...GSVVIALAHSNTERGYLHAVMRQKLAATL........
R_mackenziei_CBS_650  .DLIFTGELSHHDALAVTER...GGCVIALFHSNSERGYLWSVMRERLEKEV........
E_mesophila           .DLILTGELSHHETLAVTER...GGCVISLFHSNSERGYLWSVMRDQLERRA........
X_heveae              .DLFFTGELSHHEALAAIER...GQCIITLFHSNTERGFLHSVLRDQLSTTL........
A_subglaciale         VDLLFTGELSHHEALAAIEQ...GKCVIALFHSNTERGFLHGVLKPQLENAV........
P_nodorum             VDLLFTGEMSHHEALAAIEK...GQVVISLFHSNSERGFLAEVLLAQLEEAV........
B_bassiana            VDLLVMGETSHHSALRAIQQ...GRTLVQVFHSNSERSYLQQVLRPALQEK...L.RAAV
G_zeae                VDLIVTGETSHHSALRAIQQ...GRTLVQVFHSNSERGYLQEVLRPRLEAA...I.RETV
N_crassa_OR74A        ADMIVTGEMTHHYALALTMK...GKVVMTVFHSNSERKFLKKRLQPQLQAQ...LEKEGV
E_lata                VELLVTGEMSHHDALRHTQL...GRVVATVFHSNSERRYLRDVLKPVLEGK...LRGTPF
C_cerevisiae          VDLYYTGEMSHHEVLKWKEM...GKTVIVCNHSNTERGFLQDVMKGLLQD..........
S_pombe               ADLYFTGELSHHQVLAAMAK...GISVILCGHSNTERGYLKDVMCQKLAS.......SFH
S_complicata          ADCFFTGELGHHETLATVEN...GMSAIICGHTNTERGFLAAVMKPRLEEL...LK...E
Y_lipolytica          ADVHFTGELGHHEALGLIEN...GTSAIVCGHSNTERGYLKTFQTLLTKEL...KN...Q
T_melanosporum        VDLLFTGELSHHDALAAKER...GVGFITCFHTNTERGFLRDVMKRKLAEV...LESEVE
A_oligospora          ADLLFTGEMSHHEALAAKES...GTSIVACFHSNTERGYLPFMRQKLLETLPRAWEETVE
P_carinii             ADVLFTGELSHHDILHATEK...GSSVILCGHSNTERGFLSCVMKKKLQDVLYDEFSAMQ
T_deformans           ADLIFTGELSHHEQLAFVEA...GSHVVLCGHSNTERPFLPS.MKRQIEDL.......CK
W_ichthyophaga        VDLYVTGEMGHHDVLAAVAN...GTSVILLNHSNSERVYLQTSLRDALTEAIQIEREEAK
S_punctatus           .DLYLTGEMSHHEVLAALAD...DISVILCEHTNTERGYLAEVLQPRLQTL...LR....
B_dendrobatidis       .DVYFTGEMSHHEVLGGVSK...GINVILCEHTNTERGYLSQVLQARLLSL...LKVDLG
C_neoformans          .DLLITGEMSHHEILAYVAS...GTTVILTNHTNTERPYLSHVLQPWLQEE...LNKETK
A_macrogynus          .DLLWTGEMGHHEVLAAVQT...GSHVVLCEHTNTERGYLSAVLKPRLERD...AGL...
B_saltans             VDVLLSGEMGHHDVLAAADN...NQSVILTEHTNTERGFLRAVLKGALQEK...LGEDV.
T_cruzi               VDVLLSGEMGHHEVLAANAK...GQAVILCEHTNTERGYLEKELLPKLQQK...LEKGV.
L_major               VHVLLSGEMSHHDVLAATAA...GQAVILCEHTNTERGFLKAVLQRTLQAK...LE.GT.
M_osmundae            .DLLWTGEMSHHEILAATAR...GTHVVLCNHSNTERGYL.HT....LKRK...LEEELS
M_lychnidis-dioicae   .DLYLSGELDHHATLLCNAK...GIHVILTNHSASERPWL.KPFAPRLAKA...MNEEAG
Perkinsela_sp.        LDCVISGEISHHEILRLQQS...GTTVLLVEHGTSERGYLENKLLPRLEIV...MR....
R_irregularis         ADLYFTGEMSHHDILAALDK...NTSVILCEHSNTERGYLSEVLKPKLEQLL.......K
G_prolifera           ADLLFTGEMSHHDLLACQEK...GSIAVMCEHSGSERRYLM.CLKDVLGEVL.......K
B_floridae            ADVYLTGEMSHHNVLDAVAM...GTSVILCEHSNTERGFLR.VLQGQLTAML........
F_alba                VDGWLTGEMGHHEALSAAAR...GVSVLLCEHSNSERGYLTDVMQPLLKEKL.......Q
T_anomala             ADLYVTGEMSHHELLAANSR...GISVILLHHSNSERGYLQDVLVHLLHIFL........
U_maydis              ADLYLTGELSHHEILAYQAA...GASVIVTNHTNTERKYLRDVLQHSLQEEL........
S_borealis            VDLLFTGELSHHEALAAVEQ...GKCAVTAFHSNTERAFLKDRMQAALAEAM........
M_globosa             ADVYVTGEMGHHDILAANAR...GISVILTNHTNTERRYLVDVLQPALSSSL........
P_graminis            CDAFVTGEMSHHELLSANAR...GIHIILTAHTNTERFFLAKVLQPRLHELLLDEAAQMS
B_mori                ADLYLTGEMLHHDVLDAAQK...GITVILTNHSDSERGFLRG.FANYLHKNLNE.E....
N_vitripennis         ADLYVTGEMLHHDILDAVHG...GASVILTNHSDSERGFLKAVFAQKLTSLLQD.N....
P_humanus             ADLFLTGEMSHHSLLEAIHN...NTSVILCNHSNSERGFLKE.FSKILEGNFNKYD....
D_melanogaster        ADLIITGEMSHHEVLEFTHN...NTTVLLCNHSNSERGFLHE.FCPILAKSLNE.E....
A_gambiae             ADMYITGEMSHHEVLEATSN...GTCVVLLGHSNSERGYLSV.FKDILSKRLNG.G....
L_gigantea            ADLYVTGEMSHHDVLDAVYN...GANVILCEHSNTERRYLKQ.VKQELYKTLT..D....
I_scapularis          AELYLTGEMSHHEVLDAQQR...GVSVLLTEHSYSERGLPRAVRREAPPRAGCGRR....
X_tropicalis          AELYLTGEMSHHDVLDAVAE...GRSVVLCEHSNSERGYLQELGGQI..RQALEGQ....
E_histolytica         ADAVITGEMGHHEVLEHLEW...NTAVVLSEHTNTERGYLKHWIPRLSELFPS.......
C_viscosa             ADCWLTGEMGHHELLAALAS...GKTVILCGHTNTERGYLPLLREKLLGELGQGWE....
A_niger_YbgI          ............................................................
R_microsporus         ............................................................
Chrysochromulina_sp.  ............................................................
E_huxleyi             ............................................................
N_ceranae             ............................................................
E_cuniculi            ............................................................
V_culicis             ............................................................
C_cinerea             ............................................................
B_hominis             ............................................................
C_albicans            ............................................................
A_castellanii         ............................................................
consensus>50          adly.tgemshhevl.a......g..vil.ehsntergfl.......l............
```

```
                          β15
H_sapiens_model_Q9GZ  ***        ***************
                      360             370
H_sapiens_model_Q9GZ  .ENK........INIILSETDRDPLQVV................................
H_sapiens             .ENK........INIILSETDRDPLQVV................................
P_troglodytes         .ENK........INIILSETDRDPLQVV................................
M_mulatta             .ENK........INIILSETDRDPLQVV................................
M_fascicularis        .ENK........INIILSETDRDPLQVV................................
C_lupus               .ENK........INIILSETDRDPLHVV................................
B_taurus              .ENK........INIILSETDRDPLHVI................................
C_griseus             .ENK........INIILSETDRDPLHVV................................
M_musculus            .ENK........INIILSETDRDPLRVV................................
M_domestica           .EYK........VNIILSETDRDPLQVV................................
G_gallus              .QNK........INIILSEKDRDPLQVV................................
A_carolinensis        .DGK........IHIIVSERDRDPLKVA................................
L_chalumnae           .HKQ........LKVIVSSVDKDPLQVL................................
R_norvegicus          ............................................................
A_mississippiensis    ............................................................
X_maculatus           .PDA........VTVVLSKADRDPLEVV................................
O_niloticus           .PDS........VTVVLSKADRDPLEVV................................
S_formosus            .DDS........VSVQLSQKDRDPLEVM................................
D_rerio               .DHT........VSIVISQTDRDPLQVV................................
N_vectensis           .SGQ........VQVMVSETDADPLLVV................................
S_purpuratus          .ENK........IKVFVSEKDRDPLEVV................................
C_intestinalis        .GSSL.......VKIIVSLVDEDPLHIC................................
C_gigas               .QNQ........VDIQVSKSDKDPLDVV................................
T_adhaerens           .LDNR.......IEIITSKVDGDPLAVI................................
C_owczarzaki          .GTAG.......VQVTVSQQDADPLVIV................................
A_queenslandica       .QQSTP......LKITISGVDRDPVTIV................................
A_pisum               ...PQ.......LNVHVSKVDRDPLVFV................................
T_urticae             .QNPN.......VEVIVAKSDADPLNIV................................
D_discoideum          .FNGR.......IKVIVSKLDTDPLKVI................................
O_dioica              .IKKRD......IQFVVSDVDSDPIKVV................................
D_pulex               .ALGED......VQIDVSLNDHDPLQVV................................
H_robusta             .ELKDK......VEILVSECDVDPLVVV................................
Z_nevadensis          .SGGN.......VEVVVSQIDRDPLQVI................................
R_allomycis           .PSDE.......FTVEYSEDDHEPIQII................................
S_mansoni             .STVHNAGEIPHVELLLSEADHEPGFVV................................
H_microstoma          .AVLTDA....QILIDISSKDSEPGIYVC...............................
E_granulosus          .ATTS.......VPIDIASTDAEPGIYVLPPGIQ..........................
G_theta               .AAGYD.....KLQYIISTTDSDPLSVV................................
S_maritima            ............................................................
A_mexicanus           ............................................................
A_fumigatus           .KEEWEAQREEGLKALEETFKQGGASVIGSYEEVYKDPSCAVDVSERDRDPYGIMIRRA.
N_fumigata            .KEEWEAQREEGLKALEETFKQGGASVIGSYEEVYKDPSCAVDVSERDRDPYGIMIRRA.
R_mackenziei_CBS_650  .KSEWERVRSQNRGESGKNGVPEQITEMLDDDSV....GVVVSQRDRDP..YGIVVLDET
E_mesophila           .RIEWTKLQGENL..SNQNLSEPERAILCNHQLV....EVLVSEWDRDP..YGIVVLDDT
X_heveae              .KDEWQRIRSGELEKLRIDFPPPSSARPQSSSTR....PQISRKATSSSNFHDPFVTSSA
A_subglaciale         .KEEWERVRQEEKNKAGLSEDYLEALEDDHVE.........IHVSEVDRDPYGIMISKDE
P_nodorum             .ETEWSQVREENIGK....EELKDALADEDFE.........VIVSEVDRDPYGIVVLKPE
B_bassiana            PEA..........AVVLSEYDKDPFTIVDVAEL...........................
G_zeae                PEV..........EVVTSKVDKDPFTILDVSDLK..........................
N_crassa_OR74A        KHP..........EVLVSEEDADPFEIWDVRKMPA...WAFGKDSE..............
E_lata                GDTT.........RVIVSQADRDPFDIINIGPVA..........................
C_cerevisiae          .EGH.........EVVVSKMDCDPLTVA................................
S_pombe               KEGVD.......ANVIVSSMDADPLTTM................................
S_complicata          EEEK........IEVVVSEGDRDPVEIM................................
Y_lipolytica          HDGE........VEVIISQTDKDPLEIV................................
T_melanosporum        REGE........FEVVVSERDMDPFEIV................................
A_oligospora          EEGSDEMLDVEGWEVHISGVDRDPYDIV................................
P_carinii             DDQLLL......PEVVVSEADKDPLEVV................................
T_deformans           EEH.........VQVVVSAADASPFETV................................
W_ichthyophaga        LEPVK.......WKVTTCVEDKDPLVSV................................
S_punctatus           ...ETEPAT....EVVVSSIDRDPLVVV................................
B_dendrobatidis       LDVEADVHI....DVTVSKCDQDPFVIV................................
C_neoformans          DHSNEYGANGGKWEVLVSKADADPLKVV................................
A_macrogynus          .............KVVVSAVDRDPLVVQ................................
B_saltans             .............AIEVSTRDADPLVVW................................
T_cruzi               .............RIIVSAEDKDPLVVW................................
L_major               .............TVLVSDKDSDPLTAW................................
M_osmundae            TDPQAESEA...WRVIIAQEDRDPLDDEAASQNTF...SVVSLRCCSGAQSSFVDEPRHK
M_lychnidis-dioicae   GDAYETKGD...GSYVVKDYNNVQQGYNGLYNNPQ...HGSEGLLFKDRIYQTLDPLDHH
Perkinsela_sp.        .............DIPLENGRCDETFIQTMKSDAS...LLSYC.................
R_irregularis         EGKEEK......IDVVVSKVDKDPLETV................................
G_prolifera           SDGGDV......ANVVVSEKDKDPVEIV................................
B_floridae            ...DGK......VEVVVSDVDKDPLEVV................................
F_alba                ...SDQ......VSVVVSQVDRDPIQLL................................
T_anomala             ...GEE......YGIRVSARDHEPLSVM................................
U_maydis              ...SGK......YTVLVSQNDRDPLRVL................................
S_borealis            ...EGK......ADIAVSEVDRDPFDIVHKDEVNW.........................
M_globosa             ....PG......SHVYVSERDADPLCVA................................
P_graminis            EHQTPQ......WEVEVSVADRDPLVVA................................
B_mori                ............VEVFVSKVDKDPLITV................................
N_vitripennis         ............VVVSVSEQDEDPLKTV................................
P_humanus             ............LKVLVSKTDRDPIQTI................................
D_melanogaster        ............CLVFVSEVDKDPLVTVASDINKE...LSAFVDVYKSTSK.........
A_gambiae             ............VTVHVSSSDRDPMKLALRENAN..........................
L_gigantea            ............VSIVISDIDTDPLHIV................................
I_scapularis          ............AGVDCGHRTAGRALGGRVS.............................
X_tropicalis          ............VQVVVSQRDRDPLQVV................................
E_histolytica         ............VTFYQSECDVSPLVYMV...............................
C_viscosa             ............VDV..SEVDGHPLTVQ................................
A_niger_YbgI          ............................................................
R_microsporus         ............................................................
Chrysochromulina_sp.  ............................................................
E_huxleyi             ............................................................
N_ceranae             ............................................................
E_cuniculi            ............................................................
V_culicis             ............................................................
C_cinerea             ............................................................
B_hominis             ............................................................
C_albicans            ............................................................
A_castellanii         ............................................................
consensus>50          ..............v.vs..d.dpl.vv................................
```

H_sapiens_model_Q9GZ

```
H_sapiens_model_Q9GZ  ............................................................
H_sapiens             ............................................................
P_troglodytes         ............................................................
M_mulatta             ............................................................
M_fascicularis        ............................................................
C_lupus               ............................................................
B_taurus              ............................................................
C_griseus             ............................................................
M_musculus            ............................................................
M_domestica           ............................................................
G_gallus              ............................................................
A_carolinensis        ............................................................
L_chalumnae           ............................................................
R_norvegicus          ............................................................
A_mississippiensis    ............................................................
X_maculatus           ............................................................
O_niloticus           ............................................................
S_formosus            ............................................................
D_rerio               ............................................................
N_vectensis           ............................................................
S_purpuratus          ............................................................
C_intestinalis        ............................................................
C_gigas               ............................................................
T_adhaerens           ............................................................
C_owczarzaki          ............................................................
A_queenslandica       ............................................................
A_pisum               ............................................................
T_urticae             ............................................................
D_discoideum          ............................................................
O_dioica              ............................................................
D_pulex               ............................................................
H_robusta             ............................................................
Z_nevadensis          ............................................................
R_allomycis           ............................................................
S_mansoni             ............................................................
H_microstoma          ............................................................
E_granulosus          ............................................................
G_theta               ............................................................
S_maritima            ............................................................
A_mexicanus           ............................................................
A_fumigatus           ............................................................
N_fumigata            ............................................................
R_mackenziei_CBS_650  AVESHGMVCIATGNGDEADTAMNADSSSEMEHALNLCRRGAPPRWDRYKMTPYTDEEALA
E_mesophila           PVPGVKLESA..................................................
X_heveae              SGPPASGKSQGATDGSAVSAAALRSILEDESVEVHVSERDRDP.YGIVILKSEVDVDVGV
A_subglaciale         I...........................................................
P_nodorum             AE..........................................................
B_bassiana            ............................................................
G_zeae                ............................................................
N_crassa_OR74A        ............................................................
E_lata                ............................................................
C_cerevisiae          ............................................................
S_pombe               ............................................................
S_complicata          ............................................................
Y_lipolytica          ............................................................
T_melanosporum        ............................................................
A_oligospora          ............................................................
P_carinii             ............................................................
T_deformans           ............................................................
W_ichthyophaga        ............................................................
S_punctatus           ............................................................
B_dendrobatidis       ............................................................
C_neoformans          ............................................................
A_macrogynus          ............................................................
B_saltans             ............................................................
T_cruzi               ............................................................
L_major               ............................................................
M_osmundae            ..DKSAWGARVSLEYFEATSVIGKTAARSRT.............................
M_lychnidis-dioicae   IFPPPPWLPAPEGRWGKLATLASIIPLISS..............................
Perkinsela_sp.        ............................................................
R_irregularis         ............................................................
G_prolifera           ............................................................
B_floridae            ............................................................
F_alba                ............................................................
T_anomala             ............................................................
U_maydis              ............................................................
S_borealis            ............................................................
M_globosa             ............................................................
P_graminis            ............................................................
B_mori                ............................................................
N_vitripennis         ............................................................
P_humanus             ............................................................
D_melanogaster        ............................................................
A_gambiae             ............................................................
L_gigantea            ............................................................
I_scapularis          ............................................................
X_tropicalis          ............................................................
E_histolytica         ............................................................
C_viscosa             ............................................................
A_niger_YbgI          ............................................................
R_microsporus         ............................................................
Chrysochromulina_sp.  ............................................................
E_huxleyi             ............................................................
N_ceranae             ............................................................
E_cuniculi            ............................................................
V_culicis             ............................................................
C_cinerea             ............................................................
B_hominis             ............................................................
C_albicans            ............................................................
A_castellanii         ............................................................
consensus>50          ............................................................
```

H_sapiens_model_Q9GZ

**H_sapiens_model_Q9GZ**	....
**H_sapiens**	....
**P_troglodytes**	....
**M_mulatta**	....
**M_fascicularis**	....
**C_lupus**	....
**B_taurus**	....
**C_griseus**	....
**M_musculus**	....
**M_domestica**	....
**G_gallus**	....
**A_carolinensis**	....
**L_chalumnae**	....
**R_norvegicus**	....
**A_mississippiensis**	....
**X_maculatus**	....
**O_niloticus**	....
**S_formosus**	....
**D_rerio**	....
**N_vectensis**	....
**S_purpuratus**	....
**C_intestinalis**	....
**C_gigas**	....
**T_adhaerens**	....
**C_owczarzaki**	....
**A_queenslandica**	....
**A_pisum**	....
**T_urticae**	....
**D_discoideum**	....
**O_dioica**	....
**D_pulex**	....
**H_robusta**	....
**Z_nevadensis**	....
**R_allomycis**	....
**S_mansoni**	....
**H_microstoma**	....
**E_granulosus**	....
**G_theta**	....
**S_maritima**	....
**A_mexicanus**	....
**A_fumigatus**	....
**N_fumigata**	....
**R_mackenziei_CBS_650**	QKVSSSPWLSDTGI
**E_mesophila**	....
**X_heveae**	DAGSDTELEGDI..
**A_subglaciale**	....
**P_nodorum**	....
**B_bassiana**	....
**G_zeae**	....
**N_crassa_OR74A**	....
**E_lata**	....
**C_cerevisiae**	....
**S_pombe**	....
**S_complicata**	....
**Y_lipolytica**	....
**T_melanosporum**	....
**A_oligospora**	....
**P_carinii**	....
**T_deformans**	....
**W_ichthyophaga**	....
**S_punctatus**	....
**B_dendrobatidis**	....
**C_neoformans**	....
**A_macrogynus**	....
**B_saltans**	....
**T_cruzi**	....
**L_major**	....
**M_osmundae**	....
**M_lychnidis-dioicae**	....
**Perkinsela_sp.**	....
**R_irregularis**	....
**G_prolifera**	....
**B_floridae**	....
**F_alba**	....
**T_anomala**	....
**U_maydis**	....
**S_borealis**	....
**M_globosa**	....
**P_graminis**	....
**B_mori**	....
**N_vitripennis**	....
**P_humanus**	....
**D_melanogaster**	....
**A_gambiae**	....
**L_gigantea**	....
**I_scapularis**	....
**X_tropicalis**	....
**E_histolytica**	....
**C_viscosa**	....
**A_niger_YbgI**	....
**R_microsporus**	....
**Chrysochromulina_sp.**	....
**E_huxleyi**	....
**N_ceranae**	....
**E_cuniculi**	....
**V_culicis**	....
**C_cinerea**	....
**B_hominis**	....
**C_albicans**	....
**A_castellanii**	....
consensus>50	....

Figure S3. Motifs per superkingdom arranged into tiers representing levels of conservation.

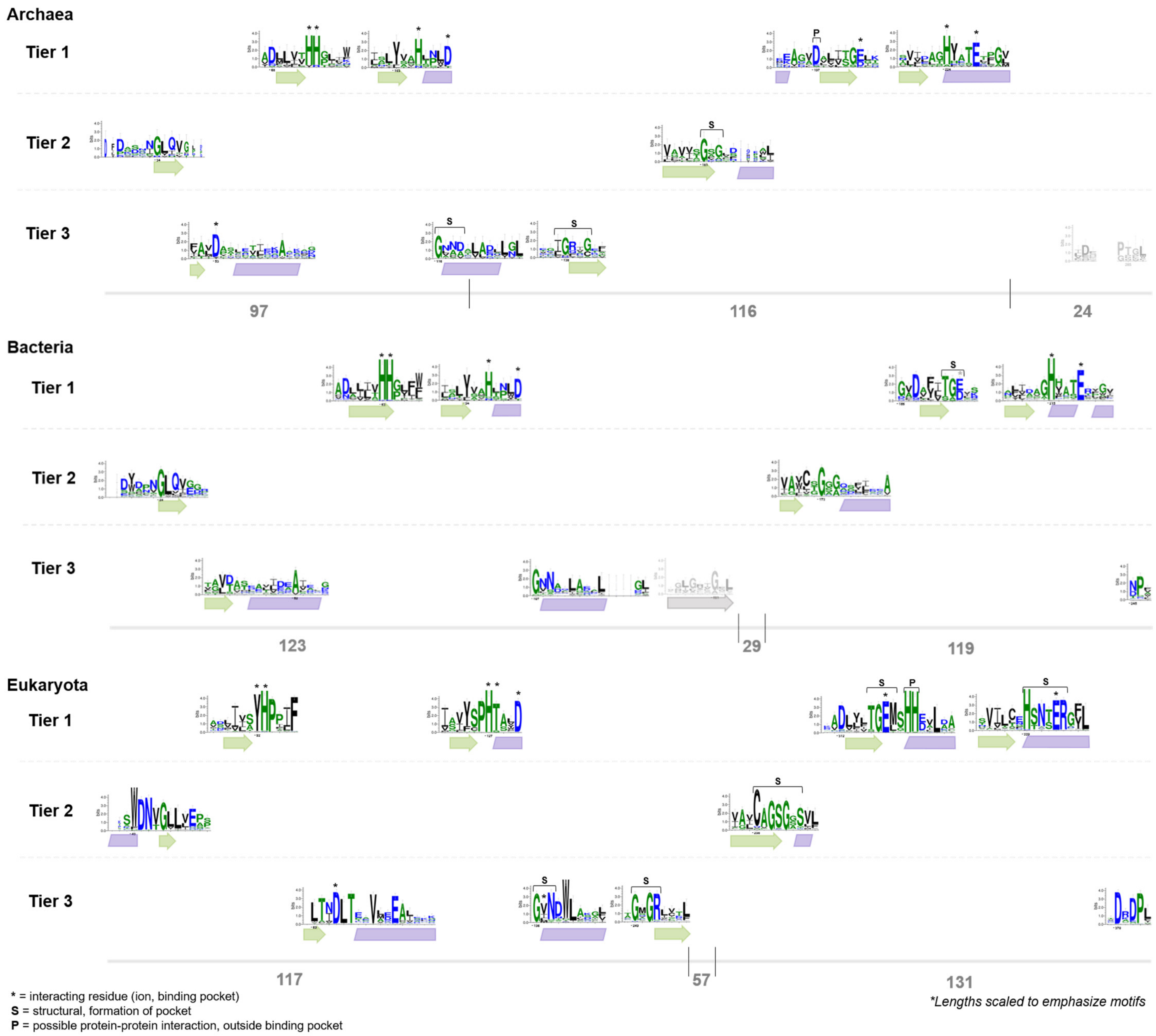

Sequences were aligned for each superkingdom and a multiple motif method was used to determine and compare family signatures. Web logos are organized vertically by tiers of decreasing relative conservation (1 being most and 3 being least highly conserved) and organized horizontally by relative location along the provided representative sequences. Tier 1 can be interpreted as containing the motifs of greatest "universal" importance, while tier 3 contains motifs most distinctive per respective superkingdom. Motifs shown in archaeal panel were grayed to indicate their absence from the seed sequence, MJ0927, of *M. jannaschii*. Motifs shown in bacterial panel were grayed to denote that they were not listed as conserved by ESPript analysis of bacterial sequences, but were determined to be notable when comparing motifs resolved across superkingdoms for this region. Diagram annotation: *asterisk denotes metal ion-binding; S denotes region of structural importance; P denotes region of putative protein-protein interaction.

Figure S4. (a) Structural alignment of *H. sapiens* NIF3L1 model (light blue; generated using Phyre2) and the YqfO-type DUF34 homolog of *B. cereus* (gray; PDB: 2GX8). Residues of identified as crucial to the dinuclear metal-binding pocket are shown as sticks, those of the NIF3L1 model distinguished in red. Spheres representing the presence of metal ions are

depicted in steel blue. Alignment binding sites were compared showing iron ions **(b)** in dark red and zinc ions **(c)** in turquoise. A tyrosine residue of NIF3L1 model replacing the first "H" of the aligned N-terminal histidine pair of YqfO is circled in yellow **(c)**. Just outside of the cleft of the NIF3L1 active site is a second histidine pair, which differs from the "YH" containing motif at this position in YqfO **(d)**. Adjacent of this pair is a tyrosine residue of YqfO (gray) that was found represented in visual duplicate, an artifact of crystallization that may also suggest a notable range of movement (yellow arrows) observable for the residue at this position. In NIF3L1, this residue is replaced by a serine preceding the histidine pair.

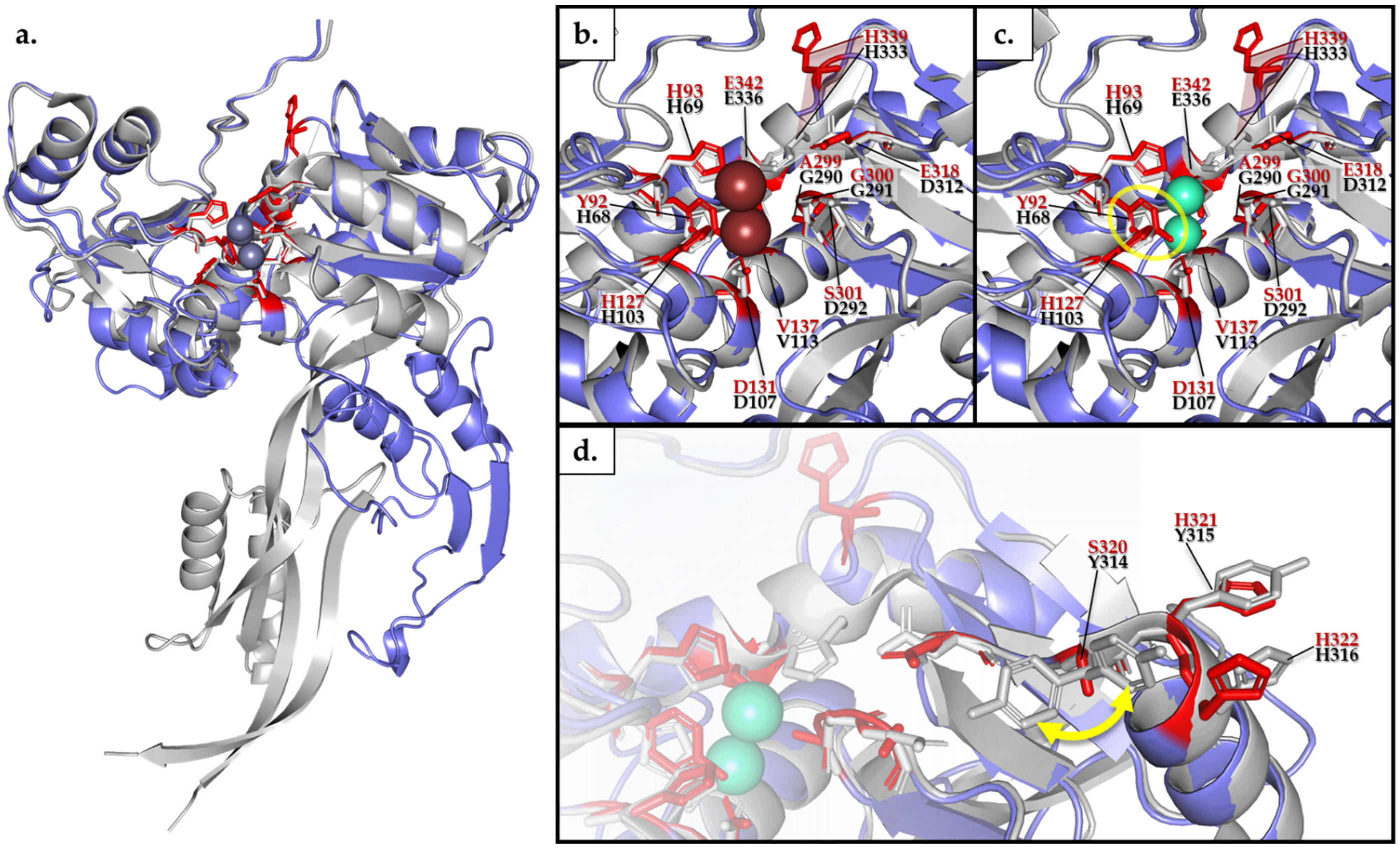

Figure S5. Histogram illustrating count per domain length range as a function of superkingdom. Color key denotes the 7 different ranges (bins) of inserted domain lengths.

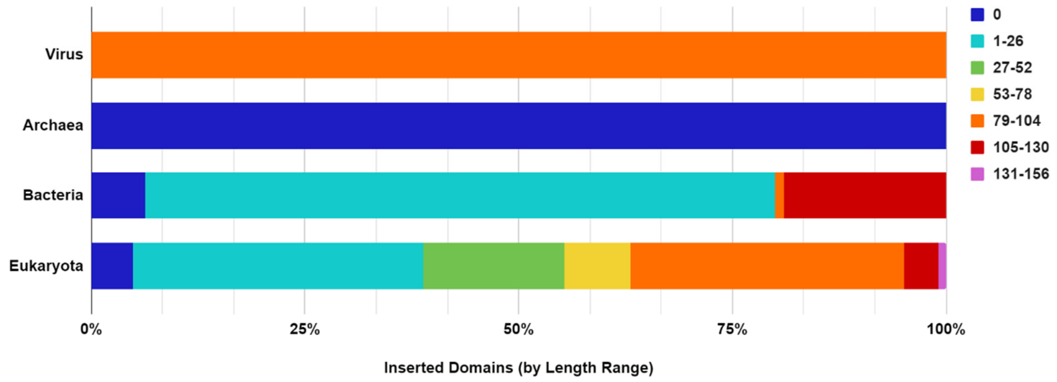

Figure S5. Sequence logos for subgroups A-C, D-G, and individual logos for A, B, C, D, E, F, and G. Alignments generated using MAFFT (E-INS-i; default settings) with default parameters for amino acid sequences. For **(a)** A-C, **(b)** D-G, and **(c)** E-G, approximate locations of the inserted domains are underlined in red.

a. Logo for all sequences of subgroups A-C (without the IPR015867 HMM profile signature).

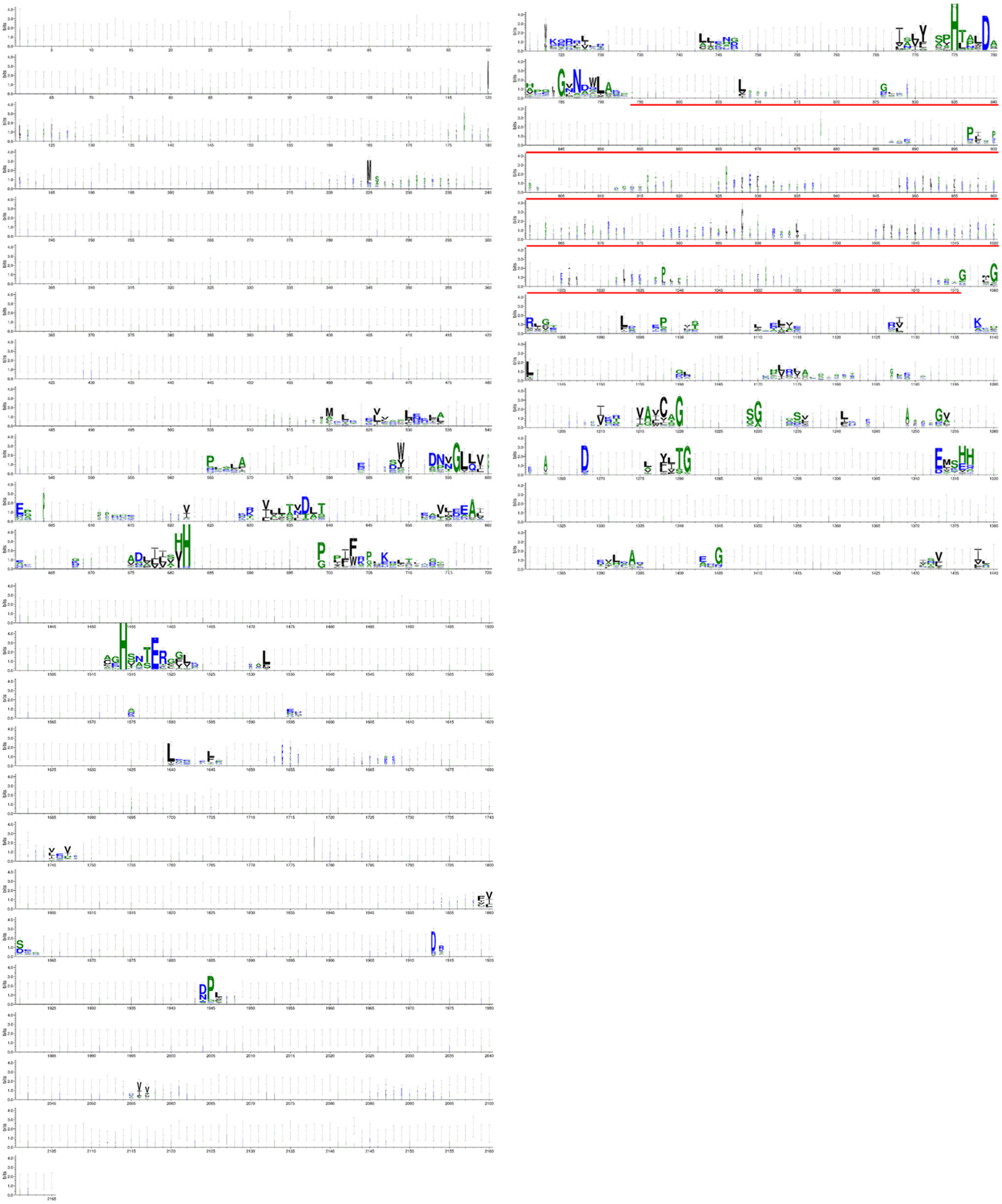

b. Logo for all sequences of subgroups D-G (IPR015867 HMM profile signature).

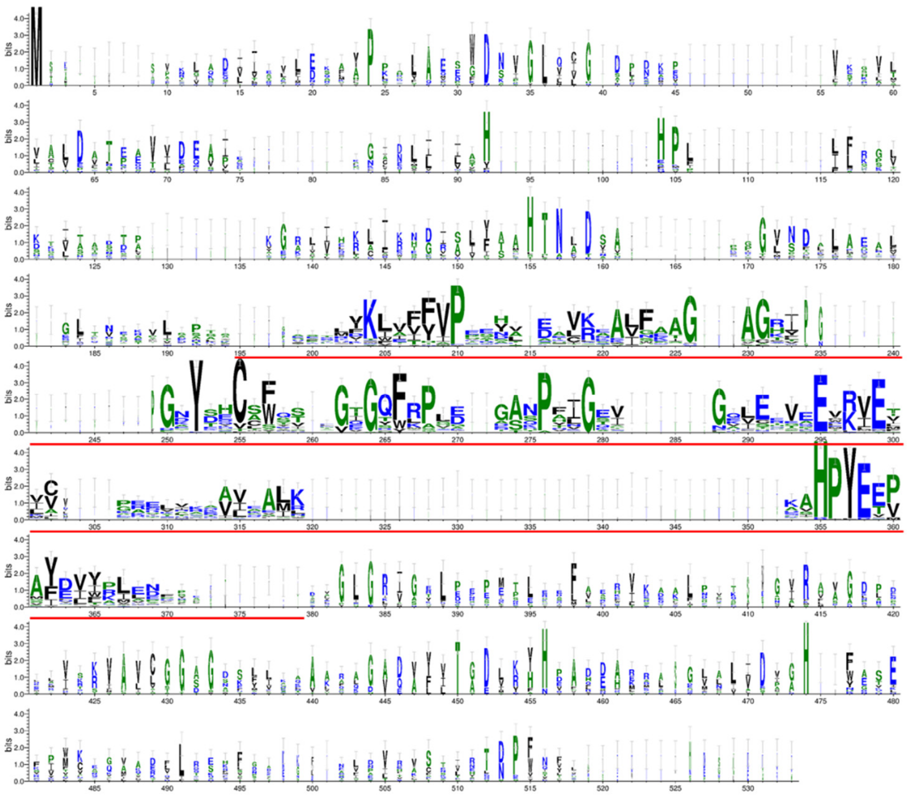

c. Sequence logo for subgroups E-G (without D, a set of free-standing YqfO/CutA1-like proteins that appear more frequently in Eukaryotes).

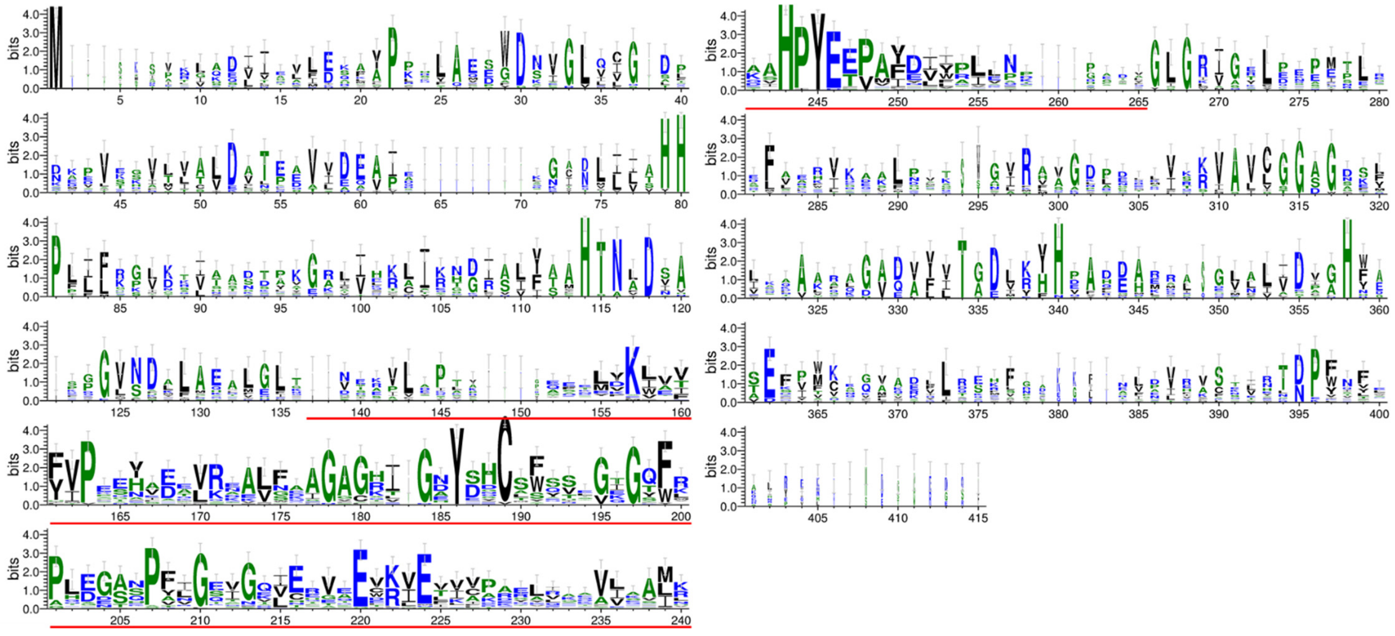

d. Sequence logo for subgroup A.

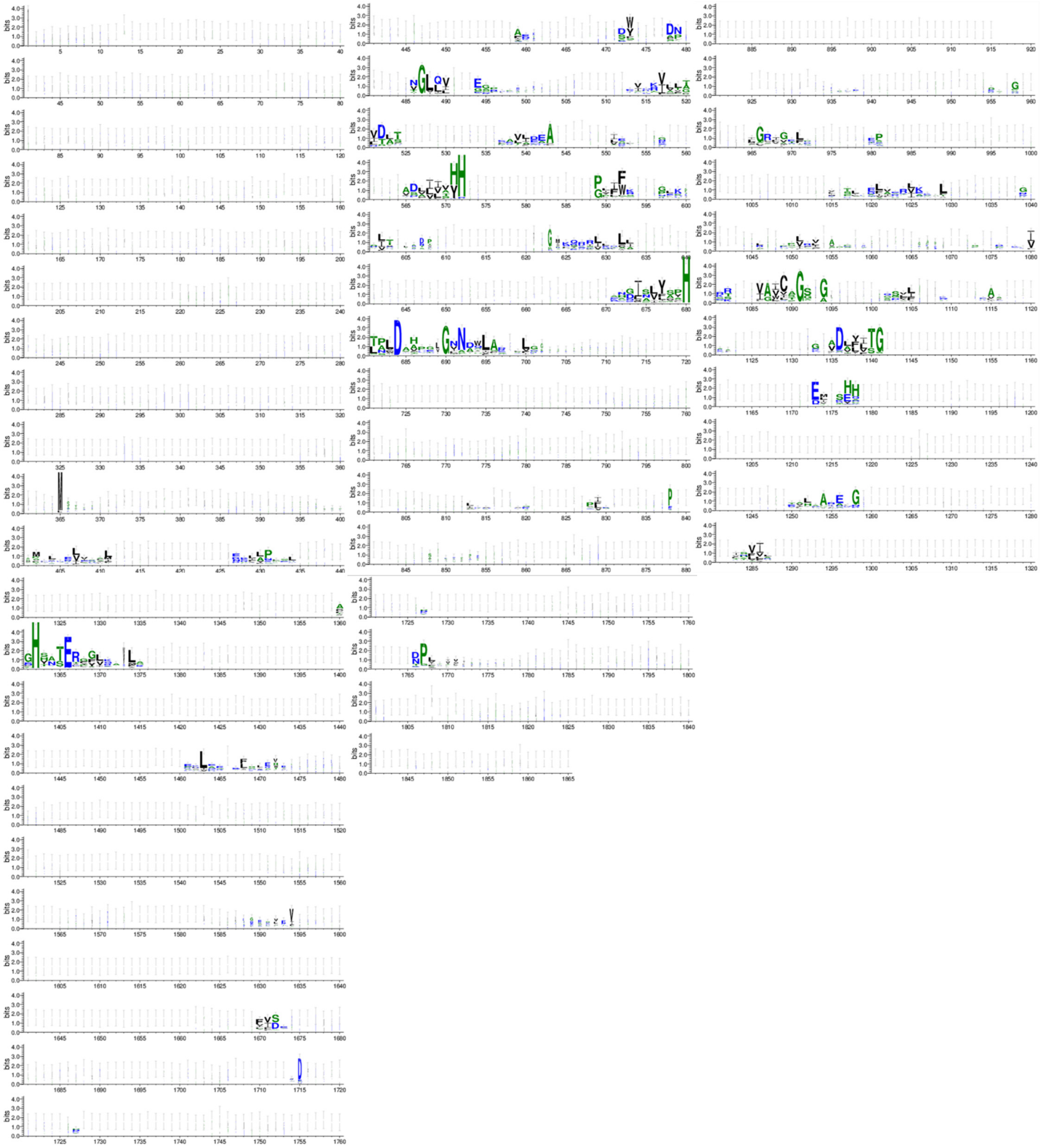

e. Sequence logo for subgroup B.

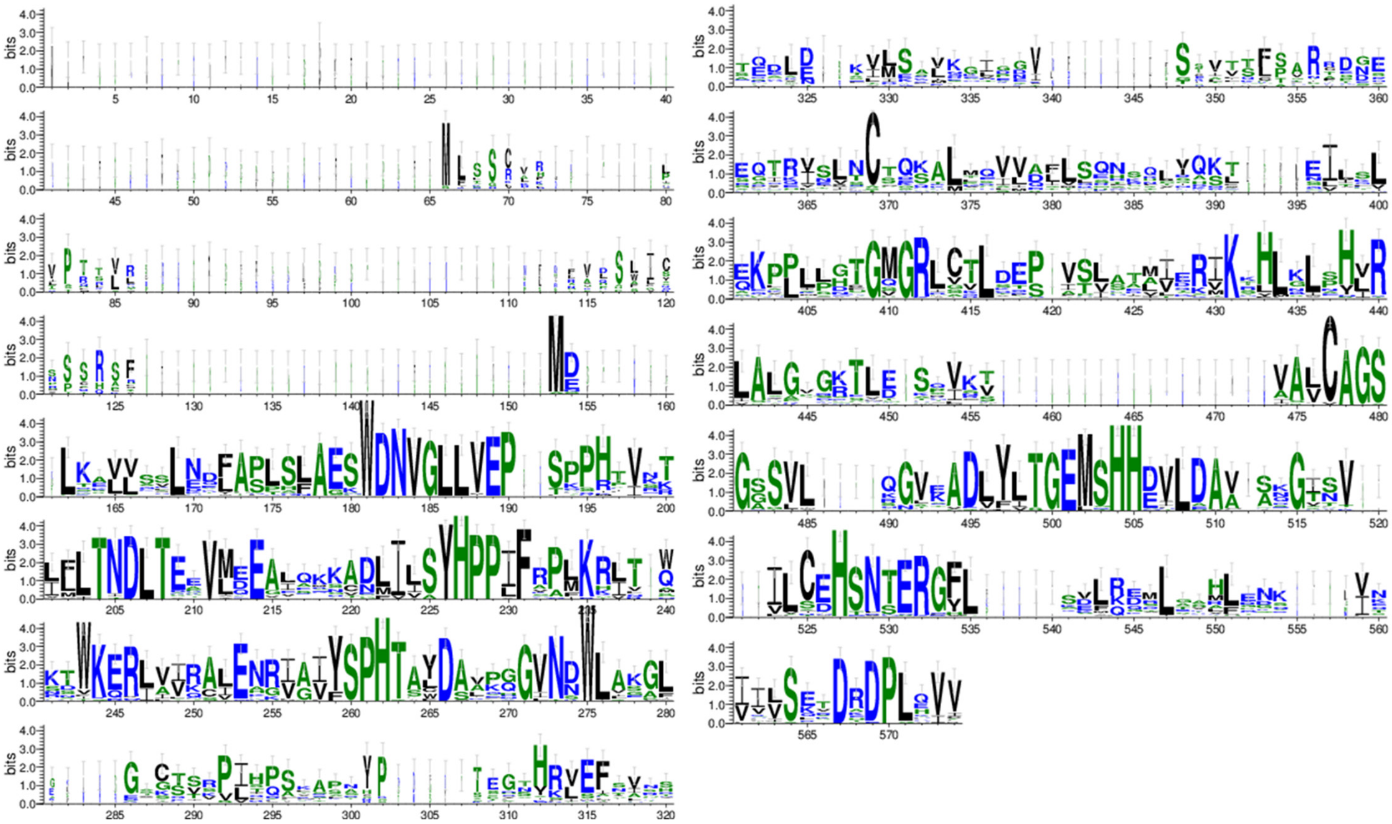

f. Sequence logo for subgroup C.

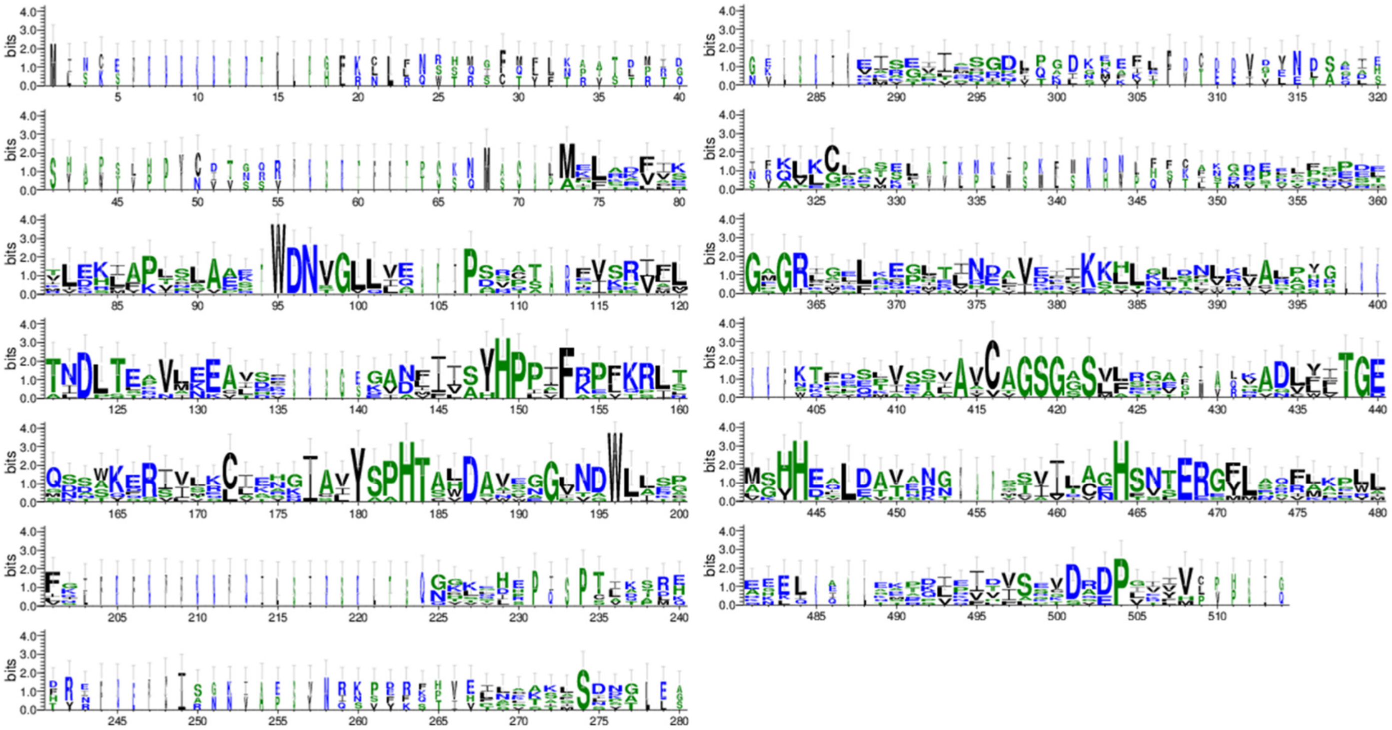

g. Sequence logo for subgroup D.

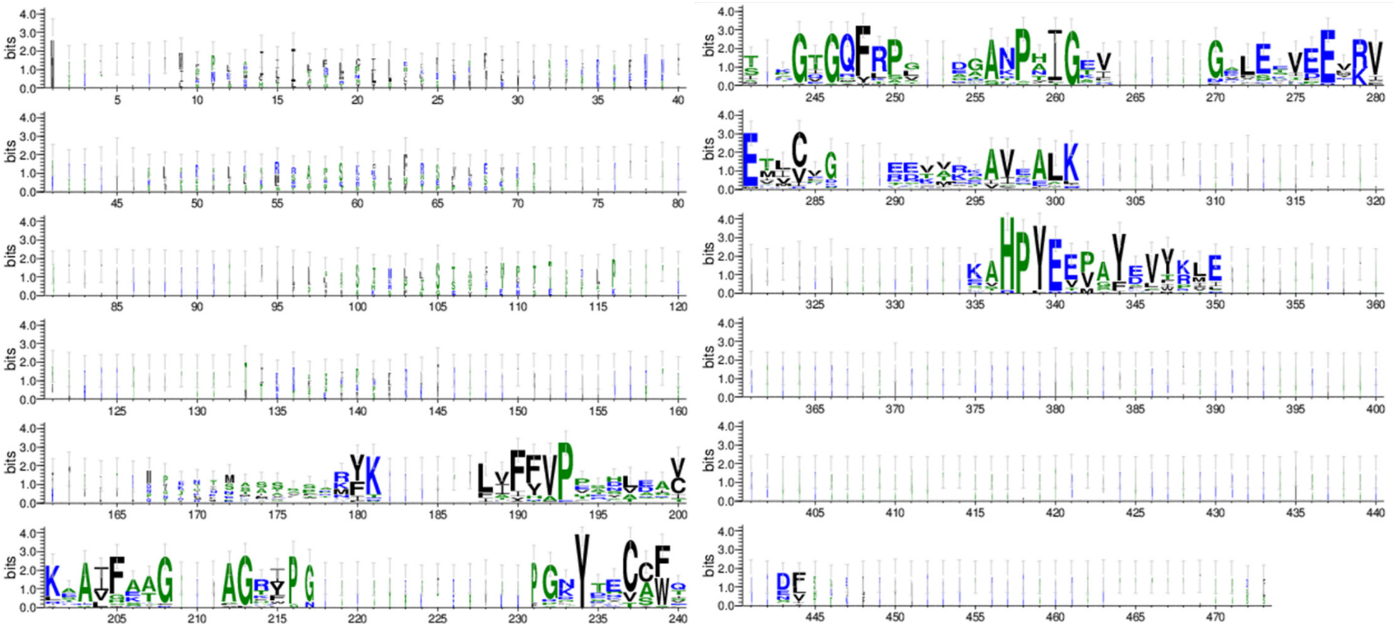

h. Sequence logo for subgroup E.

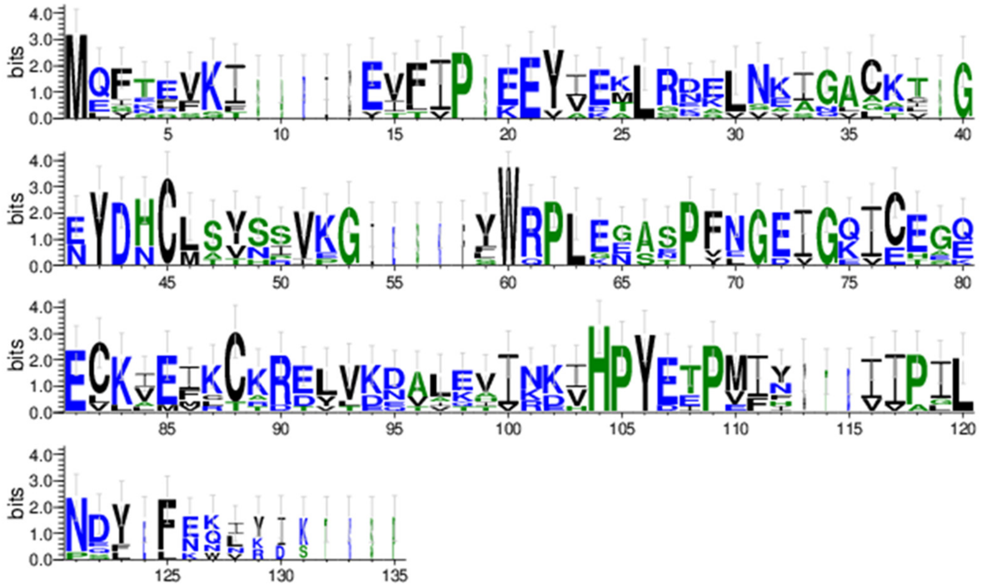

i. Sequence logo for subgroup F.

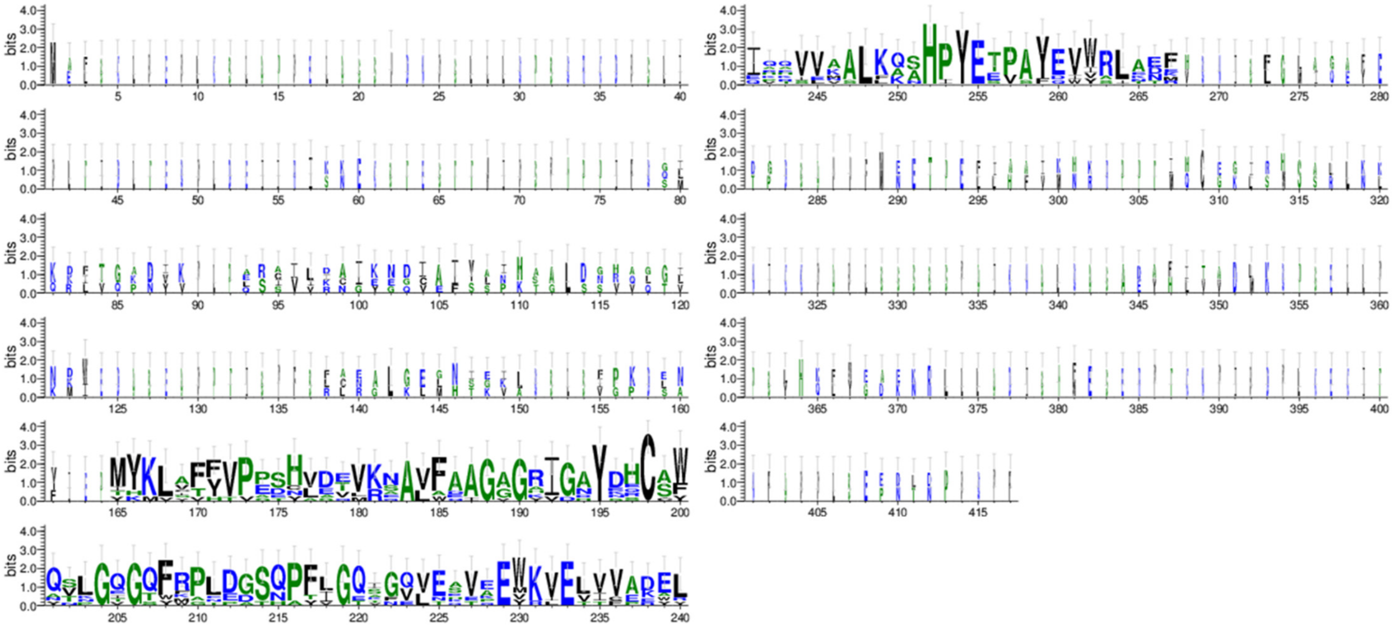

j. Sequence logo for subgroup G.

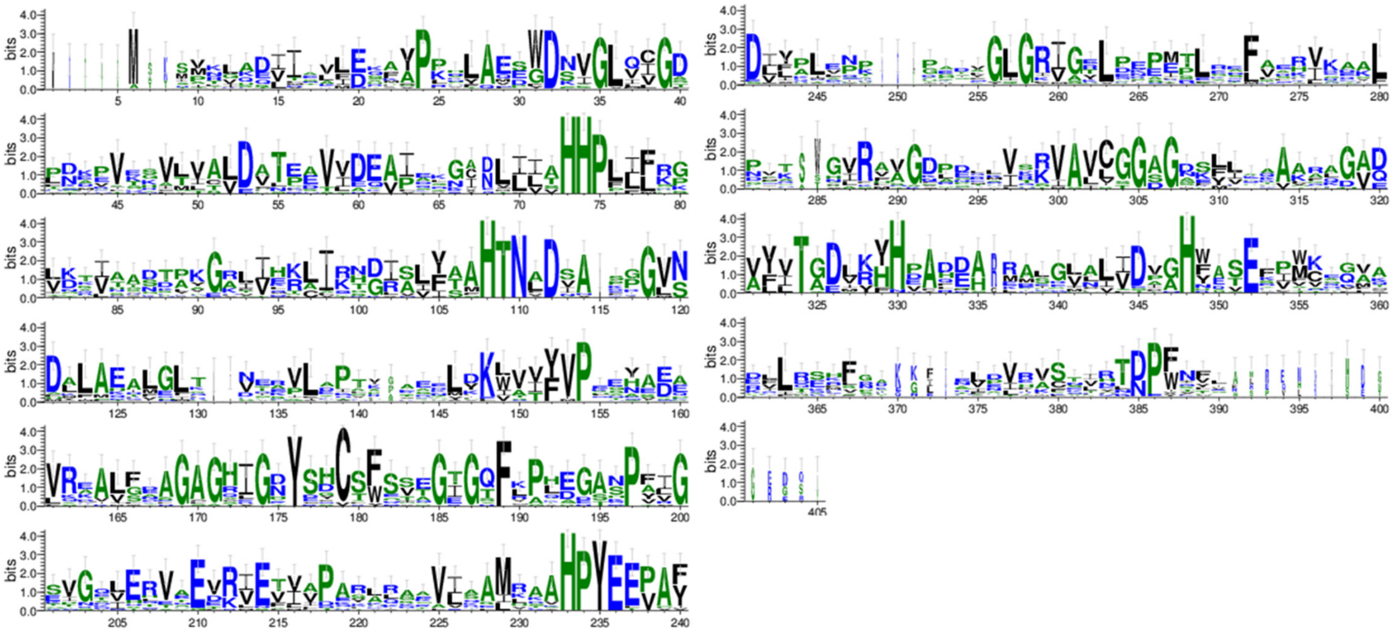

Figure S6. Pairwise alignments examining the identity relationships of predicted YqfO paralogs in *Bacillus cereus* (BC_4286, BC_2685). The evolutionary relationships between the paralogs and the CutA homologs of *Homo sapiens* (O60888) and *Escherichia coli* (P69488) are also explored.

a. Pairwise alignment of CDD-defined central inserted domain of YqfO-type DUF34 homolog (excerpt, COG3323) and the free-standing YqfO domain-containing protein, CutA of *B. cereus*. Alignment completed using COBALT (NBCI) (graphical coloring: BLOSUM45; alignment coloring: identity). Below, the excerpted domain of BC_4286 (Q818H0) is presented as Query_10001, while BC_2685 (Q81CR2) is Query_10002.

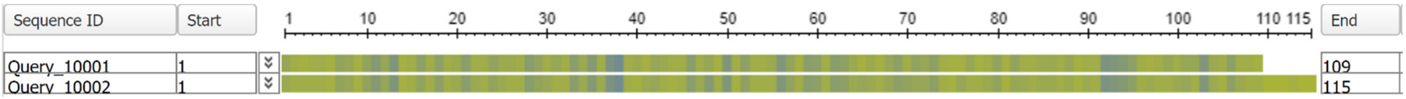


```
Query_10001  1    YAEEMKKVVVFVPVTHAEEVRKALGDAGAGHIGNYSHCTFSSEGTGTFVPQEGTNPYIGETGQLERVEEVRIETIIPASL  80
Query_10002  1    MQFTEVKIEVFIPEEYIETLRDELNKIGACKTGEYDHCLSYSSVKGYWRPLEEASPFNGEIGQICEGQECKVEIKCKREL  80

Query_10001  81   QRKVIKAMVTAHPYEEVAYDVYPLDNKGE------  109
Query_10002  81   VKDALEVINDIHPYETPMIYIIPILNDYFEKLYIK  115
```

b. Pairwise alignment of sequence BC_2685 (Q81CR2) shown as Query_10002, and full-length BC_4286 (Q818H0) shown as Query_10001.

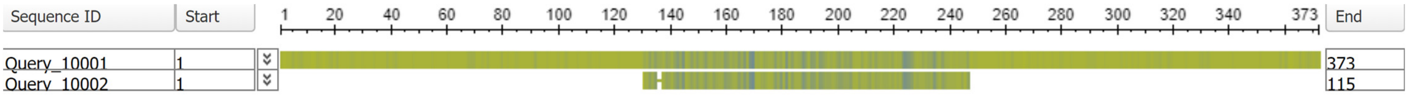


```
Query_10001  1    MSKIPNGHEIISLFESMYPKHLAMEGDKIGLQIGALNKPVRHVLIALDVTEEVVDEAIQLGANVIIAHHPLIFNPLKAIH  80
Query_10002       --------------------------------------------------------------------------------

Query_10001  81   TDKAYGKIIEKCIKNDIAIYAAHTNVDVAKGGVNDLLAEALGLQNTEVLAPTYAEEMKKVVVFVPVTHAEEVRKALGDAG  160
Query_10002  1    --------------------------------------------------MQFTE--VKIEVFIPEEYIETLRDELNKIG  28

Query_10001  161  AGHIGNYSHCTFSSEGTGTFVPQEGTNPYIGETGQLERVEEVRIETIIPASLQRKVIKAMVTAHPYEEVAYDVYPLDNKG  240
Query_10002  29   ACKTGEYDHCLSYSSVKGYWRPLEEASPFNGEIGQICEGQECKVEIKCKRELVKDALEVINDIHPYETPMIYIIPILNDY  108

Query_10001  241  ETLGLGKIGYLQEEMTLGQFAEHVKQSLDVKGARVVGKLDDKVRKVAVLGGDGNKYINQAKFKGADVYVTGDMYYHVAHD  320
Query_10002  109  FEKLYIK-------------------------------------------------------------------------  115

Query_10001  321  AMMLGLNIVDPGHNVEKVMKQGVQKQLQEKVDAKKLNVHIHASQLHTDPFIFV  373
Query_10002       -----------------------------------------------------
```

c. Pairwise alignment of sequences BC_4286 (Q818H0) and BC_2685 (Q81CR2) using EMBOSS Needle [348].

```
########################################
# Program: needle
# Rundate: Tue 22 Jun 2021 14:01:30
# Commandline: needle
#    -auto
#    -stdout
#    -asequence emboss_needle-I20210622-140129-0947-33301761-p2m.asequence
#    -bsequence emboss_needle-I20210622-140129-0947-33301761-p2m.bsequence
#    -datafile EBLOSUM62
#    -gapopen 10.0
#    -gapextend 0.5
#    -endopen 10.0
#    -endextend 0.5
#    -aformat3 pair
#    -sprotein1
#    -sprotein2
# Align_format: pair
# Report_file: stdout
########################################

#=======================================
#
# Aligned_sequences: 2
# 1: Q818H0_BACCR
# 2: Q81CR2_BACCR
# Matrix: EBLOSUM62
# Gap_penalty: 10.0
# Extend_penalty: 0.5
#
# Length: 374
# Identity:      38/374 (10.2%)
# Similarity:    59/374 (15.8%)
# Gaps:         260/374 (69.5%)
# Score: 137.0
#
#
#=======================================
```

```
Q818H0_BACCR       1 MSKIPNGHEIISLFESMYPKHLAMEGDKIGLQIGALNKPVRHVLIALDVT     50

Q81CR2_BACCR       1 --------------------------------------------------      0

Q818H0_BACCR      51 EEVVDEAIQLGANVIIAHHPLIFNPLKAIHTDKAYGKIIEKCIKNDIAIY    100

Q81CR2_BACCR       1 --------------------------------------------------      0

Q818H0_BACCR     101 AAHTNVDVAKGGVNDLLAEALGLQNTEVLAPTYAEEMKKVVVFVPVTHAE    150
                                           :|.|||          |:.||:|..:.|
Q81CR2_BACCR       1 ----------------------MQFTEV----------KIEVFIPEEYIE     18

Q818H0_BACCR     151 EVRKALGDAGAGHIGNYSHCTFSSEGTGTFVPQEGTNPYIGETGQLERVE    200
                     .:|..|...||...|.|.||...|...|.:.|.|..:|:.||.||:...:
Q81CR2_BACCR      19 TLRDELNKIGACKTGEYDHCLSYSSVKGYWRPLEEASPFNGEIGQICEGQ     68

Q818H0_BACCR     201 EVRIETIIPASLQRKVIKAMVTAHPYEEVAYDVYP-LDNKGETLGLGKIG    249
                     |.::|......|.:..::.:...||||.....:.| |::..|.|
Q81CR2_BACCR      69 ECKVEIKCKRELVKDALEVINDIHPYETPMIYIIPILNDYFEKL------    112

Q818H0_BACCR     250 YLQEEMTLGQFAEHVKQSLDVKGARVVGKLDDKVRKVAVLGGDGNKYINQ    299
                     |::
Q81CR2_BACCR     113 YIK-----------------------------------------------    115

Q818H0_BACCR     300 AKFKGADVYVTGDMYYHVAHDAMMLGLNIVDPGHNVEKVMKQGVQKQLQE    349

Q81CR2_BACCR     116 --------------------------------------------------    115

Q818H0_BACCR     350 KVDAKKLNVHIHASQLHTDPFIFV    373

Q81CR2_BACCR     116 ------------------------    115


#---------------------------------------
#---------------------------------------
```

d. Pairwise alignment using EMBOSS Matcher [348], a more rigorous algorithm compared to Needle for identifying select regions between sequences BC_4286 (Q818H0) and BC_2685 (Q81CR2) that may share homology. According to this and subsequent alignments **(d-f)**, BC_2685 (Q81CR2) of *B. cereus* demonstrates greater similarity to the human CutA homolog (O60888) than it does to that of *E. coli* (another bacterial CutA homolog) and shows greatest similarity to a paralogous region of the *B. cereus* DUF34 homolog, BC_4286 (Q818H0).

```
########################################
# Program: matcher
# Rundate: Tue 22 Jun 2021 14:00:46
# Commandline: matcher
#    -auto
#    -stdout
#    -asequence emboss_matcher-I20210622-140045-0087-7806619-p1m.asequence
#    -bsequence emboss_matcher-I20210622-140045-0087-7806619-p1m.bsequence
#    -datafile EBLOSUM62
#    -gapopen 14
#    -gapextend 4
#    -alternatives 1
#    -aformat3 pair
#    -sprotein1
#    -sprotein2
# Align_format: pair
# Report_file: stdout
########################################

#=======================================
#
# Aligned_sequences: 2
# 1: Q818H0_BACCR
# 2: Q81CR2_BACCR
# Matrix: EBLOSUM62
# Gap_penalty: 14
# Extend_penalty: 4
#
# Length: 100
# Identity:      31/100 (31.0%)
# Similarity:    48/100 (48.0%)
# Gaps:           0/100 ( 0.0%)
# Score: 141
#
#
#=======================================

Q818H0_BACCR     139 KVVVFVPVTHAEEVRKALGDAGAGHIGNYSHCTFSSEGTGTFVPQEGTNP    188
                     |:.||:|..:.|.:|..|...||...|.|.||...|...|.:.|.|..:|
Q81CR2_BACCR       7 KIEVFIPEEYIETLRDELNKIGACKTGEYDHCLSYSSVKGYWRPLEEASP     56

Q818H0_BACCR     189 YIGETGQLERVEEVRIETIIPASLQRKVIKAMVTAHPYEEVAYDVYPLDN    238
                     :.||.||:...:|.::|......|.:..::.:...||||.....:.|:.|
Q81CR2_BACCR      57 FNGEIGQICEGQECKVEIKCKRELVKDALEVINDIHPYETPMIYIIPILN    106


#---------------------------------------
#---------------------------------------
```

e. Pairwise alignment for CutA of *Homo sapiens* (O60888) and COG3323 paralog of *B. cereus*, BC_2685 (Q81CR2). Alignment completed using EMBOSS Matcher.

```
########################################
# Program: matcher
# Rundate: Tue 22 Jun 2021 17:06:14
# Commandline: matcher
#    -auto
#    -stdout
#    -asequence emboss_matcher-I20210622-171125-0386-37647102-p1m.asequence
#    -bsequence emboss_matcher-I20210622-171125-0386-37647102-p1m.bsequence
#    -datafile EBLOSUM62
#    -gapopen 14
#    -gapextend 4
#    -alternatives 1
#    -aformat3 pair
#    -sprotein1
#    -sprotein2
# Align_format: pair
# Report_file: stdout
########################################

#=======================================
#
# Aligned_sequences: 2
# 1: CUTA_HUMAN
# 2: Q81CR2_BACCR
# Matrix: EBLOSUM62
# Gap_penalty: 14
# Extend_penalty: 4
#
# Length: 51
# Identity:      15/51 (29.4%)
# Similarity:    24/51 (47.1%)
# Gaps:           0/51 ( 0.0%)
# Score: 68
#
#
#=======================================

CUTA_HUMAN       111 GKIEEDSEVLMMIKTQSSLVPALTDFVRSVHPYEVAEVIALPVEQGNFPY    160
                     |:|.|..|..:.||.:..||....:.:..:||||...:..:|:....|..
Q81CR2_BACCR      62 GQICEGQECKVEIKCKRELVKDALEVINDIHPYETPMIYIIPILNDYFEK    111

CUTA_HUMAN       161 L    161
                     |
Q81CR2_BACCR     112 L    112


#---------------------------------------
#---------------------------------------
```

f. Pairwise alignment for CutA of *E. coli* (P69488) and COG3323 paralog of *B. cereus*, BC_2685 (Q81CR2). Alignment completed using EMBOSS Matcher.

```
########################################
# Program: matcher
# Rundate: Tue 22 Jun 2021 17:15:43
# Commandline: matcher
#    -auto
#    -stdout
#    -asequence emboss_matcher-I20210622-171540-0452-65331541-p2m.asequence
#    -bsequence emboss_matcher-I20210622-171540-0452-65331541-p2m.bsequence
#    -datafile EBLOSUM62
#    -gapopen 14
#    -gapextend 4
#    -alternatives 1
#    -aformat3 pair
#    -sprotein1
#    -sprotein2
# Align_format: pair
# Report_file: stdout
########################################

#=======================================
#
# Aligned_sequences: 2
# 1: CUTA_ECOLI
# 2: Q81CR2_BACCR
# Matrix: EBLOSUM62
# Gap_penalty: 14
# Extend_penalty: 4
#
# Length: 43
# Identity:      11/43 (25.6%)
# Similarity:    24/43 (55.8%)
# Gaps:           0/43 ( 0.0%)
# Score: 59
#
#
#=======================================
```

```
CUTA_ECOLI        54 GKLEQEYEVQMILKTTVSHQQALLECLKSHHPYQTPELLVLPV     96
                     |::.:..|.::.:|......:..||.:...|||:||.:.::|:
Q81CR2_BACCR      62 GQICEGQECKVEIKCKRELVKDALEVINDIHPYETPMIYIIPI    104


#---------------------------------------
#---------------------------------------
```

g. COBALT alignment of CutA homologs for *H. sapiens* (O60888; label= Query_10001), *E. coli* (P69488; label= Query_10002), *B. cereus* (Q81CR2; label= Query_10003), and the YqfO-type DUF34 homolog of *B. cereus* (Q818H0; label= Query_10004). The IPR015867-characteristic motif, "HPYE", is highlighted by a yellow rectangle, emphasizing the single-residue distinction in this motif observable for the CutA homolog of *E. coli*.

```
Query_10001  1        MSGGRAPAVLLGGVASLLLS[6]LLPVASRLLLLPRVLLTMA[10]ASDSGSGYVpgSVSAAFVTCPNEKVAKEIA  85
Query_10002  1        --------------------   ------------------    MLDEKSSNT--ASVVVLCTAPDEATAQDLA  28
Query_10003  1        --------------------   -----------MQFTE--V    ------------KIEVFIPEEYIETLRDEL  24
Query_10004  1    [99]YAAHTNVDVAKGGVNDLLAE   ALGLQNTEVLAPTYAEEMK    ------------KVVVFVPVTHAEEVRKAL  156

Query_10001  86   RAVVEKRLAACVNLIPQITSIYEWK------------GKIEEDSEVLMMIKTQSSLVPALTDFVRSVHPYEVAEVIALPV  153
Query_10002  29   AKVLAEKLAACATLIPGATSLYYWE------------GKLEQEYEVQMILKTTVSHQQALLECLKSHHPYQTPELLVLPV  96
Query_10003  25   NKIGACKTGEYDHCLSYSSVKGYWRPLEEASPFNGEIGQICEGQECKVEIKCKRELVKDALEVINDIHPYETPMIYIIPI  104
Query_10004  157  GDAGAGHIGNYSHCTFSSEGTGTFVPQEGTNPYIGETGQLERVEEVRIETIIPASLQRKVIKAMVTAHPYEEVAYDVYPL  236

Query_10001  154  EQGNFPYLQWVRQVTESVSDSITVLP        179
Query_10002  97   THGDTDYLSWLNASLR----------        112
Query_10003  105  LN---DYFEKLYIK------------        115
Query_10004  237  DN---KGETLGLGKIGYLQEEMTLGQ[114]   373
```

Figure S7.
a.

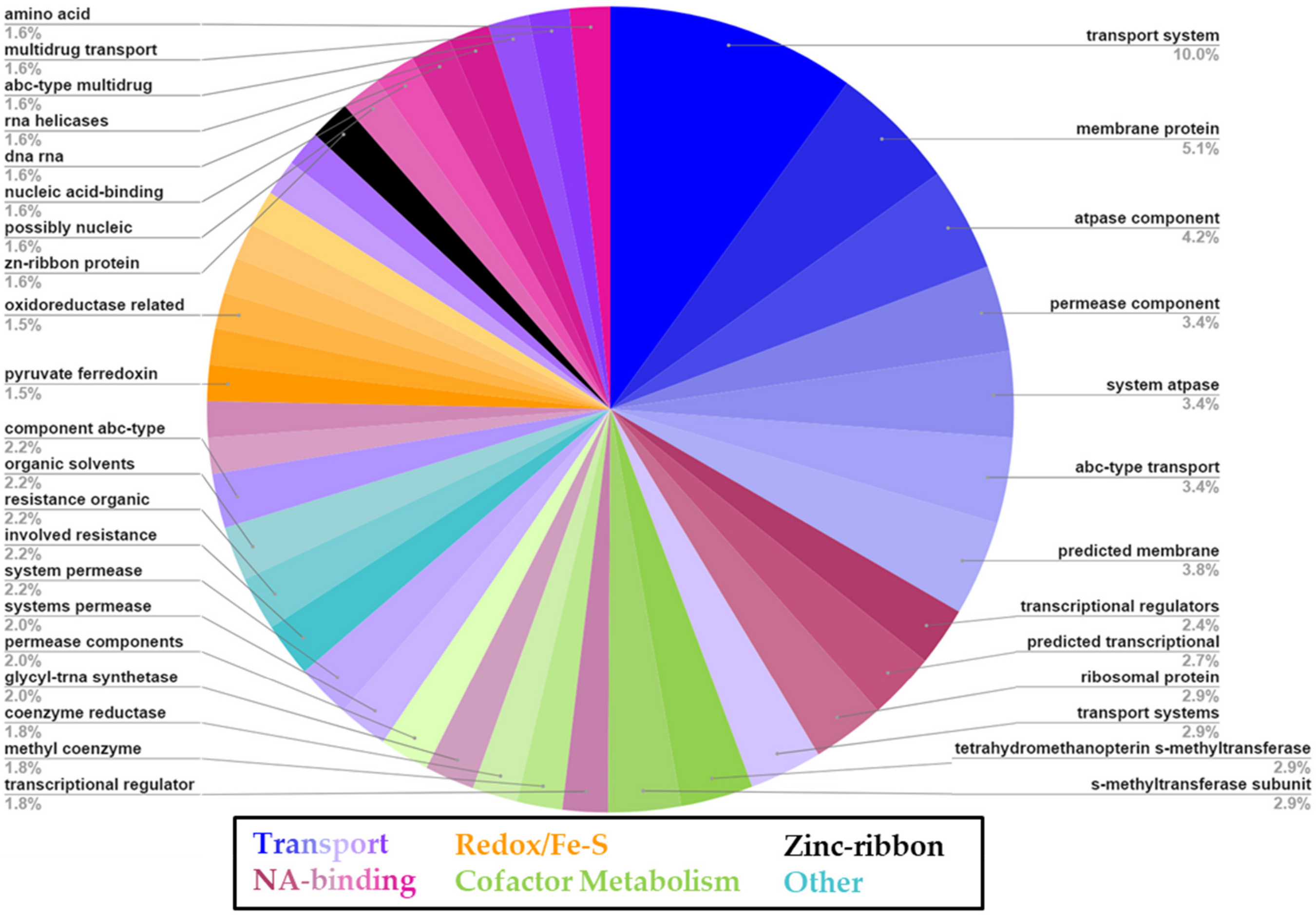
amino acid
1.6%
multidrug transport
1.6%
abc-type multidrug
1.6%
rna helicases
1.6%
dna rna
1.6%
nucleic acid-binding
1.6%
possibly nucleic
1.6%
zn-ribbon protein
1.6%
oxidoreductase related
1.5%
pyruvate ferredoxin
1.5%
component abc-type
2.2%
organic solvents
2.2%
resistance organic
2.2%
involved resistance
2.2%
system permease
2.2%
systems permease
2.0%
permease components
2.0%
glycyl-trna synthetase
2.0%
coenzyme reductase
1.8%
methyl coenzyme
1.8%
transcriptional regulator
1.8%
transport system
10.0%
membrane protein
5.1%
atpase component
4.2%
permease component
3.4%
system atpase
3.4%
abc-type transport
3.4%
predicted membrane
3.8%
transcriptional regulators
2.4%
predicted transcriptional
2.7%
ribosomal protein
2.9%
transport systems
2.9%
tetrahydromethanopterin s-methyltransferase
2.9%
s-methyltransferase subunit
2.9%
Transport
Redox/Fe-S
Zinc-ribbon
NA-binding
Cofactor Metabolism
Other

b-c.

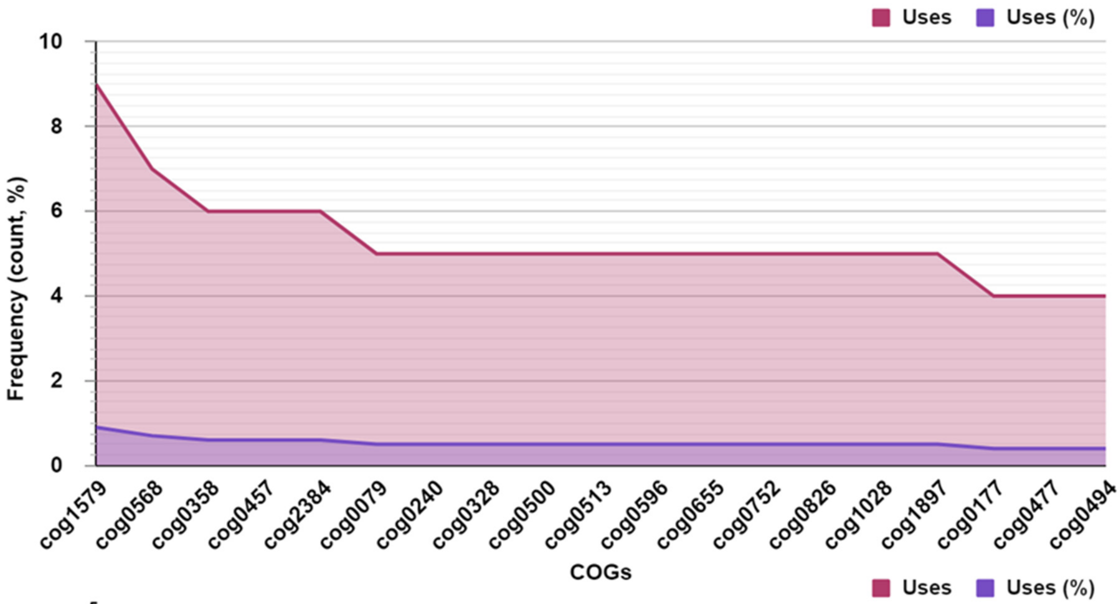

Uses
Uses (%)
Frequency (count, %)
10
8
6
4
2
0
cog1579
cog0568
cog0358
cog0457
cog2384
cog0079
cog0240
cog0328
cog0500
cog0513
cog0596
cog0655
cog0752
cog0826
cog1028
cog1897
cog0177
cog0477
cog0494
COGs

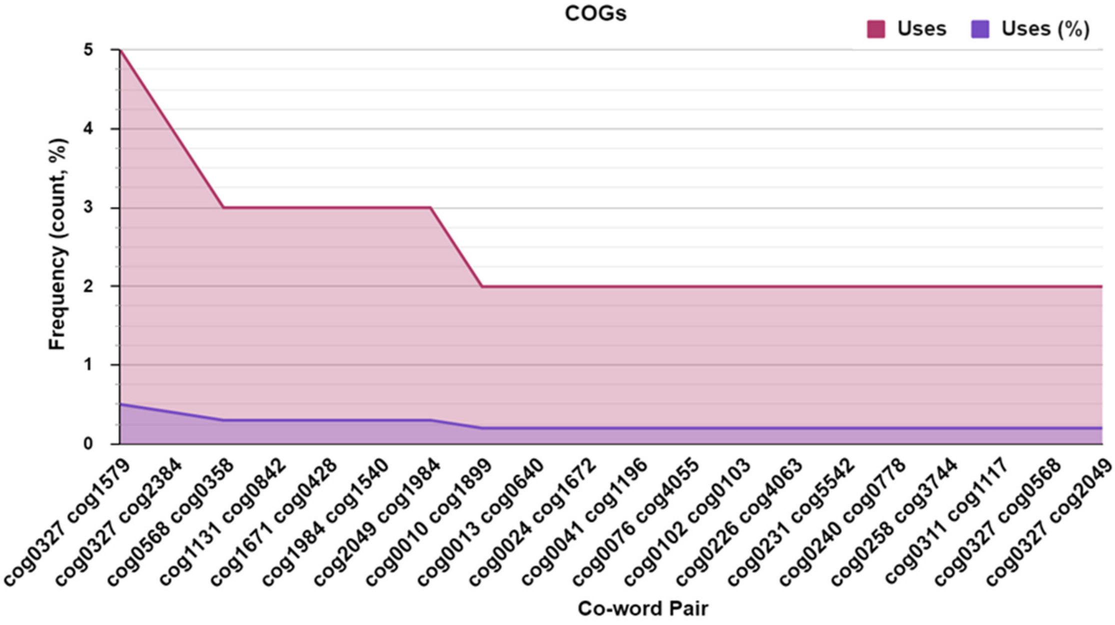

Uses
Uses (%)
Frequency (count, %)
5
4
3
2
1
0
cog0327 cog1579
cog0327 cog2384
cog0568 cog0358
cog1131 cog0842
cog1671 cog0428
cog1984 cog1540
cog2049 cog1984
cog0010 cog1899
cog0013 cog0640
cog0024 cog1672
cog0041 cog1196
cog0076 cog4055
cog0102 cog0103
cog0226 cog4063
cog0231 cog5542
cog0240 cog0778
cog0258 cog3744
cog0311 cog1117
cog0327 cog0568
cog0327 cog2049
Co-word Pair

Figure S8.

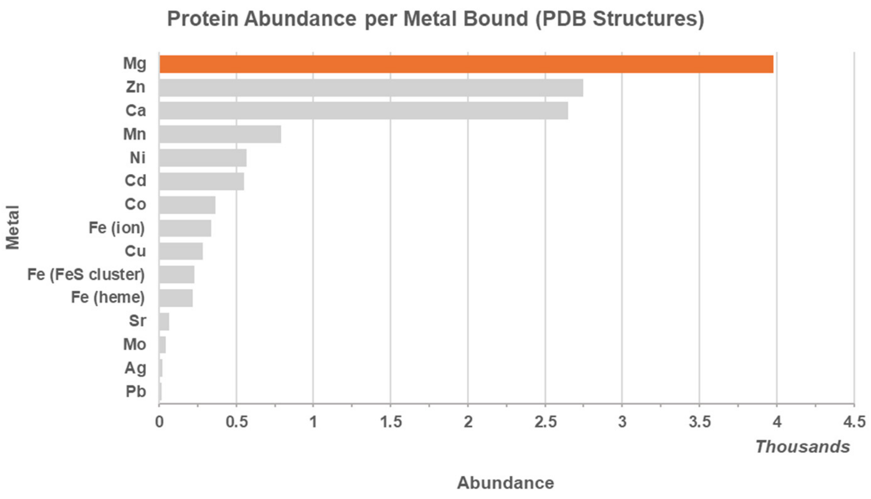

Protein Abundance per Metal Bound (PDB Structures)
Metal
Mg
Zn
Ca
Mn
Ni
Cd
Co
Fe (ion)
Cu
Fe (FeS cluster)
Fe (heme)
Sr
Mo
Ag
Pb
0
0.5
1
1.5
2
2.5
3
3.5
4
4.5
Thousands
Abundance

Figure S9.

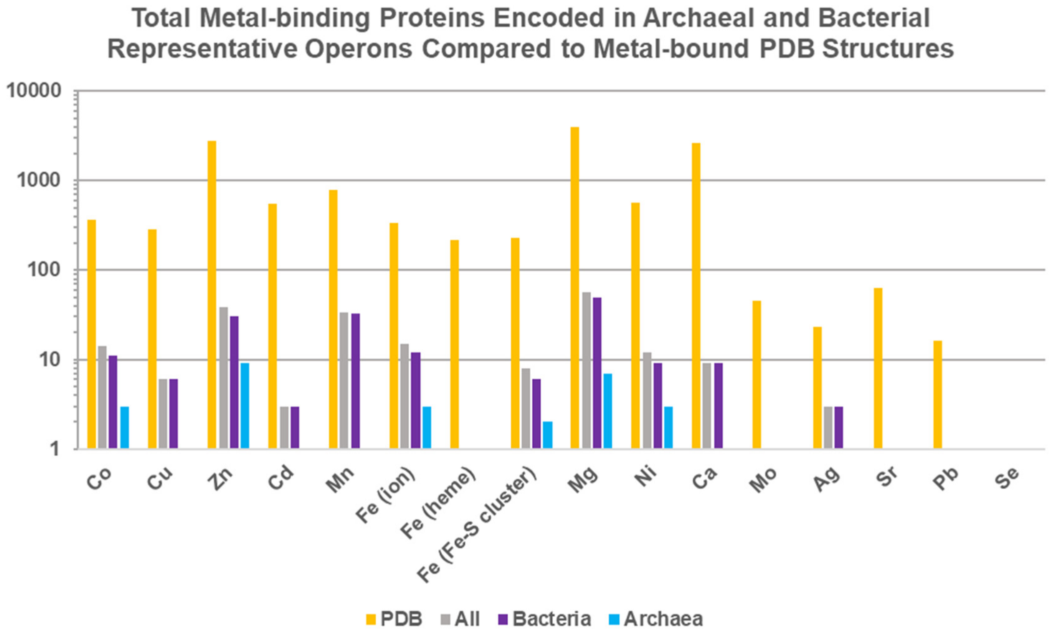

Figure S10.

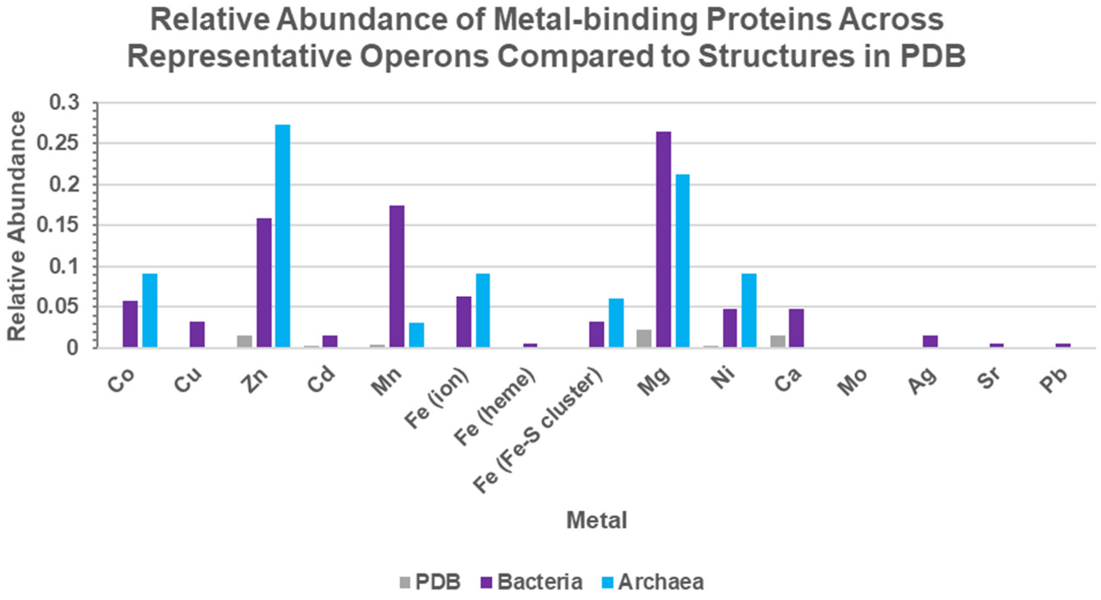

Figure S11.

a.

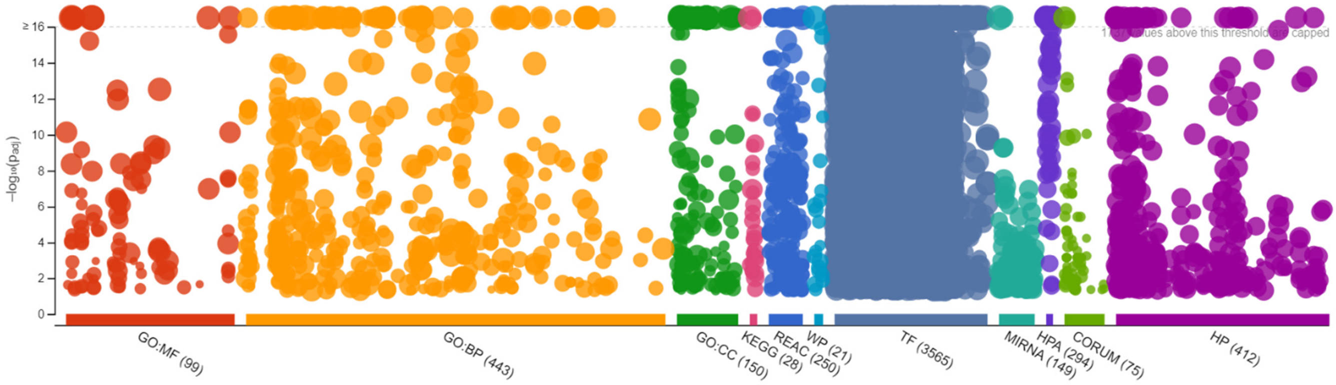

**version**	e102_eg49_p15_7a9b4d6
**date**	4/13/2021, 10:13:25 AM
**organism**	hsapiens

g:Profiler

b.

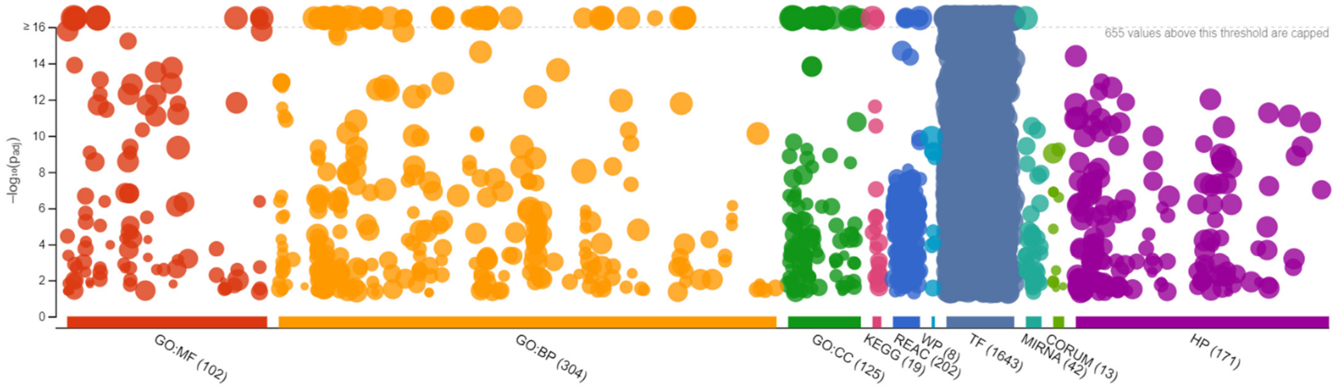

**version**	e102_eg49_p15_7a9b4d6
**date**	4/13/2021, 10:23:34 AM
**organism**	mmusculus

g:Profiler

c.

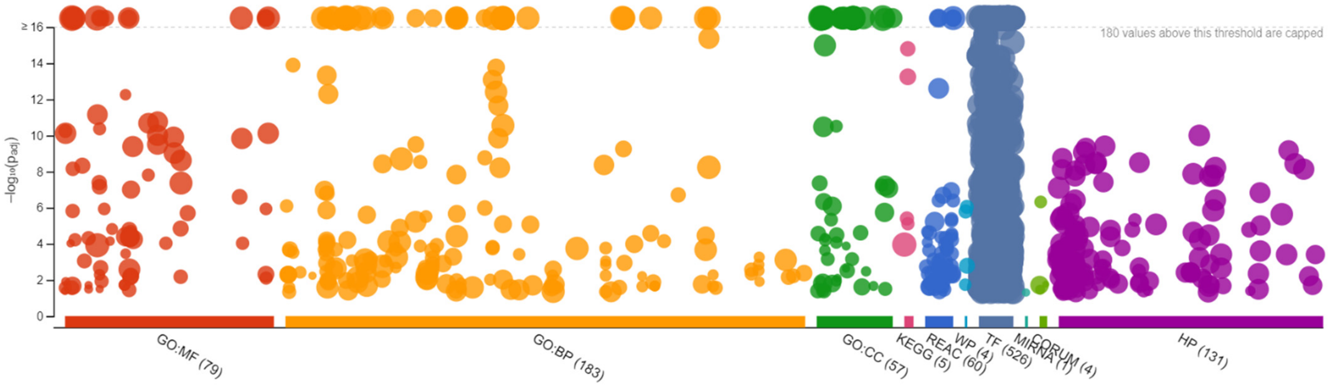

**version**	e102_eg49_p15_7a9b4d6
**date**	4/13/2021, 10:31:17 AM
**organism**	rnorvegicus

g:Profiler

d.

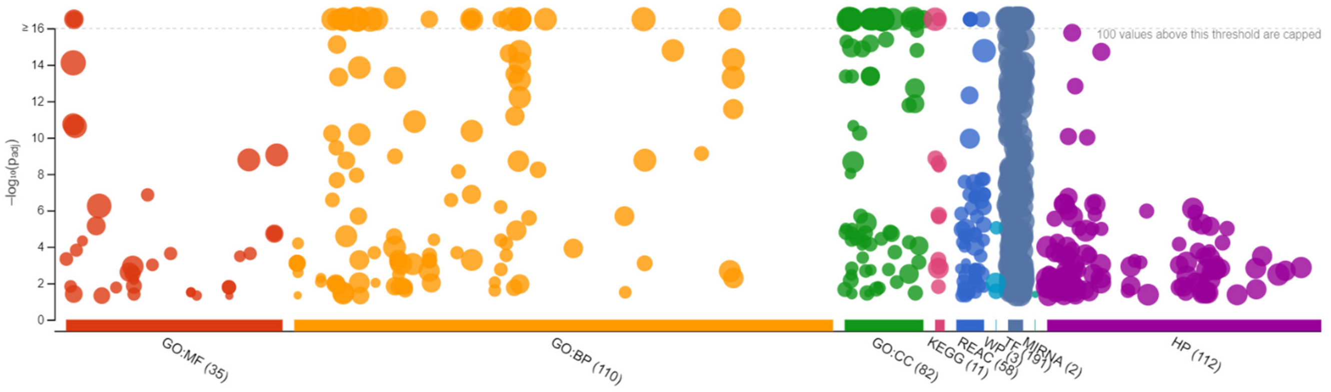

version e102_eg49_p15_7a9b4d6
date 4/13/2021, 10:39:42 AM
organism ggallus

g:Profiler

e.

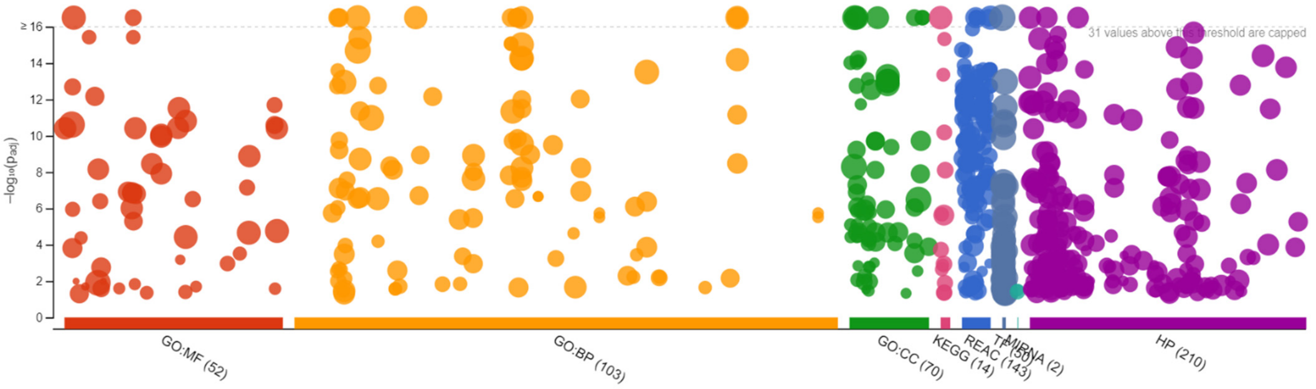

version e102_eg49_p15_7a9b4d6
date 4/13/2021, 10:46:33 AM
organism drerio

g:Profiler

f.

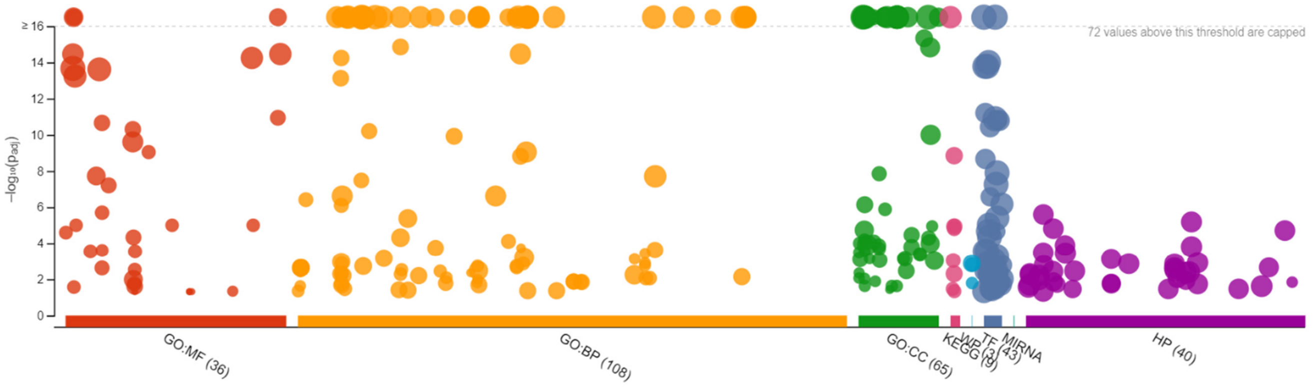

version e102_eg49_p15_7a9b4d6
date 4/13/2021, 10:51:49 AM
organism dmelanogaster

g:Profiler

g.

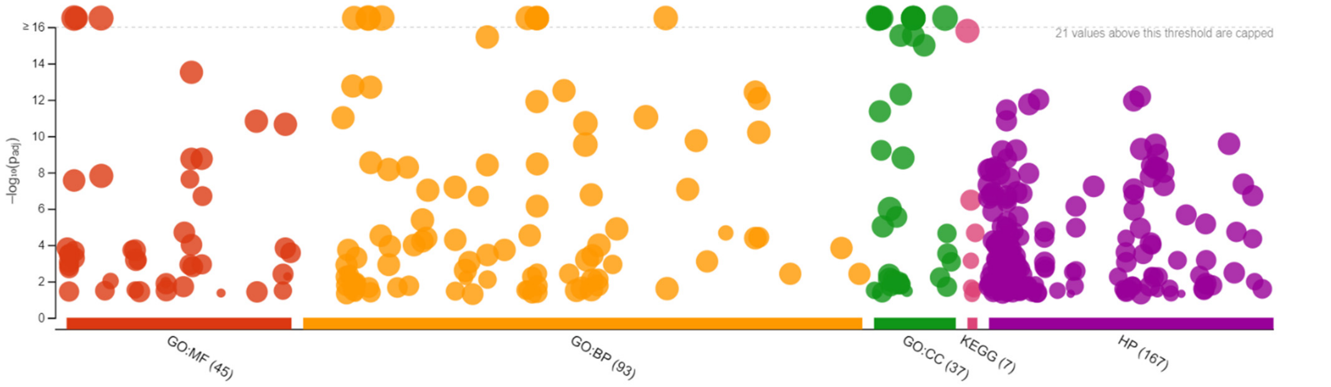

**version**	e102_eg49_p15_7a9b4d6
**date**	4/13/2021, 10:54:53 AM
**organism**	mmulatta

g:Profiler

h.

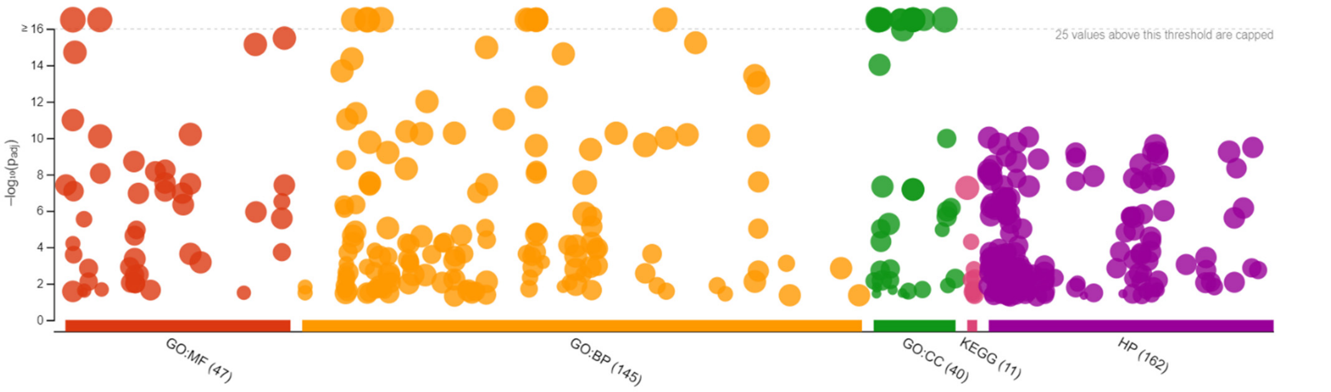

**version**	e102_eg49_p15_7a9b4d6
**date**	4/13/2021, 11:03:56 AM
**organism**	clfamiliaris

g:Profiler

i.

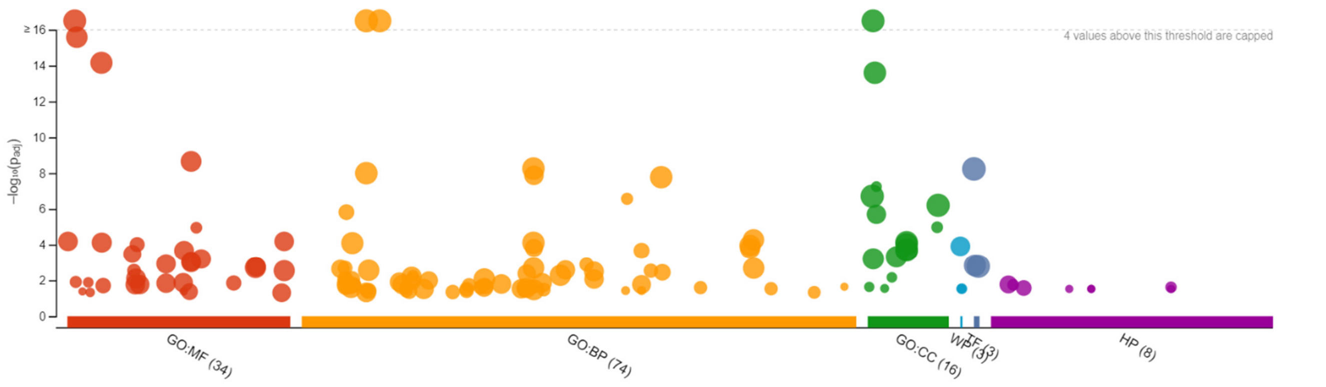

**version**	e102_eg49_p15_7a9b4d6
**date**	4/13/2021, 12:01:49 PM
**organism**	scerevisiae

g:Profiler

j.

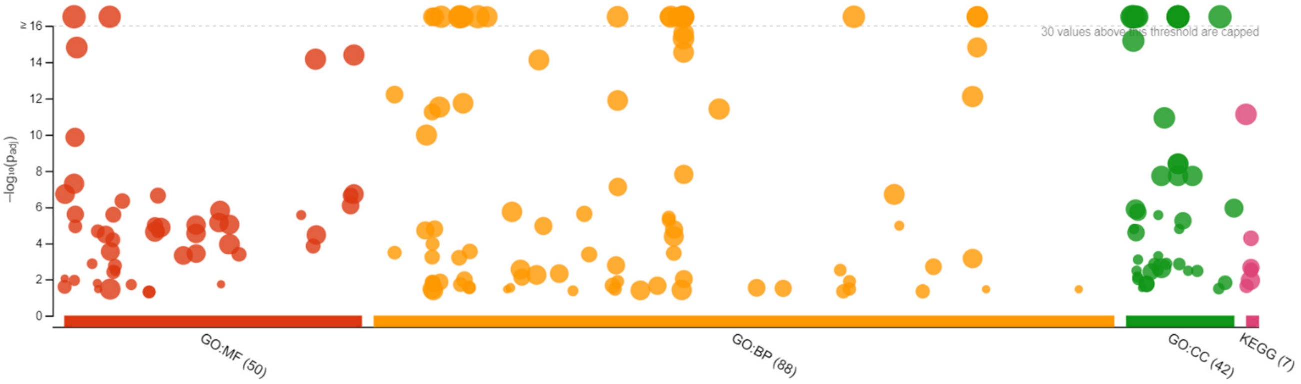

**version**	e102_eg49_p15_7a9b4d6
**date**	4/13/2021, 11:53:17 AM
**organism**	spombe

g:Profiler

Figure S12. STRING network of GSEA output of DUF34 co-regulated genes of *H. sapiens* (ProteomeHD).

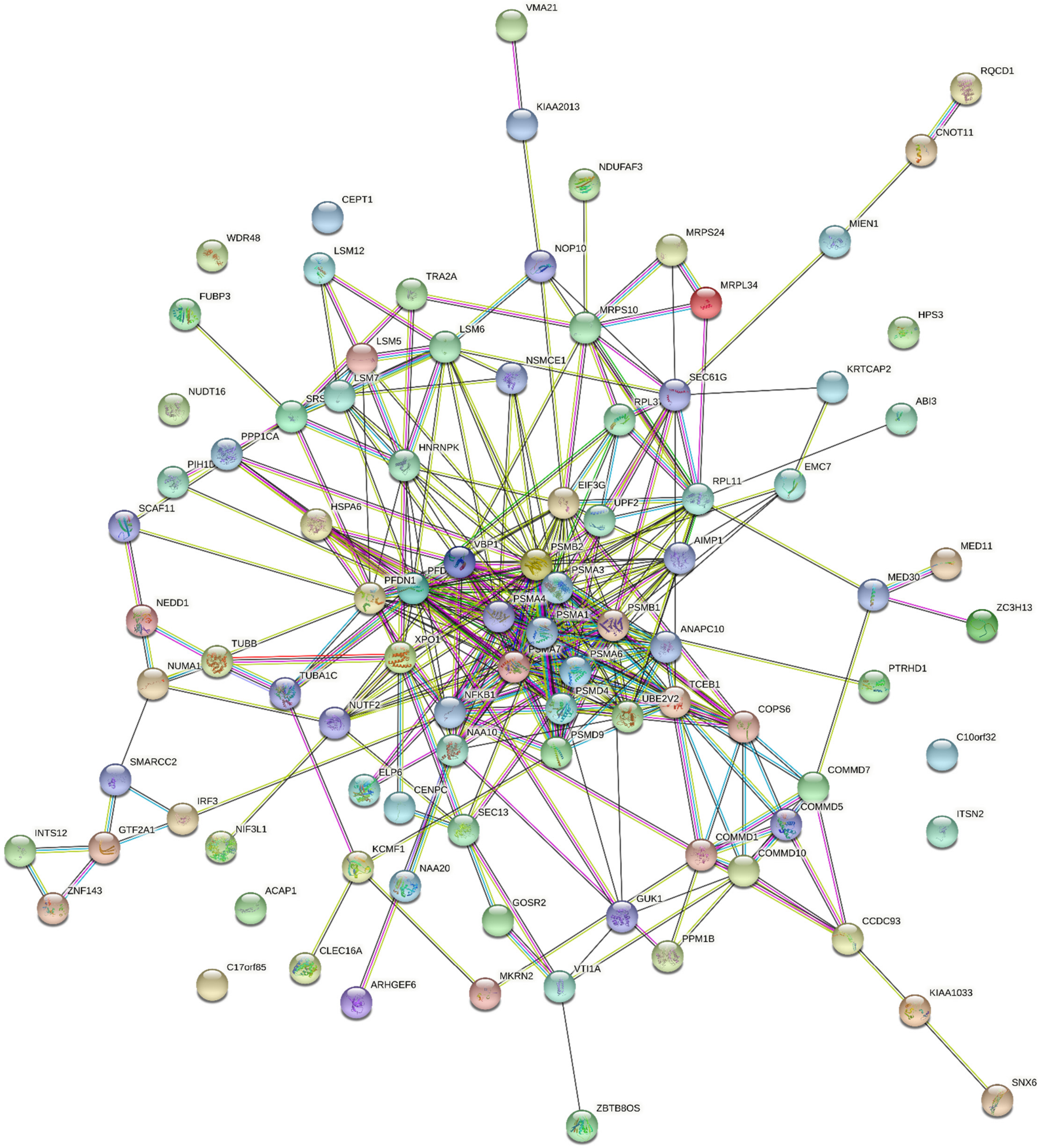

Supplemental References

[1] I. Sillitoe *et al.*, "CATH: expanding the horizons of structure-based functional annotations for genome sequences," *Nucleic Acids Res.*, vol. 47, no. D1, pp. D280–D284, Jan. 2019.

[2] F. Gabler *et al.*, "Protein Sequence Analysis Using the MPI Bioinformatics Toolkit," *Curr. Protoc. Bioinforma.*, vol. 72, no. 1, pp. 1–30, Dec. 2020.

[3] M. N. Price and A. P. Arkin, "PaperBLAST: Text Mining Papers for Information about Homologs," *mSystems*, vol. 2, no. 4, pp. 1–10, Aug. 2017.

[4] S. Lu *et al.*, "CDD/SPARCLE: the conserved domain database in 2020," *Nucleic Acids Res.*, vol. 48, no. D1, pp. D265–D268, Jan. 2020.

[5] L. Jeske, S. Placzek, I. Schomburg, A. Chang, and D. Schomburg, "BRENDA in 2019: a European ELIXIR core data resource," *Nucleic Acids Res.*, vol. 47, no. D1, pp. D542–D549, Jan. 2019.

[6] D. R. Armstrong *et al.*, "PDBe: Improved findability of macromolecular structure data in the PDB," *Nucleic Acids Res.*, vol. 48, no. D1, pp. D335–D343, 2020.

[7] S. K. Burley *et al.*, "RCSB Protein Data Bank: biological macromolecular structures enabling research and education in fundamental biology, biomedicine, biotechnology and energy," *Nucleic Acids Res.*, vol. 47, no. D1, pp. D464–D474, Jan. 2019.

[8] C. H. Wu, H. Huang, A. Nikolskaya, Z. Hu, and W. C. Barker, "The iProClass integrated database for protein functional analysis," *Comput. Biol. Chem.*, vol. 28, no. 1, pp. 87–96, Feb. 2004.

[9] J. Huerta-Cepas *et al.*, "EggNOG 5.0: A hierarchical, functionally and phylogenetically annotated orthology resource based on 5090 organisms and 2502 viruses," *Nucleic Acids Res.*, vol. 47, no. D1, pp. D309–D314, 2019.

[10] M. Blum *et al.*, "The InterPro protein families and domains database: 20 years on," *Nucleic Acids Res.*, vol. 49, no. D1, pp. D344–D354, Jan. 2021.

[11] I. Letunic, S. Khedkar, and P. Bork, "SMART: recent updates, new developments and status in 2020," *Nucleic Acids Res.*, vol. 49, no. D1, pp. D458–D460, Jan. 2021.

[12] M. Y. Galperin, Y. I. Wolf, K. S. Makarova, R. Vera Alvarez, D. Landsman, and E. V Koonin, "COG database update: focus on microbial diversity, model organisms, and widespread pathogens," *Nucleic Acids Res.*, vol. 49, no. D1, pp. D274–D281, Jan. 2021.

[13] D. Moreira and C. Brochier-Armanet, "Giant viruses, giant chimeras: The multiple evolutionary histories of *Mimivirus* genes," *BMC Evol. Biol.*, vol. 8, no. 1, p. 12, 2008.

[14] T. B. Tran, "Treatment of multidrug-resistant *Acinetobacter baumannii* with novel combinations of polymyxin B and other non-antibiotic drugs," Monash University, 2018.

[15] S. L. Alderman, D. A. Crossley, R. M. Elsey, and T. E. Gillis, "Hypoxia-induced reprogramming of the cardiac phenotype in American alligators (*Alligator mississippiensis*) revealed by quantitative proteomics," *Sci. Rep.*, vol. 9, no. 1, p. 8592, Dec. 2019.

[16] K. A. O'Hanlon *et al.*, "Targeted Disruption of Nonribosomal Peptide Synthetase *pes3* Augments the Virulence of *Aspergillus fumigatus*," *Infect. Immun.*, vol. 79, no. 10, pp. 3978–3992, Oct. 2011.

[17] F. Wang *et al.*, "FluG affects secretion in colonies of *Aspergillus niger*," *Antonie Van Leeuwenhoek*, vol. 107, no. 1, pp. 225–240, Jan. 2015.

[18] H. Antelmann *et al.*, "The extracellular and cytoplasmic proteomes of the non-virulent *Bacillus anthracis strain UM23C1-2*," *Proteomics*, vol. 5, no. 14, pp. 3684–3695, Sep. 2005.

[19] K. D. Passalacqua *et al.*, "Strand-Specific RNA-Seq Reveals Ordered Patterns of Sense and Antisense Transcription in *Bacillus anthracis*," *PLoS One*, vol. 7, no. 8, p. e43350, Aug. 2012.

[20] M. H. Godsey *et al.*, "The 2.2 Å resolution crystal structure of *Bacillus cereus* Nif3-family protein YqfO reveals a conserved dimetal-binding motif and a regulatory domain," *Protein Sci.*, vol. 16, no. 7, pp. 1285–1293, Jul. 2007.

[21] Ö. Baysal *et al.*, "A Proteomic Approach Provides New Insights into the Control of Soil-Borne Plant Pathogens by *Bacillus* Species," *PLoS One*, vol. 8, no. 1, p. e53182, Jan. 2013.

[22] P. C. Thiaville, D. Iwata-Reuyl, and V. DeCrécy-Lagard, "Diversity of the biosynthesis pathway for threonylcarbamoyladenosine (t^6A), a universal modification of tRNA," *RNA Biol.*, vol. 11, no. 12, pp. 1529–1539, 2014.

[23] M. Ogura, T. Sato, and K. Abe, "*Bacillus subtilis YlxR*, Which Is Involved in Glucose-Responsive Metabolic Changes, Regulates Expression of *tsaD* for Protein Quality Control of Pyruvate Dehydrogenase," *Front. Microbiol.*, vol. 10, no. May, pp. 1–15, May 2019.

[24] M. Ogura and K. Asai, "Glucose Induces ECF Sigma Factor Genes, *sigX* and *sigM*, Independent of Cognate Anti-sigma Factors through

Acetylation of CshA in *Bacillus subtilis*," *Front. Microbiol.*, vol. 7, no. NOV, pp. 1–13, Nov. 2016.

[25] M. Ogura and Y. Kanesaki, "Newly Identified Nucleoid-Associated-Like Protein YlxR Regulates Metabolic Gene Expression in *Bacillus subtilis*," *mSphere*, vol. 3, no. 5, pp. 1–15, Oct. 2018.

[26] P. P. Cardenas, B. Carrasco, H. Sanchez, G. Deikus, D. H. Bechhofer, and J. C. Alonso, "*Bacillus subtilis* polynucleotide phosphorylase 3′-to-5′ DNase activity is involved in DNA repair," *Nucleic Acids Res.*, vol. 37, no. 12, pp. 4157–4169, Jul. 2009.

[27] V. Ramaniuk, "Factors interacting with bacterial RNA polymerase and their effect on the regulation of transcription initiation," Czech Academy of Sciences, 2018.

[28] A. Manzourolajdad and J. Arnold, "Secondary structural entropy in RNA switch (Riboswitch) identification," *BMC Bioinformatics*, vol. 16, no. 1, p. 133, Dec. 2015.

[29] M. Tajkarimi and H. M. Wexler, "CRISPR-Cas Systems in *Bacteroides fragilis*, an Important Pathobiont in the Human Gut Microbiome," *Front. Microbiol.*, vol. 8, no. NOV, pp. 1–21, Nov. 2017.

[30] G. Dionisio, P. Kryger, and T. Steenberg, "Label-Free Differential Proteomics and Quantification of Exoenzymes from Isolates of the Entomopathogenic Fungus *Beauveria bassiana*," *Insects*, vol. 7, no. 4, p. 54, Oct. 2016.

[31] J. Yuan *et al.*, "A Proteome Reference Map and Proteomic Analysis of *Bifidobacterium longum NCC2705*," *Mol. Cell. Proteomics*, vol. 5, no. 6, pp. 1105–1118, Jun. 2006.

[32] J. Chen *et al.*, "All-trans retinoic acid affects subcellular localization of a novel BmNIF3l protein: functional deduce and tissue distribution of *NIF3l* gene from silkworm (Bombyx mori)," *Arch. Insect Biochem. Physiol.*, vol. 74, no. 4, pp. 217–231, Jul. 2010.

[33] M. S. Medrano, P. F. Policastro, T. G. Schwan, and J. Coburn, "Interaction of *Borrelia burgdorferi Hbb* with the p66 promoter," *Nucleic Acids Res.*, vol. 38, no. 2, pp. 414–427, Jan. 2010.

[34] J. A. Livengood, V. L. Schmit, and R. D. Gilmore, "Global Transcriptome Analysis of *Borrelia burgdorferi* during Association with Human Neuroglial Cells," *Infect. Immun.*, vol. 76, no. 1, pp. 298–307, Jan. 2008.

[35] R. Iyer and I. Schwartz, "Microarray-Based Comparative Genomic and Transcriptome Analysis of *Borrelia burgdorferi*," *Microarrays*, vol. 5, no. 2, p. 9, Apr. 2016.

[36] X.-D. Zheng *et al.*, "Whole Transcriptome Analysis Identifies the Taxonomic Status of a New Chinese Native Cattle Breed and Reveals Genes Related to Body Size," *Front. Genet.*, vol. 11, no. November, pp. 1–11, Nov. 2020.

[37] L. Long, Y. Zhu, Z. Li, H. Zhang, L. Liu, and J. Bai, "Differential expression of skeletal muscle mitochondrial proteins in yak, dzo, and cattle: a proteomics-based study," *J. Vet. Med. Sci.*, vol. 82, no. 8, pp. 1178–1186, 2020.

[38] A. Manzano-Marín, "No evidence for *Wolbachia* as a nutritional co-obligate endosymbiont in the aphid *Pentalonia nigronervosa*," *BioRxiv*, 2019.

[39] Z. Jiang *et al.*, "Comparative analysis of genome sequences from four strains of the *Buchnera aphidicola Mp* endosymbion of the green peach aphid, *Myzus persicae*," *BMC Genomics*, vol. 14, no. 1, p. 917, 2013.

[40] K. Held, J. Gasper, S. Morgan, R. Siehnel, P. Singh, and C. Manoil, "Determinants of Extreme β-Lactam Tolerance in the *Burkholderia pseudomallei* Complex," *Antimicrob. Agents Chemother.*, vol. 62, no. 4, Apr. 2018.

[41] S. Wagley *et al.*, "The Twin Arginine Translocation System Is Essential for Aerobic Growth and Full Virulence of *Burkholderia thailandensis*," *J. Bacteriol.*, vol. 196, no. 2, pp. 407–416, Jan. 2014.

[42] P. Malik-Kale, C. T. Parker, and M. E. Konkel, "Culture of *Campylobacter jejuni* with Sodium Deoxycholate Induces Virulence Gene Expression," *J. Bacteriol.*, vol. 190, no. 7, pp. 2286–2297, Apr. 2008.

[43] A. S. Jama, "Cj0700 is a Remote CheZ Orthologue Involved in *Campylobacter jejuni* Chemotaxis Signal Transduction," University of Leicester, 2014.

[44] K. A. Ryan, N. Karim, M. Worku, S. A. Moore, C. W. Penn, and P. W. O'Toole, "HP0958 is an essential motility gene in *Helicobacter pylori*," *FEMS Microbiol. Lett.*, vol. 248, no. 1, pp. 47–55, Jul. 2005.

[45] I. Porcelli, M. Reuter, B. M. Pearson, T. Wilhelm, and A. H. M. van Vliet, "Parallel evolution of genome structure and transcriptional landscape in the Epsilonproteobacteria," *BMC Genomics*, vol. 14, no. 1, p. 616, 2013.

[46] D. Esser, G. Alvarez-Llamas, M. P. De Vries, D. Weening, R. J. Vonk, and H. Roelofsen, "Sample Stability and Protein Composition of Saliva: Implications for Its Use as a Diagnostic Fluid," *Biomark. Insights*, vol. 3, p. BMI.S607, Jan. 2008.

[47] R. Samudrala, F. Heffron, and J. E. McDermott, "Accurate Prediction of Secreted Substrates and Identification of a Conserved Putative Secretion Signal for Type III Secretion Systems," *PLoS Pathog.*, vol. 5, no. 4, p. e1000375, Apr. 2009.

[48] W. Huston, "*Chlamydia* diagnostics and issues of treatment failure," in *Australian Sexual Health Conference*, 2017.

[49] W. Liang, Y. Bi, H. Wang, S. Dong, K. Li, and J. Li, "Gene Expression Profiling of *Clostridium botulinum* under Heat Shock Stress," *Biomed Res. Int.*, vol. 2013, pp. 1–8, 2013.

[50] K. Selby, G. Mascher, P. Somervuo, M. Lindström, and H. Korkeala, "Heat shock and prolonged heat stress attenuate neurotoxin and sporulation gene expression in group I *Clostridium botulinum strain ATCC 3502*," *PLoS One*, vol. 12, no. 5, p. e0176944, May 2017.

[51] N. Daou *et al.*, "Impact of CodY protein on metabolism, sporulation and virulence in *Clostridioides difficile ribotype 027*," *PLoS One*, vol. 14, no. 1, p. e0206896, Jan. 2019.

[52] R. W. McKee, C. K. Harvest, and R. Tamayo, "Cyclic Diguanylate Regulates Virulence Factor Genes via Multiple Riboswitches in *Clostridium difficile*," *mSphere*, vol. 3, no. 5, pp. 1–15, Oct. 2018.

[53] R. A. Stabler, E. Valiente, L. F. Dawson, M. He, J. Parkhill, and B. W. Wren, "In-depth genetic analysis of *Clostridium difficile PCR-ribotype 027* strains reveals high genome fluidity including point mutations and inversions," *Gut Microbes*, vol. 1, no. 4, pp. 269–276, Jul. 2010.

[54] T. M. Charlton, A. Kovacs-Simon, S. L. Michell, N. F. Fairweather, and E. W. Tate, "Quantitative Lipoproteomics in *Clostridium difficile* Reveals a Role for Lipoproteins in Sporulation," *Chem. Biol.*, vol. 22, no. 11, pp. 1562–1573, Nov. 2015.

[55] B. P. Girinathan, J. Ou, B. Dupuy, and R. Govind, "Pleiotropic roles of *Clostridium difficile sin* locus," *PLOS Pathog.*, vol. 14, no. 3, p. e1006940, Mar. 2018.

[56] M. Wittchen *et al.*, "Transcriptome sequencing of the human pathogen *Corynebacterium diphtheriae NCTC 13129* provides detailed insights into its transcriptional landscape and into DtxR-mediated transcriptional regulation," *BMC Genomics*, vol. 19, no. 1, p. 82, Dec. 2018.

[57] C. L. Araújo *et al.*, "In silico functional prediction of hypothetical proteins from the core genome of *Corynebacterium pseudotuberculosis* biovar *ovis*," *PeerJ*, vol. 8, p. e9643, Aug. 2020.

[58] H. Falkenberg *et al.*, "Mass spectrometric evaluation of upstream and downstream process influences on host cell protein patterns in biopharmaceutical products," *Biotechnol. Prog.*, vol. 35, no. 3, p. e2788, May 2019.

[59] Q. Luo, J. L. Groh, J. D. Ballard, and L. R. Krumholz, "Identification of Genes That Confer Sediment Fitness to Desulfovibrio desulfuricans G20," *Appl. Environ. Microbiol.*, vol. 73, no. 19, pp. 6305–6312, Oct. 2007.

[60] Q. Luo, "Genes Required for Sediment Fitness in *Desulfovibrio desulfuricans G20*," University of Oklahoma, 2005.

[61] M. Ashburner *et al.*, "An exploration of the sequence of a 2.9-Mb region of the genome of *Drosophila melanogaster*: the Adh region.," *Genetics*, vol. 153, no. 1, pp. 179–219, Sep. 1999.

[62] R. Geisler, A. Bergmann, Y. Hiromi, and C. Nüsslein-Volhard, "cactus, a gene involved in dorsoventral pattern formation of *Drosophila*, is related to the IκB gene family of vertebrates," *Cell*, vol. 71, no. 4, pp. 613–621, Nov. 1992.

[63] A. A. Fontes, "Mitochondrial and cell cycle functions of SLIMP," Universitat de Barcelona, 2018.

[64] M. Y. Galperin and E. V. Koonin, "From complete genome sequence to 'complete' understanding?," *Trends Biotechnol.*, vol. 28, no. 8, pp. 398–406, Aug. 2010.

[65] J. E. Ladner *et al.*, "Crystal structure of *Escherichia coli* protein YbgI, a toroidal structure with a dinuclear metal site.," *BMC Struct. Biol.*, vol. 3, p. 7, Sep. 2003.

[66] T. D. Niehaus, M. Elbadawi-Sidhu, V. De Crécy-Lagard, O. Fiehn, and A. D. Hanson, "Discovery of a widespread prokaryotic 5-oxoprolinase that was hiding in plain sight," *J. Biol. Chem.*, vol. 292, no. 39, pp. 16360–16367, 2017.

[67] J. P. Rooney *et al.*, "Systems based mapping demonstrates that recovery from alkylation damage requires DNA repair, RNA processing, and translation associated networks," *Genomics*, vol. 93, no. 1, pp. 42–51, Jan. 2009.

[68] J. J. Díaz-Mejía, M. Babu, and A. Emili, "Computational and experimental approaches to chart the *Escherichia coli* cell-envelope-associated proteome and interactome," *FEMS Microbiol. Rev.*, vol. 33, no. 1, pp. 66–97, Jan. 2009.

[69] R. T. Byrne, S. H. Chen, E. A. Wood, E. L. Cabot, and M. M. Cox, "*Escherichia coli* Genes and Pathways Involved in Surviving Extreme Exposure to Ionizing Radiation," *J. Bacteriol.*, vol. 196, no. 20, pp. 3534–3545, Oct. 2014.

[70] O. V. Sergeeva, D. O. Bredikhin, M. V. Nesterchuk, M. V. Serebryakova, P. V. Sergiev, and O. A. Dontsova, "Possible Role of *Escherichia coli* Protein YbgI," *Biochem.*, vol. 83, no. 3, pp. 270–280, Mar. 2018.

[71] N. Škunca *et al.*, "Phyletic Profiling with Cliques of Orthologs Is Enhanced by Signatures of Paralogy Relationships," *PLoS Comput. Biol.*, vol. 9, no. 1, p. e1002852, Jan. 2013.

[72] D. Ghersi and R. Sanchez, "Beyond structural genomics: computational approaches for the identification of ligand binding sites in protein

structures," *J. Struct. Funct. Genomics*, vol. 12, no. 2, pp. 109–117, Jul. 2011.

[73] D. K. Rollins, D. Zhai, A. L. Joe, J. W. Guidarelli, A. Murarka, and R. Gonzalez, "A novel data mining method to identify assay-specific signatures in functional genomic studies," *BMC Bioinformatics*, vol. 7, no. 1, p. 377, Dec. 2006.

[74] P. J. Pomposiello, M. H. J. Bennik, and B. Demple, "Genome-Wide Transcriptional Profiling of the *Escherichia coli* Responses to Superoxide Stress and Sodium Salicylate," *J. Bacteriol.*, vol. 183, no. 13, pp. 3890–3902, Jul. 2001.

[75] C. A. Pattrick, J. P. Webb, J. Green, R. R. Chaudhuri, M. O. Collins, and D. J. Kelly, "Proteomic Profiling, Transcription Factor Modeling, and Genomics of Evolved Tolerant Strains Elucidate Mechanisms of Vanillin Toxicity in *Escherichia coli*," *mSystems*, vol. 4, no. 4, pp. 1–29, Aug. 2019.

[76] M.-J. Han and S. Y. Lee, "The *Escherichia coli* Proteome: Past, Present, and Future Prospects," *Microbiol. Mol. Biol. Rev.*, vol. 70, no. 2, pp. 362–439, Jun. 2006.

[77] R. G. Martin, W. K. Gillette, N. I. Martin, and J. L. Rosner, "Complex formation between activator and RNA polymerase as the basis for transcriptional activation by MarA and SoxS in *Escherichia coli*," *Mol. Microbiol.*, vol. 43, no. 2, pp. 355–370, Mar. 2002.

[78] Y.-H. Ho, P. Shah, Y.-W. Chen, and C.-S. Chen, "Systematic Analysis of Intracellular-targeting Antimicrobial Peptides, Bactenecin 7, Hybrid of Pleurocidin and Dermaseptin, Proline–Arginine-rich Peptide, and Lactoferricin B, by Using *Escherichia coli* Proteome Microarrays," *Mol. Cell. Proteomics*, vol. 15, no. 6, pp. 1837–1847, Jun. 2016.

[79] A. Suzuki, T. Ando, I. Yamato, and S. Miyazaki, "FCANAL: Structure based protein function prediction method. Application to enzymes and binding proteins," *Chem-Bio Informatics J.*, vol. 5, no. 3, pp. 39–55, 2006.

[80] G. Su *et al.*, "Toxicogenomic Mechanisms of 6-HO-BDE-47, 6-MeO-BDE-47, and BDE-47 in E. coli," *Environ. Sci. Technol.*, vol. 46, no. 2, pp. 1185–1191, Jan. 2012.

[81] D. Chung *et al.*, "dPeak: High Resolution Identification of Transcription Factor Binding Sites from PET and SET ChIP-Seq Data," *PLoS Comput. Biol.*, vol. 9, no. 10, p. e1003246, Oct. 2013.

[82] Y. Yao, L. Fan, Y. Shi, I. Odsbu, and Morigen, "A Spatial Control for Correct Timing of Gene Expression during the *Escherichia coli* Cell Cycle," *Genes (Basel).*, vol. 8, no. 1, p. 1, Dec. 2016.

[83] Y. Wei, J.-M. Lee, D. R. Smulski, and R. A. LaRossa, "Global Impact of *sdiA* Amplification Revealed by Comprehensive Gene Expression Profiling of *Escherichia coli*," *J. Bacteriol.*, vol. 183, no. 7, pp. 2265–2272, Apr. 2001.

[84] C. Park, S. Zhou, J. Gilmore, and S. Marqusee, "Energetics-based Protein Profiling on a Proteomic Scale: Identification of Proteins Resistant to Proteolysis," *J. Mol. Biol.*, vol. 368, no. 5, pp. 1426–1437, May 2007.

[85] C. Dartigalongue, "EcfE, a new essential inner membrane protease: its role in the regulation of heat shock response in *Escherichia coli*," *EMBO J.*, vol. 20, no. 21, pp. 5908–5918, Nov. 2001.

[86] T. Shimada, N. Fujita, K. Yamamoto, and A. Ishihama, "Novel Roles of cAMP Receptor Protein (CRP) in Regulation of Transport and Metabolism of Carbon Sources," *PLoS One*, vol. 6, no. 6, p. e20081, Jun. 2011.

[87] N. Thorenoor *et al.*, "Localization of the Death Effector Domain of Fas-Associated Death Domain Protein into the Membrane of *Escherichia coli* Induces Reactive Oxygen Species-Involved Cell Death," *Biochemistry*, vol. 49, no. 7, pp. 1435–1447, Feb. 2010.

[88] L. Gao, X. Ma, J. Hu, X. Zhang, and T. Chai, "Proteomic analysis of ESBL-producing *Escherichia coli* under bentonite condition," *Environ. Sci. Pollut. Res.*, vol. 26, no. 22, pp. 22305–22311, Aug. 2019.

[89] C. M. Gifford, "The genes encoding endonuclease VIII and endonuclease III in *Escherichia coli* are transcribed as the terminal genes in operons," *Nucleic Acids Res.*, vol. 28, no. 3, pp. 762–769, Feb. 2000.

[90] J. P. Rooney *et al.*, "Cross-species Functionome analysis identifies proteins associated with DNA repair, translation and aerobic respiration as conserved modulators of UV-toxicity," *Genomics*, vol. 97, no. 3, pp. 133–147, Mar. 2011.

[91] K. S. Stenger, "Identification of the response pathways of *Escherichia coli* and *Enterococcus faecalis* to glyphosate and its major breakdown product aminomethyl phosphonic acid (AMPA)," North-West University, 2019.

[92] K. A. Selim *et al.*, "Functional and structural characterization of PII-like protein CutA does not support involvement in heavy metal tolerance and hints at a small-molecule carrying/signaling role," *FEBS J.*, vol. 288, no. 4, pp. 1142–1162, Feb. 2021.

[93] W. L. O. da Costa *et al.*, "Functional annotation of hypothetical proteins from the *Exiguobacterium antarcticum strain B7* reveals proteins involved in adaptation to extreme environments, including high arsenic resistance," *PLoS One*, vol. 13, no. 6, p. e0198965, Jun. 2018.

[94] I. Comas, F. González-Candelas, and M. Zúñiga, "Unraveling the evolutionary history of the phosphoryl-transfer chain of the phosphoenolpyruvate:phosphotransferase system through phylogenetic analyses and genome context," *BMC Evol. Biol.*, vol. 8, no. 1, p. 147, 2008.

[95] N. Alqazlan, "Immune responses in the gastrointestinal tract of chickens infected with low pathogenic *influenza virus subtype H9N2* and the role of probiotic *Lactobacilli* in antiviral immunity and vaccine immunogenicity," The University of Guelph, 2020.

[96] S. Shulami *et al.*, "Multiple regulatory mechanisms control the expression of the *Geobacillus stearothermophilus* gene for extracellular xylanase," *J. Biol. Chem.*, vol. 289, no. 37, pp. 25957–25975, 2014.

[97] M. J. A. Daas *et al.*, "Engineering *Geobacillus thermodenitrificans* to introduce cellulolytic activity; expression of native and heterologous cellulase genes," *BMC Biotechnol.*, vol. 18, no. 1, p. 42, Dec. 2018.

[98] M. J. A. Daas, A. H. P. van de Weijer, W. M. de Vos, J. van der Oost, and R. van Kranenburg, "Isolation of a genetically accessible thermophilic xylan degrading bacterium from compost," *Biotechnol. Biofuels*, vol. 9, no. 1, p. 210, Dec. 2016.

[99] S. Shulami *et al.*, "A Two-Component System Regulates the Expression of an ABC Transporter for Xylo-Oligosaccharides in *Geobacillus stearothermophilus*," *Appl. Environ. Microbiol.*, vol. 73, no. 3, pp. 874–884, Feb. 2007.

[100] S. Shulami, O. Gat, A. L. Sonenshein, and Y. Shoham, "The Glucuronic Acid Utilization Gene Cluster from *Bacillus stearothermophilus T-6*," *J. Bacteriol.*, vol. 181, no. 12, pp. 3695–3704, Jun. 1999.

[101] O. Alalouf, Y. Balazs, M. Volkinshtein, Y. Grimpel, G. Shoham, and Y. Shoham, "A New Family of Carbohydrate Esterases Is Represented by a GDSL Hydrolase/Acetylxylan Esterase from *Geobacillus stearothermophilus*," *J. Biol. Chem.*, vol. 286, no. 49, pp. 41993–42001, Dec. 2011.

[102] C. Leang *et al.*, "Adaptation to Disruption of the Electron Transfer Pathway for Fe(III) Reduction in *Geobacter sulfurreducens*," *J. Bacteriol.*, vol. 187, no. 17, pp. 5918–5926, Sep. 2005.

[103] D. Gangaiah *et al.*, "Activation of CpxRA in *Haemophilus ducreyi* Primarily Inhibits the Expression of Its Targets, Including Major Virulence Determinants," *J. Bacteriol.*, vol. 195, no. 15, pp. 3486–3502, Aug. 2013.

[104] M. Labandeira-Rey, J. R. Mock, and E. J. Hansen, "Regulation of Expression of the *Haemophilus ducreyi* LspB and LspA2 Proteins by CpxR," *Infect. Immun.*, vol. 77, no. 8, pp. 3402–3411, Aug. 2009.

[105] S. M. Spinola *et al.*, "Activation of the CpxRA System by Deletion of *cpxA* Impairs the Ability of *Haemophilus ducreyi* To Infect Humans," *Infect. Immun.*, vol. 78, no. 9, pp. 3898–3904, Sep. 2010.

[106] D. Gangaiah *et al.*, "*Haemophilus ducreyi* Hfq Contributes to Virulence Gene Regulation as Cells Enter Stationary Phase," *MBio*, vol. 5, no. 1, pp. 1–13, Feb. 2014.

[107] E. Kolker, "Identification and functional analysis of 'hypothetical' genes expressed in *Haemophilus influenzae*," *Nucleic Acids Res.*, vol. 32, no. 8, pp. 2353–2361, Apr. 2004.

[108] Y. Furuta, K. Abe, and I. Kobayashi, "Genome comparison and context analysis reveals putative mobile forms of restriction–modification systems and related rearrangements," *Nucleic Acids Res.*, vol. 38, no. 7, pp. 2428–2443, Apr. 2010.

[109] M. Shahbaaz, Md. ImtaiyazHassan, and F. Ahmad, "Functional Annotation of Conserved Hypothetical Proteins from *Haemophilus influenzae Rd KW20*," *PLoS One*, vol. 8, no. 12, p. e84263, Dec. 2013.

[110] S. W. Dickey, "Rhomboid Proteolysis is a Rate-Governed Reaction, Yet is Dispensable for *E. coli* Colonization of the Mouse Colon," The Johns Hopkins University, 2013.

[111] L. Prunetti *et al.*, "Deciphering the Translation Initiation Factor 5A Modification Pathway in Halophilic Archaea," *Archaea*, vol. 2016, pp. 1–14, 2016.

[112] J. H. Martin *et al.*, "GlpR Is a Direct Transcriptional Repressor of Fructose Metabolic Genes in *Haloferax volcanii*," *J. Bacteriol.*, vol. 200, no. 17, pp. 1–18, Sep. 2018.

[113] H.-P. Choi *et al.*, "Biochemical Characterization of Hypothetical Proteins from *Helicobacter pylori*," *PLoS One*, vol. 8, no. 6, p. e66605, Jun. 2013.

[114] Y.-C. Chang *et al.*, "COMBREX-DB: an experiment centered database of protein function: knowledge, predictions and knowledge gaps," *Nucleic Acids Res.*, vol. 44, no. D1, pp. D330–D335, Jan. 2016.

[115] L. E. Pereira, J. Tsang, J. Mrázek, and T. R. Hoover, "The zinc-ribbon domain of *Helicobacter pylori HP0958*: requirement for RpoN accumulation and possible roles of homologs in other bacteria," *Microb. Inform. Exp.*, vol. 1, no. 1, p. 8, 2011.

[116] S. Tascou, J. Uedelhoven, C. Dixkens, K. Nayernia, W. Engel, and P. Burfeind, "Isolation and characterization of a novel human gene, *NIF3L1*, and its mouse ortholog, *Nif3l1*, highly conserved from bacteria to mammals," *Cytogenet. Genome Res.*, vol. 90, no. 3–4, pp. 330–336, 2000.

[117] S. Tascou, T. W. Kang, R. Trappe, W. Engel, and P. Burfeind, "Identification and characterization of NIF3L1 BP1, a novel cytoplasmic interaction partner of the NIF3L1 protein," *Biochem. Biophys. Res. Commun.*, vol. 309, no. 2, pp. 440–448, 2003.

[118] B. Xia, Y. Li, J. Zhou, B. Tian, and L. Feng, "Identification of potential pathogenic genes associated with osteoporosis," *Bone Joint Res.*, vol. 6, no. 12, pp. 640–648, Dec. 2017.

[119] N. J. Camp *et al.*, "Discordant Haplotype Sequencing Identifies Functional Variants at the 2q33 Breast Cancer Risk Locus," *Cancer Res.*, vol. 76, no. 7, pp. 1916–1925, Apr. 2016.

[120] M. A. Nalls *et al.*, "Extended tracts of homozygosity identify novel candidate genes associated with late-onset Alzheimer's disease," *Neurogenetics*, vol. 10, no. 3, pp. 183–190, Jul. 2009.

[121] S. S. S. J. Ahmed, A. R. Ahameethunisa, W. Santosh, S. Chakravarthy, and S. Kumar, "Systems biological approach on neurological disorders: a novel molecular connectivity to aging and psychiatric diseases," *BMC Syst. Biol.*, vol. 5, no. 1, p. 6, 2011.

[122] H. Akiyama, N. Fujisawa, Y. Tashiro, N. Takanabe, A. Sugiyama, and F. Tashiro, "The Role of Transcriptional Corepressor Nif3l1 in Early Stage of Neural Differentiation via Cooperation with Trip15/CSN2," *J. Biol. Chem.*, vol. 278, no. 12, pp. 10752–10762, Mar. 2003.

[123] G. Antoniali *et al.*, "Mammalian APE1 controls miRNA processing and its interactome is linked to cancer RNA metabolism," *Nat. Commun.*, vol. 8, no. 1, p. 797, Dec. 2017.

[124] P. Bhatraju *et al.*, "Associations between single nucleotide polymorphisms in the FAS pathway and acute kidney injury," *Crit. Care*, vol. 19, no. 1, p. 368, Dec. 2015.

[125] L. Chauhan *et al.*, "Genome-wide association analysis identified splicing single nucleotide polymorphism in CFLAR predictive of triptolide chemo-sensitivity," *BMC Genomics*, vol. 16, no. 1, p. 483, Dec. 2015.

[126] C. M. Duzyj, M. J. Paidas, L. Jebailey, J. Huang, and E. R. Barnea, "PreImplantation factor (PIF*) promotes embryotrophic and neuroprotective decidual genes: effect negated by epidermal growth factor," *J. Neurodev. Disord.*, vol. 6, no. 1, p. 36, 2014.

[127] O. El Bounkari *et al.*, "Nuclear localization of the pre-mRNA associating protein THOC7 depends upon its direct interaction with Fms tyrosine kinase interacting protein (FMIP)," *FEBS Lett.*, vol. 583, no. 1, pp. 13–18, Jan. 2009.

[128] B. J. Fenner *et al.*, "A cellular and proteomic approach to assess proteins extracted from cryopreserved human amnion in the cultivation of corneal stromal keratocytes for stromal cell therapy," *Eye Vis.*, vol. 6, no. 1, p. 30, Dec. 2019.

[129] J. Gawinecka *et al.*, "Sporadic Creutzfeldt-Jakob disease subtype-specific alterations of the brain proteome: Impact on Rab3a recycling," *Proteomics*, vol. 12, no. 23–24, pp. 3610–3620, Dec. 2012.

[130] P. E. Geyer *et al.*, "Plasma Proteome Profiling to detect and avoid sample-related biases in biomarker studies," *EMBO Mol. Med.*, vol. 11, no. 11, pp. 1–12, Nov. 2019.

[131] C. R. Giannangelo, "Biochemical pathways and molecular targets involved in the mechanism of action of ozonide antimalarials in *Plasmodium falciparum*," Monash University, 2018.

[132] B. Giotti *et al.*, "Assembly of a parts list of the human mitotic cell cycle machinery," *J. Mol. Cell Biol.*, vol. 11, no. 8, pp. 703–718, Aug. 2019.

[133] S. Gräf *et al.*, "Identification of rare sequence variation underlying heritable pulmonary arterial hypertension," *Nat. Commun.*, vol. 9, no. 1, p. 1416, Dec. 2018.

[134] S. Hadano *et al.*, "Cloning and characterization of three novel genes, ALS2CR1, ALS2CR2, and ALS2CR3, in the juvenile amyotrophic lateral sclerosis (ALS2) critical region at chromosome 2q33-q34: Candidate genes for ALS2," *Genomics*, vol. 71, no. 2, pp. 200–213, 2001.

[135] N. S. Hadj-Hamou, "Functional Signatures of Radio-induction in Sarcomas Developing in the Radiation Field After Radiotherapy," University of Paris, 2010.

[136] T.-S. He *et al.*, "THO Complex Subunit 7 Homolog Negatively Regulates Cellular Antiviral Response against RNA Viruses by Targeting TBK1," *Viruses*, vol. 11, no. 2, p. 158, Feb. 2019.

[137] M. Johnson *et al.*, "Genomic and transcriptomic comparison of allergen and silver nanoparticle-induced mast cell degranulation reveals novel non-immunoglobulin E mediated mechanisms," *PLoS One*, vol. 13, no. 3, p. e0193499, Mar. 2018.

[138] M. Kai *et al.*, "TET1 Depletion Induces Aberrant CpG Methylation in Colorectal Cancer Cells," *PLoS One*, vol. 11, no. 12, p. e0168281, Dec. 2016.

[139] K. R. Kalari *et al.*, "An Integrated Model of the Transcriptome of HER2-Positive Breast Cancer," *PLoS One*, vol. 8, no. 11, p. e79298, Nov. 2013.

[140] H. Kölbel, D. Hathazi, M. Jennings, R. Horvath, A. Roos, and U. Schara, "Identification of Candidate Protein Markers in Skeletal Muscle of Laminin-211-Deficient CMD Type 1A-Patients," *Front. Neurol.*, vol. 10, no. MAY, May 2019.

[141] K. Kusonmano *et al.*, "Identification of highly connected and differentially expressed gene subnetworks in metastasizing endometrial cancer," *PLoS One*, vol. 13, no. 11, p. e0206665, Nov. 2018.

[142] Y. Li, B. Xie, Z. Jiang, and B. Yuan, "Relationship between osteoporosis and osteoarthritis based on DNA methylation.," *Int. J. Clin. Exp. Pathol.*, vol. 12, no. 9, pp. 3399–3407, 2019.

[143] C.-Y. Lin *et al.*, "Discovery of estrogen receptor alpha target genes and response elements in breast tumor cells.," *Genome Biol.*, vol. 5, no. 9, p.

R66, 2004.

[144] L. Liu *et al.*, “DAWN: a framework to identify autism genes and subnetworks using gene expression and genetics,” *Mol. Autism*, vol. 5, no. 1, p. 22, 2014.

[145] L. Liu *et al.*, “Insight into novel RNA-binding activities via large-scale analysis of lncRNA-bound proteome and IDH1-bound transcriptome,” *Nucleic Acids Res.*, vol. 47, no. 5, pp. 2244–2262, Mar. 2019.

[146] T.-P. Lu, Y.-Y. Hsu, L.-C. Lai, M.-H. Tsai, and E. Y. Chuang, “Identification of Gene Expression Biomarkers for Predicting Radiation Exposure,” *Sci. Rep.*, vol. 4, no. 1, p. 6293, May 2015.

[147] G. Merla, C. Howald, S. E. Antonarakis, and A. Reymond, “The subcellular localization of the ChoRE-binding protein, encoded by the Williams–Beuren syndrome critical region gene 14, is regulated by 14-3-3,” *Hum. Mol. Genet.*, vol. 13, no. 14, pp. 1505–1514, Jul. 2004.

[148] A. E. Oja *et al.*, “Trigger-happy resident memory CD4+ T cells inhabit the human lungs,” *Mucosal Immunol.*, vol. 11, no. 3, pp. 654–667, May 2018.

[149] D. Patel, D. Ythier, F. Brozzi, D. L. Eizirik, and B. Thorens, “Clic4, a novel protein that sensitizes β-cells to apoptosis,” *Mol. Metab.*, vol. 4, no. 4, pp. 253–264, Apr. 2015.

[150] L. Qiu and X. Liu, “Identification of key genes involved in myocardial infarction,” *Eur. J. Med. Res.*, vol. 24, no. 1, p. 22, Dec. 2019.

[151] D. A. Quigley *et al.*, “The 5p12 breast cancer susceptibility locus affects MRPS30 expression in estrogen-receptor positive tumors,” *Mol. Oncol.*, vol. 8, no. 2, pp. 273–284, Mar. 2014.

[152] J. Rijlaarsdam *et al.*, “An epigenome-wide association meta-analysis of prenatal maternal stress in neonates: A model approach for replication,” *Epigenetics*, vol. 11, no. 2, pp. 140–149, Feb. 2016.

[153] C. Rylander, V. Dumeaux, K. S. Olsen, M. Waaseth, T. M. Sandanger, and E. Lund, “Using blood gene signatures for assessing effects of exposure to perfluoroalkyl acids (PFAAs) in humans: the NOWAC postgenome study.,” *Int. J. Mol. Epidemiol. Genet.*, vol. 2, no. 3, pp. 207–16, Aug. 2011.

[154] M. E. Sáez *et al.*, “Genome Wide Meta-Analysis identifies common genetic signatures shared by heart function and Alzheimer’s disease,” *Sci. Rep.*, vol. 9, no. 1, p. 16665, Dec. 2019.

[155] M. R. Sailani *et al.*, “DNA-Methylation Patterns in Trisomy 21 Using Cells from Monozygotic Twins,” *PLoS One*, vol. 10, no. 8, p. e0135555, Aug. 2015.

[156] A. Schrader *et al.*, “Identification of a new gene regulatory circuit involving B cell receptor activated signaling using a combined analysis of experimental, clinical and global gene expression data,” *Oncotarget*, vol. 7, no. 30, pp. 47061–47081, Jul. 2016.

[157] T. K. Sigdel *et al.*, “Profiling of Autoantibodies in IgA Nephropathy, an Integrative Antibiomics Approach,” *Clin. J. Am. Soc. Nephrol.*, vol. 6, no. 12, pp. 2775–2784, Dec. 2011.

[158] N. Simonis *et al.*, “Host-pathogen interactome mapping for HTLV-1 and -2 retroviruses,” *Retrovirology*, vol. 9, no. 1, p. 26, 2012.

[159] N. H. Simpson *et al.*, “Genome-wide analysis identifies a role for common copy number variants in specific language impairment,” *Eur. J. Hum. Genet.*, vol. 23, no. 10, pp. 1370–1377, Oct. 2015.

[160] H. Skottman *et al.*, “Gene Expression Signatures of Seven Individual Human Embryonic Stem Cell Lines,” *Stem Cells*, vol. 23, no. 9, pp. 1343–1356, Oct. 2005.

[161] F. G. Thankam, C. S. Boosani, M. F. Dilisio, and D. K. Agrawal, “MicroRNAs associated with inflammation in shoulder tendinopathy and glenohumeral arthritis,” *Mol. Cell. Biochem.*, vol. 437, no. 1–2, pp. 81–97, Jan. 2018.

[162] Y. Tian *et al.*, “Alteration in basal and depolarization induced transcriptional network in iPSC derived neurons from Timothy syndrome,” *Genome Med.*, vol. 6, no. 10, p. 75, Oct. 2014.

[163] O. Tšuiko *et al.*, “Copy number variation analysis detects novel candidate genes involved in follicular growth and oocyte maturation in a cohort of premature ovarian failure cases,” *Hum. Reprod.*, vol. 31, no. 8, pp. 1913–1925, Aug. 2016.

[164] S. Uxa *et al.*, “DREAM and RB cooperate to induce gene repression and cell-cycle arrest in response to p53 activation,” *Nucleic Acids Res.*, vol. 47, no. 17, pp. 9087–9103, Sep. 2019.

[165] A. F. van Ouwerkerk *et al.*, “Identification of atrial fibrillation associated genes and functional non-coding variants,” *Nat. Commun.*, vol. 10, no. 1, p. 4755, Dec. 2019.

[166] L.-J. Wang *et al.*, “Interactome-wide Analysis Identifies End-binding Protein 1 as a Crucial Component for the Speck-like Particle Formation of Activated Absence in Melanoma 2 (AIM2) Inflammasomes,” *Mol. Cell. Proteomics*, vol. 11, no. 11, pp. 1230–1244, Nov. 2012.

[167] L.-S. Wang *et al.*, "Population-based study of genetic variation in individuals with autism spectrum disorders from Croatia," *BMC Med. Genet.*, vol. 11, no. 1, p. 134, Dec. 2010.

[168] M. Wang, L. Li, J. Liu, and J. Wang, "A gene interaction network-based method to measure the common and heterogeneous mechanisms of gynecological cancer," *Mol. Med. Rep.*, vol. 18, no. 1, pp. 230–242, May 2018.

[169] D. Wei *et al.*, "The Molecular Chaperone GRP78 Contributes to Toll-like Receptor 3-mediated Innate Immune Response to Hepatitis C Virus in Hepatocytes," *J. Biol. Chem.*, vol. 291, no. 23, pp. 12294–12309, Jun. 2016.

[170] A. O. Weinzierl *et al.*, "Features of TAP-independent MHC class I ligands revealed by quantitative mass spectrometry," *Eur. J. Immunol.*, vol. 38, no. 6, pp. 1503–1510, Apr. 2008.

[171] D. A. Winer *et al.*, "B cells promote insulin resistance through modulation of T cells and production of pathogenic IgG antibodies," *Nat. Med.*, vol. 17, no. 5, pp. 610–617, May 2011.

[172] Y. Xi *et al.*, "Global comparative gene expression analysis of melanoma patient samples, derived cell lines and corresponding tumor xenografts.," *Cancer Genomics Proteomics*, vol. 5, no. 1, pp. 1–35, 2011.

[173] Y. Xiang, C.-Q. Zhang, and K. Huang, "Predicting glioblastoma prognosis networks using weighted gene co-expression network analysis on TCGA data," *BMC Bioinformatics*, vol. 13, no. S2, p. S12, Dec. 2012.

[174] G. Yu *et al.*, "A discrete organoplatinum(II) metallacage as a multimodality theranostic platform for cancer photochemotherapy," *Nat. Commun.*, vol. 9, no. 1, p. 4335, Dec. 2018.

[175] N. Yu, S. Shin, and K.-A. Lee, "First Korean Case of SATB2 -Associated 2q32-q33 Microdeletion Syndrome," *Ann. Lab. Med.*, vol. 35, no. 2, p. 275, 2015.

[176] J. K. Lamba, T. Feldberg, T. M. Ghosh, N. Bhise, and B. Fridley, "Abstract 2214: Genome-wide association analysis identified genetic markers associated with triptolide cellular sensitivity using HapMap LCLs as model system.," in *Experimental and Molecular Therapeutics*, 2013, vol. 73, no. 8 Supplement, pp. 2214–2214.

[177] A. Malik, K. Pande, A. Kumar, A. Vemula, and M. R. V. Chandramohan, "Finding Pathogenic nsSNP's and their structural effect on COPS2 using Molecular Dynamic Approach," *bioRxiv*, 2020.

[178] S. S. Cury *et al.*, "Increased DSG2 plasmatic levels identified by transcriptomic-based secretome analysis is a potential prognostic biomarker in laryngeal carcinoma," *Oral Oncol.*, vol. 103, no. January, p. 104592, Apr. 2020.

[179] A. Schneeweiss *et al.*, "Update Breast Cancer 2020 Part 1 – Early Breast Cancer: Consolidation of Knowledge About Known Therapies," *Geburtshilfe Frauenheilkd.*, vol. 80, no. 03, pp. 277–287, Mar. 2020.

[180] L. Lv, D. Zhang, P. Hua, and S. Yang, "The glial-specific hypermethylated 3′ untranslated region of histone deacetylase 1 may modulates several signal pathways in Alzheimer's disease," *Life Sci.*, vol. 265, no. November 2020, p. 118760, Jan. 2021.

[181] H. Lin, "Identification of Potential coregenes in Sevoflurance induced Myocardial Energy Metabolismin Patients Undergoing Off-pump Coronary Artery Bypass Graft Surgery using Bioinformatics analysis," *Res. Sq.*, pp. 1–16, 2019.

[182] S. Qu, Q. Shi, J. Xu, W. Yi, and H. Fan, "Weighted Gene Coexpression Network Analysis Reveals the Dynamic Transcriptome Regulation and Prognostic Biomarkers of Hepatocellular Carcinoma," *Evol. Bioinforma.*, vol. 16, p. 117693432092056, Jan. 2020.

[183] J. Wu, S. Liu, Y. Xiang, X. Qu, Y. Xie, and X. Zhang, "Bioinformatic Analysis of Circular RNA-Associated ceRNA Network Associated with Hepatocellular Carcinoma," *Biomed Res. Int.*, vol. 2019, pp. 1–14, Nov. 2019.

[184] K. Fukushima, K. Tsujino, S. Futami, and H. Kida, "Natural Autoantibodies in Chronic Pulmonary Diseases," *Int. J. Mol. Sci.*, vol. 21, no. 3, p. 1138, Feb. 2020.

[185] M. Codrich *et al.*, "Inhibition of APE1-endonuclease activity affects cell metabolism in colon cancer cells via a p53-dependent pathway," *DNA Repair (Amst).*, vol. 82, no. 3, p. 102675, Oct. 2019.

[186] W. Yan *et al.*, "Cell Atlas of the Human Fovea and Peripheral Retina," *bioRxiv*, 2020.

[187] A. R. Chapman *et al.*, "Correlated gene modules uncovered by single-cell transcriptomics with high detectability and accuracy," *bioRxiv*, pp. 14–17, 2020.

[188] M. Dapas, "Investigation of the Genetic Architecture of Polycystic Ovary Syndrome," Northwestern University, 2019.

[189] J. C. Wang, G. Ramaswami, and D. H. Geschwind, "Gene co-expression network analysis in human spinal cord highlights mechanisms underlying amyotrophic lateral sclerosis susceptibility," *bioRxiv*, 2020.

[190] S. V. Jostes, "The bromodomain inhibitor JQ1 as novel therapeutic option for type II testicular germ cell tumours: The role of SOX2 and SOX17 in regulating germ cell tumour pluripotency," Rheinischen Friedrich-Wilhelms-Universität, 2019.

[191] T. Chekouo and S. E. Safo, "Bayesian Integrative Analysis and Prediction with Application to Atherosclerosis Cardiovascular Disease," *arXiv*, pp. 1–48, May 2020.

[192] H. Y. Lee, "Combined Proteomics and Metabolomics Study Reveals the TNF-*α* Regulated Metabolic Reprogramming of Estrogen Receptor/Progesterone Receptor Positive and Triple Negative Breast Cancer Cells," Seoul National University, 2018.

[193] R. Chiesa and M. Sallese, "Review: Protein misfolding diseases – the rare case of Marinesco-Sjögren syndrome," *Neuropathol. Appl. Neurobiol.*, vol. 46, no. 4, pp. 323–343, Jun. 2020.

[194] A. Ogura, M. Yoshida, M. Fukuzaki, and J. Sese, "*In vitro* homology search array comprehensively reveals highly conserved genes and their functional characteristics in non-sequenced species," *BMC Genomics*, vol. 11, no. Suppl 4, p. S9, 2010.

[195] A. A. Pulido, "Transcriptomic analysis of the response of the lactic acid bacteria *Lactococcus piscium* for the identification of possible adaptive genes during thermal shock in cold and warm temperatures," University of Helsinki, 2020.

[196] L. Cornacchione, "Mechanisms for pigmentation of *Porphyromonas gingivalis* and growth inhibition by yogurt-derived *Lactobacillus delbrueckii*," Tufts University, 2019.

[197] P. Aurass, B. Pless, K. Rydzewski, G. Holland, N. Bannert, and A. Flieger, "*bdhA-patD* Operon as a Virulence Determinant, Revealed by a Novel Large-Scale Approach for Identification of *Legionella pneumophila* Mutants Defective for Amoeba Infection," *Appl. Environ. Microbiol.*, vol. 75, no. 13, pp. 4506–4515, Jul. 2009.

[198] T. J. O'Connor, D. Boyd, M. S. Dorer, and R. R. Isberg, "Aggravating Genetic Interactions Allow a Solution to Redundancy in a Bacterial Pathogen," *Science (80-.).*, vol. 338, no. 6113, pp. 1440–1444, Dec. 2012.

[199] S. Rankin, Z. Li, and R. R. Isberg, "Macrophage-Induced Genes of *Legionella pneumophila*: Protection from Reactive Intermediates and Solute Imbalance during Intracellular Growth," *Infect. Immun.*, vol. 70, no. 7, pp. 3637–3648, Jul. 2002.

[200] D. Burstein *et al.*, "Genomic analysis of 38 *Legionella* species identifies large and diverse effector repertoires," *Nat. Genet.*, vol. 48, no. 2, pp. 167–175, Feb. 2016.

[201] R. L. Edwards, A. Bryan, M. Jules, K. Harada, C. Buchrieser, and M. S. Swanson, "Nicotinic Acid Modulates *Legionella pneumophila* Gene Expression and Induces Virulence Traits," *Infect. Immun.*, vol. 81, no. 3, pp. 945–955, Mar. 2013.

[202] A. Damianou *et al.*, "Essential roles for deubiquitination in *Leishmania* life cycle progression," *PLOS Pathog.*, vol. 16, no. 6, p. e1008455, Jun. 2020.

[203] L. Simon *et al.*, "Δ9-Tetrahydrocannabinol (Δ9-THC) Promotes Neuroimmune-Modulatory MicroRNA Profile in Striatum of *Simian Immunodeficiency Virus* (SIV)-Infected Macaques," *J. Neuroimmune Pharmacol.*, vol. 11, no. 1, pp. 192–213, Mar. 2016.

[204] H. Yang *et al.*, "Generation of haploid embryonic stem cells from *Macaca fascicularis* monkey parthenotes," *Cell Res.*, vol. 23, no. 10, pp. 1187–1200, Oct. 2013.

[205] J. Chen, H. Yamahachi, and C. D. Gilbert, "Experience-Dependent Gene Expression in Adult Visual Cortex," *Cereb. Cortex*, vol. 20, no. 3, pp. 650–660, Mar. 2010.

[206] M. G. Strillacci, S. P. Marelli, and G. Martinez-Velazquez, "Hybrid Versus Autochthonous Turkey Populations: Homozygous Genomic Regions Occurrences Due to Artificial and Natural Selection," *Animals*, vol. 10, no. 8, p. 1318, Jul. 2020.

[207] T. Fujishiro, U. Ermler, and S. Shima, "A possible iron delivery function of the dinuclear iron center of HcgD in [Fe]-hydrogenase cofactor biosynthesis," *FEBS Lett.*, vol. 588, no. 17, pp. 2789–2793, Aug. 2014.

[208] S.-M. Kuan *et al.*, "Crystallization and preliminary X-ray diffraction analysis of the Nif3-family protein MJ0927 from *Methanocaldococcus jannaschii*," *Acta Crystallogr. Sect. F Struct. Biol. Cryst. Commun.*, vol. 69, no. 1, pp. 80–82, Jan. 2013.

[209] S.-C. Chen *et al.*, "Crystal Structure of a Conserved Hypothetical Protein MJ0927 from *Methanocaldococcus jannaschii* Reveals a Novel Quaternary Assembly in the Nif3 Family," *Biomed Res. Int.*, vol. 2014, pp. 1–8, 2014.

[210] K. S. Makarova and E. V. Koonin, "Archaeal Ubiquitin-Like Proteins: Functional Versatility and Putative Ancestral Involvement in tRNA Modification Revealed by Comparative Genomic Analysis," *Archaea*, vol. 2010, pp. 1–10, 2010.

[211] T. Fujishiro *et al.*, "Identification of HcgC as a SAM-Dependent Pyridinol Methyltransferase in [Fe]-Hydrogenase Cofactor Biosynthesis," *Angew. Chemie Int. Ed.*, vol. 55, no. 33, pp. 9648–9651, Aug. 2016.

[212] L. Bai, T. Wagner, T. Xu, X. Hu, U. Ermler, and S. Shima, "A Water-Bridged H-Bonding Network Contributes to the Catalysis of the SAM-Dependent C-Methyltransferase HcgC," *Angew. Chemie Int. Ed.*, vol. 56, no. 36, pp. 10806–10809, Aug. 2017.

[213] T. Fujishiro, J. Kahnt, U. Ermler, and S. Shima, "Protein-pyridinol thioester precursor for biosynthesis of the organometallic acyl-iron ligand in [Fe]-hydrogenase cofactor," *Nat. Commun.*, vol. 6, no. 1, p. 6895, Nov. 2015.

[214] T. J. Lie, K. C. Costa, D. Pak, V. Sakesan, and J. A. Leigh, "Phenotypic evidence that the function of the [Fe]-hydrogenase Hmd in *Methanococcus*

maripaludis requires seven *hcg* (*hmd* co-occurring genes) but not *hmdII*," *FEMS Microbiol. Lett.*, vol. 343, no. 2, pp. 156–160, Jun. 2013.

[215] S. A. Kerns, "Anthracene-Scaffolded Model Complexes of [Fe]-Hydrogenase (Hmd) for Improved Functional Reactivity and Mechanistic Investigations of Biomimetic H_2 Activation and Hydride Transfer," The University of Texas at Austin, 2020.

[216] S. López-Madrigal, A. Latorre, M. Porcar, A. Moya, and R. Gil, "Mealybugs nested endosymbiosis: going into the 'matryoshka' system in *Planococcus citri* in depth," *BMC Microbiol.*, vol. 13, no. 1, p. 74, 2013.

[217] M. K. Parente, R. Rozen, S. H. Seeholzer, and J. H. Wolfe, "Integrated analysis of proteome and transcriptome changes in the mucopolysaccharidosis type VII mouse hippocampus," *Mol. Genet. Metab.*, vol. 118, no. 1, pp. 41–54, May 2016.

[218] R. A. Rachel, T. Li, and A. Swaroop, "Photoreceptor sensory cilia and ciliopathies: focus on CEP290, RPGR and their interacting proteins," *Cilia*, vol. 1, no. 1, p. 22, 2012.

[219] E. L. Huttlin *et al.*, "A Tissue-Specific Atlas of Mouse Protein Phosphorylation and Expression," *Cell*, vol. 143, no. 7, pp. 1174–1189, Dec. 2010.

[220] V. Giuffrida *et al.*, "Gene expression in mouse spermatogenesis during ontogenesis," *Int. J. Mol. Med.*, vol. 17, no. 3, pp. 523–528, Mar. 2006.

[221] M. Zuccotti *et al.*, "Maternal Oct-4 is a potential key regulator of the developmental competence of mouse oocytes," *BMC Dev. Biol.*, vol. 8, no. 1, p. 97, 2008.

[222] J. M. Bhasin, E. Chakrabarti, D.-Q. Peng, A. Kulkarni, X. Chen, and J. D. Smith, "Sex Specific Gene Regulation and Expression QTLs in Mouse Macrophages from a Strain Intercross," *PLoS One*, vol. 3, no. 1, p. e1435, Jan. 2008.

[223] G. D. Lively *et al.*, "Genetic Dependence of Central Corneal Thickness among Inbred Strains of Mice," *Investig. Opthalmology Vis. Sci.*, vol. 51, no. 1, p. 160, Jan. 2010.

[224] I. Paul *et al.*, "The ubiquitin ligase Cullin5SOCS2 regulates NDR1/STK38 stability and NF-κB transactivation," *Sci. Rep.*, vol. 7, no. 1, p. 42800, Mar. 2017.

[225] D.-M. Shin, C.-H. Lee, and H. C. Morse, "IRF8 Governs Expression of Genes Involved in Innate and Adaptive Immunity in Human and Mouse Germinal Center B Cells," *PLoS One*, vol. 6, no. 11, p. e27384, Nov. 2011.

[226] E. L. Johnson *et al.*, "Sphingolipid production by gut *Bacteroidetes* regulates glucose homeostasis," *BioRxiv*, Nov. 2019.

[227] L. Yan, X. Yao, D. Bachvarov, Z. Saifudeen, and S. S. El-Dahr, "Genome-wide analysis of gestational gene-environment interactions in the developing kidney," *Physiol. Genomics*, vol. 46, no. 17, pp. 655–670, Sep. 2014.

[228] M. Zamanian-Daryoush *et al.*, "Apolipoprotein A-I anti-tumor activity targets cancer cell metabolism," *Oncotarget*, vol. 11, no. 19, pp. 1777–1796, May 2020.

[229] N. McBrearty, "Short chain fatty acids (SCFAs) delay the pathogenesis of *Hepatitus B virus* (HBV)-associated hepatocellular carcinoma (HCC)," Temple University, 2019.

[230] Hladik *et al.*, "Combined Treatment with Low-Dose Ionizing Radiation and Ketamine Induces Adverse Changes in CA1 Neuronal Structure in Male Murine Hippocampi," *Int. J. Mol. Sci.*, vol. 20, no. 23, p. 6103, Dec. 2019.

[231] C. Stern, B. Schreier, A. Nolze, S. Rabe, S. Mildenberger, and M. Gekle, "Knockout of vascular smooth muscle EGF receptor in a mouse model prevents obesity-induced vascular dysfunction and renal damage *in vivo*," *Diabetologia*, vol. 63, no. 10, pp. 2218–2234, Oct. 2020.

[232] L. E. Bermudez, L. Danelishvili, L. Babrack, and T. Pham, "Evidence for genes associated with the ability of *Mycobacterium avium subsp. hominissuis* to escape apoptotic macrophages," *Front. Cell. Infect. Microbiol.*, vol. 5, no. AUG, pp. 1–8, Aug. 2015.

[233] A. Facciuolo, P. Gonzalez-Cano, S. Napper, P. J. Griebel, and L. M. Mutharia, "Marked Differences in Mucosal Immune Responses Induced in Ileal versus Jejunal Peyer's Patches to *Mycobacterium avium subsp. paratuberculosis* Secreted Proteins following Targeted Enteric Infection in Young Calves," *PLoS One*, vol. 11, no. 7, p. e0158747, Jul. 2016.

[234] A. Facciuolo, D. F. Kelton, and L. M. Mutharia, "Novel Secreted Antigens of *Mycobacterium paratuberculosis* as Serodiagnostic Biomarkers for Johne's Disease in Cattle," *Clin. Vaccine Immunol.*, vol. 20, no. 12, pp. 1783–1791, Dec. 2013.

[235] W. S. Kim, M.-K. Shin, and S. J. Shin, "MAP1981c, a Putative Nucleic Acid-Binding Protein, Produced by *Mycobacterium avium subsp. paratuberculosis*, Induces Maturation of Dendritic Cells and Th1-Polarization," *Front. Cell. Infect. Microbiol.*, vol. 8, no. JUN, Jun. 2018.

[236] A. A. T. Naqvi, F. Ahmad, and M. I. Hassan, "Identification of functional candidates amongst hypothetical proteins of *Mycobacterium leprae Br4923*, a causative agent of leprosy," *Genome*, vol. 58, no. 1, pp. 25–42, Jan. 2015.

[237] M. Boot *et al.*, "*iniBAC* induction Is Vitamin B12- and MutAB-dependent in *Mycobacterium marinum*," *J. Biol. Chem.*, vol. 291, no. 38, pp. 19800–19812, Sep. 2016.

[238] R. S. Gupta, B. Lo, and J. Son, "Phylogenomics and Comparative Genomic Studies Robustly Support Division of the *Genus Mycobacterium* into

an Emended Genus *Mycobacterium* and Four Novel Genera," *Front. Microbiol.*, vol. 9, no. FEB, pp. 1–41, Feb. 2018.

[239] R. Gupta, D. Chatterjee, M. S. Glickman, and S. Shuman, "Division of labor among *Mycobacterium smegmatis* RNase H enzymes: RNase H1 activity of RnhA or RnhC is essential for growth whereas RnhB and RnhA guard against killing by hydrogen peroxide in stationary phase," *Nucleic Acids Res.*, vol. 45, no. 1, pp. 1–14, Jan. 2017.

[240] J. Amon, "Mining the genomes of *Actinomycetes*: Identification of metabolic pathways and regulatory networks," Friedrich-Alexander-Universität Erlangen-Nürnberg, 2010.

[241] X. Li *et al.*, "Transcriptome Landscape of *Mycobacterium smegmatis*," *Front. Microbiol.*, vol. 8, no. DEC, pp. 1–16, Dec. 2017.

[242] N. L. Steyn, "Nucleoid Gene Regulation in *Mycobacteria*," Stellenbosch University, 2017.

[243] A. Kumar and S. Karthikeyan, "Crystal structure of the MSMEG_4306 gene product from *Mycobacterium smegmatis*," *Acta Crystallogr. Sect. F Struct. Biol. Commun.*, vol. 74, no. 3, pp. 166–173, Mar. 2018.

[244] J. L. McKenzie *et al.*, "A VapBC Toxin-Antitoxin Module Is a Posttranscriptional Regulator of Metabolic Flux in *Mycobacteria*," *J. Bacteriol.*, vol. 194, no. 9, pp. 2189–2204, May 2012.

[245] A. E. Minias *et al.*, "RNase HI Is Essential for Survival of *Mycobacterium smegmatis*," *PLoS One*, vol. 10, no. 5, p. e0126260, May 2015.

[246] S. Raman, X. Puyang, T.-Y. Cheng, D. C. Young, D. B. Moody, and R. N. Husson, "*Mycobacterium tuberculosis* SigM Positively Regulates Esx Secreted Protein and Nonribosomal Peptide Synthetase Genes and Down Regulates Virulence-Associated Surface Lipid Synthesis," *J. Bacteriol.*, vol. 188, no. 24, pp. 8460–8468, Dec. 2006.

[247] H. A. Watkins and E. N. Baker, "Cloning, expression, purification and preliminary crystallographic analysis of the RNase HI domain of the *Mycobacterium tuberculosis* protein Rv2228c as a maltose-binding protein fusion," *Acta Crystallogr. Sect. F Struct. Biol. Cryst. Commun.*, vol. 64, no. 8, pp. 746–749, Aug. 2008.

[248] H. A. Watkins and E. N. Baker, "Structural and Functional Characterization of an RNase HI Domain from the Bifunctional Protein Rv2228c from *Mycobacterium tuberculosis*," *J. Bacteriol.*, vol. 192, no. 11, pp. 2878–2886, Jun. 2010.

[249] Z. Ditse, M. H. Lamers, and D. F. Warner, "DNA Replication in *Mycobacterium tuberculosis*," *Microbiol. Spectr.*, vol. 5, no. 2, Apr. 2017.

[250] C. Duncan *et al.*, "Whole transcriptomic and proteomic analyses of an isogenic *M. tuberculosis* clinical strain with a naturally occurring 15 Kb genomic deletion," *PLoS One*, vol. 12, no. 6, p. e0179996, Jun. 2017.

[251] L. Danelishvili, M. Wu, L. S. Young, and L. E. Bermudez, "Genomic Approach to Identifying the Putative Target of and Mechanisms of Resistance to Mefloquine in *Mycobacteria*," *Antimicrob. Agents Chemother.*, vol. 49, no. 9, pp. 3707–3714, Sep. 2005.

[252] Y. Ding, F. Ma, Z. Wang, and B. Li, "Characteristics of the Vδ2 CDR3 Sequence of Peripheral γδ T Cells in Patients with Pulmonary Tuberculosis and Identification of a New Tuberculosis-Related Antigen Peptide," *Clin. Vaccine Immunol.*, vol. 22, no. 7, pp. 761–768, Jul. 2015.

[253] A. Minias, P. Minias, B. Czubat, and J. Dziadek, "Purifying Selective Pressure Suggests the Functionality of a Vitamin B12 Biosynthesis Pathway in a Global Population of *Mycobacterium tuberculosis*," *Genome Biol. Evol.*, vol. 10, no. 9, pp. 2326–2337, Sep. 2018.

[254] A. J. Martinot, "Microbial Offense vs Host Defense: Who Controls the TB Granuloma?," *Vet. Pathol.*, vol. 55, no. 1, pp. 14–26, Jan. 2018.

[255] M. Sarker *et al.*, "Combining Cheminformatics Methods and Pathway Analysis to Identify Molecules with Whole-Cell Activity Against *Mycobacterium Tuberculosis*," *Pharm. Res.*, vol. 29, no. 8, pp. 2115–2127, Aug. 2012.

[256] X. Fang, A. Wallqvist, and J. Reifman, "Development and analysis of an *in vivo*-compatible metabolic network of *Mycobacterium tuberculosis*," *BMC Syst. Biol.*, vol. 4, no. 1, p. 160, 2010.

[257] T. Hossain, H. S. Deter, and N. C. Butzin, "Antibiotic tolerance , persistence, and resistance of the evolved minimal cell, *Mycoplasma mycoides JCVI-Syn3B*," *bioRxiv*, pp. 1–18, 2020.

[258] L. A. Jackson, J.-C. Pan, M. W. Day, and D. W. Dyer, "Control of RNA Stability by NrrF, an Iron-Regulated Small RNA in *Neisseria gonorrhoeae*," *J. Bacteriol.*, vol. 195, no. 22, pp. 5166–5173, Nov. 2013.

[259] S. Sainsbury, J. Ren, N. J. Saunders, D. I. Stuart, and R. J. Owens, "Structure of the regulatory domain of the LysR family regulator NMB2055 (MetR-like protein) from *Neisseria meningitidis*," *Acta Crystallogr. Sect. F Struct. Biol. Cryst. Commun.*, vol. 68, no. 7, pp. 730–737, Jul. 2012.

[260] A. S. Punekar, J. Porter, S. B. Carr, and S. E. V. Phillips, "Structural basis for DNA recognition by the transcription regulator MetR," *Acta Crystallogr. Sect. F Struct. Biol. Commun.*, vol. 72, no. 6, pp. 417–426, Jun. 2016.

[261] W. Dong *et al.*, "Systems Biology of the Clock in *Neurospora crassa*," *PLoS One*, vol. 3, no. 8, p. e3105, Aug. 2008.

[262] C. Wang, Y. Song, N. Tang, G. Zhang, S. O. Leclercq, and J. Feng, "The Shared Resistome of Human and Pig Microbiota Is Mobilized by Distinct Genetic Elements," *Appl. Environ. Microbiol.*, vol. 87, no. 5, Dec. 2020.

[263] J. Tian, J. Du, J. Han, X. Bao, X. Song, and Z. Lu, "Proteomics reveals the preliminary physiological states of the spotted seal (*Phoca largha*) pups," *Sci. Rep.*, vol. 10, no. 1, p. 18727, Dec. 2020.

[264] E. Turlin *et al.*, "Proteome analysis of the phenotypic variation process in *Photorhabdus luminescens*," *Proteomics*, vol. 6, no. 9, pp. 2705–2725, May 2006.

[265] P. H. Degnan, "Genome sequence of *Blochmannia pennsylvanicus* indicates parallel evolutionary trends among bacterial mutualists of insects," *Genome Res.*, vol. 15, no. 8, pp. 1023–1033, Aug. 2005.

[266] R. M. E. McKenzie *et al.*, "Metabolome variations in the *Porphyromonas gingivalis vimA* mutant during hydrogen peroxide-induced oxidative stress," *Mol. Oral Microbiol.*, vol. 30, no. 2, pp. 111–127, Apr. 2015.

[267] C. D. Schlenker, "The Characterization of PG0228 in *Porphyromonas gingivalis W83*," University of Toledo, 2011.

[268] B. R. Belvin, Q. Gui, J. A. Hutcherson, and J. P. Lewis, "The *Porphyromonas gingivalis* hybrid cluster protein Hcp is required for growth with nitrite and survival with host cells," *Infect. Immun.*, vol. 87, no. 4, Jan. 2019.

[269] A. Rahmani-Badi, S. Sepehr, H. Fallahi, and S. Heidari-Keshel, "Erratum: Exposure of *E. coli* to DNA-Methylating Agents Impairs Biofilm Formation and Invasion of Eukaryotic Cells via Down Regulation of the N-Acetylneuraminate Lyase NanA," *Front. Microbiol.*, vol. 7, no. APR, pp. 1–13, Mar. 2016.

[270] M. Starkey *et al.*, "*Pseudomonas aeruginosa* Rugose Small-Colony Variants Have Adaptations That Likely Promote Persistence in the Cystic Fibrosis Lung," *J. Bacteriol.*, vol. 191, no. 11, pp. 3492–3503, Jun. 2009.

[271] L. V. Wilkinson, "Peptide 1018 inhibits swarming motility and dysregulates transcriptional regulators of swarming in *Pseudomonas aeruginosa*," The University of British Columbia, 2018.

[272] W. Gooderham, "Regulation of Virulence and Antimicrobial Peptide Resistance in *Pseudomonas Aeruginosa*," The University of British Columbia, 2008.

[273] A. Manan, Z. Bazai, J. Fan, H. Yu, and L. Li, "The Nif3-family protein YqfO03 from *Pseudomonas syringae MB03* has multiple nematicidal activities against *Caenorhabditis elegans* and *Meloidogyne incognita*," *Int. J. Mol. Sci.*, vol. 19, no. 12, p. 3915, Dec. 2018.

[274] A. Bashir, T. Tian, X. Yu, C. Meng, M. Ali, and L. Li, "Pyoverdine-Mediated Killing of *Caenorhabditis elegans* by *Pseudomonas syringae MB03* and the Role of Iron in Its Pathogenicity," *Int. J. Mol. Sci.*, vol. 21, no. 6, p. 2198, Mar. 2020.

[275] Meshram Y and Gore D, "Deciphering the enzyme coding ability in *Pseudomonas stutzeri ATCC17588* hypothetical proteins by bioinformatics approach," *J. Pharm. Res.*, vol. 13, no. 1, pp. 30–35, 2019.

[276] Y. Cherkas, M. K. McMillian, D. Amaratunga, N. Raghavan, and J. C. Sasaki, "ABC gene-ranking for prediction of drug-induced cholestasis in rats," *Toxicol. Reports*, vol. 3, pp. 252–261, 2016.

[277] R. Massart *et al.*, "Role of DNA Methylation in the Nucleus Accumbens in Incubation of Cocaine Craving," *J. Neurosci.*, vol. 35, no. 21, pp. 8042–8058, May 2015.

[278] S. Malan-Müller, V. B. C. de Souza, W. M. U. Daniels, S. Seedat, M. D. Robinson, and S. M. J. Hemmings, "Shedding Light on the Transcriptomic Dark Matter in Biological Psychiatry: Role of Long Noncoding RNAs in D-cycloserine-Induced Fear Extinction in Posttraumatic Stress Disorder," *Omi. A J. Integr. Biol.*, vol. 24, no. 6, pp. 352–369, Jun. 2020.

[279] M. C. Harrison, A. M. Chernyshova, and G. J. Thompson, "No obvious transcriptome-wide signature of indirect selection in termites," *J. Evol. Biol.*, no. July 2020, p. jeb.13749, Dec. 2020.

[280] L. F. Moreno *et al.*, "Genomic Understanding of an Infectious Brain Disease from the Desert," *G3: Genes|Genomes|Genetics*, vol. 8, no. 3, pp. 909–922, Mar. 2018.

[281] J. a Martens, J. Genereaux, C. J. Brandl, and A. Saleh, "Transcriptional Activation by Yeast PDR1p Is Inhibited by Its Association with Transcriptional Activation by Yeast PDR1p Is Inhibited by Its Association with NGG1p/ADA3p *," *J. Biol. Chem.*, vol. 271, no. 27, pp. 15884–15890, 1996.

[282] R. Page, W. Peti, I. A. Wilson, R. C. Stevens, and K. Wuthrich, "NMR screening and crystal quality of bacterially expressed prokaryotic and eukaryotic proteins in a structural genomics pipeline," *Proc. Natl. Acad. Sci.*, vol. 102, no. 6, pp. 1901–1905, Feb. 2005.

[283] Y. Li, K. sum Lam, N. Dasgupta, and P. Ye, "A yeast's eye view of mammalian reproduction: cross-species gene co-expression in meiotic prophase.," *BMC Syst. Biol.*, vol. 4, p. 125, 2010.

[284] J. Reinders, R. P. Zahedi, N. Pfanner, C. Meisinger, and A. Sickmann, "Toward the complete yeast mitochondrial proteome: Multidimensional separation techniques for mitochondrial proteomics," *J. Proteome Res.*, vol. 5, no. 7, pp. 1543–1554, 2006.

[285] S. Ghaemmaghami *et al.*, "Global analysis of protein expression in yeast," *Nature*, vol. 425, no. 6959, pp. 737–741, Oct. 2003.

[286] J. K. Risler, A. E. Kenny, R. J. Palumbo, E. R. Gamache, and M. J. Curcio, "Host co-factors of the retrovirus-like transposon Ty1," *Mob. DNA*, vol. 3, no. 1, p. 12, 2012.

[287] K. L. Ho *et al.*, "A Role for the Budding Yeast Separase, Esp1, in Ty1 Element Retrotransposition," *PLOS Genet.*, vol. 11, no. 3, p. e1005109, Mar. 2015.

[288] M. Breker, M. Gymrek, O. Moldavski, and M. Schuldiner, "LoQAtE—Localization and Quantitation atlas of the yeast proteome. A new tool for multiparametric dissection of single-protein behavior in response to biological perturbations in yeast," *Nucleic Acids Res.*, vol. 42, no. D1, pp. D726–D730, Jan. 2014.

[289] C. Peng *et al.*, "Proteomics insights into the responses of *Saccharomyces cerevisiae* during mixed-culture alcoholic fermentation with *Lachancea thermotolerans*," *FEMS Microbiol. Ecol.*, vol. 95, no. 9, pp. 1–16, Sep. 2019.

[290] R. Jothi, P. F. Cherukuri, A. Tasneem, and T. M. Przytycka, "Co-evolutionary Analysis of Domains in Interacting Proteins Reveals Insights into Domain–Domain Interactions Mediating Protein–Protein Interactions," *J. Mol. Biol.*, vol. 362, no. 4, pp. 861–875, Sep. 2006.

[291] J. C. Rosenbaum *et al.*, "Disorder Targets Misorder in Nuclear Quality Control Degradation: A Disordered Ubiquitin Ligase Directly Recognizes Its Misfolded Substrates," *Mol. Cell*, vol. 41, no. 1, pp. 93–106, Jan. 2011.

[292] S. A. Nelson and J. A. Cooper, "A Novel Pathway that Coordinates Mitotic Exit with Spindle Position," *Mol. Biol. Cell*, vol. 18, no. 9, pp. 3440–3450, Sep. 2007.

[293] G. P. Samper, "The Crabtree effect shapes *Saccharomyces cerevisiae* lag phase," Arenburg Doctoral School, 2019.

[294] M. Morgenstern *et al.*, "Definition of a High-Confidence Mitochondrial Proteome at Quantitative Scale," *Cell Rep.*, vol. 19, no. 13, pp. 2836–2852, Jun. 2017.

[295] S. Karniely, A. Rayzner, E. Sass, and O. Pines, "α-Complementation as a probe for dual localization of mitochondrial proteins," *Exp. Cell Res.*, vol. 312, no. 19, pp. 3835–3846, Nov. 2006.

[296] V. Shakya, W. Barbeau, T. Xiao, C. Knutson, and A. Hughes, "The nucleus is a quality control center for non-imported mitochondrial proteins," *bioRxiv*, 2020.

[297] O. Phuphisut *et al.*, "Transcriptomic analysis of male and female *Schistosoma mekongi* adult worms," *Parasit. Vectors*, vol. 11, no. 1, p. 504, Dec. 2018.

[298] J. Hayles *et al.*, "A genome-wide resource of cell cycle and cell shape genes of fission yeast," *Open Biol.*, vol. 3, no. 5, p. 130053, May 2013.

[299] O. Dudin, L. Merlini, F. O. Bendezú, R. Groux, V. Vincenzetti, and S. G. Martin, "A systematic screen for morphological abnormalities during fission yeast sexual reproduction identifies a mechanism of actin aster formation for cell fusion," *PLOS Genet.*, vol. 13, no. 4, p. e1006721, Apr. 2017.

[300] X. Pan *et al.*, "Identification of novel genes involved in DNA damage response by screening a genome-wide *Schizosaccharomyces pombe* deletion library," *BMC Genomics*, vol. 13, no. 1, p. 662, 2012.

[301] J. Prahlad *et al.*, "The DUF328 family member YaaA is a DNA-binding protein with a novel fold," *J. Biol. Chem.*, vol. 295, no. 41, pp. 14236–14247, Oct. 2020.

[302] R. Munoz, H. Teeling, R. Amann, and R. Rosselló-Móra, "Ancestry and adaptive radiation of *Bacteroidetes* as assessed by comparative genomics," *Syst. Appl. Microbiol.*, vol. 43, no. 2, p. 126065, Mar. 2020.

[303] B. Bagautdinov, Y. Matsuura, S. Bagautdinova, N. Kunishima, and K. Yutani, "Structure of putative CutA1 from *Homo sapiens* determined at 2.05 Å resolution," *Acta Crystallogr. Sect. F Struct. Biol. Cryst. Commun.*, vol. 64, no. 5, pp. 351–357, May 2008.

[304] K. S. Saikatendu, X. Zhang, L. Kinch, M. Leybourne, N. V. Grishin, and H. Zhang, "Structure of a conserved hypothetical protein SA1388 from *S. aureus* reveals a capped hexameric toroid with two PII domain lids and a dinuclear metal center," *BMC Struct. Biol.*, vol. 6, p. 27, Dec. 2006.

[305] P. M. van Diemen *et al.*, "The *S. aureus* 4-oxalocrotonate tautomerase SAR1376 enhances immune responses when fused to several antigens," *Sci. Rep.*, vol. 7, no. 1, p. 1745, Dec. 2017.

[306] A. Yahashiri, M. A. Jorgenson, and D. S. Weiss, "The SPOR Domain, a Widely Conserved Peptidoglycan Binding Domain That Targets Proteins to the Site of Cell Division," *J. Bacteriol.*, vol. 199, no. 14, pp. 1–10, Jul. 2017.

[307] K. B. Williams, A. Yahashiri, S. J. R. Arends, D. L. Popham, C. A. Fowler, and D. S. Weiss, "Nuclear Magnetic Resonance Solution Structure of the Peptidoglycan-Binding SPOR Domain from *Escherichia coli* DamX: Insights into Septal Localization," *Biochemistry*, vol. 52, no. 4, pp. 627–639, Jan. 2013.

[308] K. L. Anderson *et al.*, "Characterizing the effects of inorganic acid and alkaline shock on the *Staphylococcus aureus* transcriptome and messenger RNA turnover," *FEMS Immunol. Med. Microbiol.*, vol. 60, no. 3, pp. 208–250, Dec. 2010.

[309] P. M. Dunman *et al.*, "Transcription Profiling-Based Identification of *Staphylococcus aureus* Genes Regulated by the *agr* and/or *sarA* Loci," *J. Bacteriol.*, vol. 183, no. 24, pp. 7341–7353, Dec. 2001.

[310] D. Mary Rajathei and S. Selvaraj, "Analysis of sequence repeats of proteins in the PDB," *Comput. Biol. Chem.*, vol. 47, pp. 156–166, Dec. 2013.

[311] N. H. Kwon *et al.*, "Staphylococcal cassette chromosome *mec* (SCC*mec*) characterization and molecular analysis for methicillin-resistant *Staphylococcus aureus* and novel SCC*mec* subtype IVg isolated from bovine milk in Korea," *J. Antimicrob. Chemother.*, vol. 56, no. 4, pp. 624–632, Oct. 2005.

[312] K. F. Bruce, P. Warrener, J. McLarnan, and K. Hou, "Random transposon insertion in *Staphylococcus aureus* and use thereof to identify essential genes," US8173363B2, 2012.

[313] J. Drummelsmith, E. Winstall, M. G. Bergeron, G. G. Poirier, and M. Ouellette, "Comparative Proteomics Analyses Reveal a Potential Biomarker for the Detection of Vancomycin-Intermediate *Staphylococcus aureus* Strains," *J. Proteome Res.*, vol. 6, no. 12, pp. 4690–4702, Dec. 2007.

[314] P. Bleichert *et al.*, "Mutant Strains of Escherichia coli and Methicillin-Resistant *Staphylococcus aureus* Obtained by Laboratory Selection To Survive on Metallic Copper Surfaces," *Appl. Environ. Microbiol.*, vol. 87, no. 1, pp. 1–21, Dec. 2020.

[315] H. J. Bootsma, P. J. Burghout, P. W. M. Hermans, J. J. E. Bijlsma, O. P. Kuipers, and T. G. Kloosterman, "Virulence factors of *Streptococcus pnuemoniae*," US 20100183596 A1, 2010.

[316] P. W. M. Hermans, H. J. Bootsma, P. J. Burghout, J. J. E. Bijlsma, O. P. Kuipers, and T. G. Kloosterman, "New virulence factors of *Streptococcus pneumoniae*," WO 2008127094 A2, 2008.

[317] A. B. Dalia, "Characterization of Bacterial Factors that Promote Resistance to Complement-mediated Opsonophagocytic Killing and Lysis," University of Pennsylvania, 2011.

[318] L. K. Mahdi, E. Ebrahimie, D. L. Adelson, J. C. Paton, and A. D. Ogunniyi, "A Transcription Factor Contributes to Pathogenesis and Virulence in *Streptococcus pneumoniae*," *PLoS One*, vol. 8, no. 8, p. e70862, Aug. 2013.

[319] H. M. Ta and K. K. Kim, "Crystal structure of *Streptococcus pneumoniae* Sp1610, a putative tRNA methyltransferase, in complex with S-adenosyl-L-methionine," *Protein Sci.*, vol. 19, no. 3, pp. 617–624, Mar. 2010.

[320] K. L. Constantine *et al.*, "Structural and functional characterization of CFE88: Evidence that a conserved and essential bacterial protein is a methyltransferase," *Protein Sci.*, vol. 14, no. 6, pp. 1472–1484, Jan. 2009.

[321] L. Happonen *et al.*, "A quantitative *Streptococcus pyogenes*–human protein–protein interaction map reveals localization of opsonizing antibodies," *Nat. Commun.*, vol. 10, no. 1, p. 2727, Dec. 2019.

[322] U. Resch *et al.*, "A Two-Component Regulatory System Impacts Extracellular Membrane-Derived Vesicle Production in Group A *Streptococcus*," *MBio*, vol. 7, no. 6, pp. 1–10, Dec. 2016.

[323] J. A. Freiberg *et al.*, "Global Analysis and Comparison of the Transcriptomes and Proteomes of Group A *Streptococcus* Biofilms," *mSystems*, vol. 1, no. 6, pp. 1–19, Dec. 2016.

[324] M. T. Alam, E. Takano, and R. Breitling, "Prioritizing orphan proteins for further study using phylogenomics and gene expression profiles in *Streptomyces coelicolor*," *BMC Res. Notes*, vol. 4, no. 1, p. 325, Dec. 2011.

[325] L. Kaysser, S. Siebenberg, B. Kammerer, and B. Gust, "Analysis of the liposidomycin gene cluster leads to the identification of new caprazamycin derivatives," *ChemBioChem*, vol. 11, no. 2, pp. 191–196, 2010.

[326] A. Hart, M. P. Cortés, M. Latorre, and S. Martinez, "Codon usage bias reveals genomic adaptations to environmental conditions in an acidophilic consortium," *PLoS One*, vol. 13, no. 5, p. e0195869, May 2018.

[327] J. P. Nowlan, "Quantitative PCR detection of *Tenacibaculum maritimum* and *Tenacibaculum dicentrarchi* at netpen sites in British Columbia (Canada)," The University of Guelph, 2020.

[328] J. P. Nowlan, J. S. Lumsden, and S. Russell, "Advancements in Characterizing *Tenacibaculum* Infections in Canada," *Pathogens*, vol. 9, no. 12, p. 1029, Dec. 2020.

[329] C. L. Brosnahan, J. S. Munday, H. J. Ha, M. Preece, and J. B. Jones, "New Zealand rickettsia-like organism (NZ-RLO) and *Tenacibaculum maritimum*: Distribution and phylogeny in farmed Chinook salmon (*Oncorhynchus tshawytscha*)," *J. Fish Dis.*, vol. 42, no. 1, pp. 85–95, Jan. 2019.

[330] C. Brosnahan, "Diagnostic investigation into summer mortality events of farmed Chinook salmon (*Oncorhynchus tshawytscha*) in New Zealand," Massey University, Manawatū, University of New Zealand, 2020.

[331] D. Giovannelli *et al.*, "Insight into the evolution of microbial metabolism from the deep-branching bacterium, *Thermovibrio ammonificans*," *Elife*, vol. 6, no. 848, Apr. 2017.

[332] F. Tomoike, T. Wakamatsu, N. Nakagawa, S. Kuramitsu, and R. Masui, "Crystal structure of the conserved hypothetical protein TTHA1606

from *Thermus thermophilus HB8*," *Proteins Struct. Funct. Bioinforma.*, vol. 76, no. 1, pp. 244–248, Jul. 2009.

[333] M. W. Van Dyke, M. D. Beyer, E. Clay, K. J. Hiam, J. L. McMurry, and Y. Xie, "Identification of Preferred DNA-Binding Sites for the *Thermus thermophilus* Transcriptional Regulator SbtR by the Combinatorial Approach REPSA," *PLoS One*, vol. 11, no. 7, p. e0159408, Jul. 2016.

[334] A. A. T. Naqvi, M. Shahbaaz, F. Ahmad, and M. I. Hassan, "Identification of Functional Candidates amongst Hypothetical Proteins of *Treponema pallidum ssp. pallidum*," *PLoS One*, vol. 10, no. 4, p. e0124177, Apr. 2015.

[335] N. Crapoulet, P. Barbry, D. Raoult, and P. Renesto, "Global Transcriptome Analysis of *Tropheryma whipplei* in Response to Temperature Stresses," *J. Bacteriol.*, vol. 188, no. 14, pp. 5228–5239, Jul. 2006.

[336] Austin Willis, "Comparing the Genome Expression Profiles of *Verrucomicrobium sp. Strain TAV2* Cells Grown Under Two Different Oxygen Concentrations," The University of Texas at Arlington, 2010.

[337] K. L. Meibom, X. B. Li, A. T. Nielsen, C.-Y. Wu, S. Roseman, and G. K. Schoolnik, "The *Vibrio cholerae* chitin utilization program," *Proc. Natl. Acad. Sci.*, vol. 101, no. 8, pp. 2524–2529, Feb. 2004.

[338] Y. Fu, M. K. Waldor, and J. J. Mekalanos, "Tn-Seq Analysis of *Vibrio cholerae* Intestinal Colonization Reveals a Role for T6SS-Mediated Antibacterial Activity in the Host," *Cell Host Microbe*, vol. 14, no. 6, pp. 652–663, Dec. 2013.

[339] J. Huang *et al.*, "Exploration of the effects of a *degS* mutant on the growth of *Vibrio cholerae* and the global regulatory function of *degS* by RNA sequencing," *PeerJ*, vol. 7, no. 10, p. e7959, Oct. 2019.

[340] W. Zhao, F. Caro, W. Robins, and J. J. Mekalanos, "Antagonism toward the intestinal microbiota and its effect on *Vibrio cholerae* virulence," *Science (80-.).*, vol. 359, no. 6372, pp. 210–213, Jan. 2018.

[341] B. W. Jones and M. K. Nishiguchi, "Differentially expressed genes reveal adaptations between free-living and symbiotic niches of *Vibrio fischeri* in a fully established mutualism," *Can. J. Microbiol.*, vol. 52, no. 12, pp. 1218–1227, Dec. 2006.

[342] C. R. DeLoney, T. M. Bartley, and K. L. Visick, "Role for Phosphoglucomutase in *Vibrio fischeri-Euprymna scolopes* Symbiosis," *J. Bacteriol.*, vol. 184, no. 18, pp. 5121–5129, Sep. 2002.

[343] V. Ducret *et al.*, "Transcriptomic analysis of the trade-off between endurance and burst-performance in the frog *Xenopus allofraseri*," *HAL*, 2021.

[344] W. T. Boswell *et al.*, "Exposure to 4100 K fluorescent light elicits sex specific transcriptional responses in *Xiphophorus maculatus* skin," *Comp. Biochem. Physiol. Part C Toxicol. Pharmacol.*, vol. 208, no. 3, pp. 96–104, Jun. 2018.

[345] G. Qijing and Y. Zhang, "NIF3 类超家族蛋白," *Chinese J. Cell Biol.*, vol. 29, pp. 816–820, 2007.

[346] Z. Liu, B. Tang, X. Yan, and J. Guo, "Expression profiles of genes in wild-type DJ-1 and L10P mutant DJ-1 in monoclonal cell strains," *Chinese J. Contemp. Neurol. Neurosurg.*, vol. 13, no. 7, 2013.

[347] K. Avican *et al.*, "RNA Atlas of Human Bacterial Pathogens Uncovers Stress Dynamics Linked to Infection," *bioRxiv*, 2020.

[348] C. E. Cook, O. Stroe, G. Cochrane, E. Birney, and R. Apweiler, "The European Bioinformatics Institute in 2020: Building a global infrastructure of interconnected data resources for the life sciences," *Nucleic Acids Res.*, vol. 48, no. D1, pp. D17–D23, 2020.
